# Supplementary material for: Three-component radical homo Mannich reaction
Source: Nat Commun. 2021 Feb 12;12:1006. doi: 10.1038/s41467-021-21303-3 (PMC7880990; doi:10.1038/s41467-021-21303-3)
Supplement: Supplementary file 1 — Supplementary Information [file 41467_2021_21303_MOESM1_ESM.pdf]

Supplementary information for

Three-component radical homo Mannich reaction

Shuai Shi[1], Wenting Qiu[1], Pannan Miao[1], Ruining Li[1], Xianfeng Lin[1] & Zhankui Sun[1*]

[1]Shanghai Key Laboratory for Molecular Engineering of Chiral Drugs, School of Pharmacy, Shanghai Jiao Tong University, No. 800 Dongchuan Rd., Shanghai 200240, China.

✉email: zksun@sjtu.edu.cn

Contents

1. Materials and general experimental protocols S3
2. General procedure for the synthesis of substrates S4
3. Optimization of the reaction conditions S7
4. General procedure for the radical homo Mannich reactions S9
5. Characterization data for all key compounds S10
6. Scale-up reaction S48
7. Mechanistic studies S49
8. Spectral data S51
9. Supplementary References S139

Supplementary Methods

1. Materials and general experimental protocols

All the reactions were run under nitrogen atmosphere in oven-dried glassware unless otherwise stated. Commercial chemicals were used as supplied without further purification. Commercial anhydrous dichloromethane employed in photoreaction were used as supplied and stored with 4Å molecular sieves (MS). The other solvents were used directly from commercial reagents. Particle 4 Å MS were activated before use by 2-hour heating (200 ^{o}C) under high-vacuum. The radical initiator di-tert-butyl peroxide (DTBP) was used directly without further manipulation. Unless otherwise noted, all solvents in reagent grade or analytical grade were used as received.

Organic solutions were concentrated under reduced pressure on an IKA rotary evaporator using a water bath. Chromatographic purification of products was accomplished using force-flow chromatography on silica gel (200-300 mesh) according to the method of Still[1]. Thin-layer chromatography (TLC) was performed on 250 µm silica gel plates. TLC visualization was performed by fluorescence quenching or iodine stain. All key compounds were characterized by ^{1}H NMR, ^{13}C NMR, FT-IR and HRMS. ^{1}H NMR spectra were recorded on an Agilent NMR Systems 400 MHz Spectrometer (^{1}H NMR at 400 MHz) and are internally referenced to residual protic $CDCl_3$ (δ 7.26 ppm). Data for ^{1}H NMR are reported as follows: chemical shift (δ ppm), multiplicity (s = singlet, d = doublet, t = triplet, q = quartet, m = multiplet, dd = doublet of doublets, dt = doublet of triplets, br = broad), coupling constant (Hz), and assignment. Infrared spectra (FT-IR) were recorded by Thermo Scientific™ Nicolet iS5 Fourier transform Spectrometer equipped with ATR and analyzed as thin films. Absorption maxima (ν_{max}) were reported in wavenumbers (cm^{-1}) and characteristic peaks were defined (s = strong, br = broad). ^{13}C NMR spectra were recorded on an Agilent NMR Systems 400 MHz Spectrometer (^{13}C NMR at 101 MHz) and data are reported in terms of chemical shift relative to $CDCl_3$ (77.16 ppm). ^{19}F NMR spectra were recorded on an Agilent NMR Systems 400 MHz Spectrometer (^{19}F NMR at 376 MHz) HRMS: electrospray ionization mass (ESI-MS) was performed on Agilent 6210 Series TOF MS. GC-MS analyses were performed on a GC-MS with an EI mode. LC-MS spectra were acquired by Acquity^{TM} UPLC/Xevo-G2-XS-QTOF-MS using C18 column (150mm×2.1mm, 1.7μm). HPLC: analytical revered-phase HPLC was performed on Agilent (1200 series HPLC system) instrument employing an analytical C18 column (250 × 4.6 mm, 5 μm). Mobile phases of HPLC are as followed: solvent A: 0.1% TFA (v/v) in acetonitrile; Solvent B: 0.1% TFA (v/v) in water. The 40W household bulb were directly purchased from supermarket.

2. General procedure for the synthesis of substrates

General protocol A for the synthesis of thiols

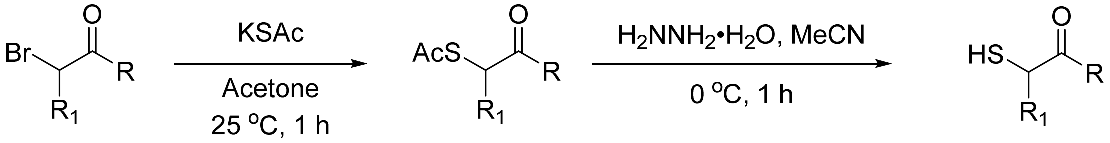

Supplementary Fig. 1 Preparation of thiols from commercial compounds in two steps.[2]

General procedure A for the preparation of thiols:

To a round bottomed flask with starting material (8.73 mmol) in acetone (87 mL) was added potassium thioacetate (1.1 equiv, 1.10 g, 9.60 mmol). The resulting mixture was stirred at room temperature for 1 hour. The mixture was filtered and the solvent was removed *in vacuo*. The resulting residue was distributed between water (100 mL) and DCM (300 mL). The organic layer was washed with brine (150 mL), dried with Na_2SO_4, concentrated and purified to provide the desired thioester.

To the round bottomed flask with thioester (5 mmol) in acetonitrile (60 mL) was added 35% aqueous solution of hydrazine hydrate (1.5 equiv, 0.7 mL, 7.5 mmol) by syringe under N_2. The reaction mixture was stirred 1 hour before it was quenched with HCl (10 mL, 1 M). The reaction mixture was extracted with DCM (50 mL×3). The combined organic layer was washed with HCl (50 mL, 1 M) and brine (80 mL), dried over Na_2SO_4. concentrated and purified by flash chromatography (10% ethyl acetate/hexanes) to give the desired thiol.

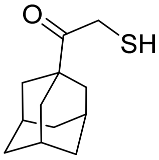

1-((3*r*,5*r*,7*r*)-adamantan-1-yl)-2-mercaptoethan-1-one (a) was prepared from 1-((3*r*,5*r*,7*r*)-adamantan-1-yl)-2-bromoethan-1-one according to the general procedures as a white solid (0.63 g, 60% yield). ^{1}H NMR (400 MHz, $CDCl_3$) δ 3.51 (d, J = 6.7 Hz, 2H), 2.08 - 1.92 (m, 4H), 1.81 (d, J = 2.8 Hz, 6H), 1.74 - 1.63 (m, 6H). ^{13}C NMR (101 MHz, $CDCl_3$) δ 209.4, 46.7, 38.6, 36.5, 29.8, 28.0. HRMS (ESI) calcd for $C_{12}H_{19}OS$ $[M + H]^+$ m/z = 211.1151, found: 211.1153. The NMR spectra matches that of previously reported literature.[2]

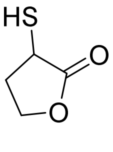

3-mercaptodihydrofuran-2(3*H*)-one (b) was prepared from 3-bromodihydrofuran-2(3*H*)-one using the general procedures as colorless oil (0.38 g, 65% yield). ^{1}H NMR (400 MHz, $CDCl_3$) δ 4.39 (ddd, J = 9.1, 7.6, 5.9 Hz, 1H), 4.26 (ddd, J = 9.1, 7.3, 6.5 Hz, 1H), 3.67 (ddd, J = 8.4, 6.9, 5.1 Hz, 1H), 2.73 - 2.53 (m, 1H), 2.28 (d, J = 5.1 Hz, 1H), 2.12 (ddt, J = 13.4, 7.5, 6.7 Hz, 1H). ^{13}C NMR (101 MHz, $CDCl_3$) δ 176.6, 66.5, 34.7, 31.9. HRMS (ESI) calcd for $C_4H_7O_2S$ $[M + H]^+$

m/z = 119.0161, found: 119.0161. The NMR spectra matches that of previously reported literature.[3]

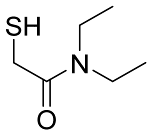

***N*, *N*-diethyl-2-mercaptoacetamide (c)** was prepared from 2-chloro-*N*, *N*-diethylacetamide according to the general procedures as colorless oil (0.55 g, 75% yield). ^{1}H NMR (400 MHz, $CDCl_3$) δ 3.43 - 3.15 (m, 6H), 2.12 (t, J = 7.5 Hz, 1H), 1.17 (t, J = 7.2 Hz, 3H), 1.08 (t, J = 7.1 Hz, 3H). ^{13}C NMR (101 MHz, $CDCl_3$) δ 169.1, 42.7, 40.8, 26.3, 14.5, 12.9. HRMS (ESI) calcd for $C_6H_{14}NOS$ $[M + H]^+$ m/z = 148.0791, found: 148.0795. The NMR spectra matches that of previously reported literature.[2]

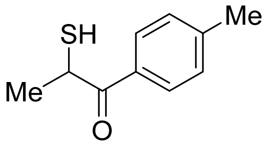

2-mercapto-1-(p-tolyl) propan-1-one (d) was prepared from 2-bromo-1-(p-tolyl) propan-1-one using the general procedures as yellow oil (0.54 g, 60% yield). ^{1}H NMR (400 MHz, $CDCl_3$) δ 7.84 (d, J = 8.2 Hz, 2H), 7.23 (d, J = 8.3 Hz, 2H), 4.34 (dq, J = 9.5, 6.8 Hz, 1H), 2.37 (s, 3H), 2.00 (d, J = 9.5 Hz, 1H), 1.57 (d, J = 6.8 Hz, 3H). ^{13}C NMR (101 MHz, $CDCl_3$) δ 197.4, 144.2, 132.2, 129.5, 128.8, 36.6, 21.7, 21.0. HRMS (ESI) calcd for $C_{10}H_{13}OS$ $[M + H]^+$ m/z = 181.0682, found: 181.0682.

General protocol B for the synthesis of thiols:

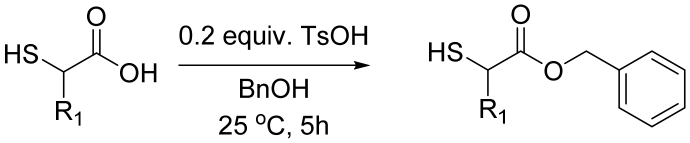

To a round bottomed flask with 5 ml BnOH and 2-mercaptoacetic acid (20 mmol) was added *p*-Toluenesulfonic acid (0.2 equiv, 0.34g, 2 mmol). The reaction mixture was stirred for 5 hours. DCM (150 mL) was added. The mixture was washed with brine (20 mL), dried over Na_2SO_4, concentrated and purified by flash chromatography to give the desired thiol.

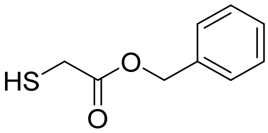

benzyl 2-mercaptoacetate (e) was prepared according to the general protocol B as transparent oil (2.9 g, 80% yield). ^{1}H NMR (400 MHz, $CDCl_3$) δ 7.46 - 7.27 (m, 5H), 5.16 (s, 2H), 3.28 (d, J = 8.3 Hz, 2H), 2.01 (t, J = 8.3 Hz, 1H). ^{13}C NMR (101 MHz, $CDCl_3$) δ 170.7, 135.4, 128.7, 128.5, 128.4, 67.4, 26.6. HRMS (ESI) calcd for $C_9H_{11}O_2S$ $[M + H]^+$ m/z =183.0474, found:183.0477. The NMR data matches that of reported literature.[4]

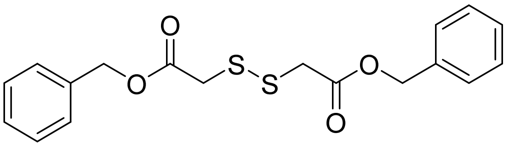

dibenzyl 2,2'-disulfanediyldiacetate (f) was prepared according to the general protocol B as transparent oil (5.9g, 82% yield). ^{1}H NMR (400 MHz, $CDCl_3$) *δ* 7.45 - 7.26 (m, 10H), 5.16 (s, 4H), 3.56 (s, 4H). ^{13}C NMR (101 MHz, $CDCl_3$) *δ* 169.2, 135.4, 128.7, 128.7, 128.6, 128.5, 128.43, 67.4, 41.5. HRMS (ESI) calcd for $C_{18}H_{19}O_4S_2$ $[M + H]^+$ m/z = 363.0719, found: 363.0721.

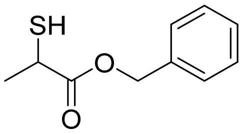

benzyl 2-mercaptopropanoate (g) was prepared according to the general protocol B as transparent oil (3.3 g, 85% yield). HRMS (ESI) calcd for $C_{10}H_{13}O_2S$ $[M + H]^+$ m/z = 197.0631, found: 197.0632. The NMR data matches that of reported literature.[5]

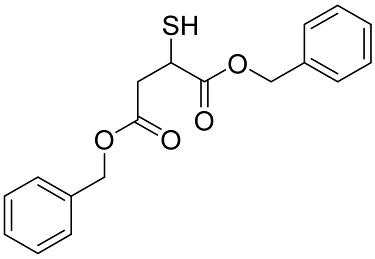

dibenzyl 2-mercaptosuccinate (h) was prepared according to the general protocol B as transparent oil (5.6 g, 85% yield). ^{1}H NMR (400 MHz, $CDCl_3$) *δ* 7.43 - 7.27 (m, 10H), 5.24 - 5.03 (m, 4H), 3.83 (td, *J* = 9.2, 5.9 Hz, 1H), 3.08 (dd, *J* = 17.0, 9.0 Hz, 1H), 2.83 (dd, *J* = 17.0, 5.9 Hz, 1H), 2.22 (d, *J* = 9.5 Hz, 1H). ^{13}C NMR (101 MHz, $CDCl_3$) *δ* 172.0, 170.0, 135.5, 135.4, 128.6, 128.4, 128.3, 128.2, 67.5, 66.9, 39.9, 36.3. HRMS (ESI) calcd for $C_{18}H_{19}O_4S$ $[M + H]^+$ m/z = 331.0999, found: 331.0997.

3. Optimization of the reaction condition

Supplementary Table 1 | Optimization of reaction conditions [a].

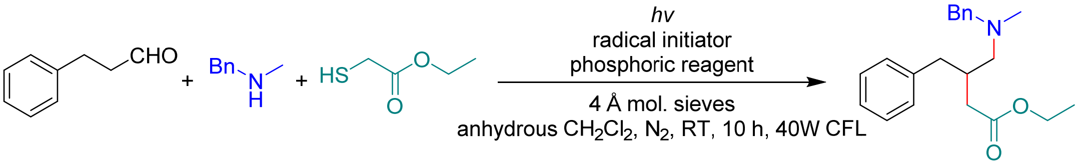

1 (1.5 equiv) **2** **3** (1.5 equiv) **4**

entry	phosphoric reagent	radical initiator	solvent	Yield (%) [b]
1	1.5 equiv. PPh_3	2.0 equiv. DTBP	DCM	91
2	2.0 equiv. PPh_3	2.0 equiv. DTBP	DCM	91
3	1.2 equiv. PPh_3	2.0 equiv. DTBP	DCM	85
4	1.5 equiv. PPh_3	1.0 equiv. DTBP	DCM	70
5	1.5 equiv. PPh_3	1.5 equiv. DTBP	DCM	82
6	1.5 equiv. PPh_3	2.5 equiv. DTBP	DCM	91
7	1.5 equiv. PPh_3	2.0 equiv. DTBP	EA	72
8	1.5 equiv. PPh_3	2.0 equiv. DTBP	CH_3CN	48
9	1.5 equiv. PPh_3	2.0 equiv. DTBP	DMF	79
10	1.5 equiv. PPh_3	2.0 equiv. DTBP	Toluene	82
11	1.5 equiv. TEP	2.0 equiv. DTBP	DCM	88
12	1.5 equiv. Ph_2POEt	2.0 equiv. DTBP	DCM	86
14	1.5 equiv. PPh_3	2.0 equiv. H_2O_2	DCM	N.D.
15	1.5 equiv. PPh_3	2.0 equiv. TBHP	DCM	N.D.

16	1.5 equiv. PPh_3	2.0 equiv. DCP	DCM	72
17	1.5 equiv. PPh_3	0.05 equiv. $Ru(bpy)_3Cl_2$	DCM	<5
18	1.5 equiv. PPh_3	0.05 equiv. $(Ir[dF(CF_3)ppy]_2(dtbpy))PF_6$	DCM	29%
19	1.5 equiv. PPh_3	0.1 equiv. Eosin Y	DCM	<5
20	1.5 equiv. PPh_3	—	DCM	N.D.
21	—	2.0 equiv. DTBP	DCM	N.D.
22[c]	1.5 equiv. PPh_3	2.0 equiv. DTBP	DCM	N.D.

[a]Reactions conditions: **1** (1.5 mmol), **2** (1.0 mmol), **3** (1.5 mmol), phosphoric reagent, radical initiator, 6 ml solvent, room temperature, 40 W household CFL bulb irradiation on two sides, 10 h. [b]Yields of isolated product. N.D. = not detected DTBP = di-tert-butyl peroxide, TBHP = t-butylhydroperoxide, DCP = Dicumyl peroxide. TEP = triethyl phosphite. [c]Without light.

4. General procedure for the three-component radical homo Mannich reaction

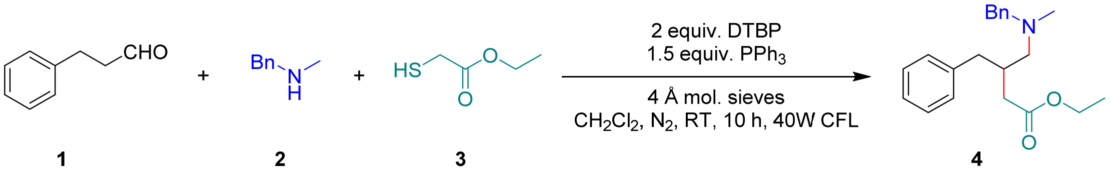

1 **2** **3** **4**

To a 10 ml oven-dried round bottomed flask were added 4 Å molecular sieves (1g) and triphenylphosphine (393 mg, 1.5 mmol). The flask was degassed three times and protected with N_2 before anhydrous DCM (6 ml) was added. Amine (1 mmol), aldehyde/ketone (1.5 mmol), thiol (1.5 mmol) and DTBP (292 mg, 2 mmol, 0.37ml) were added into the reaction mixture in order by micro-syringe. The reaction was stirred and irradiated using two 40 W household CFL bulbs (6 cm away, to keep the reaction at room temperature) at room temperature for 10 hours. When the reaction was complete, EtOAc was added (20 mL). The mixture was dried with sodium sulfate, filtered and concentrated. The residue was purified by column chromatography on silica to give the product.

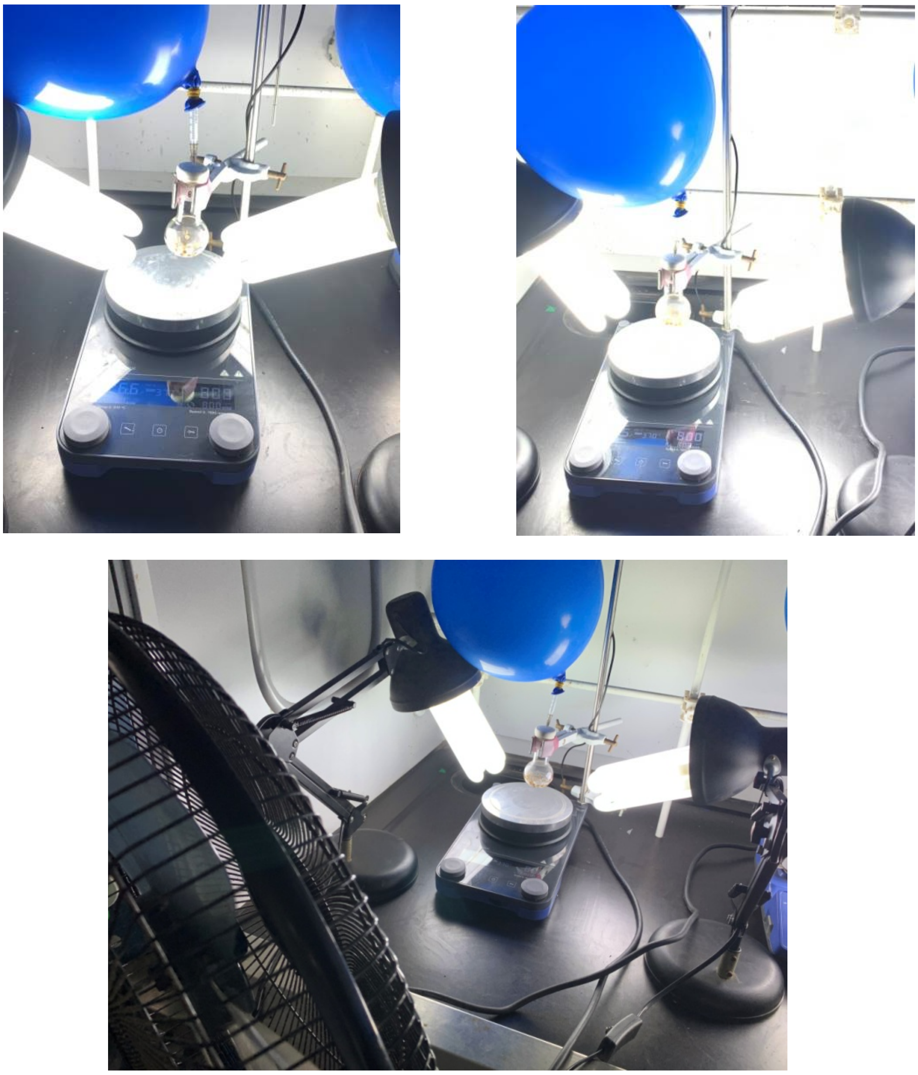

Supplementary Fig. 2 Experimental set-up pictures using visible-light

5. Characterization data for all key compounds

ethyl 3-benzyl-4-(benzyl(methyl)amino) butanoate (4)

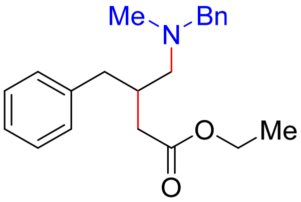

Prepared following the general procedure employing N-methyl-1-phenylmethanamine (121mg, 1 mmol), 3-phenylpropanal (201 mg, 1.5 mmol), ethyl 2-mercaptoacetate (180 mg, 1.5 mmol), triphenylphosphine (393 mg, 1.5 mmol), 4Å MS (1 g), di-tert-butyl peroxide (292 mg, 2 mmol, 0.37ml) in anhydrous dichloromethane (6 mL). The desired product was obtained as colorless oil (295 mg, 91% yield) after purification by flash column chromatography on silica gel (0.5% Et_3N in dichloromethane).

^{1}H NMR (400 MHz, $CDCl_3$) δ 7.42 - 7.04 (m, 10H), 4.06 (q, J = 7.1 Hz, 2H), 3.46 (s, 2H), 2.76 (dd, J = 13.2, 5.6 Hz, 1H), 2.59 - 2.33 (m, 3H), 2.32 - 2.18 (m, 3H), 2.15 (s, 3H), 1.22 (t, J = 7.1 Hz, 3H). ^{13}C NMR (101 MHz, $CDCl_3$) δ 173.4, 140.1, 139.5, 129.5, 129.1, 128.4, 128.3, 127.0, 126.1, 62.9, 61.6, 60.2, 42.6, 39.0, 37.4, 35.6, 14.4. HRMS (ESI) calcd for $C_{21}H_{28}NO_2$ $[M + H]^+$ m/z = 326.2115, found: 326.2117. IR ν_{max}/cm^{-1} (film): 3026, 2928, 2789, 1731, 1494, 1453, 1372, 1250, 1153, 1077, 1025, 969, 741, 699.

ethyl 3-benzyl-4-((4-methoxybenzyl)(methyl)amino)butanoate (5)

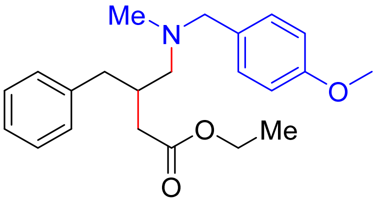

Prepared following the general procedure employing 1-(4-methoxyphenyl)-N-methylmethanamine (151 mg, 1 mmol), 3-phenylpropanal (201 mg, 1.5 mmol), ethyl 2-mercaptoacetate (180 mg, 1.5 mmol), triphenylphosphine (393 mg, 1.5 mmol), 4Å MS (1 g), di-tert-butyl peroxide (292 mg, 2 mmol) in anhydrous dichloromethane (6 mL). The desired product was obtained as yellow oil (301 mg, 85% yield) after purification by flash column chromatography on silica gel (0.5% Et_3N in ethyl acetate).

^{1}H NMR (400 MHz, $CDCl_3$) δ 7.32 - 7.09 (m, 6H), 6.95 - 6.72 (m, 3H), 4.03 (q, J = 7.1 Hz, 2H), 3.75 (s, 3H), 3.47 - 3.36 (m, 2H), 2.75 (dd, J = 13.0, 5.4 Hz, 1H), 2.56 - 2.31 (m, 3H), 2.28 - 2.16 (m, 3H), 2.15 (s, 3H), 1.19 (t, J = 7.1 Hz, 3H). ^{13}C NMR (101 MHz, $CDCl_3$) δ 173.1, 159.6, 141.1, 139.9, 129.3, 129.0, 128.2, 126.0, 121.2, 114.3, 112.4, 62.8, 61.4, 60.0, 55.0, 42.5, 38.8, 37.2, 35.4, 14.2. HRMS (ESI) calcd for $C_{22}H_{30}NO_3$ $[M + H]^+$ m/z = 356.2220, found: 356.2226. IR ν_{max}/cm^{-1} (film): 3026, 2939, 2836, 2788, 1731, 1600, 1585, 1488, 1453, 1371, 1263, 1151, 1031, 782, 745, 699.

ethyl 3-benzyl-4-((4-fluorobenzyl)(methyl)amino)butanoate (6)

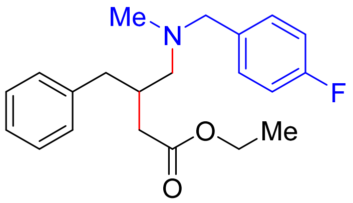

Prepared following the general procedure employing 1-(4-fluorophenyl)-N-methylmethanamine (139 mg, 1 mmol), 3-phenylpropanal (201 mg, 1.5 mmol), ethyl 2-mercaptoacetate (180 mg, 1.5 mmol), triphenylphosphine (393 mg, 1.5 mmol), 4Å MS (1 g), di-tert-butyl peroxide (292 mg, 2 mmol) in anhydrous dichloromethane (6 mL). The desired product was obtained as yellow oil (274 mg, 80% yield) after purification by flash column chromatography on silica gel (0.5% Et_3N in ethyl acetate).

^{1}H NMR (400 MHz, $CDCl_3$) δ 7.37 - 7.14 (m, 7H), 7.08 - 6.90 (m, 2H), 4.09 (q, J = 7.1 Hz, 2H), 3.44 (s, 2H), 2.78 (dd, J = 13.2, 5.7 Hz, 1H), 2.54 (m, 2H), 2.39 (dd, J = 15.5, 6.4 Hz, 1H), 2.33 - 2.19 (m, 3H), 2.16 (s, 3H), 1.25 (t, J = 7.1 Hz, 3H). ^{13}C NMR (101 MHz, $CDCl_3$) δ 173.14, 161.88 (d, J = 244.5 Hz), 139.91, 135.01 (d, J = 3.1 Hz), 130.39 (d, J = 7.8 Hz), 129.31, 128.25, 126.02, 114.88 (d, J = 21.1 Hz), 61.94, 61.37, 60.06, 42.29, 38.88, 37.29, 35.44, 14.22. ^{19}F NMR (376 MHz, $CDCl_3$) δ -116.1 (s). HRMS (ESI) calcd for $C_{21}H_{26}FNO_2$ $[M + H]^+$ m/z = 344.2020, found: 344.2024. IR ν_{max}/cm^{-1} (film): 2982, 2937, 2797, 1732, 1602, 1508, 1453, 1371, 1221, 1154, 1092, 1030, 820, 745, 700.

ethyl 3-benzyl-4-((4-bromobenzyl)(methyl)amino)butanoate (7)

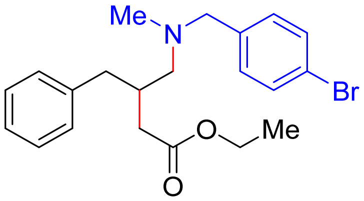

Prepared following general procedure employing 1-(4-bromophenyl)-N-methylmethanamine (199 mg, 1 mmol), 3-phenylpropanal (201 mg, 1.5 mmol), ethyl 2-mercaptoacetate (180 mg, 1.5 mmol), triphenylphosphine (393 mg, 1.5 mmol), 4Å MS (1 g), di-tert-butyl peroxide (292 mg, 2 mmol) in anhydrous dichloromethane (6 mL). The desired product was obtained as a colorless oil (282 mg, 70% yield) after purification by flash column chromatography on silica gel (0.5% Et_3N in dichloromethane).

^{1}H NMR (400 MHz, $CDCl_3$) δ 7.45 - 7.33 (m, 2H), 7.31 - 7.08 (m, 7H), 4.04 (q, J = 7.1 Hz, 2H), 3.39 (s, 2H), 2.72 (dd, J = 13.4, 5.9 Hz, 1H), 2.56 - 2.38 (m, 2H), 2.33 (dd, J = 15.5, 6.5 Hz, 1H), 2.28 - 2.14 (m, 3H), 2.12 (s, 3H), 1.21 (t, J = 7.1 Hz, 3H). ^{13}C NMR (101 MHz, $CDCl_3$) δ 173.3, 140.0, 138.6, 131.4, 130.8, 129.4, 128.4, 126.2, 120.8, 62.2, 61.6, 60.3, 42.5, 39.0, 37.4, 35.6, 14.4. HRMS (ESI) calcd for $C_{21}H_{27}BrNO_2$ $[M + H]^+$ m/z = 404.1220, found: 404.1222. IR ν_{max}/cm^{-1} (film): 2930, 2796, 1734, 1486, 1453, 1402, 1371, 1259, 1151, 1097, 1069, 1030, 1011, 798, 745, 700.

ethyl 3-benzyl-4-(methyl(naphthalen-1-ylmethyl)amino)butanoate (8)

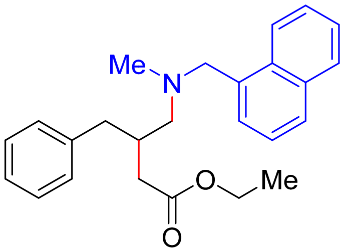

Prepared following the general procedure employing N-methyl-1-(naphthalen-1-yl) methanamine (171mg, 1 mmol), 3-phenylpropanal (201 mg, 1.5 mmol), ethyl 2-mercaptoacetate (180 mg, 1.5 mmol), triphenylphosphine (393 mg, 1.5 mmol), 4Å MS (1 g), di-tert-butyl peroxide (292 mg, 2 mmol) in anhydrous dichloromethane (6 mL). The desired product was obtained as colorless oil (244 mg, 65% yield) after purification by flash column chromatography on silica gel (0.5% Et_3N in petroleum ether: ethyl acetate = 6:1).

^{1}H NMR (400 MHz, $CDCl_3$) δ 8.31 - 8.23 (m, 1H), 7.83 (dd, J = 6.5, 2.9 Hz, 1H), 7.76 (dd, J = 6.7, 2.7 Hz, 1H), 7.53 - 7.33 (m, 4H), 7.24 - 7.01 (m, 5H), 4.00 (q, J = 7.1 Hz, 2H), 3.86 (q, J = 13.1 Hz, 2H), 2.73 (dt, J = 9.0, 4.6 Hz, 1H), 2.45 (dt, J = 13.1, 7.0 Hz, 2H), 2.40 - 2.26 (m, 3H), 2.20 (s, 3H), 2.12 (dd, J = 15.7, 6.3 Hz, 1H), 1.18 (t, J = 7.1 Hz, 3H). ^{13}C NMR (101 MHz, $CDCl_3$) δ 173.4, 140.2, 135.1, 134.0, 132.7, 129.5, 128.5, 128.3, 128.0, 127.6, 126.1, 125.8, 125.7, 125.27, 125.0, 61.9, 61.7, 60.2, 42.7, 39.0, 37.2, 35.6, 14.4. HRMS (ESI) calcd for $C_{25}H_{30}NO_2$ $[M + H]^+$ m/z = 376.2271, found: 376.2276. IR ν_{max}/cm^{-1} (film): 2968, 2793, 1734, 1438, 1260, 1095, 1028, 796, 742.

ethyl 4-(allyl(methyl)amino)-3-benzylbutanoate (9)

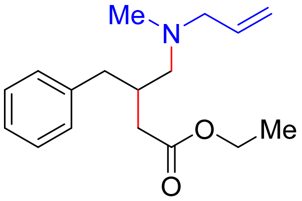

Prepared following the general procedure employing N-methylprop-2-en-1-amine (71 mg, 1 mmol), 3-phenylpropanal (201 mg, 1.5 mmol), ethyl 2-mercaptoacetate (180 mg, 1.5 mmol), triphenylphosphine (393 mg, 1.5 mmol), 4Å MS (1 g), di-tert-butyl peroxide (292 mg, 2 mmol) in anhydrous dichloromethane (6 mL). The desired product was obtained as a colorless oil (225 mg, 82% yield) after purification by flash column chromatography on silica gel (0.5% Et_3N in dichloromethane).

^{1}H NMR (400 MHz, $CDCl_3$) δ 7.34 - 7.05 (m, 5H), 5.80 (ddt, J = 16.7, 10.2, 6.5 Hz, 1H), 5.22 - 4.98 (m, 2H), 4.04 (q, J = 7.1 Hz, 2H), 3.02 - 2.86 (m, 2H), 2.71 (dd, J = 13.6, 6.0 Hz, 1H), 2.52 (dd, J = 13.6, 7.4 Hz, 1H), 2.39 (dt, J = 13.5, 6.8 Hz, 1H), 2.33 - 2.17 (m, 4H), 2.15 (s, 3H), 1.21 (t, J = 7.1 Hz, 3H). ^{13}C NMR (101 MHz, $CDCl_3$) δ 173.4, 140.1, 136.1, 129.5, 128.3, 126.1, 117.2, 61.4, 61.2, 60.2, 42.5, 39.1, 37.6, 35.6, 31.6, 29.8, 14.4. HRMS (ESI) calcd for $C_{17}H_{26}NO_2$ $[M + H]^+$ m/z = 276.1958, found: 276.1960. IR ν_{max}/cm^{-1} (film): 2977, 2928, 1733, 1453, 1371, 1257, 1149, 1031, 918, 744, 700.

ethyl 3-benzyl-4-((2-cyanoethyl)(methyl)amino)butanoate **(10)**

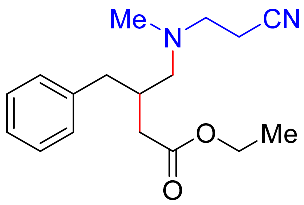

Prepared following the general procedure employing 3-(methylamino)propanenitrile (84 mg, 1 mmol), 3-phenylpropanal (201 mg, 1.5 mmol), ethyl 2-mercaptoacetate (180 mg, 1.5 mmol), triphenylphosphine (393 mg, 1.5 mmol), 4Å MS (1 g), di-tert-butyl peroxide (292 mg, 2 mmol) in anhydrous dichloromethane (6 mL). The desired product was obtained as a yellow oil (184 mg, 64% yield) after purification by flash column chromatography on silica gel (0.5% Et_3N in ethyl acetate).

^{1}H NMR (400 MHz, $CDCl_3$) δ 7.29 - 7.21 (m, 2H), 7.20 - 7.09 (m, 3H), 4.04 (q, J = 7.1 Hz, 2H), 2.71 (dd, J = 13.6, 5.7 Hz, 1H), 2.65 (dd, J = 10.5, 3.9 Hz, 2H), 2.53 (dd, J = 13.6, 7.0 Hz, 1H), 2.39 (td, J = 6.9, 1.5 Hz, 2H), 2.37 - 2.22 (m, 5H), 2.21 (s, 3H), 1.20 (t, J = 7.1 Hz, 3H). ^{13}C NMR (101 MHz, $CDCl_3$) δ 173.0, 139.6, 129.3, 128.3, 126.1, 119.0, 61.0, 60.1, 53.3, 41.9, 38.6, 36.9, 35.5, 15.9, 14.2. HRMS (ESI) calcd for $C_{17}H_{25}N_2O_2$ [M + H]$^+$ m/z = 289.1911, found: 289.1912. IR ν_{max}/cm^{-1} (film): 3026, 2950, 2852, 2806, 1728, 1495, 1453, 1373, 1259, 1209, 1152, 1030, 799, 747, 701.

ethyl 3-benzyl-4-(cyclohexyl(methyl)amino)butanoate (11)

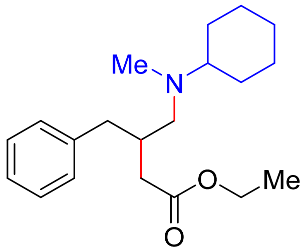

Prepared following the general procedure employing N-methylcyclohexanamine (113 mg, 1 mmol), 3-phenylpropanal (201 mg, 1.5 mmol), ethyl 2-mercaptoacetate (180 mg, 1.5 mmol), triphenylphosphine (393 mg, 1.5 mmol), 4Å MS (1 g), di-tert-butyl peroxide (292 mg, 2 mmol) in anhydrous dichloromethane (6 mL). The desired product was obtained as colorless oil (301 mg, 95% yield) after purification by flash column chromatography on silica gel (0.5% Et_3N in ethyl acetate).

^{1}H NMR (400 MHz, $CDCl_3$) δ 7.21 - 7.09 (m, 5H), 3.97 (q, J = 7.1 Hz, 2H), 2.66 (dd, J = 13.6, 5.4 Hz, 1H), 2.42 (dd, J = 13.6, 7.1 Hz, 1H), 2.34 - 2.15 (m, 5H), 2.15 - 2.04 (m, 4H), 1.75 - 1.45 (m, 5H), 1.21 - 0.93 (m, 8H). ^{13}C NMR (101 MHz, $CDCl_3$) δ 173.3, 140.3, 129.2, 128.2, 125.8, 63.5, 59.9, 57.9, 39.0, 37.8, 37.4, 35.9, 28.6, 28.4, 26.4, 26.1, 14.2. HRMS (ESI) calcd for $C_{20}H_{32}NO_2$ [M + H]$^+$ m/z = 318.2428, found: 318.2429. IR ν_{max}/cm^{-1} (film): 2928, 2854, 1734, 1495, 1451, 1370, 1259, 1144, 1098, 1037, 946, 796, 743, 700.

ethyl 3-benzyl-4-(diethylamino)butanoate (12)

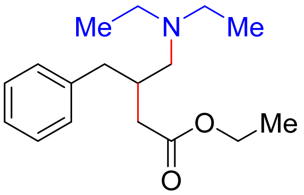

Prepared following the general procedure employing diethylamine (73 mg, 1 mmol), 3-phenylpropanal (201 mg, 1.5 mmol), ethyl 2-mercaptoacetate (180 mg, 1.5 mmol), triphenylphosphine (393 mg, 1.5 mmol), 4Å MS (1 g), di-tert-butyl peroxide (292 mg, 2 mmol) in anhydrous dichloromethane (6 mL). The desired product was obtained as colorless oil (244 mg, 88% yield) after purification by flash column chromatography on silica gel (0.5% Et_3N in dichloromethane).

^{1}H NMR (400 MHz, $CDCl_3$) δ 7.25 (dd, J = 10.5, 4.4 Hz, 2H), 7.17 - 7.14 (m, 3H), 4.03 (q, J = 7.1 Hz, 2H), 2.73 (dd, J = 13.6, 5.6 Hz, 1H), 2.57 - 2.38 (m, 5H), 2.37 - 2.13 (m, 5H), 1.20 (t, J = 7.1 Hz, 3H), 0.94 (t, J = 7.1 Hz, 6H). ^{13}C NMR (101 MHz, $CDCl_3$) δ 173.5, 140.4, 129.4, 128.3, 126.0, 60.2, 57.4, 47.4, 39.2, 37.6, 36.0, 14.4, 11.7. HRMS (ESI) calcd for $C_{17}H_{28}NO_2$ $[M + H]^+$ m/z = 278.2115, found: 278.2115. IR ν_{max}/cm^{-1} (film): 2968, 2930, 1733, 1453, 1371, 1256, 1202, 1148, 1031, 744, 700.

ethyl 3-benzyl-4-(ethyl(pyridin-4-ylmethyl)amino)butanoate (13)

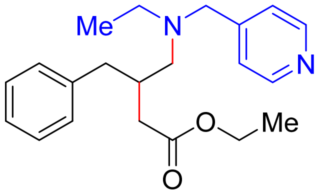

Prepared following the general procedure employing N-(pyridin-4-ylmethyl) ethanamine (136 mg, 1 mmol), 3-phenylpropanal (201 mg, 1.5 mmol), ethyl 2-mercaptoacetate (180 mg, 1.5 mmol), triphenylphosphine (393 mg, 1.5 mmol), 4Å MS (1 g), di-tert-butyl peroxide (292 mg, 2 mmol) in anhydrous dichloromethane (6 mL). The desired product was obtained as colorless oil (272 mg, 80% yield) after purification by flash column chromatography on silica gel (0.5% Et_3N in ethyl acetate).

^{1}H NMR (400 MHz, $CDCl_3$) δ 8.49 (dd, J = 4.4, 1.6 Hz, 2H), 7.29 - 7.14 (m, 5H), 7.11 (dd, J = 5.1, 3.1 Hz, 2H), 4.04 (q, J = 7.1 Hz, 2H), 3.51 (dd, J = 37.2, 14.8 Hz, 2H), 2.75 (dd, J = 13.5, 5.4 Hz, 1H), 2.54 - 2.24 (m, 7H), 2.17 (dd, J = 17.7, 8.1 Hz, 1H), 1.20 (t, J = 7.1 Hz, 3H), 0.97 (t, J = 7.1 Hz, 3H). ^{13}C NMR (101 MHz, $CDCl_3$) δ 173.3, 149.6, 140.1, 129.3, 128.4, 126.2, 123.9, 60.3, 57.9, 57.7, 48.1, 39.1, 37.3, 35.8, 14.4, 11.7. HRMS (ESI) calcd for $C_{21}H_{29}N_2O_2$ $[M + H]^+$ m/z = 341.2224, found: 341.2225. IR ν_{max}/cm^{-1} (film): 2966, 2939, 1734, 1413, 1370, 1259, 1201, 1151, 1029, 799, 743, 701.

ethyl 3-benzyl-4-(benzyl(2-ethoxy-2-oxoethyl)amino)butanoate (14)

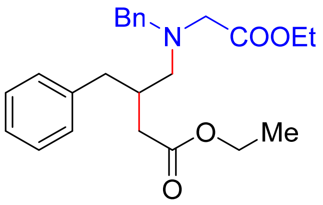

Prepared following the general procedure employing ethyl benzylglycinate (193 mg, 1 mmol), 3-phenylpropanal (201 mg, 1.5 mmol), ethyl 2-mercaptoacetate (180 mg, 1.5 mmol), triethyl phosphite (249 mg, 1.5 mmol), 4Å MS (1 g), di-tert-butyl peroxide (292 mg, 2 mmol) in anhydrous dichloromethane (6 mL). The desired product was obtained as pink oil (222 mg, 56% yield) after purification by flash column chromatography on silica gel (0.5% Et_3N in petroleum ether: ethyl acetate = 8:1).

^{1}H NMR (400 MHz, $CDCl_3$) δ 7.36 - 7.20 (m, 7H), 7.20 - 7.03 (m, 3H), 4.10 (q, J = 7.1 Hz, 2H), 4.04 (q, J = 7.1 Hz, 2H), 3.75 (dd, J = 29.9, 13.5 Hz, 2H), 3.26 (s, 2H), 2.76 (dd, J = 13.5, 5.5 Hz, 1H), 2.61 (dd, J = 12.7, 5.8 Hz, 1H), 2.55 - 2.33 (m, 4H), 2.18 (dd, J = 17.6, 8.4 Hz, 1H), 1.22 (td, J = 7.1, 5.1 Hz, 6H). ^{13}C NMR (101 MHz, $CDCl_3$) δ 173.2, 171.3, 134.0, 138.9, 129.2, 129.0, 128.2, 127.0, 126.0, 60.1, 60.1, 58.4, 58.0, 54.4, 38.7, 36.9, 35.8, 14.2, 14.2. HRMS (ESI) calcd for $C_{24}H_{32}NO_4$ $[M + H]^+$ m/z = 398.2326, found: 398.2330. IR ν_{max}/cm^{-1} (film): 2980, 2927, 1734, 1451, 1370, 1256, 1184, 1150, 1028, 742, 699.

ethyl 3-benzyl-4-(benzyl(3-ethoxy-3-oxopropyl)amino)butanoate (15)

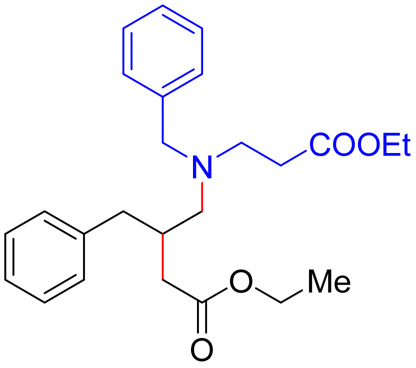

Prepared following the general procedure employing ethyl 3-(benzylamino)propanoate (207mg, 1 mmol), 3-phenylpropanal (201 mg, 1.5 mmol), ethyl 2-mercaptoacetate (180 mg, 1.5 mmol), triphenylphosphine (393 mg, 1.5 mmol), 4Å MS (1 g), di-tert-butyl peroxide (292 mg, 2 mmol) in anhydrous dichloromethane (6 mL). The desired product was obtained as colorless oil (378 mg, 92% yield) after purification by flash column chromatography on silica gel (0.5% Et_3N in dichloromethane).

^{1}H NMR (400 MHz, $CDCl_3$) δ 7.25 - 7.08 (m, 8H), 7.04 (d, J = 7.1 Hz, 2H), 4.09 - 3.90 (m, 4H), 3.53 - 3.41 (m, 2H), 2.79 - 2.59 (m, 3H), 2.41 - 2.17 (m, 7H), 2.07 (dd, J = 15.4, 5.7 Hz, 1H), 1.15 (td, J = 7.1, 4.3 Hz, 6H). ^{13}C NMR (101 MHz, $CDCl_3$) δ 173.3, 172.8, 140.3, 139.3, 129.4, 129.1, 128.4, 128.3, 127.1, 126.1, 60.5, 60.2, 58.9, 58.0, 50.1, 38.9, 37.1, 35.7, 32.6, 14.4, 14.3. HRMS (ESI) calcd for $C_{25}H_{34}NO_4$ $[M + H]^+$ m/z = 412.2482, found: 412.2482. IR ν_{max}/cm^{-1} (film): 3028, 2976, 2908, 1734, 1451, 1370, 1258, 1179, 1029, 800, 741, 700.

ethyl 3-benzyl-4-(dibenzylamino)butanoate (16)

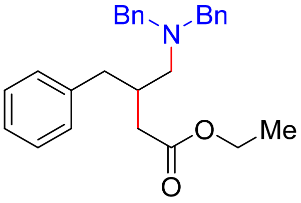

Prepared following the general procedure employing dibenzylamine (197 mg, 1 mmol), 3-phenylpropanal (201 mg, 1.5 mmol), ethyl 2-mercaptoacetate (180 mg, 1.5 mmol), triphenylphosphine (393 mg, 1.5 mmol), 4Å MS (1 g), di-tert-butyl peroxide (292 mg, 2 mmol) in anhydrous dichloromethane (6 mL). The desired product was obtained as colorless oil (320 mg, 80% yield) after purification by flash column chromatography on silica gel (0.5% Et_3N in petroleum ether: ethyl acetate = 15:1).

^{1}H NMR (400 MHz, $CDCl_3$) δ 7.36 - 7.13 (m, 13H), 7.06 (d, J = 6.9 Hz, 2H), 4.02 (q, J = 7.1 Hz, 2H), 3.52 (q, J = 13.6 Hz, 4H), 2.76 (dd, J = 13.4, 5.9 Hz, 1H), 2.51 - 2.41 (m, 1H), 2.41 - 2.20 (m, 4H), 2.06 (dd, J = 15.8, 6.6 Hz, 1H), 1.19 (t, J = 7.1 Hz, 3H). ^{13}C NMR (101 MHz, $CDCl_3$) δ 173.4, 140.4, 139.5, 129.4, 129.2, 128.4, 128.3, 127.0, 126.1, 60.3, 58.9, 57.7, 39.1, 37.3, 35.7, 29.9, 14.4. HRMS (ESI) calcd for $C_{27}H_{32}NO_2$ [M + H]$^+$ m/z = 402.2428, found: 402.2429. IR ν_{max}/cm^{-1} (film): 2960, 1734, 1437, 1420, 1261, 1027, 873, 798, 742, 700.

ethyl 3-benzyl-4-(bis(4-methoxybenzyl)amino)butanoate (17)

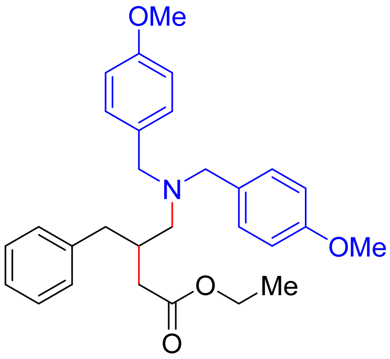

Prepared following the general procedure employing bis(4-methoxybenzyl)amine (257mg, 1 mmol), 3-phenylpropanal (201 mg, 1.5 mmol), ethyl 2-mercaptoacetate (180 mg, 1.5 mmol), triphenylphosphine (393 mg, 1.5 mmol), 4Å MS (1 g), di-tert-butyl peroxide (292 mg, 2 mmol) in anhydrous dichloromethane (6 mL). The desired product was obtained as colorless oil (277 mg, 60% yield) after purification by flash column chromatography on silica gel (0.5% Et_3N in dichloromethane).

^{1}H NMR (400 MHz, $CDCl_3$) δ 7.18 - 7.05 (m, 7H), 7.01 (d, J = 7.0 Hz, 2H), 6.76 (d, J = 8.6 Hz, 4H), 3.96 (q, J = 7.1 Hz, 2H), 3.73 (s, 6H), 3.38 (q, J = 13.4 Hz, 4H), 2.67 (dd, J = 13.3, 5.9 Hz, 1H), 2.39 (dt, J = 13.3, 6.7 Hz, 1H), 2.34 - 2.09 (m, 4H), 1.99 (dd, J = 15.6, 6.6 Hz, 1H), 1.14 (t, J = 7.1 Hz, 3H). ^{13}C NMR (101 MHz, $CDCl_3$) δ 173.5, 158.7, 140.5, 131.6, 130.3, 129.4, 128.4, 126.0, 113.7, 60.3, 58.0, 57.3, 55.4, 39.1, 37.4, 35.7, 14.4. HRMS (ESI) calcd for $C_{29}H_{36}NO_4$ [M + H]$^+$ m/z = 462.2639, found: 462.2643. IR ν_{max}/cm^{-1} (film): 2958, 1734, 1511, 1256, 1096, 1033, 801, 743, 700.

ethyl 4-(azetidin-1-yl)-3-benzylbutanoate (18)

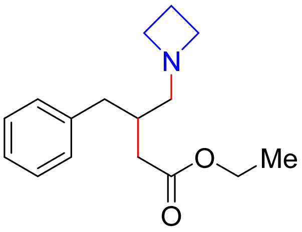

Prepared following the general procedure employing azetidine (57 mg, 1 mmol), 3-phenylpropanal (201 mg, 1.5 mmol), ethyl 2-mercaptoacetate (180 mg, 1.5 mmol), triphenylphosphine (393 mg, 1.5 mmol), 4Å MS (1 g), di-tert-butyl peroxide (292 mg, 2 mmol) in anhydrous dichloromethane (6 mL). The desired product was obtained as colorless oil (211 mg, 81% yield) after purification by flash column chromatography on silica gel (0.5% Et_3N in dichloromethane: methanol = 20:1).

^{1}H NMR (400 MHz, $CDCl_3$) δ 7.29 - 7.21 (m, 2H), 7.21 - 7.05 (m, 3H), 4.15 - 3.94 (m, 2H), 3.20 (tt, J = 13.6, 6.9 Hz, 4H), 2.71 (dd, J = 13.6, 6.1 Hz, 1H), 2.51 (dd, J = 13.6, 7.3 Hz, 1H), 2.42 - 2.10 (m, 5H), 2.04 (p, J = 7.1 Hz, 2H), 1.21 (t, J = 7.1 Hz, 3H). ^{13}C NMR (101 MHz, $CDCl_3$) δ 173.2, 139.9, 129.4, 128.4, 126.2, 63.2, 60.3, 55.7, 38.8, 37.1, 35.8, 17.6, 14.4. HRMS (ESI) calcd for $C_{16}H_{24}NO_2$ [M + H]$^+$ m/z = 262.1802, found: 262.1805. IR ν_{max}/cm^{-1} (film): 2956, 2817, 1732, 1691, 1495, 1452, 1370, 1246, 1198, 1150, 1030, 744, 700.

ethyl 3-benzyl-4-(pyrrolidin-1-yl)butanoate (19)

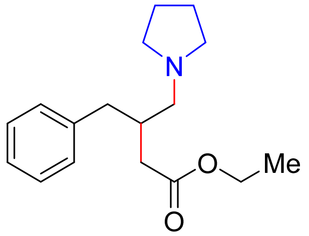

Prepared following the general procedure employing pyrrolidine (71 mg, 1 mmol), 3-phenylpropanal (201 mg, 1.5 mmol), ethyl 2-mercaptoacetate (180 mg, 1.5 mmol), triphenylphosphine (393 mg, 1.5 mmol), 4Å MS (1 g), di-tert-butyl peroxide (292 mg, 2 mmol) in anhydrous dichloromethane (6 mL). The desired product was obtained as colorless oil (239 mg, 87% yield) after purification by flash column chromatography on silica gel (0.5% Et_3N in dichloromethane: methanol = 20:1).

^{1}H NMR (400 MHz, $CDCl_3$) δ 7.28 - 7.23 (m, 2H), 7.17 (dd, J = 10.4, 4.5 Hz, 3H), 4.03 (q, J = 7.1 Hz, 2H), 2.74 (dd, J = 13.5, 4.8 Hz, 1H), 2.59 - 2.28 (m, 9H), 2.20 (dd, J = 14.9, 5.8 Hz, 1H), 1.80 - 1.63 (m, 4H), 1.21 (t, J = 7.1 Hz, 3H). ^{13}C NMR (101 MHz, $CDCl_3$) δ 173.4, 140.0, 129.5, 128.4, 126.1, 60.3, 60.2, 54.3, 39.1, 37.7, 36.6, 23.7, 14.4. HRMS (ESI) calcd for $C_{17}H_{26}NO_2$ [M + H]$^+$ m/z = 276.1958, found: 276.1958. IR ν_{max}/cm^{-1} (film): 2960, 2928, 1734, 1451, 1370, 1255, 1151, 1030, 742, 700.

ethyl 3-benzyl-4-(2-(methoxymethyl)pyrrolidin-1-yl)butanoate (20)

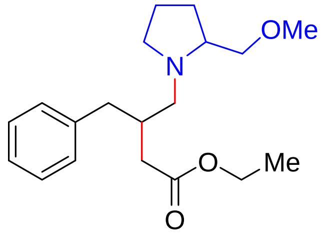

Prepared following the general procedure employing 2-(methoxymethyl) pyrrolidine (115 mg, 1 mmol), 3-phenylpropanal (201 mg, 1.5 mmol), ethyl 2-mercaptoacetate (180 mg, 1.5 mmol), triphenylphosphine (393 mg, 1.5 mmol), 4Å MS (1 g), di-tert-butyl peroxide (292 mg, 2 mmol) in anhydrous dichloromethane (6 mL). The desired product was obtained as colorless oil (274 mg,

86% yield) after purification by flash column chromatography on silica gel (0.5% Et_3N in ethyl acetate). The dr value (1.5:1) was based on ^1H NMR spectra.

^1H NMR (400 MHz, $CDCl_3$) δ 7.27 - 7.08 (m, 5H), 4.08 - 3.93 (m, 2H), 3.37 - 3.32 (m, 0.6H), 3.30 (s, 1.2 H, minor diastereomer)), 3.29 (s, 1.8H, major diastereomer), 3.16 - 3.12 (m, 0.4H), 3.10 - 2.93 (m, 1H), 2.70 - 2.09 (m, 8H), 2.02 - 1.41 (m, 5H), 1.19 (dt, J = 8.7, 7.1 Hz, 3H). ^{13}C NMR (101 MHz, $CDCl_3$, double carbon signals) δ 173.4, 173.0, 140.3, 140.0, 129.4, 129.2, 128.2, 128.1, 125.9, 125.8, 76.9, 76.5, 64.0, 63.4, 60.0, 60.0, 59.9, 59.8, 58.9, 58.9, 54.8, 54.4, 39.5, 38.4, 37.7, 37.2, 37.0, 36.7, 28.7, 28.6, 23.2, 23.2, 14.2, 14.2. HRMS (ESI) calcd for $C_{19}H_{30}NO_3$ $[M + H]^+$ m/z = 320.2220, found: 320.2222. IR ν_{max}/cm^{-1} (film): 2929, 2809, 1732, 1453, 1372, 1254, 1198, 1151, 1119, 1031, 745, 700.

ethyl 3-benzyl-4-(2-phenylpyrrolidin-1-yl) butanoate (21)

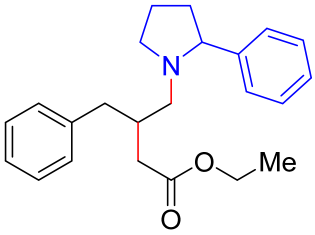

Prepared following the general procedure employing 2-phenylpyrrolidine (147 mg, 1 mmol), 3-phenylpropanal (201 mg, 1.5 mmol), ethyl 2-mercaptoacetate (180 mg, 1.5 mmol), triphenylphosphine (393 mg, 1.5 mmol), 4Å MS (1 g), di-tert-butyl peroxide (292 mg, 2 mmol) in anhydrous dichloromethane (6 mL). The desired product was obtained as colorless oil (291 mg, 83% yield) after purification by flash column chromatography on silica gel (0.5% Et_3N in dichloromethane). The dr value (7:1) was based on HPLC spectra.

^1H NMR (400 MHz, $CDCl_3$) δ 7.36 - 7.12 (m, 8H), 7.07 (d, J = 7.1 Hz, 2H), 4.15 - 3.95 (m, 2H), 3.32 (td, J = 8.5, 2.4 Hz, 1H), 3.16 (t, J = 8.2 Hz, 1H), 2.47 (m, 4H), 2.26 (t, J = 11.1 Hz, 1H), 2.17 - 1.94 (m, 3H), 1.94 - 1.51 (m, 4H), 1.25 (t, J = 7.1 Hz, 3H). ^{13}C NMR (101 MHz, $CDCl_3$) δ 173.7, 144.4, 140.3, 129.2, 128.4, 128.3, 127.7, 127.0, 126.0, 70.5, 60.1, 58.8, 53.7, 39.8, 37.8, 36.6, 35.6, 22.8, 14.4. HRMS (ESI) calcd for $C_{23}H_{30}NO_2$ $[M + H]^+$ m/z = 352.2271, found: 352.2276. IR ν_{max}/cm^{-1} (film): 2965, 1732, 1493, 1452, 1371, 1198, 1150, 1118, 1029, 912, 747, 700.

ethyl 3-benzyl-4-(piperidin-1-yl) butanoate (22)

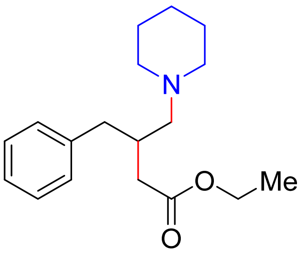

Prepared following the general procedure employing piperidine (85 mg, 1 mmol), 3-phenylpropanal (201 mg, 1.5 mmol), ethyl 2-mercaptoacetate (180 mg, 1.5 mmol), triphenylphosphine (393 mg, 1.5 mmol), 4Å MS (1 g), di-tert-butyl peroxide (292 mg, 2 mmol) in anhydrous dichloromethane (6 mL). The desired product was obtained as colorless oil (260 mg,

90% yield) after purification by flash column chromatography on silica gel (0.5% Et_3N in dichloromethane: methanol = 20:1).

^{1}H NMR (400 MHz, $CDCl_3$) δ 7.28 - 7.22 (m, 2H), 7.18 - 7.15(m, 3H), 4.12 - 3.99 (m, 2H), 2.69 (dd, J = 13.5, 6.0 Hz, 1H), 2.54 (dd, J = 13.5, 7.3 Hz, 1H), 2.50 - 2.12 (m, 8H), 2.09 (dd, J = 12.4, 8.4 Hz, 1H), 1.56 - 1.44 (m, 4H), 1.38 (m, 2H), 1.22 (t, J = 7.1 Hz, 3H). ^{13}C NMR (101 MHz, $CDCl_3$) δ 173.4, 140.1, 129.4, 128.2, 126.0, 63.4, 60.1, 55.0, 39.1, 37.8, 34.8, 26.2, 24.6, 14.3. HRMS (ESI) calcd for $C_{18}H_{28}NO_2$ [M + H]$^+$ m/z = 290.2115, found: 290.2117. IR ν_{max}/cm^{-1} (film): 2934, 1733, 1453, 1372, 1298, 1210, 1155, 1114, 1038, 744, 700.

ethyl 3-benzyl-4-(octahydroquinolin-1(2H)-yl)butanoate (23)

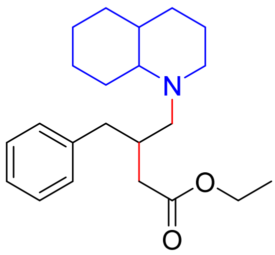

Prepared following the general procedure employing decahydroquinoline (139 mg, 1 mmol), 3-phenylpropanal (201 mg, 1.5 mmol), ethyl 2-mercaptoacetate (180 mg, 1.5 mmol), triphenylphosphine (393 mg, 1.5 mmol), 4Å MS (1 g), di-tert-butyl peroxide (292 mg, 2 mmol) in anhydrous dichloromethane (6 mL). The desired product was obtained as colorless oil (274 mg, 80% yield) after purification by flash column chromatography on silica gel (0.5% Et_3N in petroleum ether: ethyl acetate = 6:1). The dr value (2:1) was determined by ^{1}H NMR spectra.

^{1}H NMR (400 MHz, $CDCl_3$) δ 7.28 - 7.10 (m, 5H), 4.04 (qd, J = 7.1, 1.4 Hz, 2H), 2.99 – 2.88 (m, 1.2H, major diastereomer), 2.88 – 2.78 (m, 0.6H, minor diastereomer), 2.70 – 1.99 (m, 8H), 1.93 - 1.78 (m, 2H), 1.66 - 1.44 (m, 6H), 1.22 - 0.87 (m, 8H). ^{13}C NMR (101 MHz, $CDCl_3$, double carbon signals) δ 173.6, 173.5, 140.6, 140.4, 129.5, 129.3, 128.3, 128.3, 126.0, 126.0, 68.4, 67.4, 60.2, 60.1, 57.6, 56.9, 55.7, 54.8, 42.4, 42.0, 39.6, 39.1, 38.2, 37.7, 37.5, 36.1, 33.5, 33.4, 32.8, 31.7, 31.1, 30.9, 30.3, 29.8, 26.2, 26.2, 26.0, 26.0, 25.7, 14.4. HRMS (ESI) calcd for $C_{22}H_{34}NO_2$ [M + H]$^+$ m/z = 344.2584, found: 344.2587. IR ν_{max}/cm^{-1} (film): 2924, 2853, 1733, 1451, 1185, 1148, 1031, 748, 699.

ethyl 1-(2-benzyl-4-ethoxy-4-oxobutyl)piperidine-4-carboxylate (24)

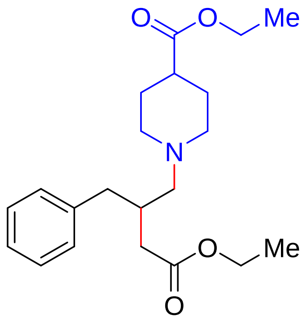

Prepared following the general procedure employing ethyl piperidine-4-carboxylate (157 mg, 1 mmol), 3-phenylpropanal (201 mg, 1.5 mmol), ethyl 2-mercaptoacetate (180 mg, 1.5 mmol), triphenylphosphine (393 mg, 1.5 mmol), 4Å MS (1 g), di-tert-butyl peroxide (292 mg, 2 mmol) in anhydrous dichloromethane (6 mL). The desired product was obtained as colorless oil (318 mg,

88% yield) after purification by flash column chromatography on silica gel (0.5% Et_3N in petroleum ether: ethyl acetate = 8:1).

^{1}H NMR (400 MHz, $CDCl_3$) δ 7.25 - 6.99 (m, 5H), 4.11 - 3.95 (m, 4H), 2.76 (dd, J = 21.4, 9.5 Hz, 2H), 2.63 (dd, J = 8.9, 4.5 Hz, 1H), 2.55 - 2.34 (m, 2H), 2.27 - 1.90 (m, 6H), 1.86 - 1.55 (m, 5H), 1.27 - 1.10 (m, 6H). ^{13}C NMR (101 MHz, $CDCl_3$) δ 175.0, 173.0, 139.6, 129.2, 128.0, 125.8, 62.5, 60.0, 59.8, 53.7, 52.7, 41.1, 38.8, 37.5, 34.7, 28.4, 28.2, 14.1, 14.1. HRMS (ESI) calcd for $C_{21}H_{32}NO_4$ $[M + H]^+$ m/z = 362.2326, found: 362.2327. IR ν_{max}/cm^{-1} (film): 2946, 1732, 1452, 1374, 1258, 1179, 1046, 745, 701.

ethyl 4-([1,4'-bipiperidin]-1'-yl)-3-benzylbutanoate (25)

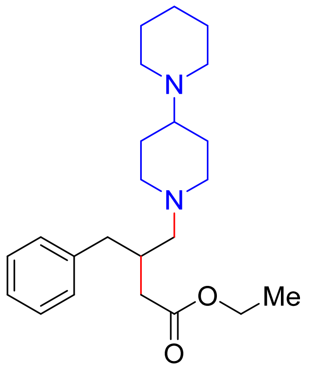

Prepared following the general procedure employing 1,4'-bipiperidine (168 mg, 1 mmol), 3-phenylpropanal (201 mg, 1.5 mmol), ethyl 2-mercaptoacetate (180 mg, 1.5 mmol), triphenylphosphine (393 mg, 1.5 mmol), 4Å MS (1 g), di-tert-butyl peroxide (292 mg, 2 mmol) in anhydrous dichloromethane (6 mL). The desired product was obtained as colorless oil (290 mg, 78% yield) after purification by flash column chromatography on silica gel (0.5% Et_3N in dichloromethane: methanol = 20:1).

^{1}H NMR (400 MHz, $CDCl_3$) δ 7.30 - 6.96 (m, 5H), 4.00 (dq, J = 10.9, 7.2 Hz, 2H), 2.86 (t, J = 11.1 Hz, 2H), 2.71 - 2.43 (m, 6H), 2.43 - 2.02 (m, 6H), 1.98 - 1.72 (m, 4H), 1.71 - 1.58 (m, 4H), 1.58 - 1.34 (m, 4H), 1.17 (t, J = 7.1 Hz, 3H). ^{13}C NMR (101 MHz, $CDCl_3$) δ 173.2, 139.8, 129.3, 128.2, 126.0, 63.2, 62.2, 60.1, 53.9, 52.9, 50.0, 38.9, 37.6, 34.9, 27.5, 27.3, 25.4, 24.2, 14.2. HRMS (ESI) calcd for $C_{23}H_{37}N_2O_2$ $[M + H]^+$ m/z = 373.2850, found: 373.2850. IR ν_{max}/cm^{-1} (film): 2932, 1732, 1452, 1373, 1148, 1112, 1030, 744, 700.

ethyl 3-benzyl-4-(4-bromopiperidin-1-yl)butanoate (26)

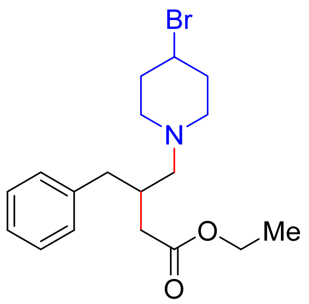

Prepared following the general procedure employing 4-bromopiperidine (163 mg, 1 mmol), 3-phenylpropanal (201 mg, 1.5 mmol), ethyl 2-mercaptoacetate (180 mg, 1.5 mmol), triphenylphosphine (393 mg, 1.5 mmol), 4Å MS (1 g), di-tert-butyl peroxide (292 mg, 2 mmol) in

anhydrous dichloromethane (6 mL). The desired product was obtained as colorless oil (304 mg, 83% yield) after purification by flash column chromatography on silica gel (0.5% Et_3N in dichloromethane).

1H NMR (400 MHz, CDCl3) δ 7.30 - 7.09 (m, 5H), 4.20 - 3.94 (m, 3H), 2.79 - 2.59 (m, 3H), 2.54 (dd, J = 13.6, 7.2 Hz, 1H), 2.47 - 2.33 (m, 1H), 2.32 - 2.02 (m, 8H), 2.01 - 1.89 (m, 2H), 1.22 (t, J = 7.1 Hz, 3H). 13C NMR (101 MHz, CDCl3) δ 173.4, 139.9, 129.4, 128.4, 126.2, 62.4, 60.3, 52.6, 50.4, 39.1, 37.8, 36.7, 35.0, 14.4. HRMS (ESI) calcd for $C_{18}H_{27}BrNO_2$ [M + H] + m/z = 368.1220, found: 368.1222. IR ν_{max}/cm^{-1} (film): 2949, 1731, 1451, 1371, 1300, 1250, 1195, 1149, 1031, 996, 743, 700.

ethyl 3-benzyl-4-(4-cyanopiperidin-1-yl)butanoate (27)

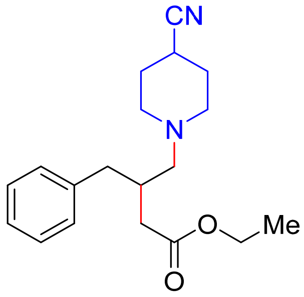

Prepared following the general procedure employing piperidine-4-carbonitrile (110 mg, 1 mmol), 3-phenylpropanal (201 mg, 1.5 mmol), ethyl 2-mercaptoacetate (180 mg, 1.5 mmol), triphenylphosphine (393 mg, 1.5 mmol), 4Å MS (1 g), di-tert-butyl peroxide (292 mg, 2 mmol) in anhydrous dichloromethane (6 mL). The desired product was obtained as colorless oil (270 mg, 86% yield) after purification by flash column chromatography on silica gel (0.5% Et_3N in dichloromethane: methanol = 100:1).

^{1}H NMR (400 MHz, $CDCl_3$) δ 7.27 - 7.21 (m, 2H), 7.19 - 7.10 (m, 3H), 4.09 - 4.00 (m, 2H), 2.66 - 2.50 (m, 5H), 2.46 - 2.34 (m, 1H), 2.34 - 2.04 (m, 6H), 1.90 - 1.70 (m, 4H), 1.21 (t, J = 7.1 Hz, 3H). ^{13}C NMR (101 MHz, $CDCl_3$) δ 173.2, 139.7, 129.3, 128.3, 126.1, 121.9, 62.6, 60.1, 51.9, 51.7, 38.9, 37.7, 34.7, 29.0, 28.9, 26.2, 14.3. HRMS (ESI) calcd for $C_{19}H_{27}N_2O_2$ $[M + H]^+$ m/z = 315.2067, found: 315.2067. IR ν_{max}/cm^{-1} (film): 2950, 2931, 1729, 1452, 1373, 1252, 1201, 1139, 1040, 746, 701.

ethyl 3-benzyl-4-(4-methoxypiperidin-1-yl)butanoate (28)

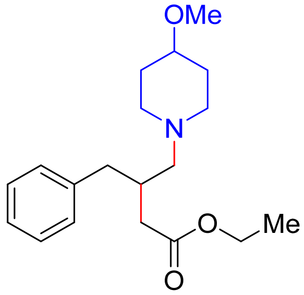

Prepared following the general procedure employing 4-methoxypiperidine (115 mg, 1 mmol), 3-phenylpropanal (201 mg, 1.5 mmol), ethyl 2-mercaptoacetate (180 mg, 1.5 mmol), triphenylphosphine (393 mg, 1.5 mmol), 4Å MS (1 g), di-tert-butyl peroxide (292 mg, 2 mmol) in

anhydrous dichloromethane (6 mL). The desired product was obtained as colorless oil (304 mg, 95% yield) after purification by flash column chromatography on silica gel (0.5% Et_3N in petroleum ether: ethyl acetate = 3:1).

^{1}H NMR (400 MHz, $CDCl_3$) δ 7.27 - 7.06 (m, 5H), 4.08 - 3.94 (m, 2H), 3.25 (s, 3H), 3.15 - 3.01 (m, 1H), 2.74 - 2.44 (m, 4H), 2.44 - 2.32 (m, 1H), 2.29 - 2.01 (m, 5H), 1.99 - 1.72 (m, 3H), 1.53 - 1.39 (m, 2H), 1.17 (t, J = 7.1 Hz, 3H). ^{13}C NMR (101 MHz, $CDCl_3$) δ 173.1, 139.7, 129.2, 128.1, 125.9, 76.5, 62.3, 59.9, 55.3, 51.7, 51.1, 38.9, 37.6, 34.9, 31.0, 30.9, 14.2. HRMS (ESI) calcd for $C_{19}H_{30}NO_3$ [M + H]$^+$ m/z = 320.2220, found: 320.2221. IR ν_{max}/cm^{-1} (film): 2942, 2818, 1732, 1495, 1453, 1371, 1139, 1099, 1036, 946, 785, 745, 700.

ethyl 3-benzyl-4-(4-ethylpiperazin-1-yl)butanoate (29)

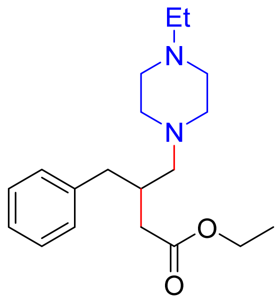

Prepared following the general procedure employing 1-ethylpiperazine (114 mg, 1 mmol), 3-phenylpropanal (201 mg, 1.5 mmol), ethyl 2-mercaptoacetate (180 mg, 1.5 mmol), triphenylphosphine (393 mg, 1.5 mmol), 4Å MS (1 g), di-tert-butyl peroxide (292 mg, 2 mmol) in anhydrous dichloromethane (6 mL). The desired product was obtained as colorless oil (245 mg, 77% yield) after purification by flash column chromatography on silica gel (0.5% Et_3N in ethyl acetate: petroleum ether = 3:1).

^{1}H NMR (400 MHz, $CDCl_3$) δ 7.27 (t, J = 7.3 Hz, 2H), 7.21 - 7.14 (m, 3H), 4.11 - 4.01 (m, 2H), 2.72 (dd, J = 13.4, 5.7 Hz, 1H), 2.57 - 2.35 (m, 10H), 2.33 - 2.12 (m, 6H), 1.23 (t, J = 7.1 Hz, 3H), 1.07 (t, J = 7.2 Hz, 3H). ^{13}C NMR (101 MHz, $CDCl_3$) δ 173.4, 139.8, 129.5, 128.3, 126.1, 62.6, 60.2, 53.5, 53.1, 52.5, 39.0, 37.7, 34.7, 14.4, 12.1. HRMS (ESI) calcd for $C_{19}H_{31}N_2O_2$ [M + H]$^+$ m/z = 319.2380, found: 319.2385. IR ν_{max}/cm^{-1} (film): 2935, 2808, 1732, 1452, 1376, 1300, 1165, 1038, 1014, 744, 701.

benzyl 4-(2-benzyl-4-ethoxy-4-oxobutyl)piperazine-1-carboxylate (30)

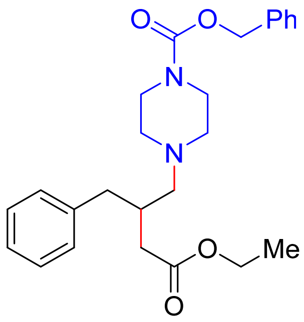

Prepared following the general procedure employing benzyl piperazine-1-carboxylate (220 mg, 1 mmol), 3-phenylpropanal (201 mg, 1.5 mmol), ethyl 2-mercaptoacetate (180 mg, 1.5 mmol), triphenylphosphine (393 mg, 1.5 mmol), 4Å MS (1 g), di-tert-butyl peroxide (292 mg, 2 mmol) in

anhydrous dichloromethane (6 mL). The desired product was obtained as colorless oil (361 mg, 85% yield) after purification by flash column chromatography on silica gel (0.5% Et_3N in petroleum ether: ethyl acetate = 3:1).

^{1}H NMR (400 MHz, $CDCl_3$) δ 7.45 - 7.11 (m, 10H), 5.14 (s, 2H), 4.17 - 4.02 (m, 2H), 3.47 (br, 4H), 2.70 (dd, J = 13.5, 6.1 Hz, 1H), 2.58 (dd, J = 13.5, 7.2 Hz, 1H), 2.52 - 2.38 (m, 3H), 2.37 - 2.09 (m, 6H), 1.24 (t, J = 7.1 Hz, 3H). ^{13}C NMR (101 MHz, $CDCl_3$) δ 173.1, 155.2, 139.6, 136.8, 129.3, 128.5, 128.4, 128.4, 128.3, 128.0, 127.8, 126.1, 67.0, 62.6, 60.2, 53.2, 43.9, 39.0, 37.7, 34.5, 14.3. HRMS (ESI) calcd for $C_{25}H_{33}N_2O_4$ [M + H]$^+$ m/z = 425.2435, found: 425.2438. IR ν_{max}/cm^{-1} (film): 2939, 1729, 1702, 1496, 1428, 1364, 1291, 1236, 1126, 1028, 1005, 746, 699.

ethyl 3-benzyl-4-thiomorpholinobutanoate (31)

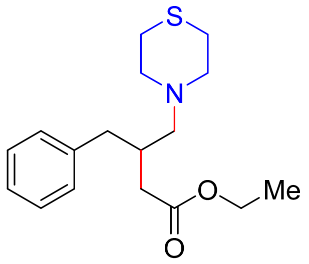

Prepared following the general procedure employing thiomorpholine (103 mg, 1 mmol), 3-phenylpropanal (201 mg, 1.5 mmol), ethyl 2-mercaptoacetate (180 mg, 1.5 mmol), triphenylphosphine (393 mg, 1.5 mmol), 4Å MS (1 g), di-tert-butyl peroxide (292 mg, 2 mmol) in anhydrous dichloromethane (6 mL). The desired product was obtained as colorless oil (230 mg, 75% yield) after purification by flash column chromatography on silica gel (0.5% Et_3N in dichloromethane: methanol = 100:1).

^{1}H NMR (400 MHz, $CDCl_3$) δ 7.41 - 6.98 (m, 5H), 4.10 - 4.03 (m, 2H), 2.97 - 2.33 (m, 11H), 2.33 - 2.16 (m, 3H), 2.12 (dd, J = 12.6, 8.6 Hz, 1H), 1.22 (t, J = 7.1 Hz, 3H). ^{13}C NMR (101 MHz, $CDCl_3$) δ 173.3, 139.8, 129.4, 128.4, 126.2, 63.4, 60.2, 55.6, 39.0, 37.8, 34.8, 28.1, 14.4. HRMS (ESI) calcd for $C_{17}H_{26}NO_2S$ [M + H]$^+$ m/z = 308.1679, found: 308.1682. IR ν_{max}/cm^{-1} (film): 2928, 2806, 1731, 1453, 1372, 1281, 1150, 1124, 1030, 958, 745, 700.

ethyl 3-benzyl-4-morpholinobutanoate (32)

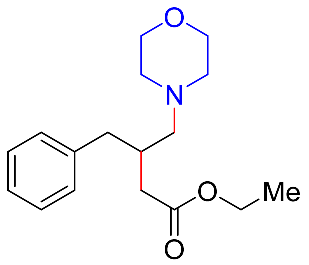

Prepared following the general procedure employing morpholine (87 mg, 1 mmol), 3-phenylpropanal (201 mg, 1.5 mmol), ethyl 2-mercaptoacetate (180 mg, 1.5 mmol), triphenylphosphine (393 mg, 1.5 mmol), 4Å MS (1 g), di-tert-butyl peroxide (292 mg, 2 mmol) in anhydrous dichloromethane (6 mL). The desired product was obtained as colorless oil (259 mg, 89% yield) after purification by flash column chromatography on silica gel (0.5% Et_3N in dichloromethane: methanol = 50:1).

^{1}H NMR (400 MHz, $CDCl_3$) δ 7.25 (t, J = 7.3 Hz, 2H), 7.1 - 7.12 (m, 3H), 4.06 (qd, J = 6.8, 1.0 Hz, 2H), 3.62 (t, J = 4.6 Hz, 4H), 2.68 (dd, J = 13.5, 6.1 Hz, 1H), 2.54 (dd, J = 13.5, 7.3 Hz, 1H), 2.50 - 2.07 (m, 9H), 1.21 (t, J = 7.1 Hz, 3H). ^{13}C NMR (101 MHz, $CDCl_3$) δ 173.2, 139.7, 129.4, 128.3, 126.1, 67.1, 63.0, 60.2, 54.0, 39.0, 37.7, 34.3, 14.3. HRMS (ESI) calcd for $C_{17}H_{26}NO_3$ [M + H]$^+$ m/z = 292.1907, found: 292.1911. IR ν_{max}/cm^{-1} (film): 2956, 2854, 1731, 1454, 1372, 1201, 1144, 1118, 1034, 1010, 863, 746, 701.

ethyl 3-benzyl-4-(4-hydroxypiperidin-1-yl)butanoate (33)

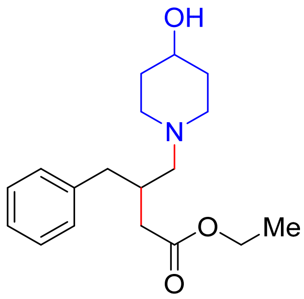

Prepared following the general procedure employing piperidin-4-ol (101 mg, 1 mmol), 3-phenylpropanal (201 mg, 1.5 mmol), ethyl 2-mercaptoacetate (180 mg, 1.5 mmol), triphenylphosphine (393 mg, 1.5 mmol), 4Å MS (1 g), di-tert-butyl peroxide (292 mg, 2 mmol) in anhydrous dichloromethane (6 mL). The desired product was obtained as colorless oil (258 mg, 85% yield) after purification by flash column chromatography on silica gel (0.5% Et_3N in petroleum: ethyl acetate = 1:1).

^{1}H NMR (400 MHz, $CDCl_3$) δ 7.27 - 7.13 (m, 5H), 4.08 - 4.02 (m, 2H), 3.68 - 3.53 (m, 1H), 2.82 - 2.59 (m, 3H), 2.54 (dd, J = 13.6, 7.2 Hz, 1H), 2.48 - 2.33 (m, 1H), 2.33 - 2.03 (m, 5H), 2.03 - 1.75 (m, 3H), 1.58 - 1.42 (m, 2H), 1.21 (t, J = 7.2 Hz, 3H). ^{13}C NMR (101 MHz, $CDCl_3$) δ 173.4, 140.0, 129.4, 128.4, 126.1, 68.3, 62.4, 60.2, 51.9, 51.3, 39.1, 37.8, 35.1, 34.7, 34.6, 14.4. HRMS (ESI) calcd for $C_{18}H_{28}NO_3$ [M + H]$^+$ m/z = 306.2064, found: 306.2064. IR ν_{max}/cm^{-1} (film): 3414, 2924, 2852, 1731, 1452, 1370, 1149, 1066, 1041, 744, 700.

ethyl 3-benzyl-4-((4-methoxyphenyl)(methyl)amino)butanoate (34)

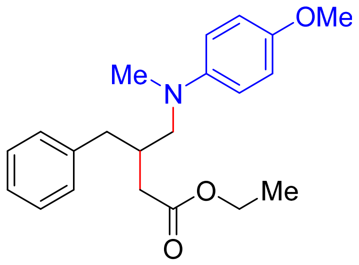

Prepared following the general procedure employing 4-methoxy-N-methylaniline (137 mg, 1 mmol), 3-phenylpropanal (201 mg, 1.5 mmol), ethyl 2-mercaptoacetate (180 mg, 1.5 mmol), triethyl phosphite (249 mg, 1.5 mmol), 4Å MS (1 g), di-tert-butyl peroxide (292 mg, 2 mmol) in anhydrous dichloromethane (6 mL). The desired product was obtained as pink oil (256 mg, 75% yield) after purification by flash column chromatography on silica gel (0.5% Et_3N in petroleum ether: ethyl acetate = 15:1).

^{1}H NMR (400 MHz, $CDCl_3$) δ 7.31 - 7.12 (m, 5H), 6.79 (d, J = 9.1 Hz, 2H), 6.64 (d, J = 9.1 Hz, 2H), 4.08 - 3.94 (m, 2H), 3.74 (s, 3H), 3.18 - 3.03 (m, 2H), 2.82 (s, 3H), 2.74 (dd, J = 12.2, 5.1 Hz, 1H), 2.66 - 2.49 (m, 2H), 2.28 (d, J = 6.3 Hz, 2H), 1.16 (t, J = 7.1 Hz, 3H). ^{13}C NMR (101 MHz, $CDCl_3$) δ 173.0, 151.9, 145.1, 139.9, 129.4, 128.5, 126.3, 114.8, 60.4, 58.5, 56.0, 40.2, 38.9, 37.0, 36.6, 14.3. HRMS (ESI) calcd for $C_{21}H_{28}NO_3$ $[M + H]^+$ m/z = 342.2064, found: 342.2063. IR ν_{max}/cm^{-1} (film): 2964, 1734, 1512, 1438, 1258, 1036, 912, 800, 742, 701.

ethyl 3-benzyl-4-(methyl(p-tolyl)amino)butanoate (35)

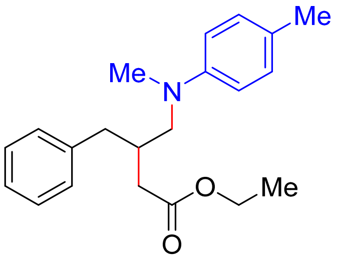

Prepared following the general procedure employing N,4-dimethylaniline (121mg, 1 mmol), 3-phenylpropanal (201 mg, 1.5 mmol), ethyl 2-mercaptoacetate (180 mg, 1.5 mmol), triethyl phosphite (249 mg, 1.5 mmol), 4Å MS (1 g), di-tert-butyl peroxide (292 mg, 2 mmol) in anhydrous dichloromethane (6 mL). The desired product was obtained as pink oil (234 mg, 72% yield) after purification by flash column chromatography on silica gel (0.5% Et_3N in petroleum ether: ethyl acetate = 40:1).

^{1}H NMR (400 MHz, $CDCl_3$) δ 7.35 - 7.13 (m, 5H), 7.01 (d, J = 8.3 Hz, 2H), 6.59 (d, J = 8.6 Hz, 2H), 4.08 - 3.96 (m, 2H), 3.28 - 3.13 (m, 2H), 2.88 (s, 3H), 2.75 (dd, J = 12.4, 5.5 Hz, 1H), 2.70 - 2.53 (m, 2H), 2.29 (d, J = 6.3 Hz, 2H), 2.24 (s, 3H), 1.18 (t, J = 7.1 Hz, 3H). ^{13}C NMR (101 MHz, $CDCl_3$) δ 172.9, 148.0, 139.9, 129.7, 129.3, 128.5, 126.3, 125.8, 112.9, 60.4, 57.6, 39.7, 38.8, 36.9, 36.7, 20.3, 14.3. HRMS (ESI) calcd for $C_{21}H_{28}NO_2$ $[M + H]^+$ m/z = 326.2115, found: 326.2117. IR ν_{max}/cm^{-1} (film): 2978, 1734, 1617, 1521, 1451, 1370, 1150, 1029, 801, 743, 700.

ethyl 3-benzyl-4-(6-methoxy-3,4-dihydroquinolin-1(2H)-yl)butanoate (36)

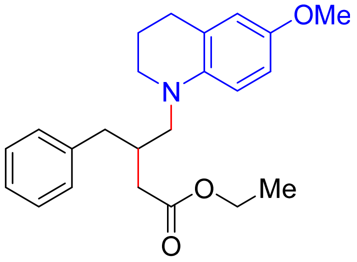

Prepared following the general procedure employing 6-methoxy-1, 2, 3, 4-tetrahydroquinoline (163 mg, 1 mmol), 3-phenylpropanal (201 mg, 1.5 mmol), ethyl 2-mercaptoacetate (180 mg, 1.5 mmol), triethyl phosphite (249 mg, 1.5 mmol), 4Å MS (1 g), di-tert-butyl peroxide (292 mg, 2 mmol) in anhydrous dichloromethane (6 mL). The desired product was obtained as pink oil (146.8 mg, 40% yield) after purification by flash column chromatography on silica gel (0.5% Et_3N in petroleum ether: ethyl acetate = 40:1).

^{1}H NMR (400 MHz, $CDCl_3$) δ 7.29 - 7.15 (m, 5H), 6.63 - 6.51 (m, 2H), 6.44 (d, J = 8.8 Hz, 1H), 4.01 (qd, J = 7.1, 1.0 Hz, 2H), 3.70 (s, 3H), 3.23 - 3.01 (m, 4H), 2.81 - 2.48 (m, 5H), 2.28 (d, J =

6.4 Hz, 2H), 1.94 - 1.83 (m, 2H), 1.16 (t, J = 7.1 Hz, 3H). ^{13}C NMR (101 MHz, $CDCl_3$) δ 173.0, 151.0, 140.8, 140.0, 129.4, 128.5, 126.3, 124.2, 115.4, 112.6, 112.5, 60.4, 57.2, 56.0, 50.9, 38.9, 37.0, 36.6, 28.4, 22.4, 14.3. HRMS (ESI) calcd for $C_{23}H_{30}NO_3$ [M + H]$^+$ m/z = 368.2220, found: 368.2224. IR ν_{max}/cm^{-1} (film): 2944, 1734, 1495, 1451, 1370, 1151, 1029, 742, 700.

ethyl 4-(benzyl(methyl)amino) butanoate (37)

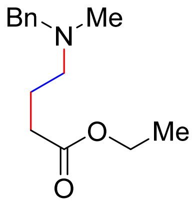

Prepared following the general procedure employing N-methyl-1-phenylmethanamine (121mg, 1 mmol), acetaldehyde (5 mol/L in THF) (0.4 ml, 2 mmol), ethyl 2-mercaptoacetate (180 mg, 1.5 mmol), triphenylphosphine (393 mg, 1.5 mmol), Potassium carbonate (276 mg, 2 mmol), di-tert-butyl peroxide (292 mg, 2 mmol) in anhydrous THF (6 mL). The desired product was obtained as colorless oil (211 mg, 90% yield) after purification by flash column chromatography on silica gel (0.5% Et_3N in dichloromethane).

^{1}H NMR (400 MHz, $CDCl_3$) δ 7.37 - 7.12 (m, 5H), 4.09 (q, J = 7.1 Hz, 2H), 3.45 (s, 2H), 2.37 (t, J = 7.3 Hz, 2H), 2.33 (t, J = 7.3 Hz, 2H), 2.16 (s, 3H), 1.81 (p, J = 7.3 Hz, 2H), 1.22 (t, J = 7.1 Hz, 3H). ^{13}C NMR (101 MHz, $CDCl_3$) δ 173.7, 139.3, 129.0, 128.2, 126.9, 62.4, 60.2, 56.4, 42.0, 32.1, 22.7, 14.3. HRMS (ESI) calcd for $C_{14}H_{22}NO_2$ [M + H]$^+$ m/z = 236.1645, found: 236.1646. IR ν_{max}/cm^{-1} (film): 2978, 2790, 1735, 1452, 1370, 1251, 1174, 1122, 1026, 737, 699.

ethyl 3-((benzyl(methyl)amino)methyl)pentanoate (38)

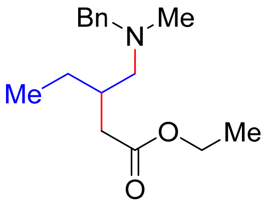

Prepared following the general procedure employing N-methyl-1-phenylmethanamine (121mg, 1 mmol), butyraldehyde (110 mg, 1.5 mmol), ethyl 2-mercaptoacetate (180 mg, 1.5 mmol), triphenylphosphine (393 mg, 1.5 mmol), 4Å MS (1 g), di-tert-butyl peroxide (292 mg, 2 mmol) in anhydrous dichloromethane (6 mL). The desired product was obtained as colorless oil (239 mg, 91% yield) after purification by flash column chromatography on silica gel (0.5% Et_3N in dichloromethane).

^{1}H NMR (400 MHz, $CDCl_3$) δ 7.39 - 6.96 (m, 5H), 4.11 (q, J = 7.2 Hz, 2H), 3.45 (dd, J = 28.7, 13.1 Hz, 2H), 2.42 (dd, J = 15.0, 6.0 Hz, 1H), 2.28 - 2.00 (m, 7H), 1.47 - 1.28 (m, 2H), 1.24 (t, J = 7.1 Hz, 3H), 0.87 (t, J = 7.5 Hz, 3H). ^{13}C NMR (101 MHz, $CDCl_3$) δ 173.8, 139.6, 129.0, 128.2, 126.9, 63.1, 61.8, 60.1, 42.5, 37.7, 35.0, 25.5, 14.4, 11.0. HRMS (ESI) calcd for $C_{16}H_{26}NO_2$ [M + H]$^+$ m/z = 264.1958, found: 264.1960. IR ν_{max}/cm^{-1} (film): 2961, 2788, 1734, 1453, 1371, 1249, 1173, 1036, 740, 699.

ethyl 3-((benzyl(methyl)amino)methyl)-4-methylpentanoate (39)

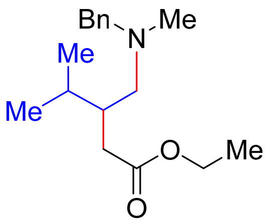

Prepared following the general procedure employing N-methyl-1-phenylmethanamine (121mg, 1 mmol), 3-methylbutanal (129 mg, 1.5 mmol), ethyl 2-mercaptoacetate (180 mg, 1.5 mmol), triphenylphosphine (393 mg, 1.5 mmol), 4Å MS (1 g), di-tert-butyl peroxide (292 mg, 2 mmol) in anhydrous dichloromethane (6 mL). The desired product was obtained as colorless oil (246 mg, 89% yield) after purification by flash column chromatography on silica gel (0.5% Et_3N in dichloromethane).

^1H NMR (400 MHz, $CDCl_3$) δ 7.36 - 7.11 (m, 5H), 4.11 (q, J = 7.1 Hz, 2H), 3.45 (q, J = 13.1 Hz, 2H), 2.37 - 2.16 (m, 5H), 2.13 (s, 3H), 1.89 - 1.74 (m, 1H), 1.25 (t, J = 7.1 Hz, 3H), 0.87 (d, J = 6.9 Hz, 3H), 0.80 (d, J = 6.9 Hz, 3H). ^{13}C NMR (101 MHz, $CDCl_3$) δ 174.2, 139.6, 129.1, 128.1, 126.9, 63.2, 60.1, 59.8, 42.5, 39.0, 35.0, 29.1, 19.8, 18.5, 14.3. HRMS (ESI) calcd for $C_{17}H_{28}O_2$ $[M + H]^+$ m/z = 278.2115, found:278.2116. IR ν_{max}/cm^{-1} (film): 2958, 1734, 1453, 1370, 1266, 1171, 1128, 1035, 740, 699.

ethyl 3-((benzyl(methyl)amino)methyl)-4,4-dimethylpentanoate (40)

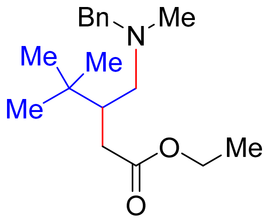

Prepared following the general procedure employing N-methyl-1-phenylmethanamine (121mg, 1 mmol), 3, 3-dimethylbutanal (150 mg, 1.5 mmol), ethyl 2-mercaptoacetate (180 mg, 1.5 mmol), triphenylphosphine (393 mg, 1.5 mmol), 4Å MS (1 g), di-tert-butyl peroxide (292 mg, 2 mmol) in anhydrous dichloromethane (6 mL). The desired product was obtained as colorless oil (247 mg, 85% yield) after purification by flash column chromatography on silica gel (0.5% Et_3N in dichloromethane).

^1H NMR (400 MHz, $CDCl_3$) δ 7.36 - 7.11 (m, 5H), 4.09 (q, J = 7.1 Hz, 2H), 3.57 (d, J =13.0 Hz, 1H), 3.31 (d, J =13.0 Hz, 1H), 2.40 (dd, J = 15.5, 6.3 Hz, 1H), 2.34 - 2.04 (m, 7H), 1.24 (t, J = 7.2 Hz, 3H), 0.86 (s, 9H). ^{13}C NMR (101 MHz, $CDCl_3$) δ 174.8, 139.6, 129.3, 128.1, 126.9, 63.3, 60.2, 59.3, 43.0, 42.4, 35.4, 32.6, 27.7, 14.3. HRMS (ESI) calcd for $C_{18}H_{30}NO_2$ $[M + H]^+$ m/z = 292.2271, found: 292.2274. IR ν_{max}/cm^{-1} (film): 2961, 1733, 1453, 1367, 1255, 1201, 1157, 1034, 739, 699.

ethyl 3-((benzyl(methyl)amino)methyl)decanoate (41)

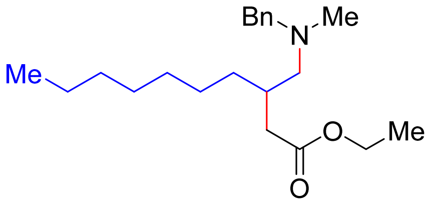

Prepared following the general procedure employing N-methyl-1-phenylmethanamine (121mg, 1 mmol), nonanal (214 mg, 1.5 mmol), ethyl 2-mercaptoacetate (180 mg, 1.5 mmol), triphenylphosphine (393 mg, 1.5 mmol), 4Å MS (1 g), di-tert-butyl peroxide (292 mg, 2 mmol) in anhydrous dichloromethane (6 mL). The desired product was obtained as colorless oil (276 mg, 83% yield) after purification by flash column chromatography on silica gel (0.5% Et_3N in dichloromethane).

^{1}H NMR (400 MHz, $CDCl_3$) δ 7.36 - 7.12 (m, 5H), 4.10 (q, J = 7.1 Hz, 2H), 3.48 (d, J = 13.1 Hz, 1H), 3.41 (d, J = 13.1 Hz, 1H), 2.49 - 2.37 (m, 1H), 2.29 - 2.04 (m, 7H), 1.47 - 1.07 (m, 15H), 0.87 (t, J = 6.8 Hz, 3H). ^{13}C NMR (101 MHz, $CDCl_3$) δ 173.7, 139.4, 128.9, 128.0, 126.8, 63.0, 62.1, 60.0, 42.4, 38.1, 33.6, 32.9, 31.8, 29.9, 29.2, 26.6, 22.6, 14.2, 14.1. HRMS (ESI) calcd for $C_{21}H_{36}NO_2$ $[M + H]^+$ m/z = 334.2741, found: 334.2745. IR ν_{max}/cm^{-1} (film): 2927, 2854, 1734, 1453, 1251, 1162, 1027, 740, 699.

ethyl 3-((benzyl(methyl)amino)methyl)-4,8-dimethylnon-7-enoate (42)

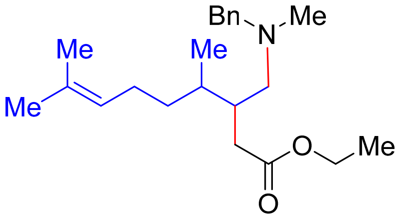

Prepared following the general procedure employing N-methyl-1-phenylmethanamine (121mg, 1 mmol), 3, 7-dimethyloct-6-enal (232 mg, 1.5 mmol), ethyl 2-mercaptoacetate (180 mg, 1.5 mmol), triphenylphosphine (393 mg, 1.5 mmol), 4Å MS (1 g), di-tert-butyl peroxide (292 mg, 2 mmol) in anhydrous dichloromethane (6 mL). The desired product was obtained as colorless oil (297 mg, 86% yield) after purification by flash column chromatography on silica gel (0.5% Et_3N in petroleum ether: ethyl acetate = 4:1). The dr value (2:1) was based on ^{1}H NMR spectra.

^{1}H NMR (400 MHz, $CDCl_3$) δ 7.39 - 7.14 (m, 5H), 5.14 - 4.97 (m, 1H), 4.10 (q, J = 7.1 Hz, 2H), 3.57 – 3.29 (m, 2H), 2.46 - 2.33 (m, 1H), 2.31 - 2.15 (m, 4H), 2.12 (s, 3H), 2.06 - 1.84 (m, 2H), 1.67 (s, 3H), 1.59 (s, 3H), 1.35 - 1.26 (m, 1H), 1.26 - 1.21 (m, 3H), 1.19 - 1.02 (m, 1H), 0.90 - 0.83 (m, 1H), 0.82 (d, J = 6.9 Hz, 2H, major diastereomer), 0.74 (d, J = 6.9 Hz, 1H, minor diastereomer). ^{13}C NMR (101 MHz, $CDCl_3$, double carbon signals) δ 174.2, 174.1, 139.6, 139.6, 131.4, 131.3, 129.1, 129.1, 128.2, 128.2, 126.9, 126.9, 124.8, 124.8, 63.3, 63.1, 60.3, 60.1, 58.8, 58.7, 42.5, 42.5, 38.0, 37.4, 36.0, 34.6, 34.4, 34.0, 33.7, 33.3, 26.3, 26.1, 25.8, 17.8, 17.8, 16.0, 15.9, 15.2, 14.4, 14.3. HRMS (ESI) calcd for $C_{22}H_{36}NO_2$ $[M + H]^+$ m/z = 346.2741, found: 346.2741. IR ν_{max}/cm^{-1} (film): 2961, 1734, 1452, 1374, 1270, 1171, 1035, 740, 699.

ethyl 3-phenyl-4-(pyrrolidin-1-yl)butanoate (43)

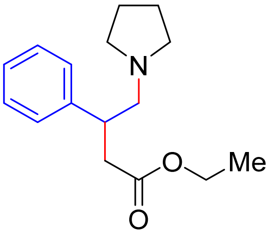

Prepared following the general procedure employing pyrrolidine (72 mg, 1 mmol), 2-phenylacetaldehyde (180 mg, 1.5 mmol), ethyl 2-mercaptoacetate (180 mg, 1.5 mmol), triphenylphosphine (393 mg, 1.5 mmol), 4Å MS (1 g), di-tert-butyl peroxide (292 mg, 2 mmol) in anhydrous dichloromethane (6 mL). The desired product was obtained as colorless oil (219 mg, 84% yield) after purification by flash column chromatography on silica gel (0.5% Et_3N in ethyl acetate).

^{1}H NMR (400 MHz, $CDCl_3$) δ 7.36 - 7.05 (m, 5H), 4.02 - 3.95 (m, 2H), 3.39 - 3.24 (m, 1H), 2.85 (dd, J = 15.2, 6.2 Hz, 1H), 2.76 (dd, J = 12.0, 9.7 Hz, 1H), 2.62 - 2.34 (m, 6H), 1.82 - 1.59 (m, 4H), 1.11 (t, J = 7.1 Hz, 3H). ^{13}C NMR (101 MHz, $CDCl_3$) δ 172.8, 143.2, 128.5, 127.6, 126.7, 62.5, 60.2, 54.3, 41.8, 39.7, 23.7, 14.2. HRMS (ESI) calcd for $C_{16}H_{24}NO_2$ $[M + H]^+$ m/z = 262.1802, found: 262.1805. IR ν_{max}/cm^{-1} (film): 2964, 1732, 1453, 1371, 1253, 1160, 1120, 1030, 761, 700.

ethyl 4-(benzyl(methyl)amino)-3-((methylthio)methyl)butanoate (44)

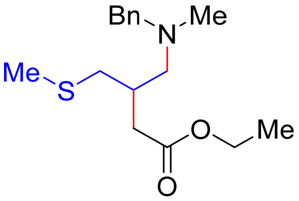

Prepared following the general procedure employing N-methyl-1-phenylmethanamine (121mg, 1 mmol), 3-(methylthio)propanal (157 mg, 1.5 mmol), ethyl 2-mercaptoacetate (180 mg, 1.5 mmol), triphenylphosphine (393 mg, 1.5 mmol), 4Å MS (1 g), di-tert-butyl peroxide (292 mg, 2 mmol) in anhydrous dichloromethane (6 mL). The desired product was obtained as colorless oil (266 mg, 90% yield) after purification by flash column chromatography on silica gel (0.5% Et_3N in dichloromethane).

^{1}H NMR (400 MHz, $CDCl_3$) δ 7.38 - 7.00 (m, 5H), 4.11 (q, J = 7.1 Hz, 2H), 3.46 (s, 2H), 2.64 (dd, J = 13.1, 4.6 Hz, 1H), 2.49 - 2.18 (m, 6H), 2.15 (s, 3H), 2.07 (s, 3H), 1.24 (t, J = 7.1 Hz, 3H). ^{13}C NMR (101 MHz, $CDCl_3$) δ 173.1, 139.3, 129.0, 128.2, 127.0, 63.0, 61.1, 60.3, 42.5, 37.6, 37.0, 33.6, 16.4, 14.3. HRMS (ESI) calcd for $C_{16}H_{26}NO_2S$ $[M + H]^+$ m/z = 296.1679, found: 296.1680. IR ν_{max}/cm^{-1} (film): 2978, 2915, 2790, 1732, 1452, 1372, 1253, 1153, 1024, 740, 699.

ethyl 4-(benzyl(methyl)amino)-3-(benzyloxy)butanoate (45)

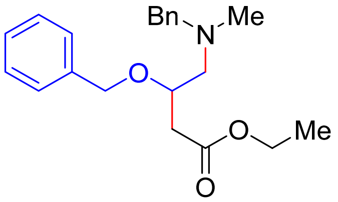

Prepared following the general procedure employing N-methyl-1-phenylmethanamine (121mg, 1 mmol), 2-(benzyloxy) acetaldehyde (225 mg, 1.5 mmol), ethyl 2-mercaptoacetate (180 mg, 1.5

mmol), triphenylphosphine (393 mg, 1.5 mmol), 4Å MS (1 g), di-tert-butyl peroxide (292 mg, 2 mmol) in anhydrous dichloromethane (6 mL). The desired product was obtained as colorless oil (259 mg, 76% yield) after purification by flash column chromatography on silica gel (0.5% Et_3N in petroleum ether: ethyl acetate = 8:1).

^{1}H NMR (400 MHz, $CDCl_3$) δ 7.37 - 7.25 (m, 10H), 4.60 (dd, J = 25.1, 11.4 Hz, 2H), 4.17 - 3.98 (m, 3H), 3.56 - 3.46 (m, 2H), 2.70 (dd, J = 15.5, 4.8 Hz, 1H), 2.59 (dd, J = 12.8, 5.5 Hz, 1H), 2.54 – 2.43 (m, 2H), 2.21 (s, 3H), 1.22 (t, J = 7.1 Hz, 3H). ^{13}C NMR (101 MHz, $CDCl_3$) δ 172.2, 164.4, 139.1, 138.7, 129.1, 128.4, 128.3, 127.9, 127.7, 127.1, 74.8, 72.3, 63.3, 60.8, 60.5, 43.2, 39.2, 14.4. HRMS (ESI) calcd for $C_{21}H_{28}NO_3$ $[M + H]^+$ m/z = 342.2064, found: 342.2068. IR ν_{max}/cm^{-1} (film): 2935, 1734, 1452, 1324, 1256, 1172, 1098, 1027, 740, 698.

ethyl 3-((benzyl(methyl)amino)methyl)-6-chlorohexanoate (46)

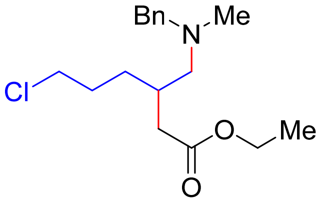

Prepared following the general procedure employing N-methyl-1-phenylmethanamine (121mg, 1 mmol), 5-chloropentanal (180 mg, 1.5 mmol), ethyl 2-mercaptoacetate (180 mg, 1.5 mmol), triphenylphosphine (393 mg, 1.5 mmol), 4Å MS (1 g), di-tert-butyl peroxide (292 mg, 2 mmol) in anhydrous dichloromethane (6 mL). The desired product was obtained as colorless oil (267 mg, 86% yield) after purification by flash column chromatography on silica gel (0.5% Et_3N in dichloromethane).

^{1}H NMR (400 MHz, $CDCl_3$) δ 7.40 - 7.10 (m, 5H), 4.10 (q, J = 7.2 Hz, 2H), 3.60 - 3.33 (m, 4H), 2.49 - 2.06 (m, 8H), 1.83 - 1.68 (m, 2H), 1.54 - 1.36 (m, 2H), 1.24 (t, J = 7.2 Hz, 3H). ^{13}C NMR (101 MHz, $CDCl_3$) δ 171.3, 133.8, 130.7, 129.2, 127.0, 63.4, 61.1, 59.8, 44.3, 37.7, 28.4, 27.6, 20.5, 14.3, 14.3. HRMS (ESI) calcd for $C_{17}H_{27}ClNO_2$ $[M + H]^+$ m/z = 312.1725, found:312.1731. IR ν_{max}/cm^{-1} (film): 2927, 1727, 1458, 1286, 1254, 1189, 1030, 765, 703.

ethyl 2-(2-(pyrrolidin-1-yl)cyclopentyl)acetate (47)

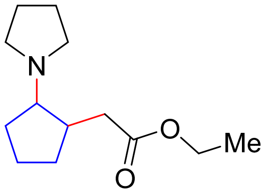

Prepared following the general procedure employing pyrrolidine (72 mg, 1 mmol), cyclopentanone (126 mg, 1.5 mmol), ethyl 2-mercaptoacetate (180 mg, 1.5 mmol), triphenylphosphine (393 mg, 1.5 mmol), 4Å MS (1 g), di-tert-butyl peroxide (292 mg, 2 mmol) in anhydrous dichloromethane (6 mL). The desired product was obtained as colorless oil (153 mg, 68% yield) after purification by flash column chromatography on silica gel (0.5% Et_3N in ethyl acetate). The dr value (1.3:1) was based on HPLC spectra.

^{1}H NMR (400 MHz, $CDCl_3$) δ 4.12 (q, J = 7.2 Hz, 2H), 2.69 - 2.25 (m, 7H), 2.25 - 1.89 (m, 2H), 1.89 - 1.41 (m, 9H), 1.25 (td, J = 7.2, 1.5 Hz, 3H). ^{13}C NMR (101 MHz, $CDCl_3$, double carbon signals) δ 174.4, 173.1, 70.9, 69.8, 60.2, 60.1, 53.8, 52.2, 40.2, 39.1, 33.2, 33.2, 31.7, 30.9, 28.8,

28.7, 23.8, 23.4, 23.4, 20.4, 14.4, 14.3. HRMS (ESI) calcd for $C_{13}H_{24}NO_2$ $[M + H]^+$ m/z = 226.1802, found:226.1802. IR ν_{max}/cm^{-1} (film): 2959, 2873, 2780, 1734, 1371, 1345, 1255, 1177, 1034.

ethyl 2-(2-(pyrrolidin-1-yl)cyclohexyl)acetate (48)

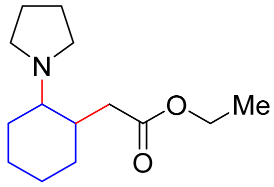

Prepared following the general procedure employing pyrrolidine (72 mg, 1 mmol), cyclohexanone (147 mg, 1.5 mmol), ethyl 2-mercaptoacetate (180 mg, 1.5 mmol), triphenylphosphine (393 mg, 1.5 mmol), 4Å MS (1 g), di-tert-butyl peroxide (292 mg, 2 mmol) in anhydrous dichloromethane (6 mL). The desired product was obtained as colorless oil (170 mg, 71% yield) after purification by flash column chromatography on silica gel (0.5% Et_3N in dichloromethane: methanol = 30:1). The dr value (4:1) was based on HPLC spectra.

^{1}H NMR (400 MHz, $CDCl_3$) δ 4.04 (q, J = 7.1 Hz, 2H), 2.82 - 2.50 (m, 5H), 2.45 (d, J = 11.2 Hz, 1H), 2.33 (dd, J = 15.2, 11.4 Hz, 1H), 2.13 (dd, J = 8.0, 3.7 Hz, 1H), 1.85 - 1.49 (m, 7H), 1.42 - 1.10 (m, 5H), 1.17 (t, J = 7.1 Hz, 3H). ^{13}C NMR (101 MHz, $CDCl_3$, double carbon signals) δ 174.1, 173.7, 67.2, 63.1, 60.2, 59.9, 52.0, 47.8, 39.2, 38.2, 33.9, 32.0, 30.8, 30.2, 28.6, 26.5, 25.3, 25.2, 25.1, 23.8, 23.2, 23.1, 19.7, 14.3. HRMS (ESI) calcd for $C_{14}H_{26}NO_2$ $[M + H]^+$ m/z = 240.1958, found: 240.1956. IR ν_{max}/cm^{-1} (film): 2931, 2862, 1732, 1451, 1367, 1284, 1173, 1033. The dr value (1:1.3) was based on HPLC spectra.

ethyl 2-(2-(pyrrolidin-1-yl)cycloheptyl)acetate (49)

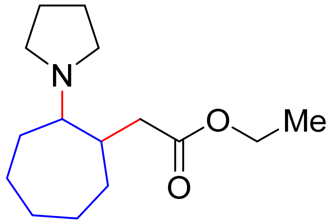

Prepared following the general procedure employing pyrrolidine (72 mg, 1 mmol), cycloheptanone (168 mg, 1.5 mmol), ethyl 2-mercaptoacetate (180 mg, 1.5 mmol), triphenylphosphine (393 mg, 1.5 mmol), 4Å MS (1 g), di-tert-butyl peroxide (292 mg, 2 mmol) in anhydrous dichloromethane (6 mL). The desired product was obtained as colorless oil (187 mg, 74% yield) after purification by flash column chromatography on silica gel (0.5% Et_3N in dichloromethane: methanol = 30:1). The dr value (6:1) was based on HPLC spectra.

^{1}H NMR (400 MHz, $CDCl_3$) δ 4.04 (qd, J = 7.1, 1.0 Hz, 2H), 2.67 – 2.50 (m, 3H), 2.43 (d, J = 7.0 Hz, 2H), 2.37 – 2.26 (m, 1H), 2.19 – 2.06 (m, 2H), 1.77 – 1.28 (m, 14H), 1.22 (t, J = 7.1 Hz, 3H). ^{13}C NMR (101 MHz, $CDCl_3$) δ 174.0, 65.8, 60.0, 48.1, 41.0, 40.0, 31.4, 29.6, 26.0, 25.0, 24.9, 23.9, 14.4. HRMS (ESI) calcd for $C_{15}H_{28}NO_2$ $[M + H]^+$ m/z = 254.2115, found: 254.2119. IR ν_{max}/cm^{-1} (film): 2927, 1731, 1451, 1369, 1267, 1183, 1153, 1028. The dr value (1:6) was based on HPLC spectra.

ethyl 2-(5-phenyl-2-(pyrrolidin-1-yl)cyclohexyl)acetate (50)

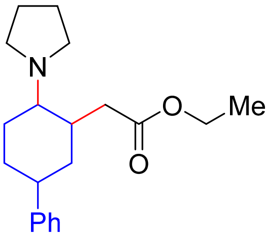

Prepared following the general procedure employing pyrrolidine (72 mg, 1 mmol), 4-phenylcyclohexan-1-one (261 mg, 1.5 mmol), ethyl 2-mercaptoacetate (180 mg, 1.5 mmol), triphenylphosphine (393 mg, 1.5 mmol), 4Å MS (1 g), di-tert-butyl peroxide (292 mg, 2 mmol) in anhydrous dichloromethane (6 mL). The desired product was obtained as colorless oil (227 mg, 72% yield) after purification by flash column chromatography on silica gel (0.5% Et_3N in dichloromethane: methanol = 30:1). The dr value (5:1) was based on HPLC spectra.

^{1}H NMR (400 MHz, $CDCl_3$) δ 7.52 - 6.89 (m, 5H), 4.11 (q, J = 7.2 Hz, 2H), 2.90 - 2.23 (m, 8H), 2.21 - 1.58 (m, 9H), 1.58 - 1.35 (m, 2H), 1.23 (t, J = 7.2 Hz, 3H). ^{13}C NMR (101 MHz, $CDCl_3$) δ 174.1, 146.4, 128.4, 126.9, 126.2, 66.6, 60.3, 52.1, 37.3, 36.4, 34.8, 32.9, 31.6, 27.1, 24.0, 23.6, 23.3, 14.4. HRMS (ESI) calcd for $C_{20}H_{30}NO_2$ $[M + H]^+$ m/z = 316.2271, found: 316.2282. IR ν_{max}/cm^{-1} (film): 2929, 1731, 1451, 1369, 1283, 1155, 1031, 756, 699.

ethyl 2-(1-benzyl-4-(pyrrolidin-1-yl)piperidin-3-yl)acetate (51)

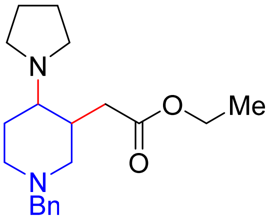

Prepared following the general procedure employing pyrrolidine (72 mg, 1 mmol), 1-benzylpiperidin-4-one (284 mg, 1.5 mmol), ethyl 2-mercaptoacetate (180 mg, 1.5 mmol), triphenylphosphine (393 mg, 1.5 mmol), 4Å MS (1 g), di-tert-butyl peroxide (292 mg, 2 mmol) in anhydrous dichloromethane (6 mL). The desired product was obtained as colorless oil (231 mg, 70% yield) after purification by flash column chromatography on silica gel (0.5% Et_3N in dichloromethane: methanol = 15:1). The dr value (7:1) was based on HPLC spectra.

^{1}H NMR (400 MHz, $CDCl_3$) δ 7.38 - 7.07 (m, 5H), 4.10 - 3.91 (m, 2H), 3.41 (dd, J = 53.9, 13.4 Hz, 2H), 2.88 - 2.60 (m, 4H), 2.60 - 2.38 (m, 4H), 2.38 - 2.26 (m, 1H), 2.07 - 1.87 (m, 3H), 1.85 - 1.69 (m, 4H), 1.69 - 1.49 (m, 2H), 1.17 (t, J = 7.1 Hz, 3H). ^{13}C NMR (101 MHz, $CDCl_3$) δ 174.6, 139.0, 128.8, 128.2, 126.9, 65.5, 62.7, 60.1, 56.0, 53.2, 51.9, 35.0, 30.8, 27.7, 23.3, 14.4. HRMS (ESI) calcd for $C_{20}H_{31}N_2O_2$ $[M + H]^+$ m/z = 331.2380, found: 331.2383. IR ν_{max}/cm^{-1} (film): 2945, 2774, 1729, 1452, 1367, 1289, 1167, 1123, 1035, 890, 739, 698.

ethyl 2-(1-benzyl-5-methyl-4-(pyrrolidin-1-yl)piperidin-3-yl)acetate (52)

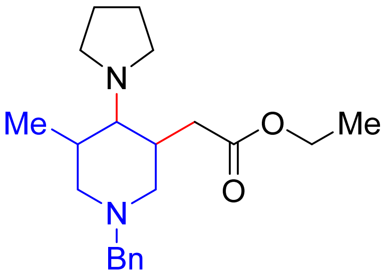

Prepared following the general procedure employing pyrrolidine (72 mg, 1 mmol), 1-benzyl-3-methylpiperidin-4-one (305 mg, 1.5 mmol), ethyl 2-mercaptoacetate (180 mg, 1.5 mmol), triphenylphosphine (393 mg, 1.5 mmol), 4Å MS (1 g), di-tert-butyl peroxide (292 mg, 2 mmol) in anhydrous dichloromethane (6 mL). The desired product was obtained as colorless oil (99 mg, 29% yield) after purification by flash column chromatography on silica gel (0.5% Et_3N in dichloromethane: methanol = 20:1).

^{1}H NMR (400 MHz, $CDCl_3$) δ 7.37 – 7.26 (m, 4H), 7.21 (t, J = 7.1 Hz, 1H), 4.04 (qd, J = 7.1, 1.6 Hz, 2H), 3.47 (d, J = 13.4 Hz, 1H), 3.42 (d, J = 13.5 Hz, 1H), 2.79 – 2.59 (m, 4H), 2.51 – 2.35 (m, 4H), 2.34 – 2.25 (m, 1H), 2.10 – 2.00 (m, 2H), 1.93 (t, J = 4.8 Hz, 1H), 1.89 – 1.82 (m, 1H), 1.82 – 1.67 (m, 4H), 1.19 (t, J = 7.1 Hz, 3H), 1.05 (d, J = 7.0 Hz, 3H). ^{13}C NMR (101 MHz, $CDCl_3$) δ 175.0, 139.6, 128.8, 128.2, 126.9, 67.2, 62.8, 60.1, 59.5, 57.2, 51.6, 35.1, 33.1, 32.8, 23.3, 14.8, 14.4. HRMS (ESI) calcd for $C_{21}H_{32}N_2O_2$ $[M + H]^+$ m/z = 345.2537, found: 345.2537. IR ν_{max}/cm^{-1} (film): 3059, 2964, 2930, 2774, 1728, 1437, 1378, 1303, 1274, 1250, 1198, 1178, 1069, 1027, 720, 696.

benzyl 5,5-dimethoxy-4-(pyrrolidin-1-yl)pentanoate (53)

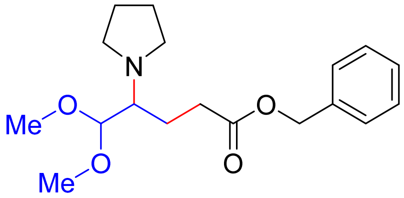

Prepared following the general procedure employing pyrrolidine (72 mg, 1 mmol), 1,1-dimethoxypropan-2-one (177 mg, 1.5 mmol), ethyl 2-mercaptoacetate (180 mg, 1.5 mmol), triphenylphosphine (393 mg, 1.5 mmol), 4Å MS (1 g), di-tert-butyl peroxide (292 mg, 2 mmol) in anhydrous dichloromethane (6 mL). The desired product was obtained as colorless oil (221 mg, 69% yield) after purification by flash column chromatography on silica gel (0.5% Et_3N in dichloromethane: methanol = 20:1).

^{1}H NMR (400 MHz, $CDCl_3$) δ 7.47 – 7.28 (m, 5H), 5.10 (s, 2H), 4.34 (d, J = 4.7 Hz, 1H), 3.39 (s, 3H), 3.37 (s, 3H), 2.78 – 2.56 (m, 5H), 2.51 (t, J = 7.6 Hz, 2H), 1.96 – 1.78 (m, 2H), 1.77 – 1.62 (m, 4H). δ 174.0, 136.3, 128.6, 128.2, 128.1, 106.8, 66.0, 61.2, 55.1, 49.6, 31.8, 23.7, 22.7. HRMS (ESI) calcd for $C_{18}H_{28}NO_4$ $[M + H]^+$ m/z = 322.2013, found: 322.2015. IR ν_{max}/cm^{-1} (film): 2957, 2830, 1734, 1454, 1380, 1162, 1122, 1073, 975, 735, 697.

ethyl 4-phenyl-4-(pyrrolidin-1-yl)butanoate (54)

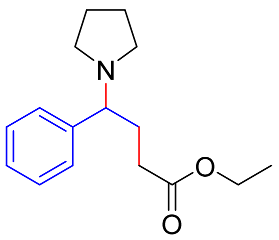

Prepared following the general procedure employing pyrrolidine (72 mg, 1 mmol)，acetophenone (180 mg, 1.5 mmol), ethyl 2-mercaptoacetate (180 mg, 1.5 mmol), triphenylphosphine (393 mg, 1.5 mmol), 4Å MS (1 g), di-tert-butyl peroxide (292 mg, 2 mmol) in anhydrous dichloromethane (6 mL). The desired product was obtained as a colorless oil (170 mg, 65% yield) after purification by flash column chromatography on silica gel (0.5% Et_3N in ethyl acetate).

^{1}H NMR (400 MHz, $CDCl_3$) δ 7.38 - 7.04 (m, 5H), 4.04 (q, J = 7.1 Hz, 2H), 3.08 (dd, J = 9.2, 4.1 Hz, 1H), 2.60 - 2.47 (m, 2H), 2.42 - 2.31 (m, 2H), 2.31 - 2.21 (m, 1H), 2.11 - 1.94 (m, 3H), 1.79 - 1.61 (m, 4H), 1.18 (t, J = 7.1 Hz, 3H). ^{13}C NMR (101 MHz, $CDCl_3$) δ 173.7, 142.2, 128.4, 128.27, 127.3, 70.2, 60.4, 52.8, 31.0, 30.8, 23.4, 14.3. HRMS (ESI) calcd for $C_{16}H_{24}NO_2$ [M + H]$^+$ m/z = 262.1802, found: 262.1806. IR ν_{max}/cm^{-1} (film): 2966, 2782, 1734, 1451, 1370, 1159, 1028, 763, 702.

ethyl 4-(pyrrolidin-1-yl)-4-(p-tolyl)butanoate (55)

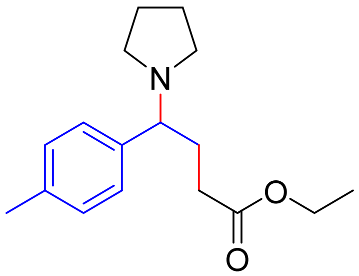

Prepared following the general procedure employing pyrrolidine (72 mg, 1 mmol), 1-(p-tolyl)ethan-1-one (201 mg, 1.5 mmol), ethyl 2-mercaptoacetate (180 mg, 1.5 mmol), triphenylphosphine (393 mg, 1.5 mmol), 4Å MS (1 g), di-tert-butyl peroxide (292 mg, 2 mmol) in anhydrous dichloromethane (6 mL). The desired product was obtained as colorless oil (170 mg, 62% yield) after purification by flash column chromatography on silica gel (0.5% Et_3N in ethyl acetate).

^{1}H NMR (400 MHz, $CDCl_3$) δ 7.20 (d, J = 8.0 Hz, 2H), 7.11 (d, J = 7.8 Hz, 2H), 4.04 (q, J = 7.1 Hz, 2H), 3.32 – 3.17 (m, 1H), 2.80 – 2.59 (m, 2H), 2.60 – 2.41 (m, 2H), 2.31 (s, 3H), 2.22 – 1.88 (m, 4H), 1.87 – 1.69 (m, 4H), 1.18 (t, J = 7.1 Hz, 3H). ^{13}C NMR (101 MHz, $CDCl_3$) δ 173.5, 137.6, 129.4, 128.6, 128.3, 70.0, 60.4, 52.9, 30.8, 30.2, 23.4, 21.2, 14.3. HRMS (ESI) calcd for $C_{17}H_{26}NO_2$ [M + H]$^+$ m/z = 276.1958, found: 276.1960. IR ν_{max}/cm^{-1} (film): 2959, 1734, 1596, 1443, 1258, 1169, 1069, 1022, 911, 814, 664.

ethyl 4-(3-fluorophenyl)-4-(pyrrolidin-1-yl) butanoate (56)

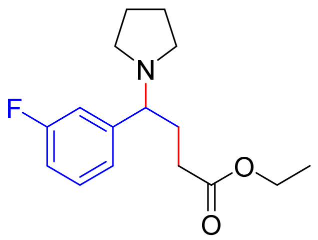

Prepared following the general procedure employing pyrrolidine (72 mg, 1 mmol)，1-(3-fluorophenyl)ethan-1-one (207 mg, 1.5 mmol), ethyl 2-mercaptoacetate (180 mg, 1.5 mmol), triphenylphosphine (393 mg, 1.5 mmol), 4Å MS (1 g), di-tert-butyl peroxide (292 mg, 2 mmol) in anhydrous dichloromethane (6 mL). The desired product was obtained as colorless oil (206 mg, 74% yield) after purification by flash column chromatography on silica gel (0.5% Et_3N in ethyl acetate).

^{1}H NMR (400 MHz, $CDCl_3$) δ 7.27 - 7.20 (m, 1H), 7.08 - 6.96 (m, 2H), 6.96 - 6.86 (m, 1H), 4.04 (q, J = 7.1 Hz, 2H), 3.12 (dd, J = 9.2, 3.8 Hz, 1H), 2.63 - 2.46 (m, 2H), 2.45 - 2.29 (m, 2H), 2.30 - 2.16 (m, 1H), 2.14 - 1.89 (m, 3H), 1.81 - 1.64 (m, 4H), 1.19 (t, J = 7.1 Hz, 3H). ^{13}C NMR (101 MHz, $CDCl_3$) δ 173.5, 163.06 (d, J = 245.8 Hz), 144.92 (d, J = 6.0 Hz), 129.86 (d, J = 8.2 Hz), 123.91 (d, J = 2.8 Hz), 114.98 (d, J = 21.3 Hz), 114.32 (d, J = 21.2 Hz), 69.7, 60.5, 52.7, 30.7, 30.7, 23.4, 14.3. ^{19}F NMR (376 MHz, $CDCl_3$) δ -113.4 (s). HRMS (ESI) calcd for $C_{16}H_{23}FNO_2$ [M + H]$^+$ m/z = 280.1707, found: 280.1707. IR ν_{max}/cm^{-1} (film): 2965, 1734, 1588, 1483, 1448, 1369, 1248, 1158, 1029, 786, 701.

ethyl 4-(4-chlorophenyl)-4-(pyrrolidin-1-yl)butanoate (57)

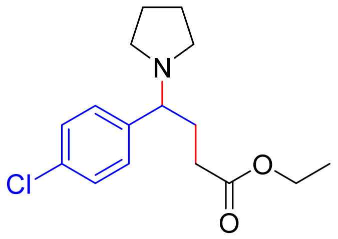

Prepared following the general procedure employing pyrrolidine (72 mg, 1 mmol), 1-(4-chlorophenyl) ethan-1-one (231 mg, 1.5 mmol), ethyl 2-mercaptoacetate (180 mg, 1.5 mmol), triphenylphosphine (393 mg, 1.5 mmol), 4Å MS (1 g), di-tert-butyl peroxide (292 mg, 2 mmol) in anhydrous dichloromethane (6 mL). The desired product was obtained as colorless oil (198 mg, 67% yield) after purification by flash column chromatography on silica gel (0.5% Et_3N in ethyl acetate).

^{1}H NMR (400 MHz, $CDCl_3$) δ 7.26 (d, J = 8.6 Hz, 2H), 7.20 (d, J = 8.5 Hz, 2H), 4.03 (q, J = 7.1 Hz, 2H), 3.08 (dd, J = 9.3, 4.0 Hz, 1H), 2.63 - 2.41 (m, 2H), 2.41 - 2.29 (m, 2H), 2.29 - 2.18 (m, 1H), 2.12 - 1.85 (m, 3H), 1.80 - 1.61 (m, 4H), 1.18 (t, J = 7.1 Hz, 3H). ^{13}C NMR (101 MHz, $CDCl_3$) δ 173.5, 140.8, 133.0, 129.6, 128.6, 69.4, 60.4, 52.7, 30.8, 30.8, 23.4, 14.3. HRMS (ESI) calcd for $C_{16}H_{23}ClNO_2$ [M + H]$^+$ m/z = 296.1412, found: 296.1414. IR ν_{max}/cm^{-1} (film): 2967, 2788, 1734, 1478, 1370, 1159, 1089, 1014, 828.

ethyl 4-(4-bromophenyl)-4-(pyrrolidin-1-yl) butanoate (58)

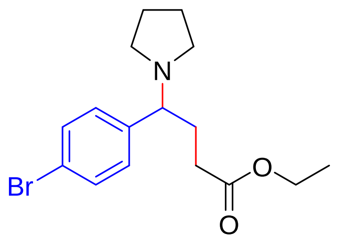

Prepared following the general procedure employing pyrrolidine (72 mg, 1 mmol), 1-(4-bromophenyl) ethan-1-one (297 mg, 1.5 mmol), ethyl 2-mercaptoacetate (180 mg, 1.5 mmol), triphenylphosphine (393 mg, 1.5 mmol), 4Å MS (1 g), di-tert-butyl peroxide (292 mg, 2 mmol) in anhydrous dichloromethane (6 mL). The desired product was obtained as colorless oil (227 mg, 67% yield) after purification by flash column chromatography on silica gel (0.5% Et_3N in ethyl acetate).

^{1}H NMR (400 MHz, $CDCl_3$) δ 7.42 (d, J = 8.0 Hz, 2H), 7.16 (d, J = 8.0 Hz, 2H), 4.04 (q, J = 7.1 Hz, 2H), 3.16 - 3.02 (m, 1H), 2.70 - 2.47 (m, 2H), 2.47 - 2.31 (m, 2H), 2.26 - 1.92 (m, 4H), 1.81 - 1.58 (m, 4H), 1.19 (t, J = 7.1 Hz, 3H). ^{13}C NMR (101 MHz, $CDCl_3$) δ 173.4, 140.9, 131.6, 130.0, 121.2, 69.5, 60.5, 53.0, 52.8, 30.7, 30.6, 23.5, 23.4, 14.3. HRMS (ESI) calcd for $C_{16}H_{23}BrNO_2$ [M + H]$^+$ m/z = 340.0907, found: 340.0907. IR ν_{max}/cm^{-1} (film): 2966, 1734, 1484, 1369, 1179, 1070, 1009, 824, 722.

ethyl 4-(pyrrolidin-1-yl)-4-(4-(trifluoromethyl)phenyl)butanoate (59)

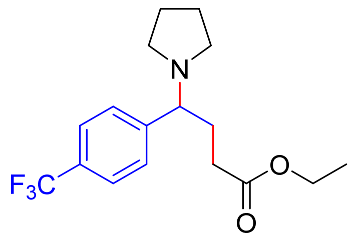

Prepared following the general procedure employing pyrrolidine (72 mg, 1 mmol), 1-(4-(trifluoromethyl) phenyl) ethan-1-one (282 mg, 1.5 mmol), ethyl 2-mercaptoacetate (180 mg, 1.5 mmol), triphenylphosphine (393 mg, 1.5 mmol), 4Å MS (1 g), di-tert-butyl peroxide (292 mg, 2 mmol) in anhydrous dichloromethane (6 mL). The desired product was obtained as colorless oil (250 mg, 76% yield) after purification by flash column chromatography on silica gel (0.5% Et_3N in ethyl acetate).

^{1}H NMR (400 MHz, $CDCl_3$) δ 7.54 (d, J = 8.0 Hz, 2H), 7.39 (d, J = 8.1 Hz, 2H), 4.03 (q, J = 7.1 Hz, 2H), 3.17 (dd, J = 8.5, 4.1 Hz, 1H), 2.56 - 2.44 (m, 2H), 2.42 - 2.30 (m, 2H), 2.29 - 2.20 (m, 1H), 2.11 - 1.91 (m, 3H), 1.77 - 1.64 (m, 4H), 1.18 (t, J = 7.1 Hz, 3H). ^{13}C NMR (101 MHz, $CDCl_3$) δ 173.4, 146.6, 129.6 (d, J = 32.3 Hz), 128.6, 125.4 (q, J = 3.8 Hz), 123.0, 69.7, 60.5, 52.7, 30.8, 30.6, 23.4, 14.3. ^{19}F NMR (376 MHz, $CDCl_3$) δ -62.5 (s). HRMS (ESI) calcd for $C_{17}H_{23}F_3NO_2$ [M + H]$^+$ m/z = 330.1675, found: 330.1669. IR ν_{max}/cm^{-1} (film): 2969, 2791, 1735, 1418, 1325, 1163, 1125, 1067, 1017, 842.

ethyl 4-(pyridin-3-yl)-4-(pyrrolidin-1-yl)butanoate (60)

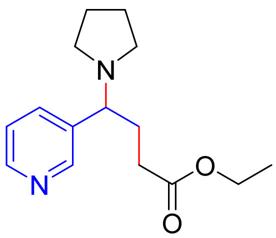

Prepared following the general procedure employing pyrrolidine (72 mg, 1 mmol), 1-(pyridin-3-yl) ethan-1-one (182 mg, 1.5 mmol), ethyl 2-mercaptoacetate (180 mg, 1.5 mmol), triphenylphosphine (393 mg, 1.5 mmol), 4Å MS (1 g), di-tert-butyl peroxide (292 mg, 2 mmol) in anhydrous dichloromethane (6 mL). The desired product was obtained as yellow oil (188 mg, 72% yield) after purification by flash column chromatography on silica gel (0.5% Et_3N in dichloromethane: methanol = 20:1).

^{1}H NMR (400 MHz, $CDCl_3$) δ 8.48 - 8.30 (m, 2H), 7.59 (dt, J = 7.9, 1.9 Hz, 1H), 7.18 (ddd, J = 7.8, 4.8, 0.7 Hz, 1H), 3.97 (q, J = 7.1 Hz, 2H), 3.13 (dd, J = 8.8, 4.2 Hz, 1H), 2.55 - 2.39 (m, 2H), 2.35 - 2.26 (m, 2H), 2.26 - 2.15 (m, 1H), 2.09 - 1.88 (m, 3H), 1.74 - 1.55 (m, 4H), 1.12 (t, J = 7.1 Hz, 3H). ^{13}C NMR (101 MHz, $CDCl_3$) δ 173.2, 149.8, 148.9, 137.4, 135.5, 123.5, 67.2, 60.4, 52.5, 30.5, 23.2, 14.2. HRMS (ESI) calcd for $C_{15}H_{23}N_2O_2$ [M + H]$^+$ m/z = 263.1754, found: 263.1755. IR ν_{max}/cm^{-1} (film): 2968, 2788, 1733, 1576, 1424, 1370, 1319, 1180, 1025, 808, 718.

ethyl 4-(naphthalen-2-yl)-4-(pyrrolidin-1-yl)butanoate (61)

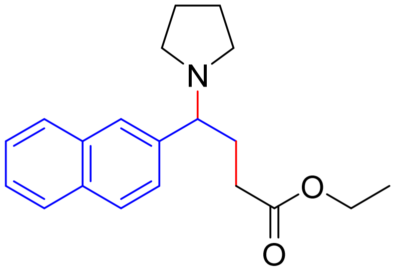

Prepared following the general procedure employing pyrrolidine (72 mg, 1 mmol), 1-(naphthalen-2-yl) ethan-1-one (255 mg, 1.5 mmol), ethyl 2-mercaptoacetate (180 mg, 1.5 mmol), triphenylphosphine (393 mg, 1.5 mmol), 4Å MS (1 g), di-tert-butyl peroxide (292 mg, 2 mmol) in anhydrous dichloromethane (6 mL). The desired product was obtained as colorless oil (180 mg, 58% yield) after purification by flash column chromatography on silica gel (0.5% Et_3N in ethyl acetate).

^{1}H NMR (400 MHz, $CDCl_3$) δ 7.80 (t, J = 7.5 Hz, 2H), 7.75 – 7.57 (m, 2H), 7.58 – 7.50 (m, 1H), 7.49 – 7.42 (m, 2H), 4.01 (q, J = 7.1 Hz, 2H), 3.53 – 3.40 (m, 1H), 2.83 – 2.63 (m, 2H), 2.56 – 2.44 (m, 2H), 2.43 – 2.31 (m, 1H), 2.11 – 1.98 (m, 2H), 1.97 – 1.88 (m, 1H), 1.87 – 1.71 (m, 4H), 1.15 (t, J = 7.1 Hz, 3H). ^{13}C NMR (101 MHz, $CDCl_3$) δ 173.5, 133.3, 133.2, 132.3, 132.2, 128.7, 128.6, 128.0, 127.8, 126.2, 126.0, 70.5, 60.4, 53.1, 30.9, 29.6, 23.5, 14.3. HRMS (ESI) calcd for $C_{20}H_{26}NO_2$ [M + H]$^+$ m/z = 312.1958, found: 312.1961. IR ν_{max}/cm^{-1} (film): 2965, 1731, 1437, 1197, 1118, 1027, 747, 720, 695.

ethyl 4-(9H-fluoren-3-yl)-4-(pyrrolidin-1-yl)butanoate (62)

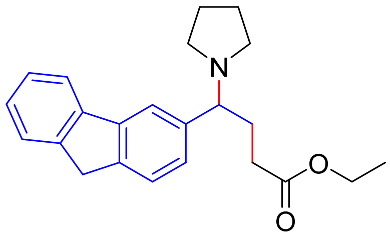

Prepared following the general procedure employing pyrrolidine (72 mg, 1 mmol), 1-(9H-fluoren-3-yl) ethan-1-one (312 mg, 1.5 mmol), ethyl 2-mercaptoacetate (180 mg, 1.5 mmol), triphenylphosphine (393 mg, 1.5 mmol), 4Å MS (1 g), di-tert-butyl peroxide (292 mg, 2 mmol) in anhydrous dichloromethane (6 mL). The desired product was obtained as colorless oil (143 mg, 41% yield) after purification by flash column chromatography on silica gel (0.5% Et_3N in dichloromethane: methanol = 20:1).

^{1}H NMR (400 MHz, $CDCl_3$) δ 7.72 (dd, J = 12.8, 7.7 Hz, 2H), 7.57 - 7.47 (m, 2H), 7.40 - 7.25 (m, 3H), 4.03 (q, J = 7.1 Hz, 2H), 3.87 (s, 2H), 3.47 (t, J = 6.8 Hz, 1H), 2.80 - 2.66 (m, 2H), 2.59 - 2.50 (m, 2H), 2.42 - 2.29 (m, 2H), 2.20 - 2.09 (m, 1H), 1.94 - 1.87 (m, 1H), 1.85 - 1.74 (m, 4H), 1.17 (t, J = 7.1 Hz, 3H). ^{13}C NMR (101 MHz, $CDCl_3$) δ 173.5, 143.8, 143.5, 141.6, 141.4, 127.3, 126.9, 125.2, 124.8, 119.9, 119.9, 70.5, 60.4, 53.0, 37.0, 30.9, 30.9, 23.4, 14.3. HRMS (ESI) calcd for $C_{23}H_{28}NO_2$ $[M + H]^+$ m/z = 350.2115, found: 350.2117. IR ν_{max}/cm^{-1} (film): 2965, 1732, 1640, 1436, 1372, 1268, 1176, 1028, 771, 738.

benzyl 3-benzyl-4-(benzyl(methyl)amino)butanoate (63)

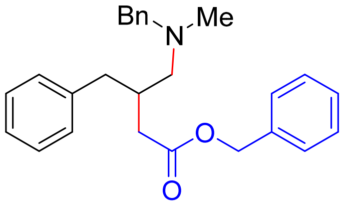

Prepared following the general procedure employing N-methyl-1-phenylmethanamine (121mg, 1 mmol), 3-phenylpropanal (201 mg, 1.5 mmol), benzyl 2-mercaptoacetate (273 mg, 1.5 mmol), triphenylphosphine (393 mg, 1.5 mmol), 4Å MS (1 g), di-tert-butyl peroxide (292 mg, 2 mmol) in anhydrous dichloromethane (6 mL). The desired product was obtained as colorless oil (352 mg, 91% yield) after purification by flash column chromatography on silica gel (0.5% Et_3N in dichloromethane).

^{1}H NMR (400 MHz, $CDCl_3$) δ 7.55 - 6.93 (m, 15H), 5.08 (s, 2H), 3.48 (s, 2H), 2.79 (dd, J = 10.6, 5.2 Hz, 1H), 2.61 - 2.42 (m, 3H), 2.35 - 2.25 (m, 3H), 2.16 (s, 3H). ^{13}C NMR (101 MHz, $CDCl_3$) δ 173.1, 134.0, 139.4, 136.2, 129.4, 129.1, 128.6, 128.4, 128.3, 128.2, 127.0, 126.1, 66.1, 62.9, 61.5, 42.5, 38.9, 37.3, 35.6. HRMS (ESI) calcd for $C_{26}H_{30}NO_2$ $[M + H]^+$ m/z = 388.2271, found: 388.2277. IR ν_{max}/cm^{-1} (film): 3027, 2928, 1734, 1495, 1453, 1256, 1148, 1025, 740, 698.

benzyl 3-benzyl-4-(benzyl(methyl)amino)butanoate (derived from disulfide) **(63)**

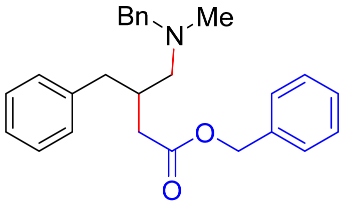

Prepared following the general procedure employing N-methyl-1-phenylmethanamine (121mg, 1 mmol), 3-phenylpropanal (201 mg, 1.5 mmol), dibenzyl 2, 2'-disulfanediyldiacetate (273 mg, 0.75 mmol), triphenylphosphine (393 mg, 1.5 mmol), 4Å MS (1 g), di-tert-butyl peroxide (292 mg, 2 mmol) in anhydrous dichloromethane (6 mL). The desired product was obtained as colorless oil (305 mg, 79% yield) after purification by flash column chromatography on silica gel (0.5% Et_3N in dichloromethane).

^{1}H NMR (400 MHz, $CDCl_3$) δ 7.72 - 6.98 (m, 15H), 5.12 (s, 2H), 3.50 (s, 2H), 2.82 (dd, J = 12.7, 5.0 Hz, 1H), 2.65 - 2.44 (m, 3H), 2.41 - 2.23 (m, 3H), 2.20 (s, 3H). ^{13}C NMR (101 MHz, $CDCl_3$) δ 173.1, 140.0, 139.4, 136.2, 129.4, 129.0, 128.6, 128.4, 128.3, 128.2, 128.2, 126.9, 126.1, 66.1, 62.8, 61.5, 42.5, 38.9, 37.3, 35.6. HRMS (ESI) calcd for $C_{26}H_{29}NO_2$ [M + H]$^+$ m/z = 388.2271, found: 388.2276. IR ν_{max}/cm^{-1} (film): 3027, 2943, 1732, 1495, 1453, 1380, 1256, 1211, 1148, 1025, 740, 698.

ethyl 3-benzyl-4-(benzyl(methyl)amino)-2-methylbutanoate (64)

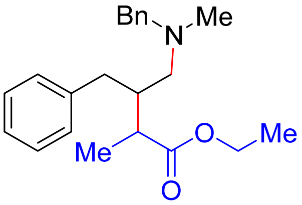

Prepared following the general procedure employing N-methyl-1-phenylmethanamine (121mg, 1 mmol), 3-phenylpropanal (201 mg, 1.5 mmol), ethyl 2-mercaptopropanoate (201 mg, 1.5 mmol), triphenylphosphine (393 mg, 1.5 mmol), 4Å MS (1 g), di-tert-butyl peroxide (292 mg, 2 mmol) in anhydrous dichloromethane (6 mL). The desired product was obtained as colorless oil (258 mg, 76% yield) after purification by flash column chromatography on silica gel (0.5% Et_3N in dichloromethane). The dr value (1:1) was determined by ^{1}H NMR spectra.

^{1}H NMR (400 MHz, $CDCl_3$) δ 7.47 - 6.93 (m, 10H), 4.05 (q, J = 7.1 Hz, 2H), 3.52 (dd, J = 26.0, 12.4 Hz, 1H), 3.33 (dd, J = 13.1, 8.9 Hz, 1H), 2.93 - 2.12 (m, 6H), 2.10 (s, 3H), 1.22 (dt, J = 9.3, 7.2 Hz, 3H), 1.08 (d, J = 7.1 Hz, 1.5H, diastereomer A), 1.02 (d, J = 7.1 Hz, 1.5H, diastereomer B). ^{13}C NMR (101 MHz, $CDCl_3$, double carbon signals) δ 176.5, 175.9, 140.9, 140.9, 129.3, 129.2, 129.2, 128.7, 128.6, 128.5, 128.4, 128.3, 128.3, 128.2, 127.0, 127.0, 126.0, 126.0, 63.1, 62.7, 60.2, 60.2, 58.9, 58.4, 42.5, 42.5, 40.7, 40.6, 39.5, 36.6, 35.5, 34.6, 14.4, 14.4, 11.4, 11.4. HRMS (ESI) calcd for $C_{22}H_{30}NO_2$ [M + H]$^+$ m/z = 340.2271, found: 340.2273. IR ν_{max}/cm^{-1} (film): 3026, 2978, 2932, 1728, 1494, 1453, 1367, 1183, 1025, 740, 699.

benzyl 3-benzyl-4-(benzyl(methyl)amino)-2-methylbutanoate (65)

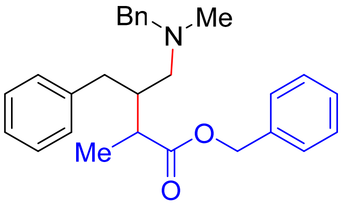

Prepared following the general procedure employing N-methyl-1-phenylmethanamine (121mg, 1 mmol), 3-phenylpropanal (201 mg, 1.5 mmol), benzyl 2-mercaptopropanoate (294 mg, 1.5 mmol), triphenylphosphine (393 mg, 1.5 mmol), 4Å MS (1 g), di-tert-butyl peroxide (292 mg, 2 mmol) in anhydrous dichloromethane (6 mL). The desired product was obtained as colorless oil (257 mg,

64% yield) after purification by flash column chromatography on silica gel (0.5% Et_3N in dichloromethane). The dr value (1:1) was determined by ^{1}H NMR spectra.

^{1}H NMR (400 MHz, $CDCl_3$) δ 7.41 - 7.05 (m, 15H), 5.22 - 4.89 (m, 2H), 3.55 - 3.25 (m, 2H), 3.05 - 2.70 (m, 2H), 2.69 - 2.14 (m, 4H), 2.09 (s, 3H), 1.14 (d, J = 7.1 Hz, 1.5H, diastereomer A), 1.07 (d, J = 7.1 Hz, 1.5H, diastereomer B). ^{13}C NMR (101 MHz, $CDCl_3$, double carbon signals) δ 176.3, 175.7, 140.7, 139.5, 139.4, 136.4, 136.4, 129.3, 129.2, 129.1, 128.6, 128.6, 128.6, 128.4, 128.4, 128.3, 128.3, 128.2, 128.2, 128.1, 127.7, 127.1, 127.0, 126.0, 125.9, 66.1, 66.1, 63.0, 62.6, 58.8, 58.7, 58.3, 42.5, 42.4, 40.7, 40.7, 39.6, 39.5, 36.6, 35.4, 11.4, 11.3. HRMS (ESI) calcd for $C_{27}H_{32}NO_2$ $[M + H]^+$ m/z = 402.2428, found: 402.2430. IR ν_{max}/cm^{-1} (film): 3027, 2944, 1730, 1495, 1453, 1170, 1027, 740, 698.

dibenzyl 2-(1-(benzyl(methyl)amino)-3-phenylpropan-2-yl) succinate (66)

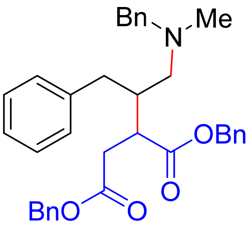

Prepared following the general procedure employing N-methyl-1-phenylmethanamine (121mg, 1 mmol), 3-phenylpropanal (201 mg, 1.5 mmol), dibenzyl 2-mercaptosuccinate (495 mg, 1.5 mmol), triphenylphosphine (393 mg, 1.5 mmol), 4Å MS (1 g), di-tert-butyl peroxide (292 mg, 2 mmol) in anhydrous dichloromethane (6 mL). The desired product was obtained as colorless oil (417 mg, 78% yield) after purification by flash column chromatography on silica gel (0.5% Et_3N in petroleum ether: ethyl acetate = 3:1). The dr value (1:1) was determined by ^{1}H NMR spectra.

^{1}H NMR (400 MHz, $CDCl_3$) δ 7.68 - 6.90 (m, 20H), 5.31 - 5.01 (m, 4H), 3.64 (dd, J = 16.0, 12.5 Hz, 1H), 3.50 - 3.28 (m, 2H), 2.97 - 2.07 (m, 7H), 2.23 (s, 1.5H, diastereomer A), 2.11 (s, 1.5H, diastereomer B). ^{13}C NMR (101 MHz, $CDCl_3$, double carbon signals) δ 174.4, 173.6, 172.1, 172.0, 141.4, 141.2, 139.9, 139.1, 139.0, 136.0, 135.9, 135.9, 129.1, 129.0, 128.9, 128.6, 128.6, 128.5, 128.4, 128.4, 128.4, 128.3, 128.3, 128.2, 128.2, 128.1, 128.1, 128.1, 127.4, 127.0, 126.9, 126.8, 126.5, 126.3, 126.1, 126.0, 66.3, 66.3, 66.3, 60.3, 62.8, 62.6, 58.8, 57.4, 42.4, 42.3, 41.8, 41.2, 40.0, 39.8, 36.5, 35.8, 31.9, 30.9. HRMS (ESI) calcd for $C_{35}H_{38}NO_4$ $[M + H]^+$ m/z = 536.2795, found: 536.2795. IR ν_{max}/cm^{-1} (film): 3062, 3029, 2945, 1732, 1496, 1454, 1387, 1259, 1213, 1159, 1002, 744, 699.

3-(1-(benzyl(methyl)amino)-3-phenylpropan-2-yl) dihydrofuran-2(3H)-one (67)

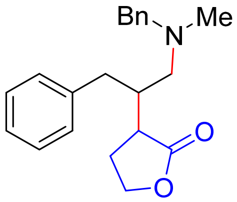

Prepared following the general procedure employing N-methyl-1-phenylmethanamine (121mg, 1 mmol), 3-phenylpropanal (201 mg, 1.5 mmol), 3-mercaptodihydrofuran-2(3H)-one (177 mg, 1.5 mmol), triphenylphosphine (393 mg, 1.5 mmol), 4Å MS (1 g), di-tert-butyl peroxide (292 mg, 2 mmol) in anhydrous dichloromethane (6 mL). The desired product was obtained as a colorless oil

(216 mg, 67% yield) after purification by flash column chromatography on silica gel (0.5% Et_3N in petroleum ether: ethyl acetate = 4:1). The dr value (1:1) was determined by ^1H NMR spectra.

^1H NMR (400 MHz, $CDCl_3$) δ 7.43 - 7.02 (m, 10H), 4.31 - 4.02 (m, 2H), 3.59 - 3.05 (m, 3H), 2.93 - 2.68 (m, 1H), 2.67 - 2.57 (m, 1H), 2.55 - 2.24 (m, 3H), 2.18 (s, 1.5H, diastereomer A), 2.12 (s, 1.5H, diastereomer B), 1.94 - 1.81 (m, 2H). ^{13}C NMR (101 MHz, $CDCl_3$, double carbon signals) δ 179.8, 179.2, 140.0, 139.8, 139.3, 139.2, 129.3, 129.3, 129.2, 129.1, 128.7, 128.5, 128.5, 128.3, 127.2, 127.1, 126.5, 126.3, 66.6, 66.5, 63.4, 62.7, 58.8, 57.8, 42.9, 42.8, 40.8, 40.1, 38.2, 38.0, 37.8, 35.7, 23.5, 22.6. HRMS (ESI) calcd for $C_{21}H_{26}NO_2$ $[M + H]^+$ m/z = 324.1958, found: 324.1959. IR ν_{max}/cm^{-1} (film): 2927, 1766, 1494, 1452, 1372, 1161, 1026, 741, 699.

3-benzyl-4-(benzyl(methyl)amino)-N,N-diethylbutanamide (68)

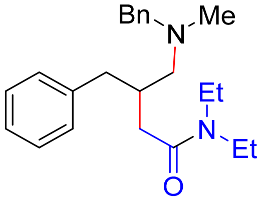

Prepared following the general procedure employing N-methyl-1-phenylmethanamine (121mg, 1 mmol), 3-phenylpropanal (201 mg, 1.5 mmol), N, N-diethyl-2-mercaptoacetamide (220 mg, 1.5 mmol), triphenylphosphine (393 mg, 1.5 mmol), 4Å MS (1 g), di-tert-butyl peroxide (292 mg, 2 mmol) in anhydrous dichloromethane (6 mL). The desired product was obtained as colorless oil (229 mg, 65% yield) after purification by flash column chromatography on silica gel (0.5% Et_3N in petroleum ether: ethyl acetate = 1:1).

^1H NMR (400 MHz, $CDCl_3$) δ 7.47 - 6.98 (m, 10H), 3.57 (br, 2H), 3.32 (dq, J = 14.1, 7.0 Hz, 2H), 3.17 (dq, J = 14.1, 7.0 Hz, 2H), 2.77 (dd, J = 13.4, 6.4 Hz, 1H), 2.66 (dd, J = 13.4, 6.4 Hz, 1H), 2.59 - 2.50 (m, 1H), 2.46 - 2.03 (m, 7H), 1.06 (dt, J = 10.0, 7.1 Hz, 6H). ^{13}C NMR (101 MHz, $CDCl_3$) δ 171.5, 131.6, 129.6, 128.4, 128.4, 126.1, 62.4, 61.0, 42.4, 42.0, 40.4, 38.9, 35.3, 35.2, 14.4, 13.3. HRMS (ESI) calcd for $C_{23}H_{33}N_2O$ $[M + H]^+$ m/z = 353.2587, found: 353.2589. IR ν_{max}/cm^{-1} (film): 2929, 1640, 1494, 1452, 1257, 1130, 1073, 1025, 740, 699.

1-((3R,5R)-adamantan-1-yl)-3-benzyl-4-(benzyl(methyl)amino)butan-1-one (69)

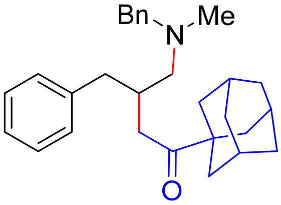

Prepared following the general procedure employing N-methyl-1-phenylmethanamine (121mg, 1 mmol), 3-phenylpropanal (201 mg, 1.5 mmol), 1-((3R,5R)-adamantan-1-yl)-2-mercaptoethan-1-one (315 mg, 1.5 mmol), triphenylphosphine (393 mg, 1.5 mmol), 4Å MS (1 g), di-tert-butyl peroxide (292 mg, 2 mmol) in anhydrous dichloromethane (6 mL). The desired product was obtained as colorless oil (365 mg, 88% yield) after purification by flash column chromatography on silica gel (0.5% Et_3N in dichloromethane).

^1H NMR (400 MHz, $CDCl_3$) δ 7.52 - 6.91 (m, 10H), 3.44 (s, 2H), 2.74 - 2.63 (m, 1H), 2.62 - 2.46 (m, 3H), 2.34 - 2.15 (m, 3H), 2.13 (s, 3H), 2.01 (s, 3H), 1.86 - 1.52 (m, 12H). ^{13}C NMR (101 MHz,

$CDCl_3$) δ 215.4, 140.6, 139.5, 129.5, 129.2, 128.3, 128.2, 126.9, 125.9, 62.5, 61.4, 46.5, 42.7, 38.9, 38.7, 38.4, 36.8, 33.5, 28.2. HRMS (ESI) calcd for $C_{29}H_{38}NO$ [M + H]$^+$ m/z = 416.2948, found: 416.2947. IR ν_{max}/cm^{-1} (film): 3025, 2904, 2849, 2790, 1696, 1494, 1452, 1364, 1024, 739, 699.

4-benzyl-5-(benzyl(methyl)amino)-3-methylpentan-2-one (70)

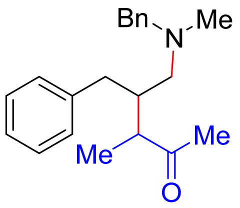

Prepared following the general procedure employing N-methyl-1-phenylmethanamine (121mg, 1 mmol), 3-phenylpropanal (201 mg, 1.5 mmol), 3-mercaptobutan-2-one (156 mg, 1.5 mmol), triphenylphosphine (393 mg, 1.5 mmol), 4Å MS (1 g), di-tert-butyl peroxide (292 mg, 2 mmol) in anhydrous dichloromethane (6 mL). The desired product was obtained as colorless oil (216 mg, 70% yield) after purification by flash column chromatography on silica gel (0.5% Et_3N in petroleum ether: ethyl acetate = 5:1). The dr value (1:1) was determined by ^1H NMR spectra.

^1H NMR (400 MHz, $CDCl_3$) δ 7.38 - 6.98 (m, 10H), 3.56 - 3.44 (m, 1H), 3.38 - 3.18 (m, 1H), 2.99 - 2.80 (m, 1H), 2.65 - 2.13 (m, 5H), 2.11 (s, 1.5H), 2.09 (s, 1.5H), 2.07 (s, 1.5H), 2.03 (s, 1.5H), 1.01 (d, J = 7.0 Hz, 1.5H, diastereomer A), 0.96 (d, J = 7.0 Hz, 1.5H, diastereomer B). ^{13}C NMR (101 MHz, $CDCl_3$, double carbon signals) δ 212.3, 211.5, 140.9, 140.8, 139.3, 139.2, 129.2, 129.2, 129.2, 129.1, 128.6, 128.6, 128.4, 128.3, 127.1, 127.0, 126.2, 126.1, 63.0, 62.6, 58.8, 58.4, 48.1, 47.1, 42.6, 42.6, 40.3, 39.9, 37.0, 35.2, 28.7, 28.6, 10.8, 10.1. HRMS (ESI) calcd for $C_{21}H_{28}NO$ [M + H]$^+$ m/z = 310.2165, found: 310.2165. IR ν_{max}/cm^{-1} (film): 3026, 2926, 2852, 2788, 1707, 1494, 1453, 1353, 1075, 1026, 741, 699.

3-benzyl-4-(benzyl(methyl)amino)-2-methyl-1-(p-tolyl)butan-1-one (71)

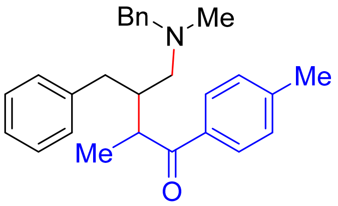

Prepared following the general procedure employing N-methyl-1-phenylmethanamine (121mg, 1 mmol), 3-phenylpropanal (201 mg, 1.5 mmol), 2-mercapto-1-(p-tolyl) propan-1-one (270 mg, 1.5 mmol), triphenylphosphine (393 mg, 1.5 mmol), 4Å MS (1 g), di-tert-butyl peroxide (292 mg, 2 mmol) in anhydrous dichloromethane (6 mL). The desired product was obtained as colorless oil (301 mg, 78% yield) after purification by flash column chromatography on silica gel (0.5% Et_3N in petroleum ether: ethyl acetate = 5:1). The dr value (1:1) was determined by ^1H NMR spectra.

^1H NMR (400 MHz, $CDCl_3$) δ 7.89 (d, J = 8.2 Hz, 1H), 7.42 (d, J = 8.2 Hz, 1H), 7.37 - 7.04 (m, 11H), 6.98 (d, J = 6.9 Hz, 1H), 3.92 (qd, J = 6.8, 3.1 Hz, 0.5H), 3.59 - 3.39 (m, 1.5H), 3.25 (t, J = 13.4 Hz, 1H), 3.07 (dd, J = 13.5, 4.6 Hz, 0.5H), 2.79 - 2.63 (m, 1H), 2.52 (dd, J = 13.4, 9.0 Hz, 0.5H), 2.41 (s, 1.5H), 2.35 (s, 1.5H), 2.32 - 2.07 (m, 3H), 2.06 (s, 1.5H), 1.95 (s, 1.5H), 1.13 (d, J = 6.8 Hz, 1.5H, diastereomer A), 1.01 (d, J = 6.8 Hz, 1.5H, diastereomer B). ^{13}C NMR (101 MHz, $CDCl_3$, double carbon signals) δ 203.9, 203.4, 143.2, 143.2, 141.1, 140.9, 134.8, 134.4, 129.5, 129.3, 129.2, 129.2, 129.2, 129.1, 128.7, 128.6, 128.6, 128.5, 128.5, 128.5, 128.3, 128.2, 127.0,

126.9, 126.2, 125.9, 62.7, 62.6, 58.5, 58.1, 42.7, 41.6, 41.3, 40.7, 40.4, 36.9, 34.6, 34.6, 21.7, 21.6, 10.9, 10.3. HRMS (ESI) calcd for $C_{27}H_{32}NO$ $[M + H]^+$ m/z = 386.2478, found: 386.2478. IR ν_{max}/cm^{-1} (film): 3025, 2941, 1676, 1605, 1452, 1255, 1181, 1026, 971, 741, 699.

ethane-1,2-diyl bis(3-benzyl-4-(benzyl(methyl)amino)butanoate) (72)

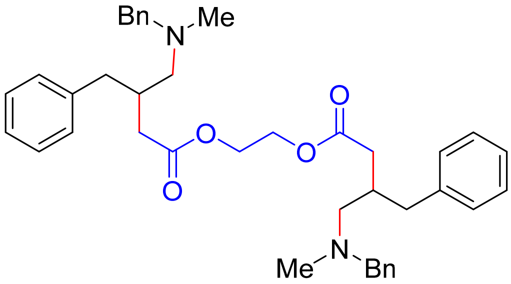

Prepared following the general procedure employing N-methyl-1-phenylmethanamine (242 mg, 2 mmol), 3-phenylpropanal (402 mg, 3 mmol), ethane-1,2-diyl bis(2-mercaptoacetate) (105 mg, 0.5 mmol), triphenylphosphine (393 mg, 1.5 mmol), 4Å MS (1 g), di-tert-butyl peroxide (292 mg, 2 mmol) in anhydrous dichloromethane (6 mL). The desired product was obtained as colorless oil (446 mg, 72% yield) after purification by flash column chromatography on silica gel (0.5% Et_3N in petroleum ether: ethyl acetate = 3:1).

^{1}H NMR (400 MHz, $CDCl_3$) δ 7.51 - 7.04 (m, 20H), 4.24 (s, 4H), 3.53 (dd, J = 15.7, 13.1 Hz, 4H), 2.88 - 2.72 (m, 2H), 2.71 - 2.26 (m, 12H), 2.23 (s, 6H). ^{13}C NMR (101 MHz, $CDCl_3$) δ 172.9, 139.8, 139.2, 129.3, 129.0, 128.2, 128.1, 126.9, 126.0, 62.8, 61.9, 61.4, 42.4, 38.8, 37.1, 35.4. HRMS (ESI) calcd for $C_{40}H_{49}N_2O_4$ $[M + H]^+$ m/z = 621.3687, found: 621.3688. IR ν_{max}/cm^{-1} (film): 3026, 2946, 2790, 1736, 1494, 1453, 1375, 1246, 1147, 1025, 741, 699.

Late-stage modification of commercial drugs

ethyl 3-benzyl-4-(methyl(3-phenyl-3-(4-(trifluoromethyl)phenoxy)propyl)amino)butanoate (73)

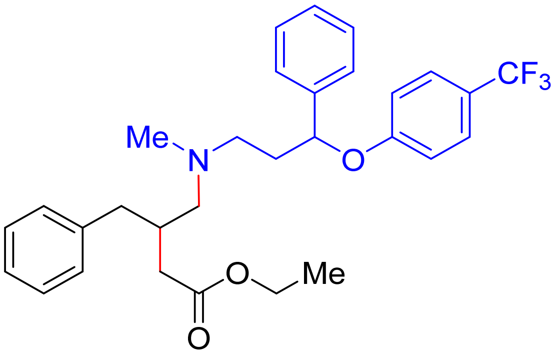

Prepared following the general procedure employing N-methyl-3-phenyl-3-(4-(trifluoromethyl) phenoxy)propan-1-amine (309 mg, 1 mmol), 3-phenylpropanal (201 mg, 1.5 mmol), ethyl 2-mercaptoacetate (180 mg, 1.5 mmol), triphenylphosphine (393 mg, 1.5 mmol), 4Å MS (1 g), di-tert-butyl peroxide (292mg, 2 mmol) in anhydrous dichloromethane (6 mL). The desired product was obtained as colorless oil (437 mg, 85% yield) after purification by flash column

chromatography on silica gel (0.5% Et_3N in dichloromethane). The dr value (1:1) was determined by ^1H NMR spectra.

^1H NMR (400 MHz, $CDCl_3$) δ 7.47 - 7.12 (m, 12H), 6.94 (d, J = 8.8 Hz, 2H), 5.35 (ddd, J = 8.4, 4.4, 1.4 Hz, 1H), 4.14 - 3.93 (m, 2H), 3.19 - 2.38 (m, 7H), 2.37 - 2.10 (m, 3H), 2.22 (s, 3H), 2.01 - 1.89 (m, 1H), 1.23 (t, J = 7.1 Hz, 1.5H, diastereomer A), 1.22 (t, J = 7.1 Hz, 1.5H, diastereomer B). ^{13}C NMR (101 MHz, $CDCl_3$, double carbon signals) δ 173.30, 173.23, 160.82, 141.49, 141.43, 139.93, 139.92, 129.31, 129.31, 129.05, 128.97, 128.81, 128.76, 128.55, 128.47, 128.31, 127.82, 127.81, 126.95 – 126.67 (m), 126.64, 126.50, 126.08, 125.94, 125.92, 123.02 (d, J = 30.4 Hz), 122.38 (d, J = 32.5 Hz), 115.85, 78.34, 78.30, 62.12, 62.00, 58.77, 58.37, 54.41, 54.37, 42.48, 42.46, 39.01, 38.94, 37.38, 37.33, 36.65, 36.59, 35.64, 35.50, 14.26. ^{19}F NMR (376 MHz, $CDCl_3$) δ -61.39 (s), -61.40 (s). HRMS (ESI) calcd for $C_{30}H_{35}F_3NO_3$ $[M + H]^+$ m/z = 514.2564, found: 514.2566. IR ν_{max}/cm^{-1} (film): 2932, 1730, 1614, 1518, 1495, 1453, 1327, 1251, 1160, 1118, 1067, 1030, 835, 746, 700.

ethyl 3-benzyl-4-(((1S,4S)-4-(3,4-dichlorophenyl)-1,2,3,4-tetrahydronaphthalen-1-yl) (methyl) amino)butanoate (74)

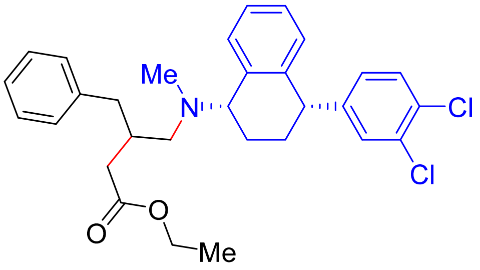

Prepared following the general procedure employing (1S,4S)-4-(3,4-dichlorophenyl)-N-methyl-1,2,3,4-tetrahydronaphthalen-1-amine (305 mg, 1 mmol), 3-phenylpropanal (201 mg, 1.5 mmol), ethyl 2-mercaptoacetate (180 mg, 1.5 mmol), triphenylphosphine (393 mg, 1.5 mmol), 4Å MS (1 g), di-tert-butyl peroxide (292 mg, 2 mmol) in anhydrous dichloromethane (6 mL). The desired product was obtained as colorless oil (417 mg, 82% yield) after purification by flash column chromatography on silica gel (0.5% Et_3N in petroleum ether: ethyl acetate = 3:1). The dr value (2:1) was determined by ^1H NMR spectra.

^1H NMR (400 MHz, $CDCl_3$) δ 7.85 (d, J = 7.8 Hz, 1H), 7.41 - 7.00 (m, 9H), 6.95 - 6.77 (m, 2H), 4.20 - 4.01 (m, 3H), 3.99 - 3.82 (m, 1H), 2.98 - 2.26 (m, 7H), 2.21 (s, 2H, major diastereomer), 2.17 (s, 1H, minor diastereomer), 2.13 - 1.89 (m, 2H), 1.73 - 1.51 (m, 2H), 1.25 (td, J = 7.1, 2.9 Hz, 3H). ^{13}C NMR (101 MHz, $CDCl_3$, double carbon signals) δ 173.4, 173.4, 164.4, 147.6, 140.2, 140.1, 139.6, 139.5, 138.2, 138.2, 132.2, 130.8, 130.3, 130.3, 130.0, 130.0, 129.9, 129.9, 129.4, 129.4, 128.7, 128.7, 128.4, 128.3, 128.3, 127.1, 126.9, 126.9, 126.1, 126.1, 62.6, 62.5, 60.2, 58.7, 57.5, 43.7, 38.9, 37.1, 37.0, 36.2, 35.8, 35.7, 30.2, 30.2, 15.5, 15.4, 14.4. HRMS (ESI) calcd for $C_{30}H_{34}Cl_2NO_2$ $[M + H]^+$ m/z = 510.1961, found: 510.1962. IR ν_{max}/cm^{-1} (film): 2936, 2855, 1731, 1466, 1452, 1373, 1252, 1149, 1029, 784, 740, 700.

ethyl 3-benzyl-4-(4-(8-chloro-5,6-dihydro-11H-benzo[5,6]cyclohepta[1,2-b]pyridin-11-ylidene) piperidin-1-yl)butanoate (75)

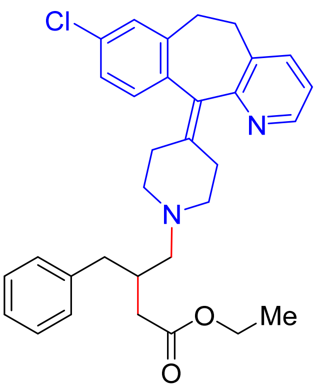

Prepared following the general procedure employing 8-chloro-11-(piperidin-4-ylidene)-6,11-dihydro-5H-benzo[5,6]cyclohepta[1,2-b]pyridine (310 mg, 1 mmol), 3-phenylpropanal (201 mg, 1.5 mmol), ethyl 2-mercaptoacetate (180 mg, 1.5 mmol), triphenylphosphine (393 mg, 1.5 mmol), 4Å MS (1 g), di-tert-butyl peroxide (292 mg, 2 mmol) in anhydrous dichloromethane (6 mL). The desired product was obtained as colorless oil (411 mg, 80% yield) after purification by flash column chromatography on silica gel (0.5% Et_3N in petroleum ether: ethyl acetate = 1:1).

^{1}H NMR (400 MHz, $CDCl_3$) δ 8.36 (d, J = 4.6 Hz, 1H), 7.39 (d, J = 7.5 Hz, 1H), 7.29 - 6.91 (m, 9H), 4.06 (q, J = 7.2 Hz, 2H), 3.42 - 3.27 (m, 2H), 2.88 - 2.60 (m, 5H), 2.59 - 2.50 (m, 1H), 2.48 - 2.36 (m, 2H), 2.33 - 1.88 (m, 9H), 1.21 (t, J = 7.2 Hz, 3H). ^{13}C NMR (101 MHz, $CDCl_3$) δ 173.4, 157.8, 146.7, 140.0, 139.6, 139.5, 138.0, 137.3, 133.5, 132.7, 132.4, 131.0, 130.0, 129.4, 129.0, 128.3, 126.1, 122.1, 62.4, 60.3, 55.7, 55.0, 39.2, 38.0, 35.0, 32.0, 31.6, 31.2, 31.0, 14.4. HRMS (ESI) calcd for $C_{32}H_{36}ClN_2O_2$ $[M + H]^+$ m/z = 515.2460, found: 515.2461. IR ν_{max}/cm^{-1} (film): 2924, 1730, 1477, 1437, 1371, 1150, 1029, 829, 744, 700.

ethyl 3-benzyl-4-((3-(10,11-dihydro-5H-dibenzo[a,d][7]annulen-5-ylidene)propyl)(methyl)amino)butanoate (76)

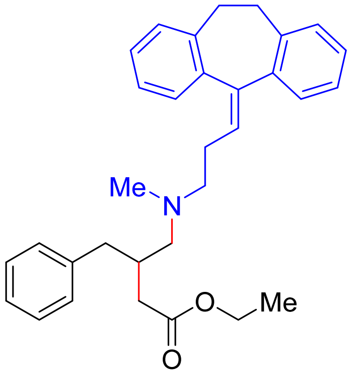

Prepared following the general procedure employing 3-(10,11-dihydro-5H-dibenzo[a,d][7]annulen-5-ylidene)-N-methylpropan-1-amine (263 mg, 1 mmol), 3-phenylpropanal (201 mg, 1.5 mmol), ethyl 2-mercaptoacetate (180 mg, 1.5 mmol), triphenylphosphine (393 mg, 1.5 mmol), 4Å MS (1 g), di-tert-butyl peroxide (292 mg, 2 mmol) in anhydrous dichloromethane (6 mL). The desired product was obtained as colorless oil (397 mg, 85% yield) after purification by flash column chromatography on silica gel (0.5% Et_3N in petroleum ether: ethyl acetate = 3:1).

^{1}H NMR (400 MHz, $CDCl_3$) δ 7.50 - 6.91 (m, 13H), 5.93 (t, J = 7.4 Hz, 1H), 4.05 (q, J = 7.0 Hz, 2H), 3.55 - 2.93 (m, 4H), 2.91 - 2.67 (m, 2H), 2.61 - 2.20 (m, 8H), 2.18 (s, 3H), 2.04 - 1.78 (m, 1H), 1.23 (t, J = 7.1 Hz, 3H). ^{13}C NMR (101 MHz, $CDCl_3$) δ 173.3, 143.4, 141.5, 140.2, 140.0, 139.4, 137.1, 130.0, 129.8, 129.4, 128.6, 128.3, 128.3, 128.1, 127.4, 127.0, 126.0, 126.0, 125.8, 61.7, 60.1, 58.0, 42.3, 38.9, 37.3, 35.6, 33.8, 32.2, 27.5, 14.3. HRMS (ESI) calcd for $C_{32}H_{38}NO_2$ $[M + H]^+$ m/z = 468.2897, found: 468.2897. IR ν_{max}/cm^{-1} (film): 3024, 2934, 1731, 1495, 1485, 1453, 1371, 1158, 1037, 768, 754, 700.

Modified Haloperidol (77)

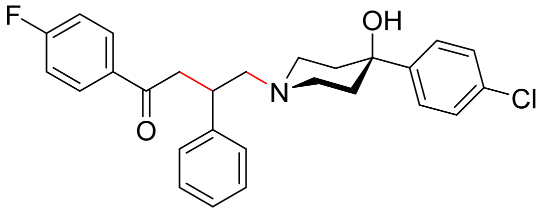

Prepared following the general procedure employing 4-(4-chlorophenyl)piperidin-4-ol (211 mg, 1 mmol), 2-phenylacetaldehyde (180 mg, 1.5 mmol), 1-(4-fluorophenyl)-2-mercaptoethan-1-one (255 mg, 1.5 mmol), triphenylphosphine (393 mg, 1.5 mmol), 4Å MS (1 g), di-tert-butyl peroxide (292 mg, 2 mmol) in anhydrous dichloromethane (6 mL). The desired product was obtained as colorless oil (248 mg, 55% yield) after purification by flash column chromatography on silica gel (0.5% Et_3N in petroleum ether: ethyl acetate = 1:2).

^{1}H NMR (400 MHz, $CDCl_3$) δ 8.03 (dd, J = 8.7, 5.5 Hz, 2H), 7.34 – 7.08 (m, 11H), 3.86 – 3.68 (m, 1H), 3.56 (dd, J = 15.2, 8.3 Hz, 1H), 3.05 – 2.73 (m, 2H), 2.73 – 2.41 (m, 4H), 2.36 – 2.21 (m, 1H), 1.73 (br, 1H), 1.65 – 1.36 (m, 4H). ^{13}C NMR (101 MHz, $CDCl_3$) δ 196.6, 165.6 (d, J = 254.3 Hz), 146.9, 143.3, 134.6, 132.8, 130.9 (d, J = 9.1 Hz), 128.8, 128.4, 127.7, 126.9, 126.1, 115.7 (d, J = 21.7 Hz), 71.0, 51.2, 48.1, 43.3, 40.4, 37.9. ^{19}F NMR (376 MHz, $CDCl_3$) δ -106.2 (s). HRMS (ESI) calcd for $C_{27}H_{28}ClFNO_2$ $[M + H]^+$ m/z = 452.1787, found: 452.1792. IR ν_{max}/cm^{-1} (film): 3441, 3028, 2922, 2817, 1727, 1673, 1596, 1491, 1451, 1406, 1372, 1299, 1231, 1155, 1094, 1042, 1011, 914, 838, 760, 700.

1,3,3-trimethyl-2-(methylene-*d*$_2$)indoline (79)

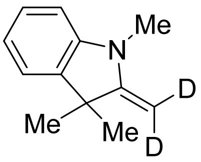

This compound is prepared by dissolving 1,3,3-trimethyl-2-methyleneindoline in D_2O.

^{1}H NMR (400 MHz, $CDCl_3$) δ 7.19 – 7.03 (m, 2H), 6.76 (t, J = 7.4 Hz, 1H), 6.54 (d, J = 7.8 Hz, 1H), 3.04 (s, 3H), 1.35 (s, 6H). ^{13}C NMR (101 MHz, $CDCl_3$) δ 162.9, 146.6, 137.7, 127.7, 121.9, 118.5, 105.0, 73.9 – 71.4 (m), 44.2, 30.1, 28.9.

ethyl 3-(1,3,3-trimethylindolin-2-yl) propanoate (80a)

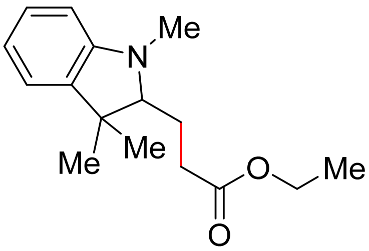

Prepared following the general procedure employing 1, 3, 3-trimethyl-2-methyleneindoline (173 mg, 1 mmol), ethyl 2-mercaptoacetate (180 mg, 1.5 mmol), triphenylphosphine (393 mg, 1.5 mmol), di-tert-butyl peroxide (292 mg, 2 mmol) in anhydrous dichloromethane (6 mL). The desired product was obtained as brown oil (222 mg, 85% yield) after purification by flash column chromatography on silica gel (0.5% Et_3N in dichloromethane).

^{1}H NMR (400 MHz, $CDCl_3$) δ 7.10 (m, 1H), 7.02 (dd, J = 7.3, 0.9 Hz, 1H), 6.75 (td, J = 7.4, 0.8 Hz, 1H), 6.52 (d, J = 7.8 Hz, 1H), 4.18 (q, J = 7.1 Hz, 2H), 2.86 (dd, J = 8.3, 4.1 Hz, 1H), 2.77 (s, 3H), 2.62 - 2.42 (m, 2H), 2.24 - 2.10 (m, 1H), 2.09 - 1.94 (m, 1H), 1.38 (s, 3H), 1.30 (t, J = 7.1 Hz, 3H), 1.13 (s, 3H). ^{13}C NMR (101 MHz, $CDCl_3$) δ 173.3, 151.6, 139.1, 127.6, 121.5, 118.6, 107.9, 76.0, 60.6, 43.1, 34.9, 31.8, 27.6, 23.7, 23.5, 14.4. HRMS (ESI) calcd for $C_{16}H_{24}NO_2$ $[M + H]^+$ m/z = 262.1802, found:262.1807. IR ν_{max}/cm^{-1} (film): 2960, 1734, 1605, 1484, 1459, 1368, 1300, 1184, 1022, 741.

ethyl 3-(1,3,3-trimethylindolin-2-yl-2-*d*)propanoate-3,3-*d*$_2$ (80)

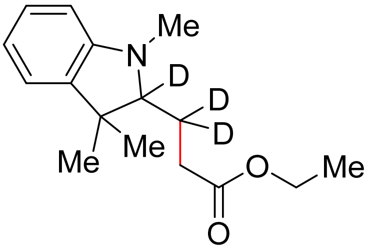

Prepared following the general procedure employing 1,3,3-trimethyl-2-(methylene-*d*$_2$)indoline (175 mg, 1 mmol), ethyl 2-mercaptoacetate (180 mg, 1.5 mmol), triphenylphosphine (393 mg, 1.5 mmol), di-tert-butyl peroxide (292 mg, 2 mmol) in dichloromethane/D_2O (6 mL, v/v=4:2). The desired product was obtained as brown oil (238 mg, 92% yield, 90% D-incorporation) after purification by flash column chromatography on silica gel (0.5% Et_3N in dichloromethane).

^{1}H NMR (400 MHz, $CDCl_3$) δ 7.11 (t, J = 7.6 Hz, 1H), 7.02 (d, J = 7.3 Hz, 1H), 6.75 (t, J = 7.4 Hz, 1H), 6.52 (d, J = 7.8 Hz, 1H), 4.18 (q, J = 7.1 Hz, 2H), 2.77 (s, 3.1H), 2.50 (dd, J = 35.8, 16.0 Hz, 2H), 1.38 (s, 3H), 1.30 (t, J = 7.1 Hz, 3H), 1.13 (s, 3H). ^{13}C NMR (101 MHz, $CDCl_3$) δ 173.2, 151.6, 151.5, 139.1, 127.5, 121.5, 118.5, 107.8, 76.1 – 74.8 (m), 60.5, 42.9, 34.8, 31.6, 27.5, 23.5, 23.3 – 22.6 (m), 14.3. HRMS (ESI) calcd for $C_{16}H_{21}D_3NO_2$ $[M + H]^+$ m/z = 265.1990, found:265.1997. IR ν_{max}/cm^{-1} (film): 2960, 1734, 1605, 1484, 1459, 1368, 1300, 1184, 1116, 1022, 741.

6. Scale-up reaction

We did the scale-up reaction for compound **4** to further demonstrate the feasibility of this protocol. The product was isolated in 86% yield on 10 mmol scale according to the general procedure under the sunlight.

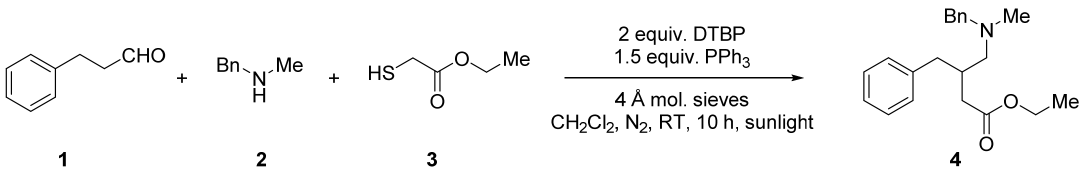

1 15 mmol, 2.0 g

2 10 mmol, 1.2 g

3 15 mmol, 1.8 g

4 2.8 g, 86% yield

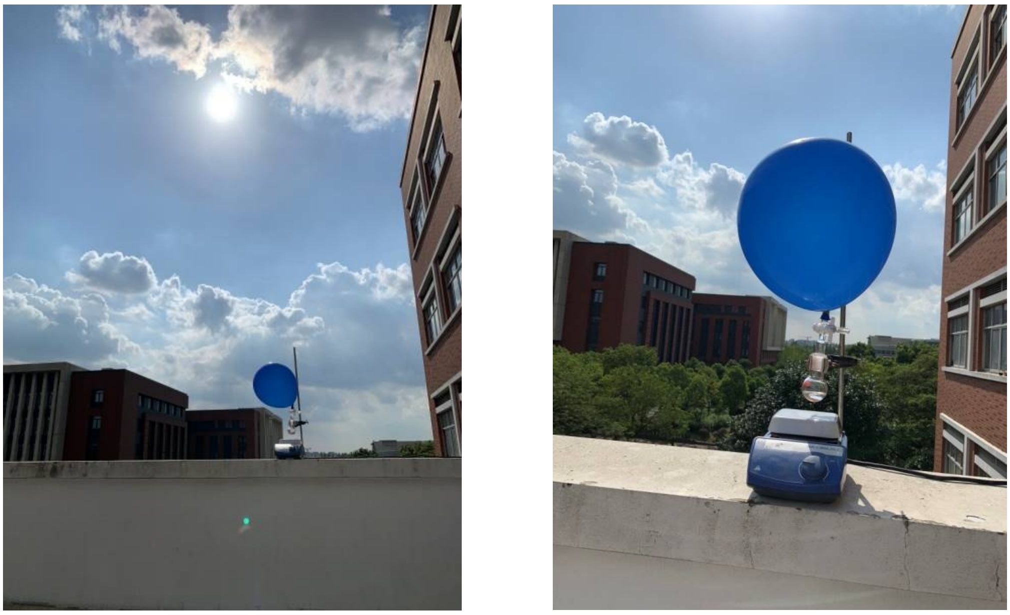

Supplementary Fig. 3 Experimental set-up for the scale-up reaction under sunlight

7. Mechanistic studies

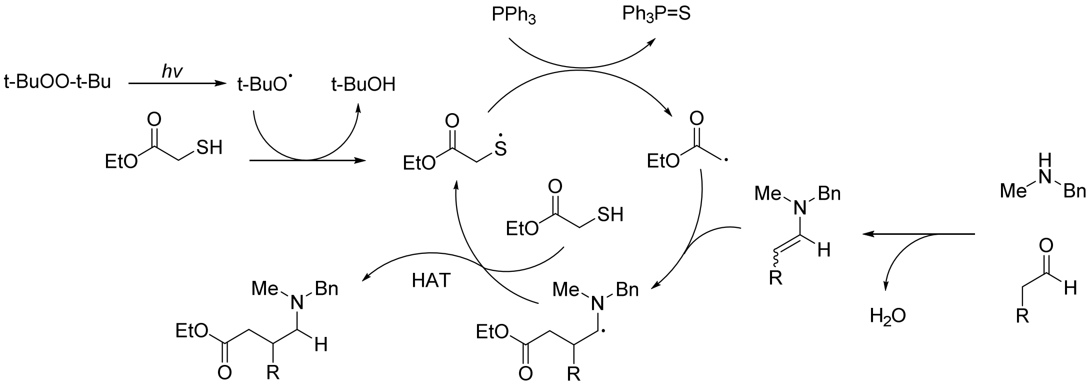

Supplementary Fig. 4 Proposed mechanism.

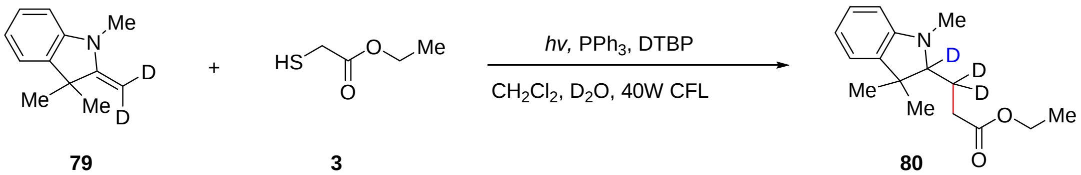

79 **3** **80**

92%, 90% D-incorporation

Supplementary Fig. 5 Deuterium-labelling study

The reaction was performed according to the general procedure employing 1, 3, 3-trimethyl-2-methyleneindoline (173 mg, 1 mmol), ethyl 2-mercaptoacetate (180 mg, 1.5 mmol), triphenylphosphine (393 mg, 1.5 mmol), di-tert-butyl peroxide (292 mg, 2 mmol) in dichloromethane/D_2O (6 mL, v/v=4:2). The desired product was obtained as brown oil (238 mg, 92% yield, 90% D-incorporation) after purification by flash column chromatography on silica gel (0.5% Et_3N in dichloromethane).

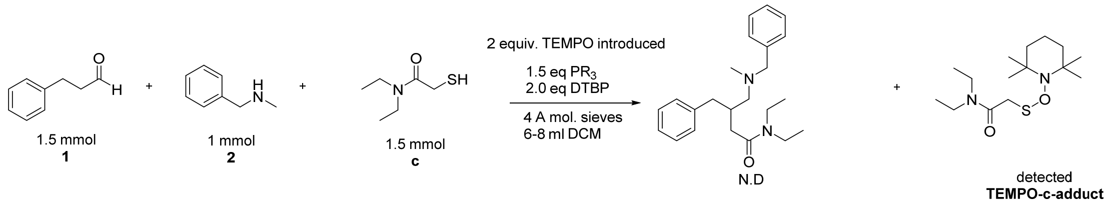

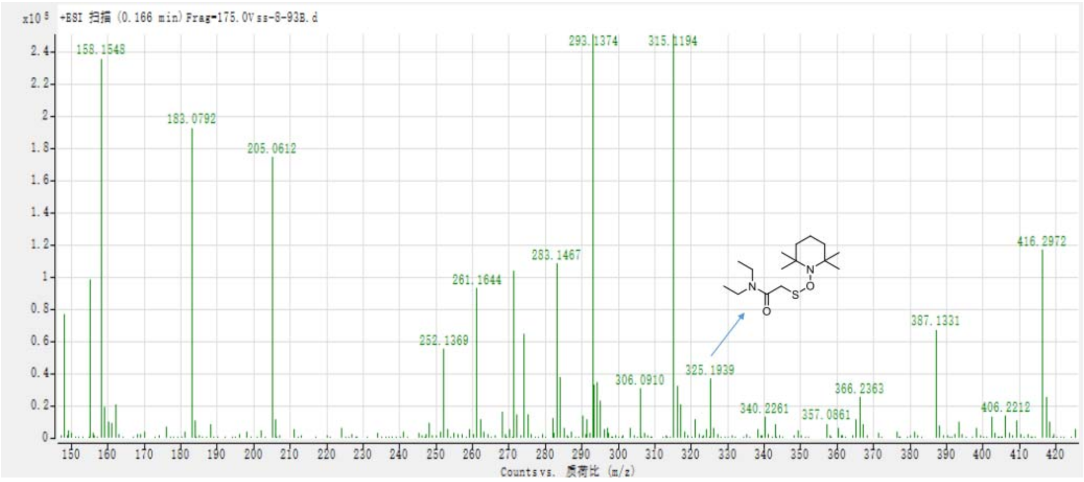

Supplementary Fig. 6 Radical trapping with TEMPO

The radical trapping experiment was executed as follows: to a 25 ml round-bottom flask equipped with a stir bar was added N-methyl-1-phenylmethanamine (121mg, 1 mmol), 3-phenylpropanal (201 mg, 1.5 mmol), N,N-diethyl-2-mercaptoacetamide (220 mg, 1.5 mmol), triphenylphosphine (393 mg, 1.5 mmol), 4Å MS (1 g), di-tert-butyl peroxide (292 mg, 2 mmol) in anhydrous dichloromethane (6 mL). Then, TEMPO (2.0 mmol) was introduced into the solution. The flask was charged with N_2 and the reaction was stirred and irradiated using two 40 W household CFL bulb (6 cm away, to keep the reaction at room temperature) at room temperature for 10 hours. After 10 hours, the reaction was monitor by LC-MS. Substrate and TEMPO-**c**-adduct can be detected in mass spectrometry. However, the product was not found.

8. Spectral data

4

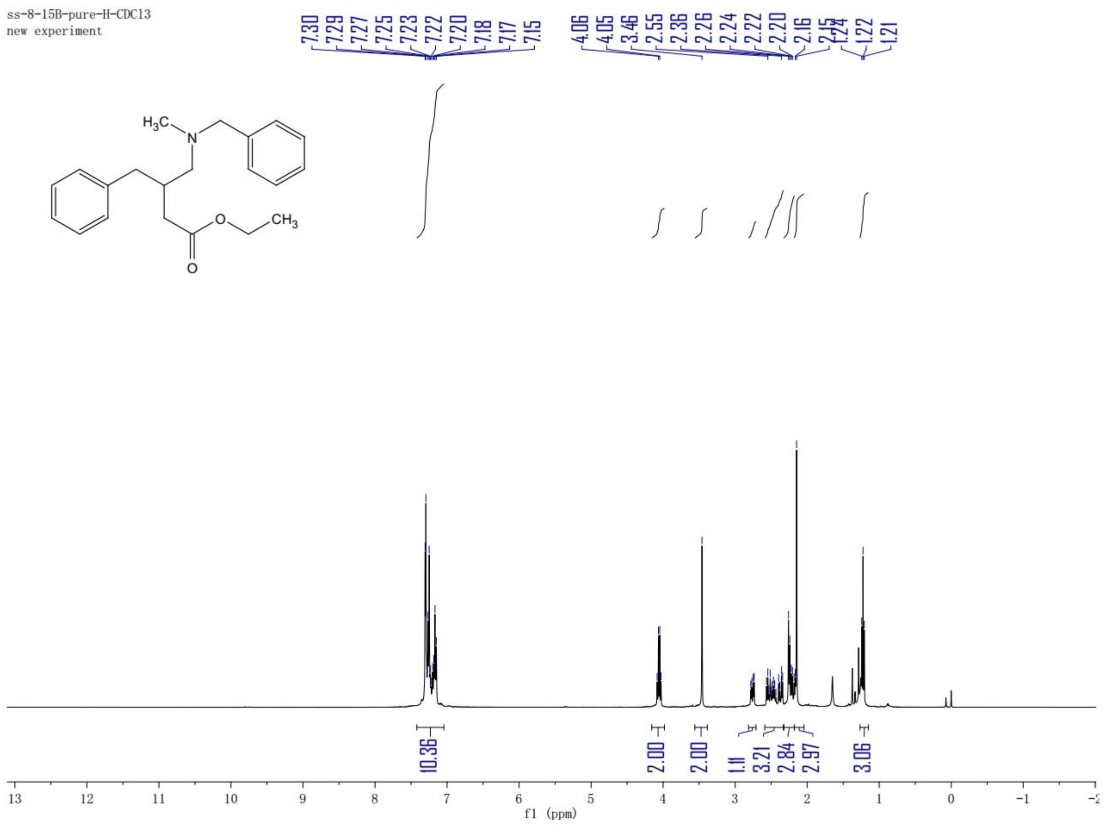

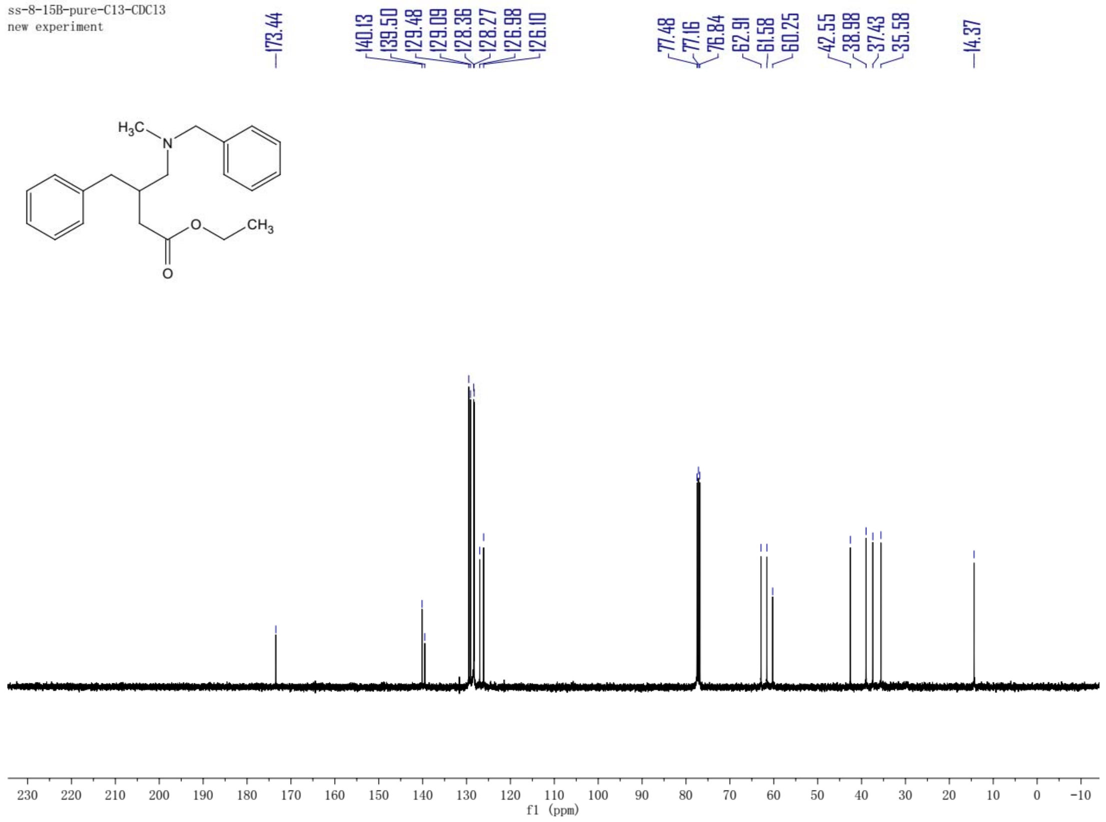

5

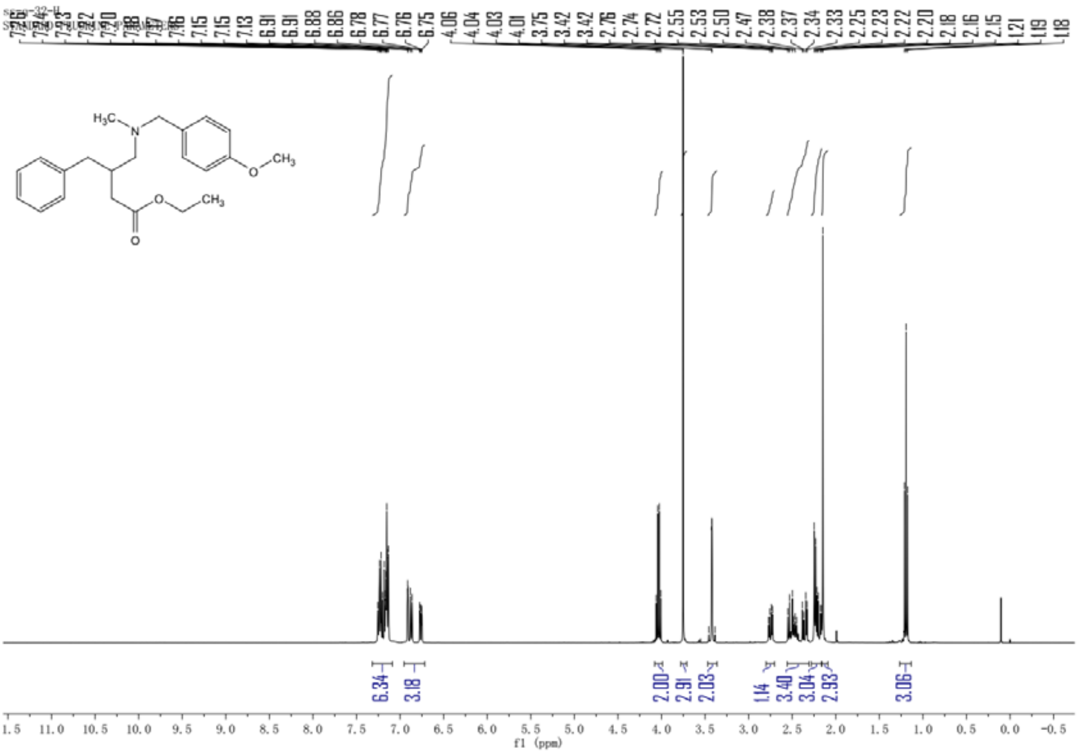

7.15
7.15
7.13
6.91
6.91
6.88
6.86
6.78
6.77
6.76
6.75
4.06
4.04
4.03
4.01
3.75
3.42
3.42
2.76
2.74
2.72
2.55
2.53
2.50
2.47
2.38
2.37
2.34
2.33
2.25
2.23
2.22
2.20
2.18
2.16
2.15
1.21
1.19
1.18
H3C
N
O
CH3
O
CH3
O
6.34
3.18
2.00
2.91
2.03
1.14
3.40
3.04
2.93
3.06
f1 (ppm)

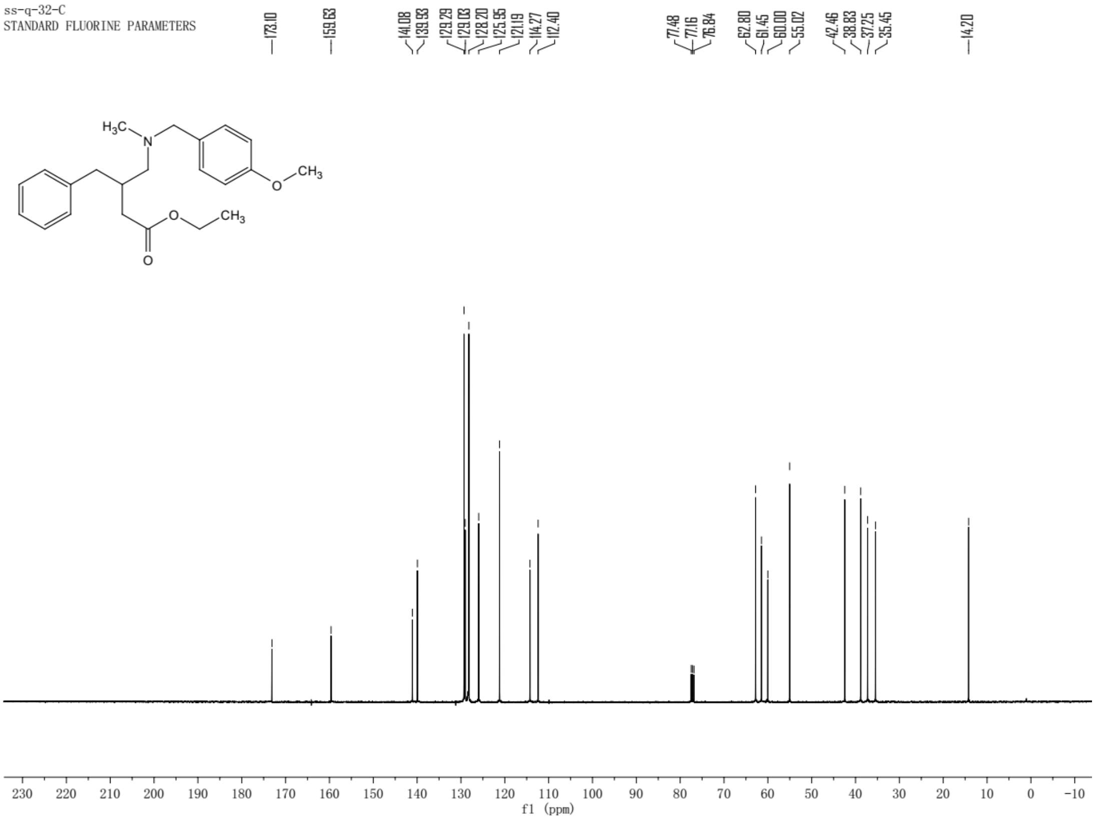

ss-q-32-C
STANDARD FLUORINE PARAMETERS
173.10
159.63
141.08
139.93
129.29
129.03
128.20
125.95
121.19
114.27
112.40
77.48
77.16
76.84
62.80
61.45
60.00
55.02
42.46
38.83
37.25
35.45
14.20
H3C
N
O
CH3
O
CH3
O
f1 (ppm)

6

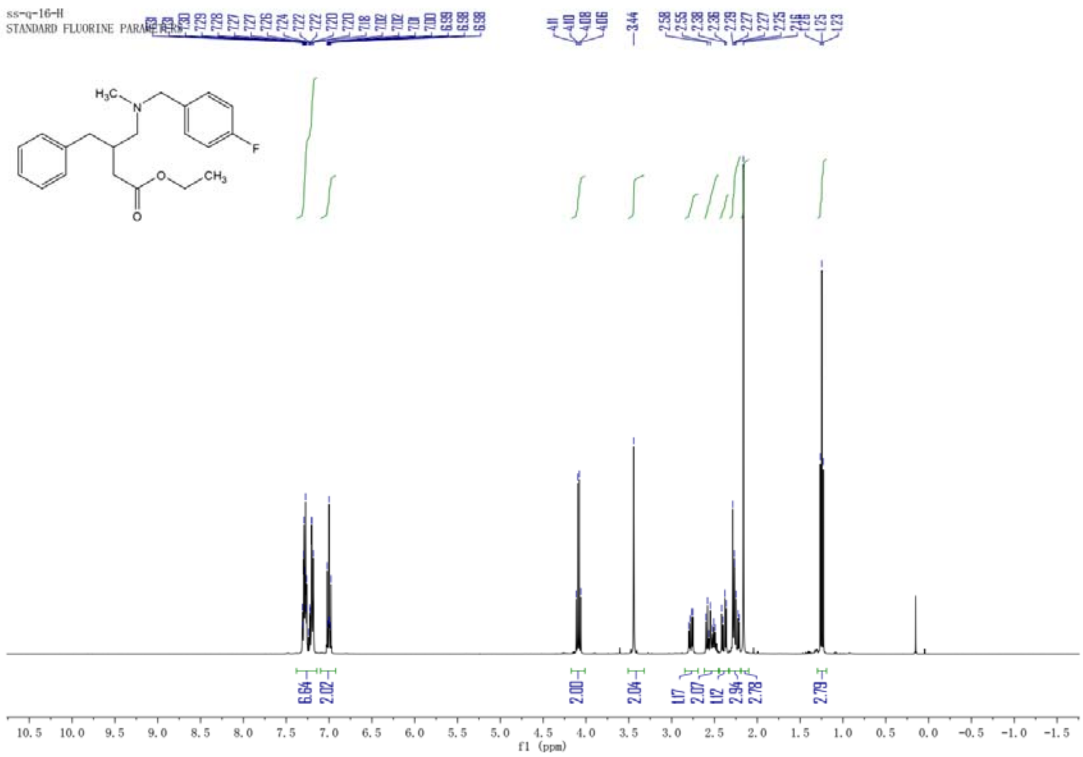

ss-q-16-H
STANDARD FLUORINE PARAMETERS
f1 (ppm)

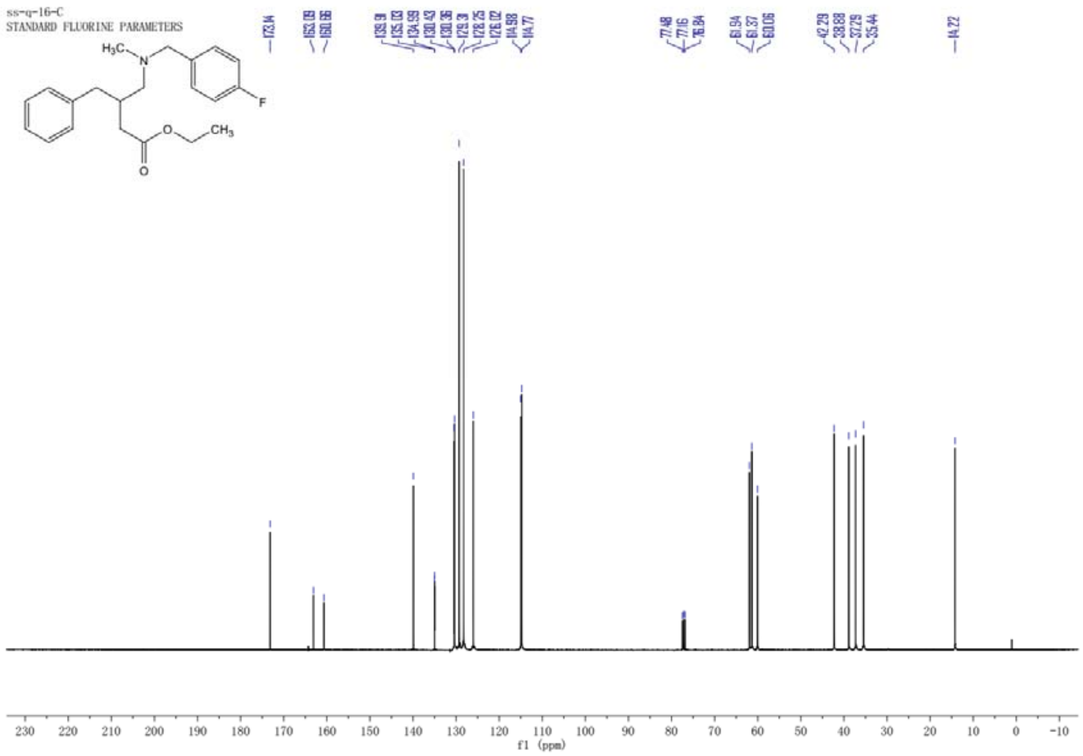

ss-q-16-C
STANDARD FLUORINE PARAMETERS
f1 (ppm)

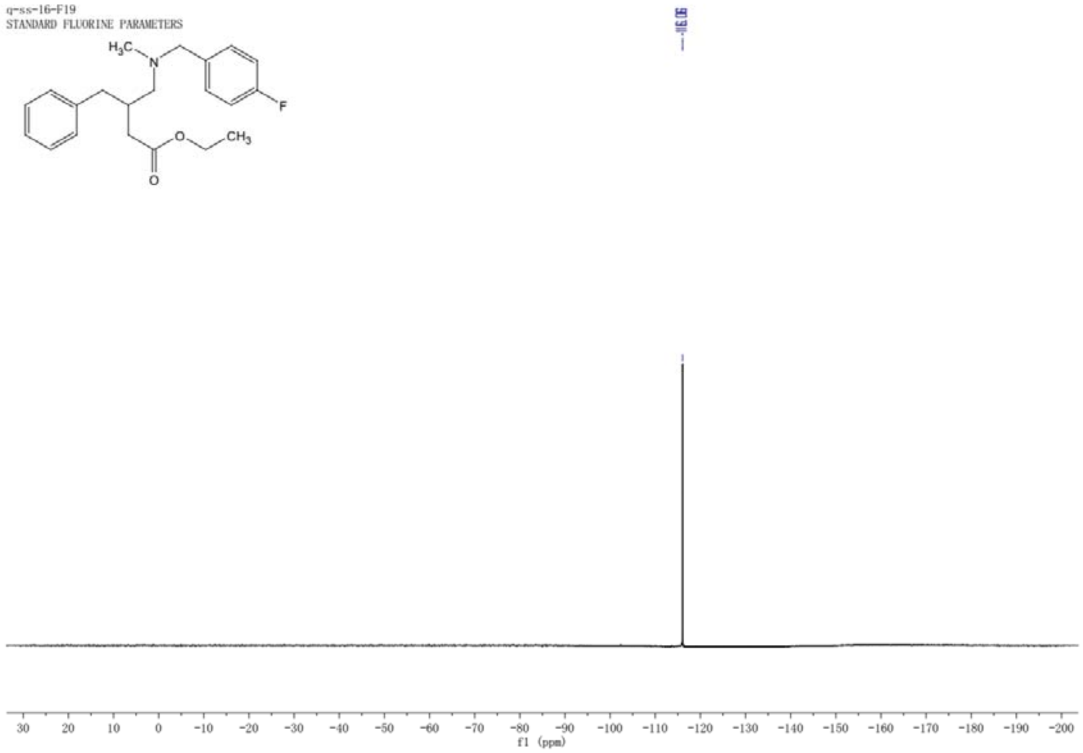
q-ss-16-F19
STANDARD FLUORINE PARAMETERS
f1 (ppm)

7

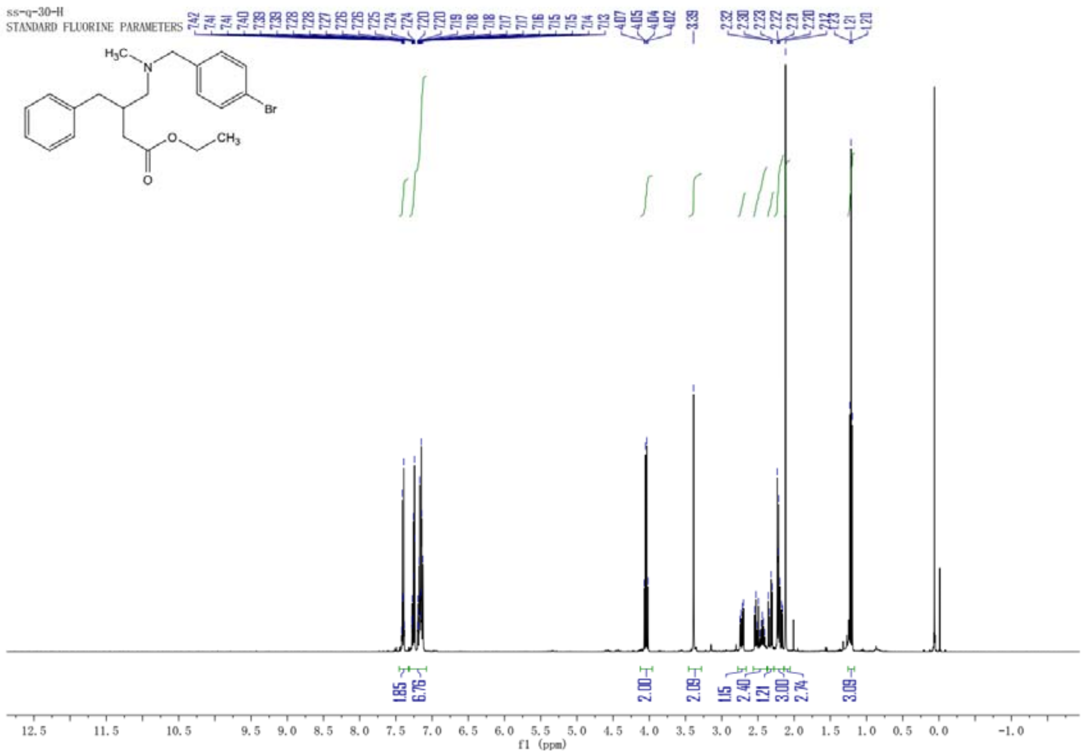
ss-q-30-H
STANDARD FLUORINE PARAMETERS
f1 (ppm)

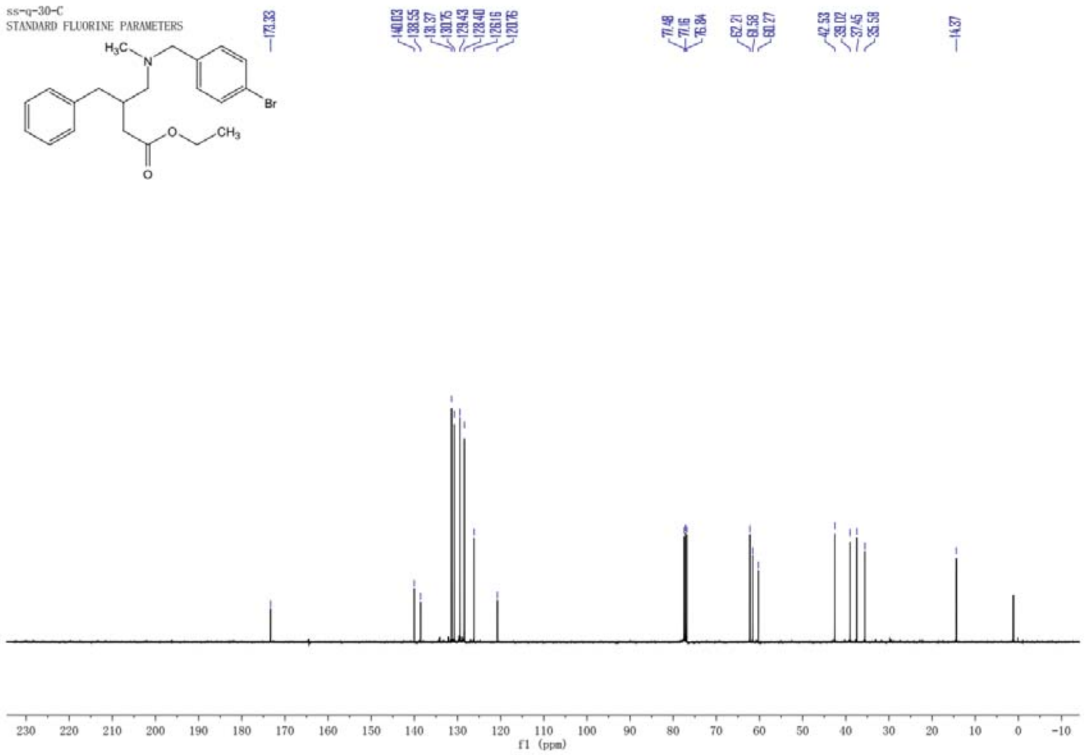

8

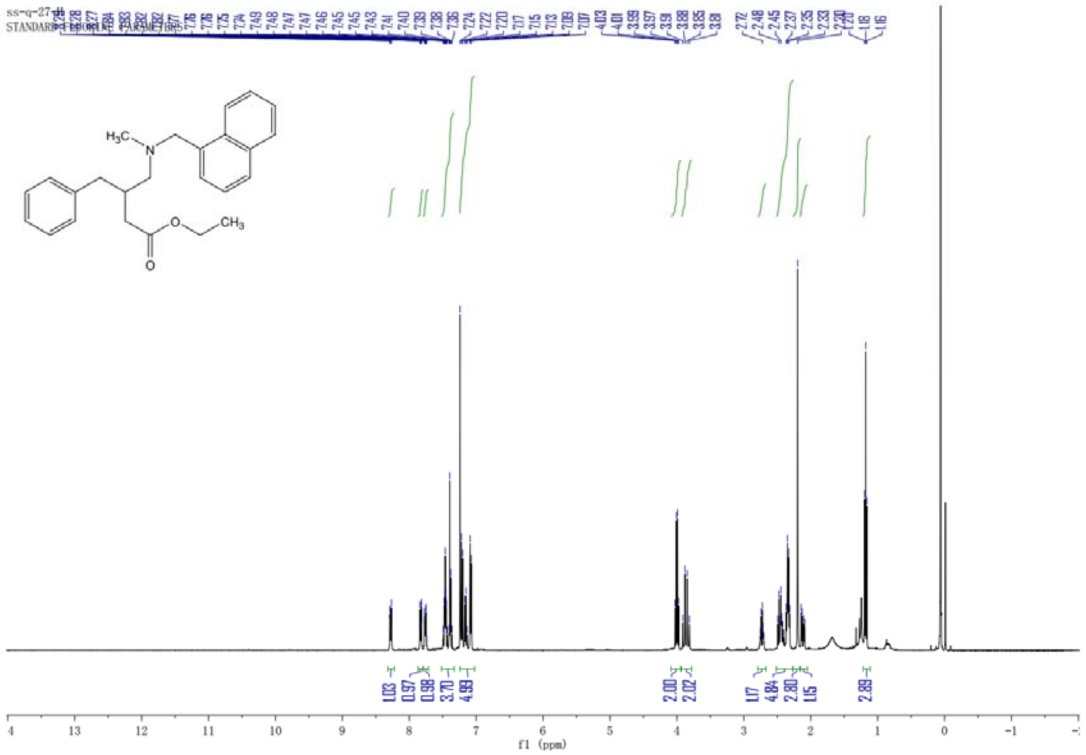

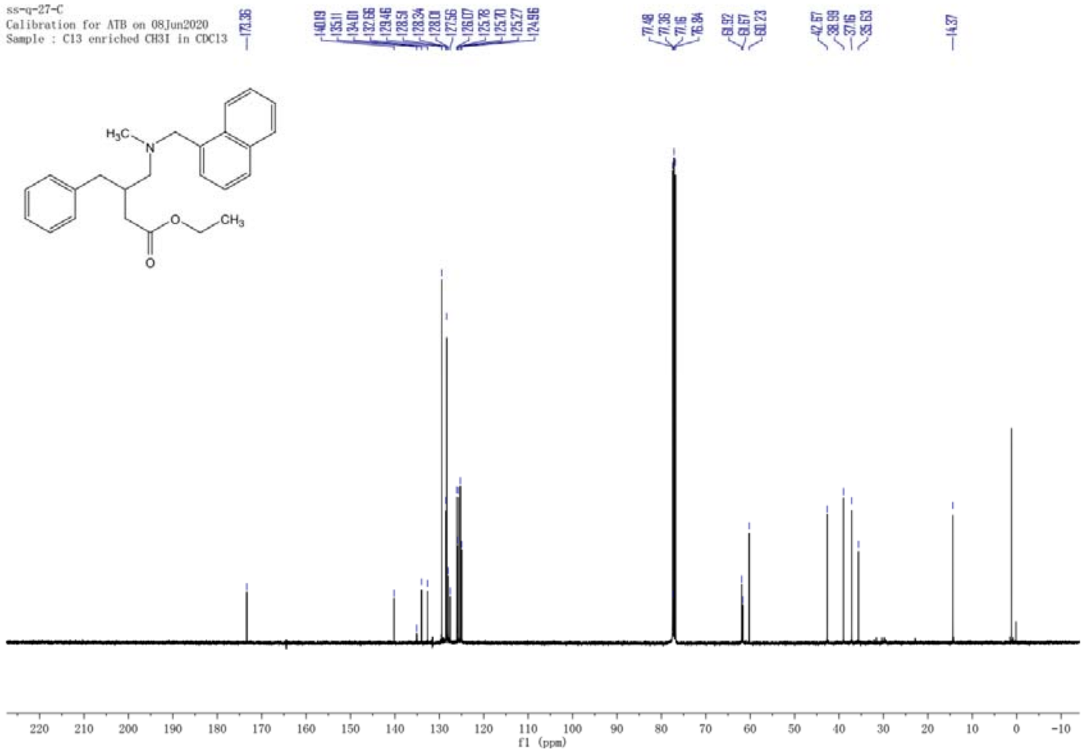
ss-q-27-C
Calibration for ATB on 08Jun2020
Sample : C13 enriched CH3I in CDCl3
f1 (ppm)

9

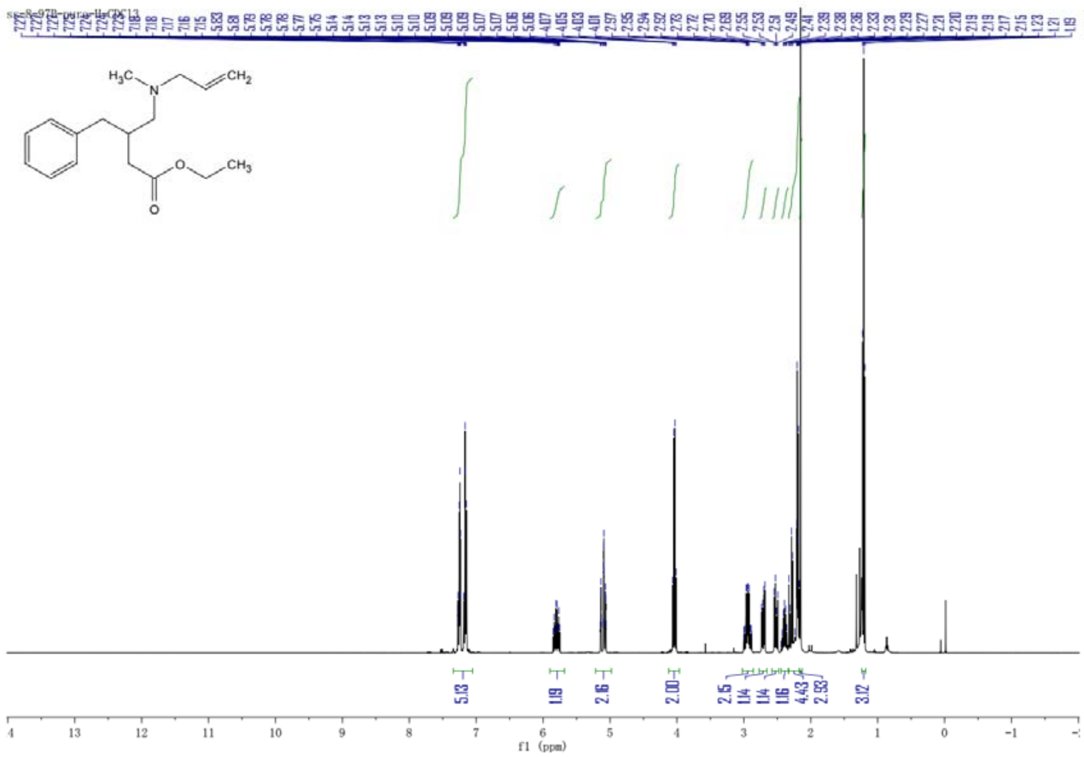
f1 (ppm)

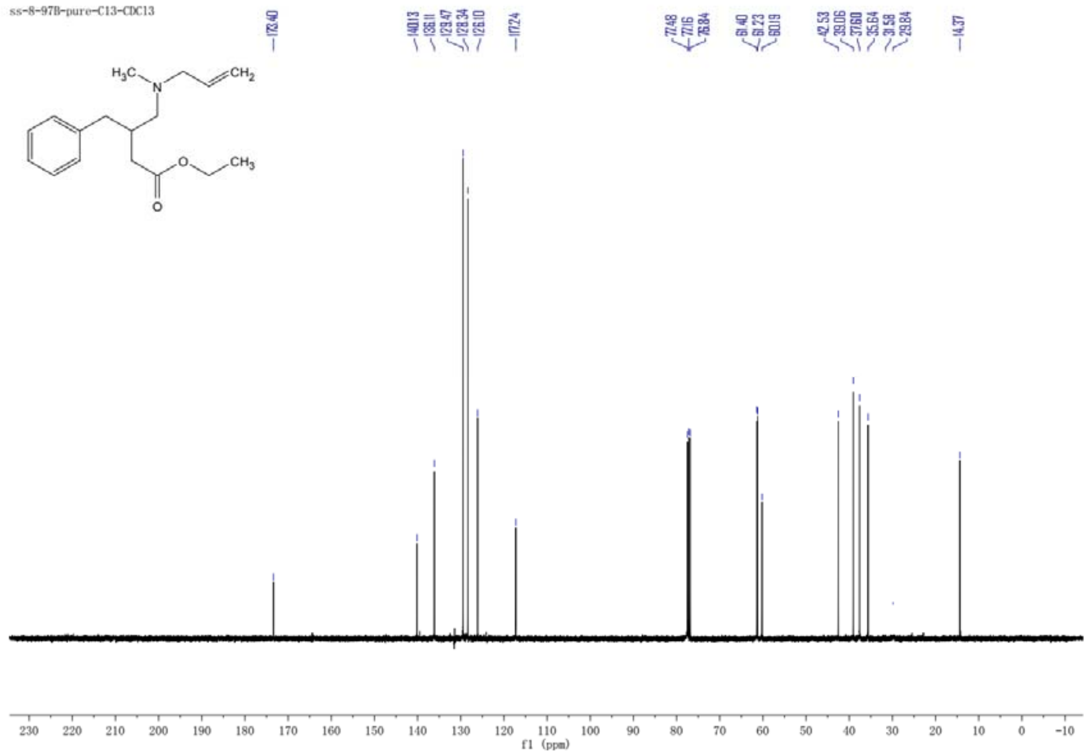
ss-8-97B-pure-C13-CDCl3
173.40
140.13
138.11
129.47
128.34
126.10
117.24
77.48
77.16
76.84
61.40
61.23
60.19
42.53
39.06
37.60
35.64
31.58
29.84
14.37
f1 (ppm)

10

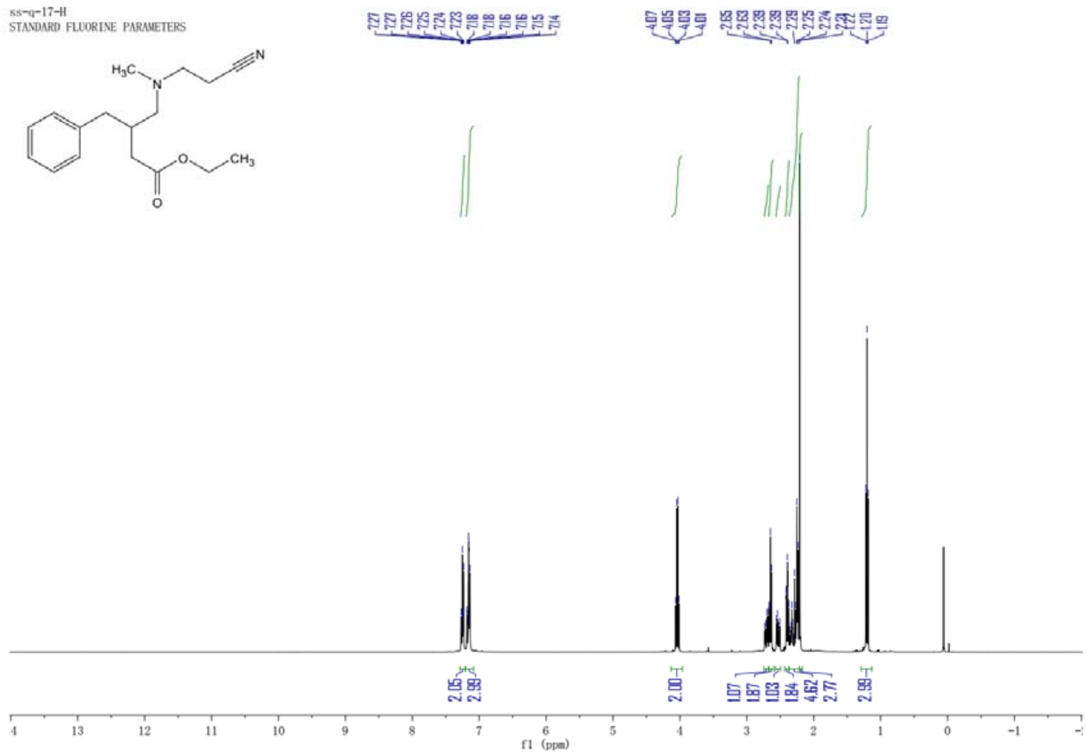
ss-q-17-H
STANDARD FLUORINE PARAMETERS
f1 (ppm)

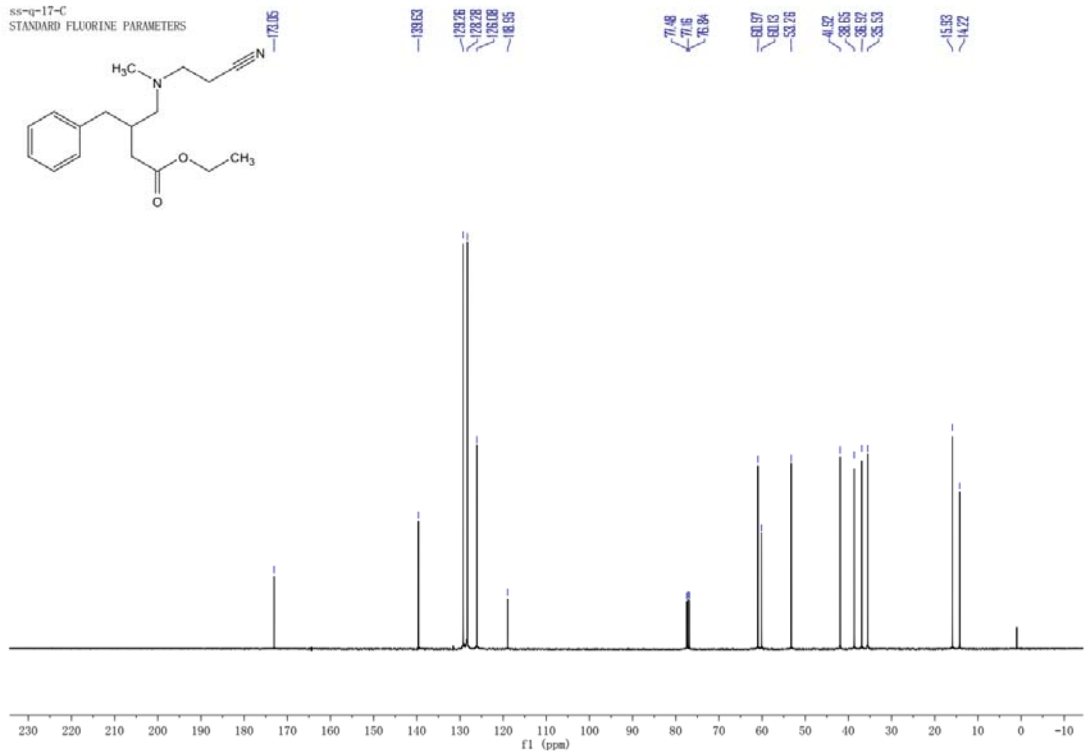

11

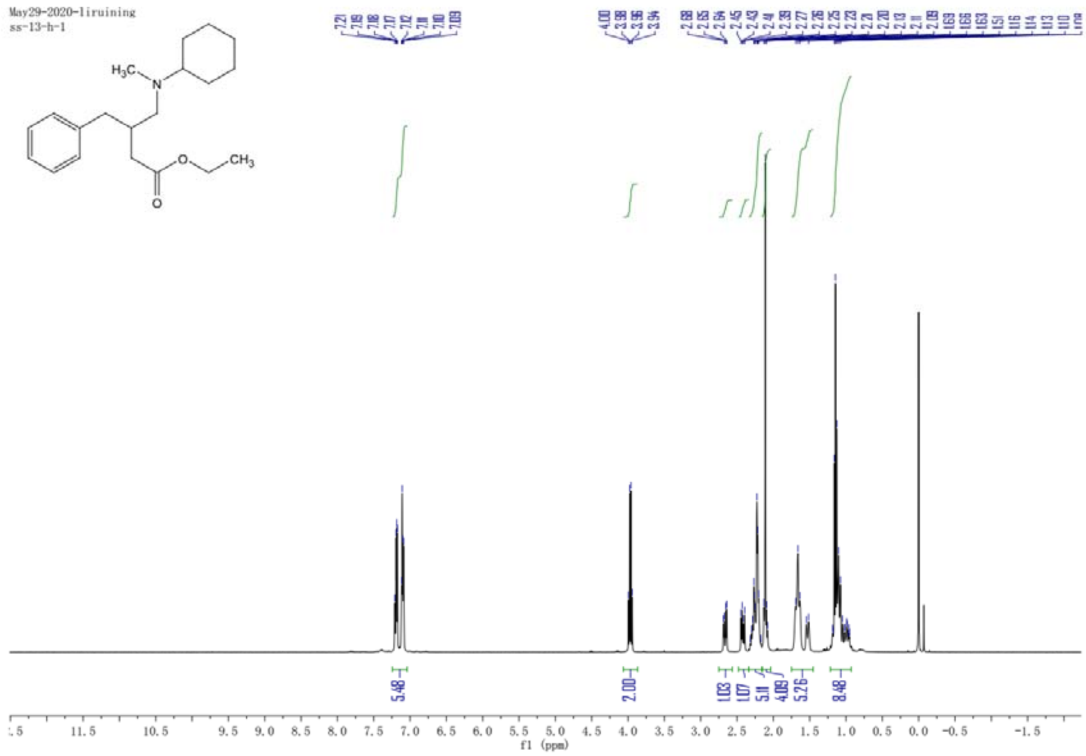

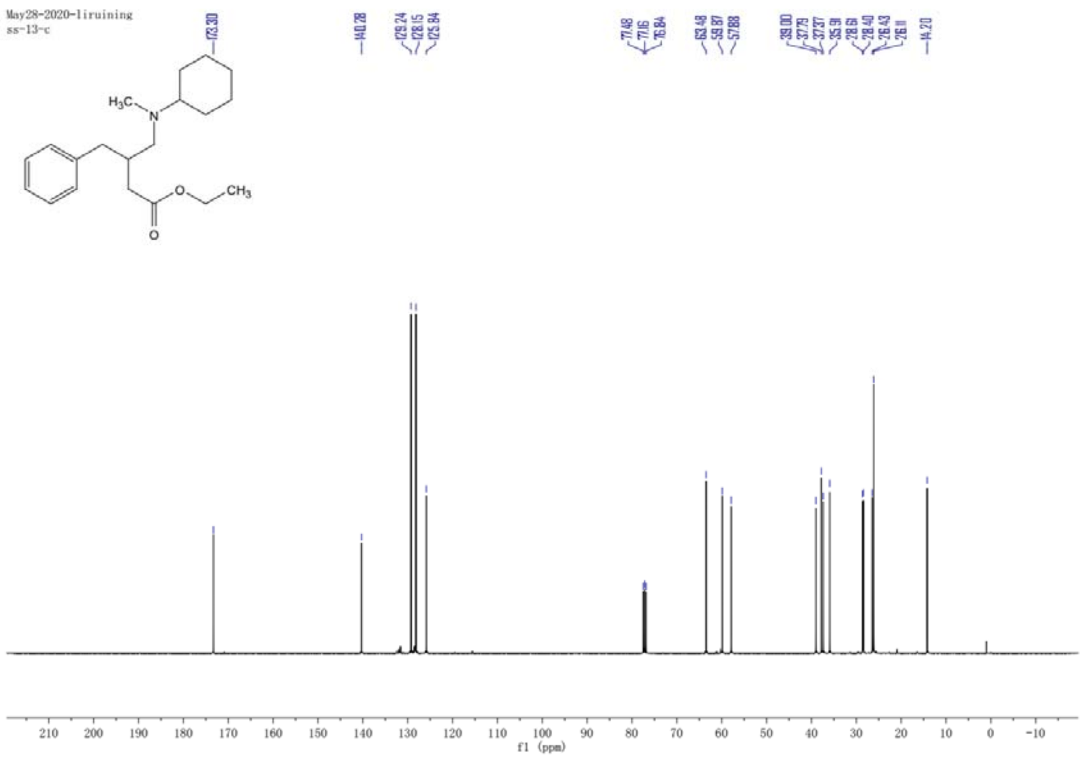
May28-2020-liruining
ss-13-c
f1 (ppm)

12

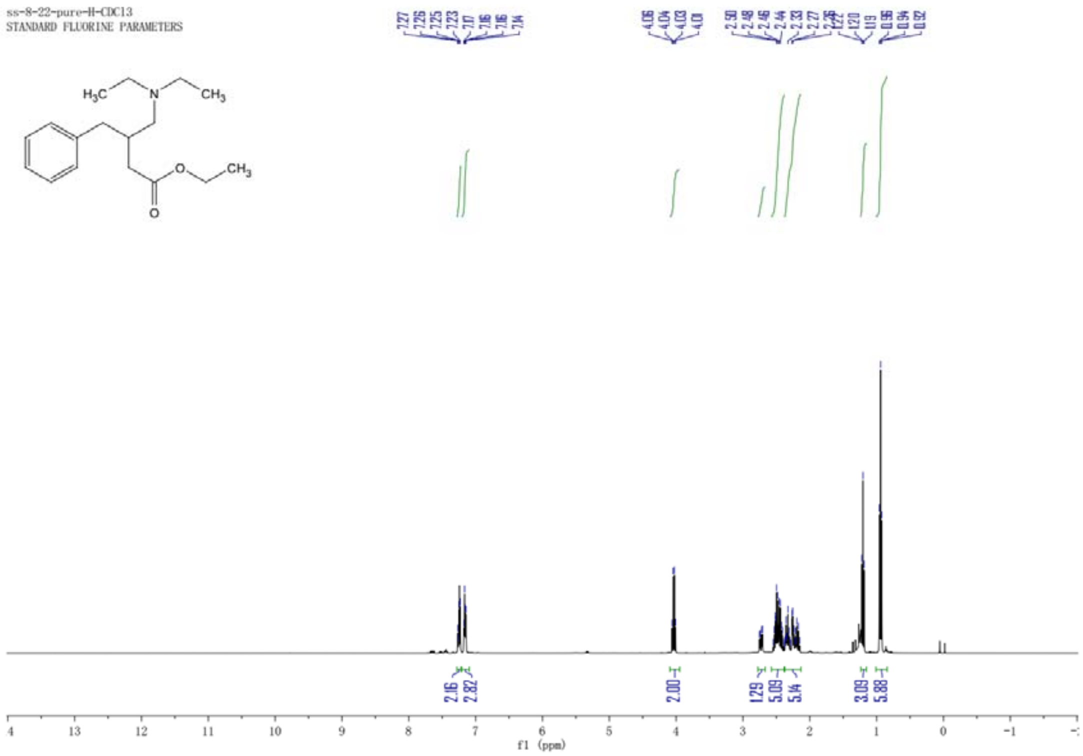
ss-8-22-pure-H-CDC13
STANDARD FLUORINE PARAMETERS
f1 (ppm)

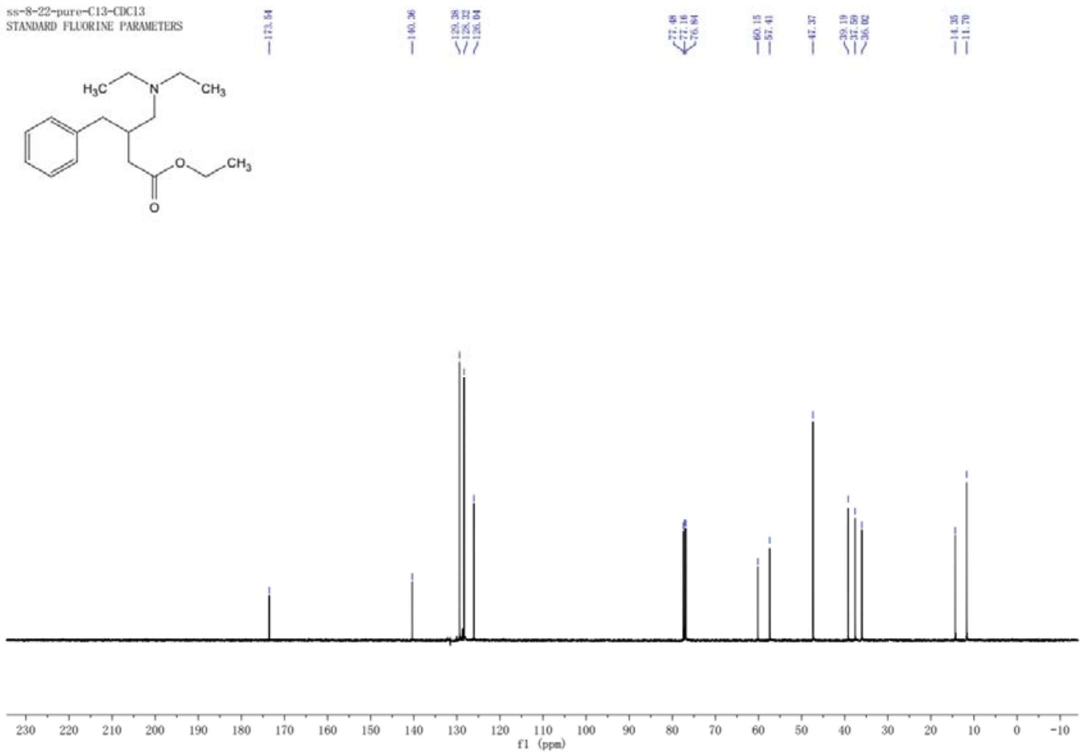
ss-8-22-pure-C13-CDCl3
STANDARD FLUORINE PARAMETERS
173.54
140.36
129.38
128.32
126.04
77.48
77.16
76.84
60.15
57.41
47.37
39.19
37.58
36.02
14.35
11.70
f1 (ppm)

13

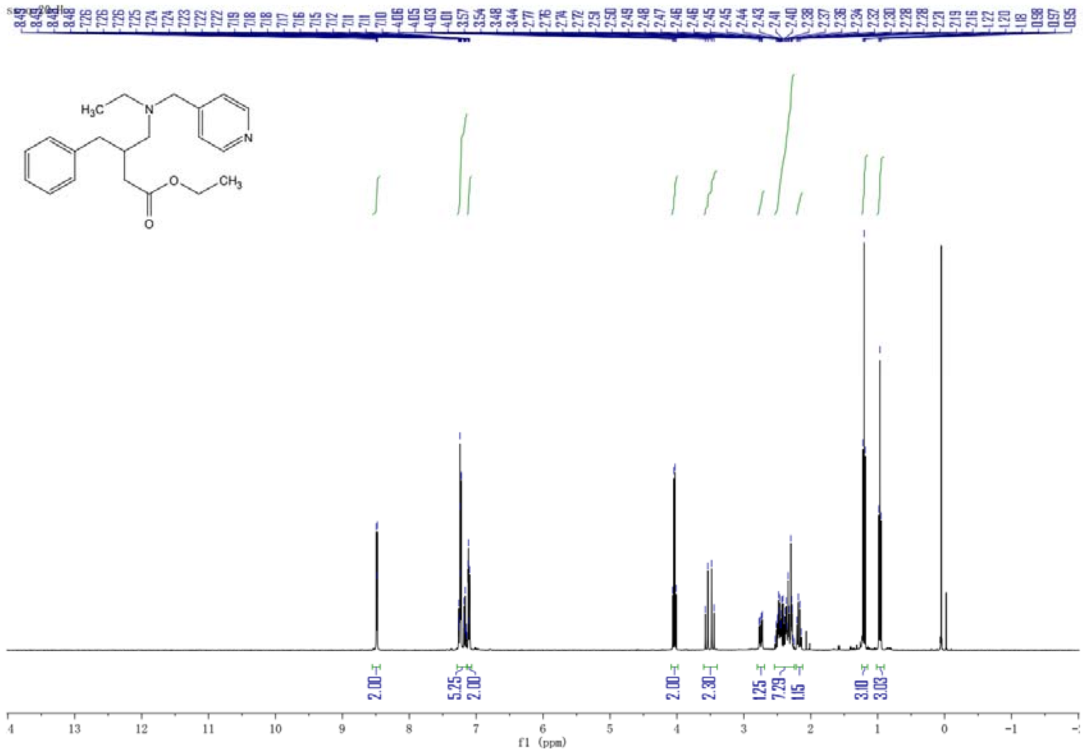
2.00
5.25
2.00
2.00
2.30
1.25
7.29
1.15
3.10
3.03
f1 (ppm)

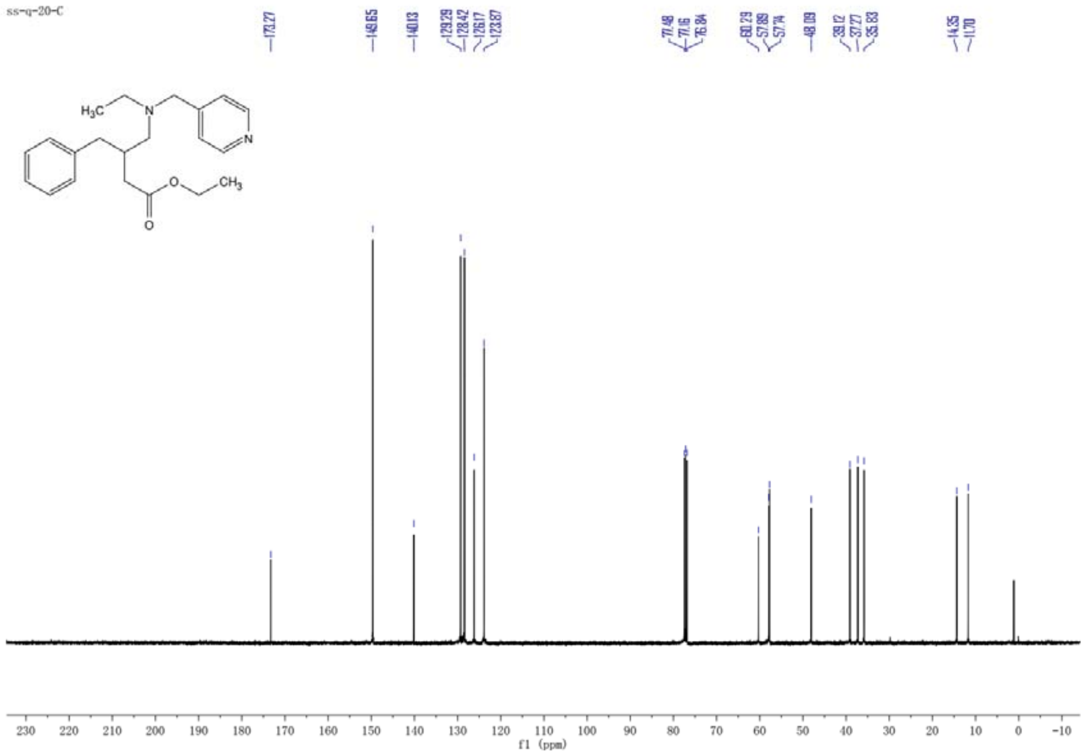
ss-q-20-C
173.27
149.65
140.13
129.29
128.42
126.17
123.87
77.48
77.16
76.84
60.29
57.89
57.74
48.09
39.12
37.77
35.83
14.35
11.70
f1 (ppm)

14

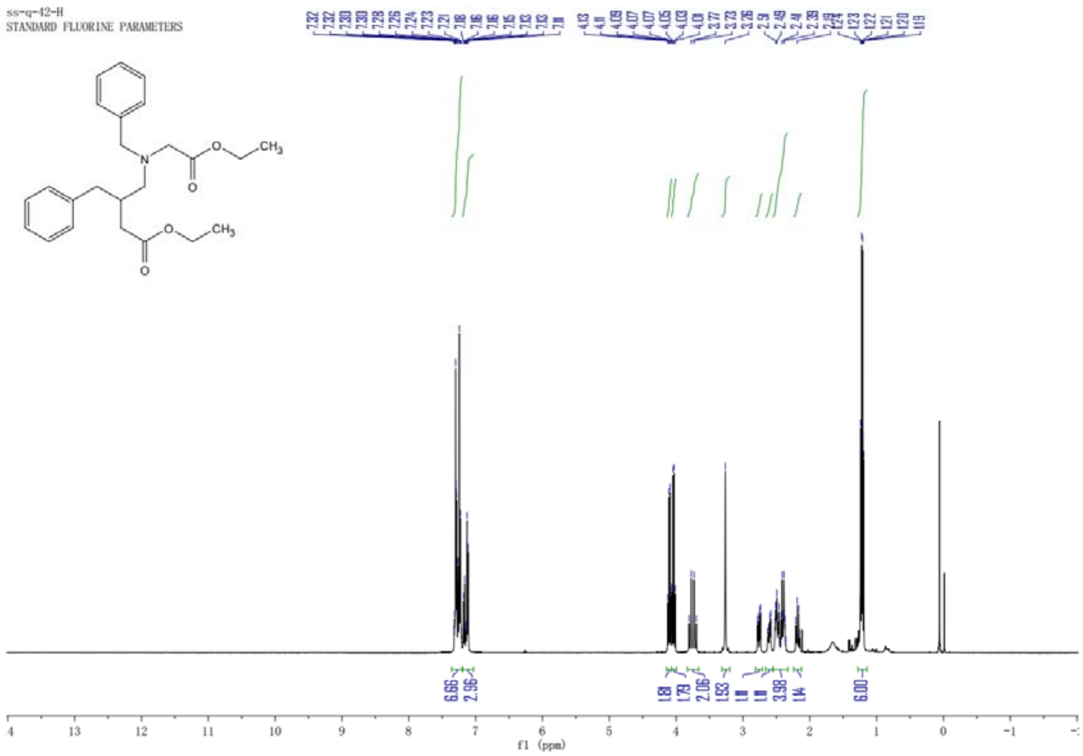
ss-q-42-H
STANDARD FLUORINE PARAMETERS
6.66
2.96
1.81
1.79
2.06
1.93
6.00
f1 (ppm)

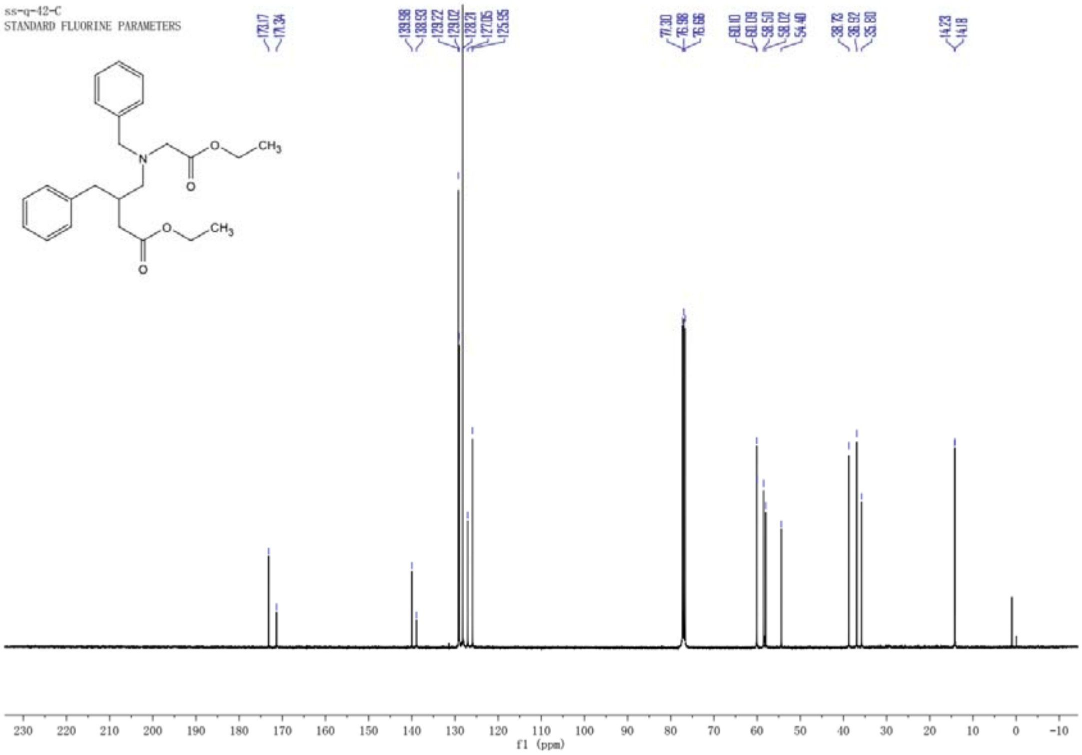
ss-q-42-C
STANDARD FLUORINE PARAMETERS
173.17
171.34
139.98
138.53
129.22
129.02
128.21
127.05
125.95
77.30
76.98
76.66
60.10
60.09
58.50
58.02
54.40
38.73
36.92
35.80
14.23
14.18
CH3
N
O
f1 (ppm)

15

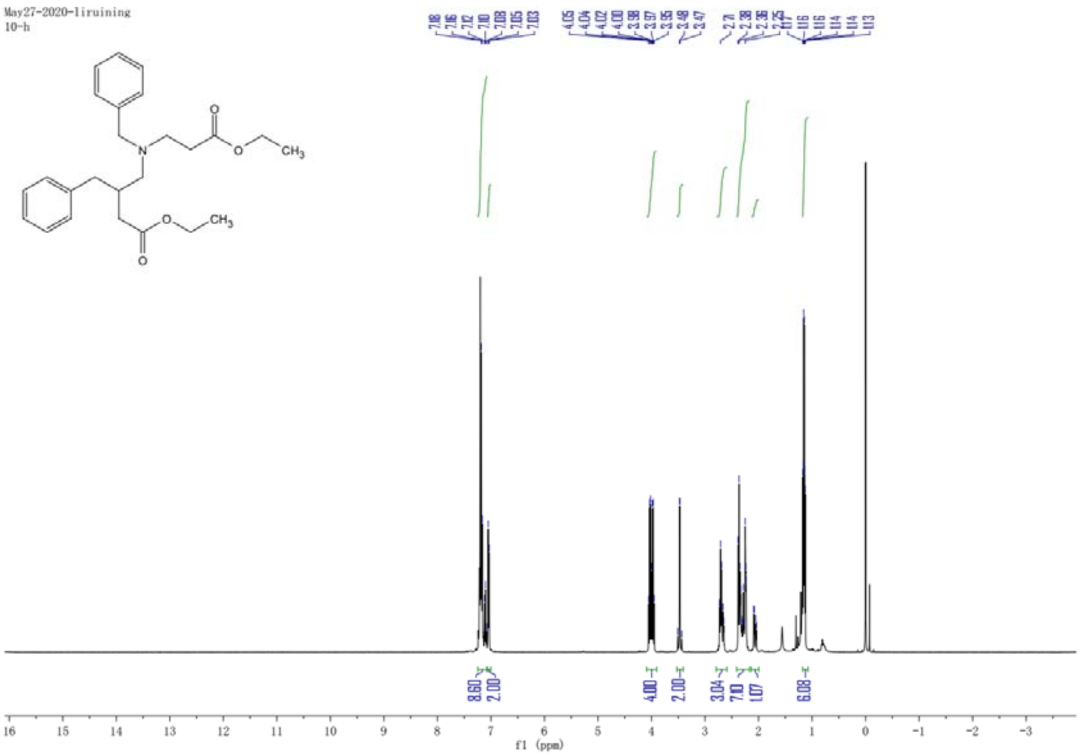
May27-2020-liruining
10-h
CH3
N
O
8.60
2.00
4.00
2.00
3.04
7.10
1.07
6.08
f1 (ppm)

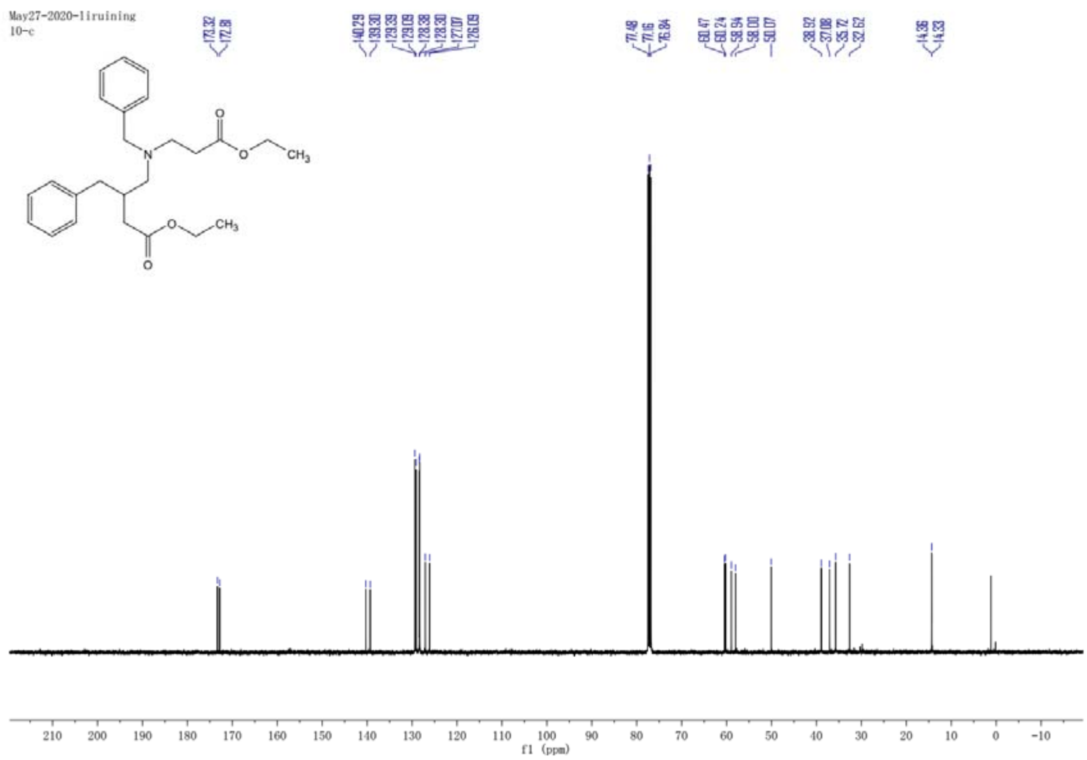
May27-2020-liruining
10-c
f1 (ppm)

16

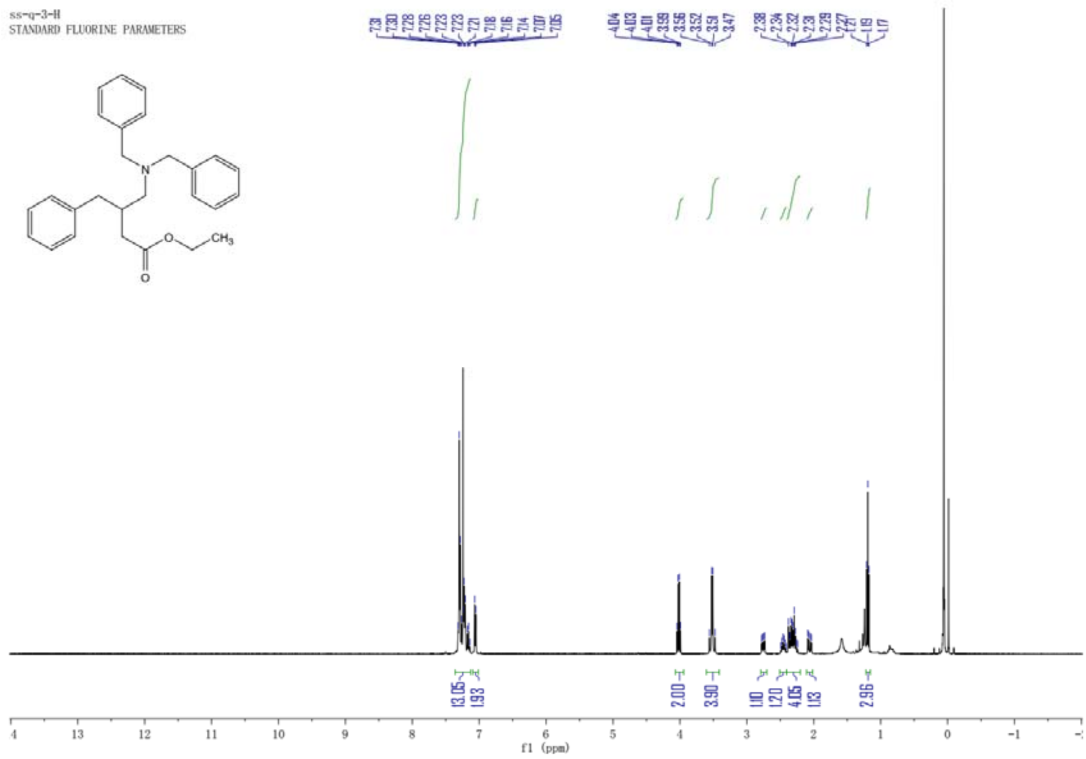
ss-q-3-H
STANDARD FLUORINE PARAMETERS
f1 (ppm)

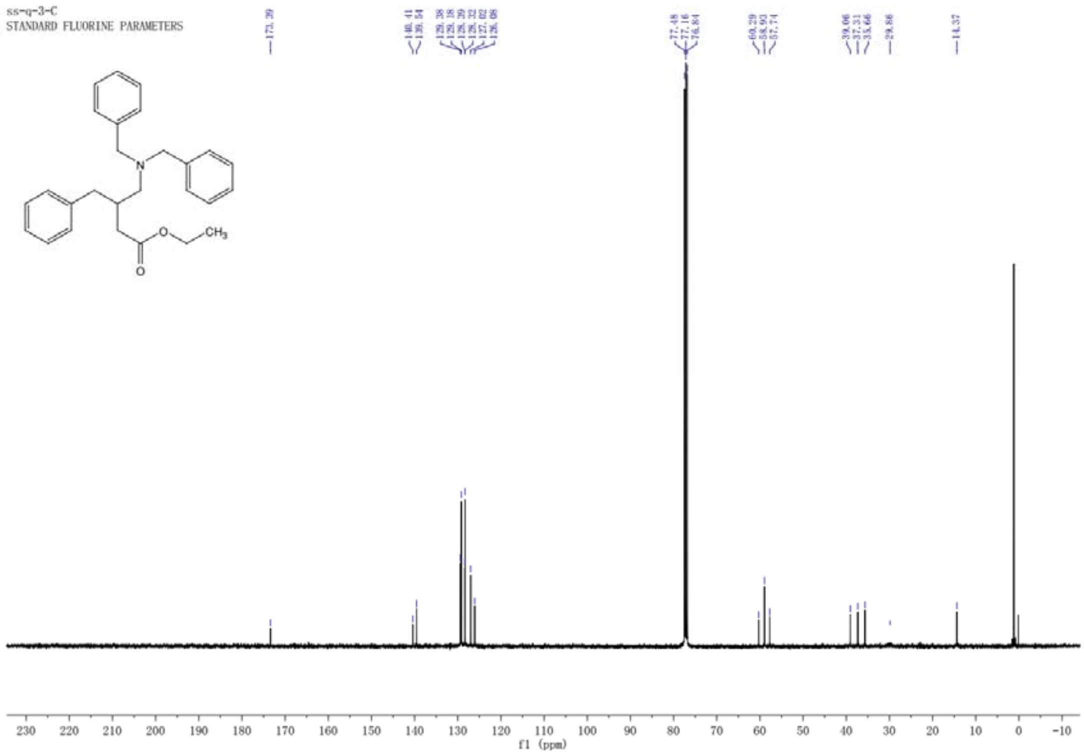
ss-q-3-C
STANDARD FLUORINE PARAMETERS
173.29
140.41
139.54
129.38
129.18
128.39
128.32
127.02
126.08
77.48
77.16
76.84
60.29
58.93
57.74
39.06
37.31
35.66
29.88
14.37
O
N
CH3
f1 (ppm)

17

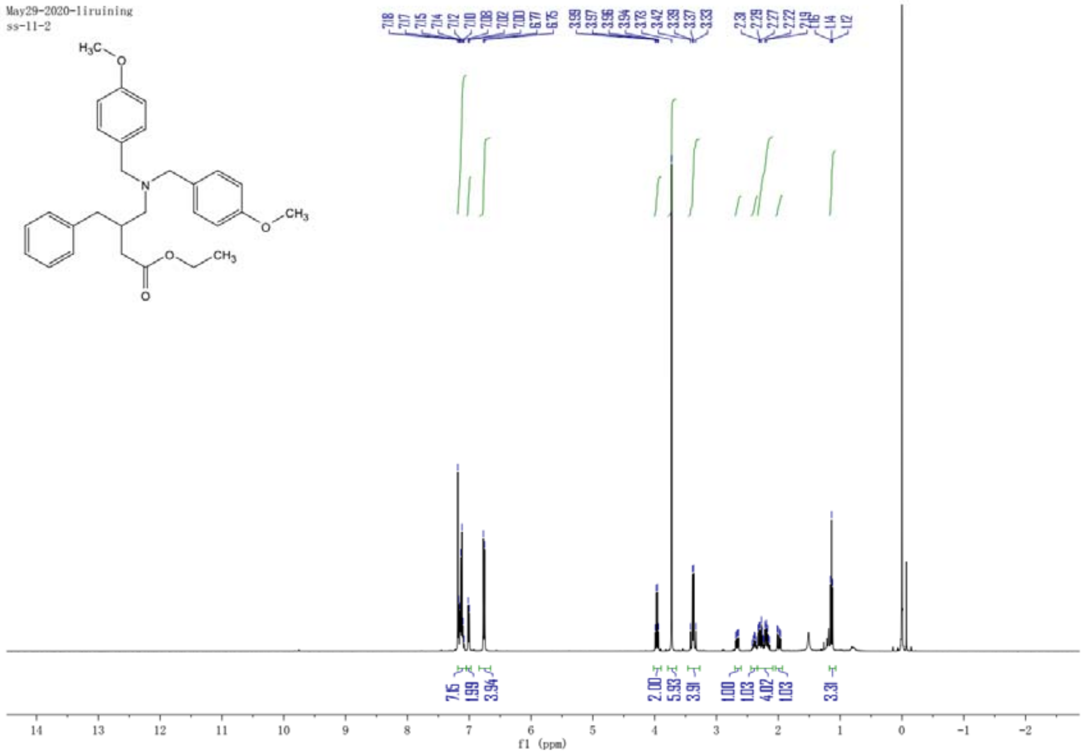
May29-2020-liruining
ss-11-2
H3C
O
N
O
CH3
CH3
7.18
7.17
7.15
7.14
7.12
7.10
7.08
7.02
7.00
6.77
6.75
3.99
3.97
3.96
3.94
3.73
3.42
3.39
3.37
3.33
2.31
2.29
2.27
2.22
2.19
2.16
1.14
1.12
7.15
1.99
3.94
2.00
5.93
3.91
1.00
1.03
4.02
1.03
3.31
f1 (ppm)

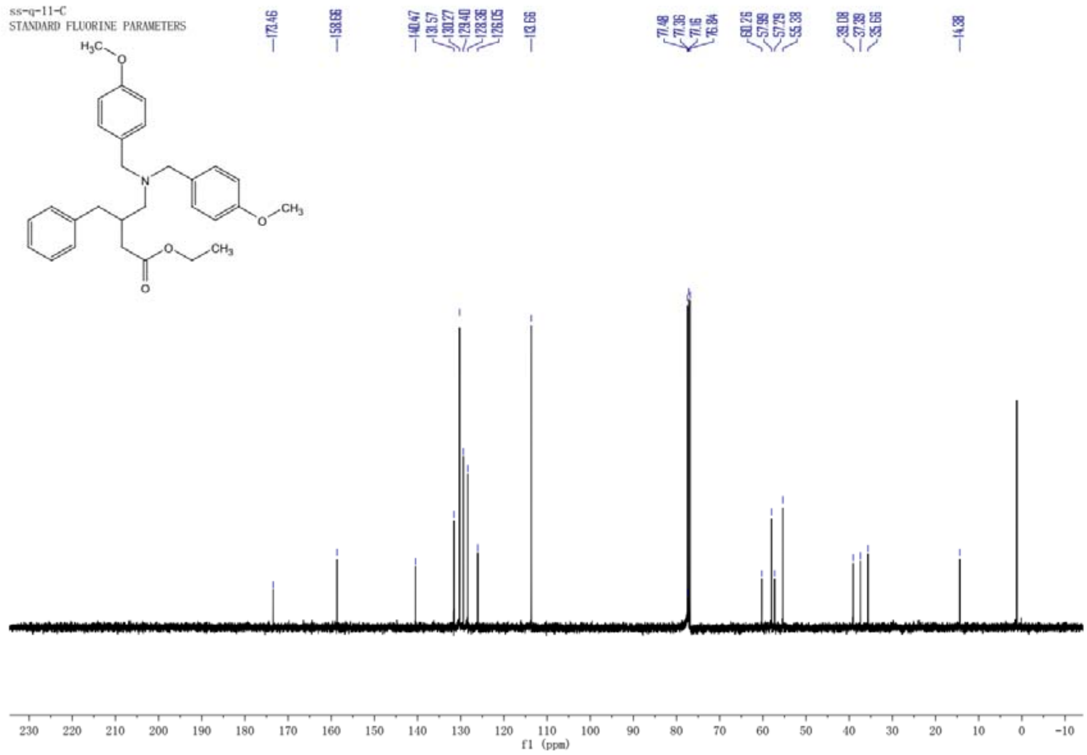

18

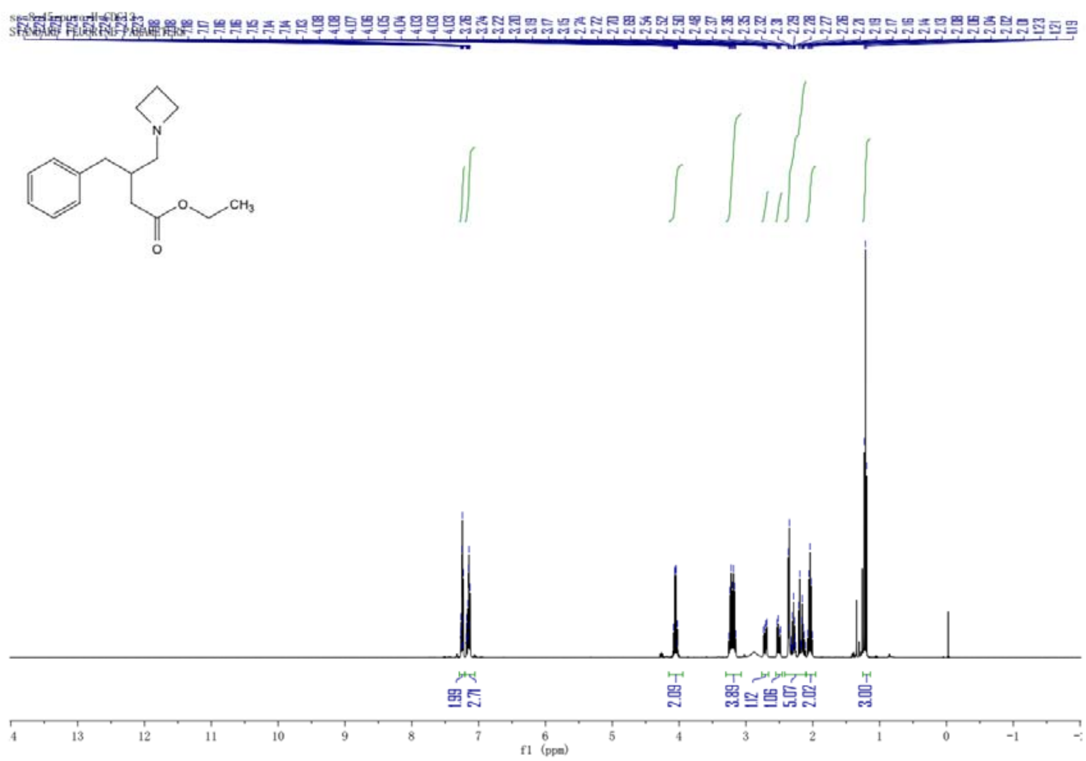

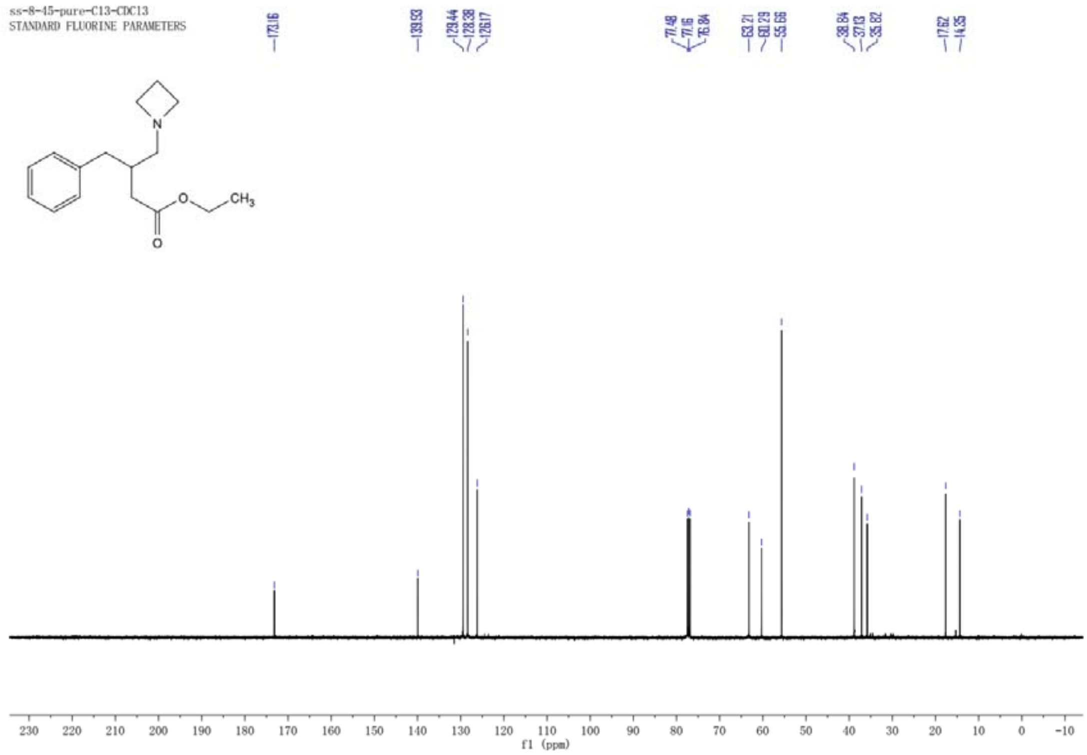
ss-8-45-pure-C13-CDCl3
STANDARD FLUORINE PARAMETERS
173.16
139.53
129.44
128.38
126.17
77.48
77.16
76.84
63.21
60.29
55.66
38.84
37.13
35.82
17.62
14.35
N
O
CH3
O
f1 (ppm)

19

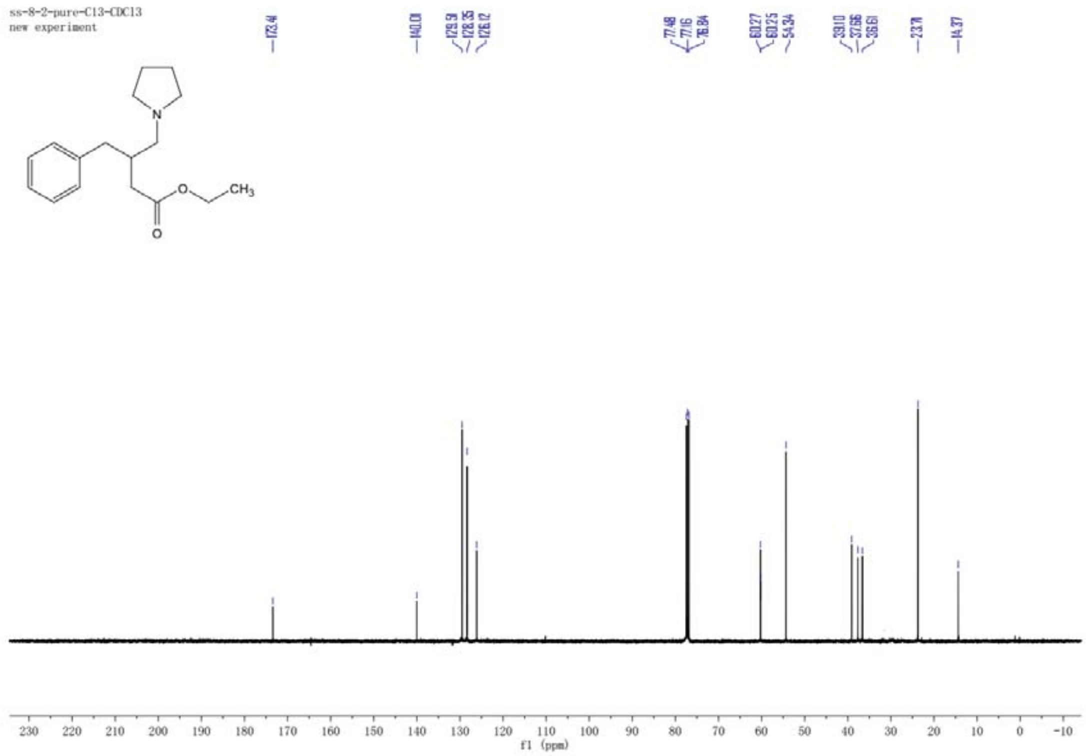
ss-8-2-pure-C13-CDCl3
new experiment
173.41
140.01
129.51
128.35
126.12
77.48
77.16
76.84
60.27
60.25
54.34
39.10
37.66
36.61
23.71
14.37
N
O
CH3
O
f1 (ppm)

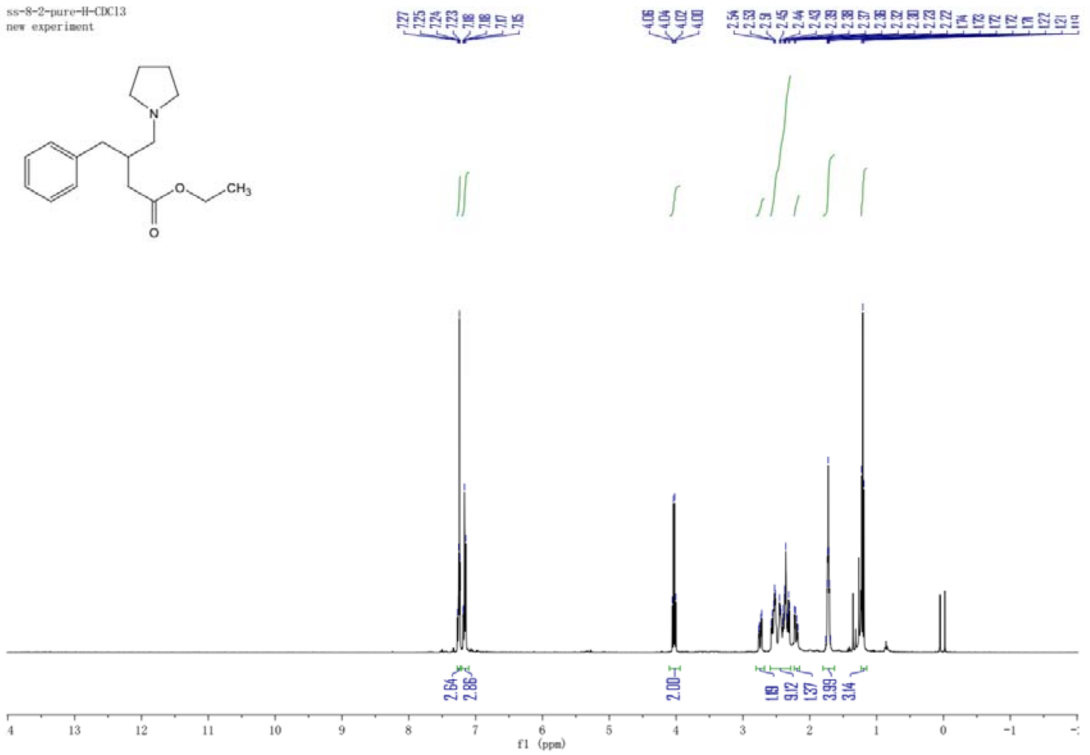

20

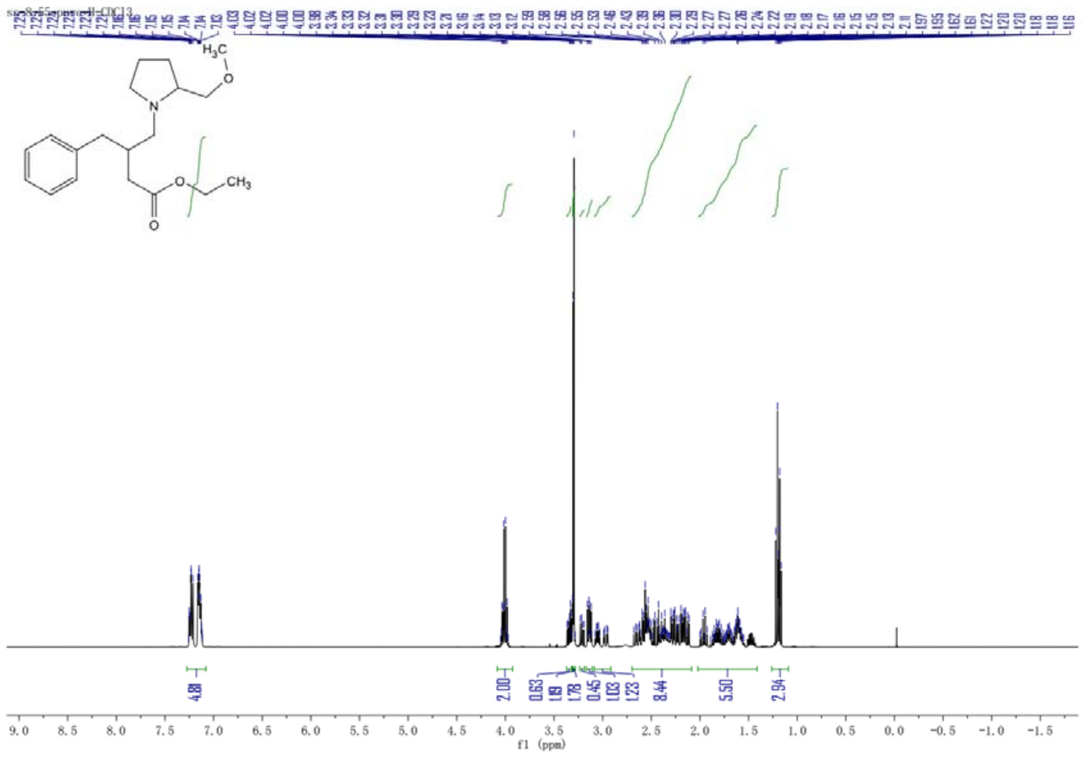

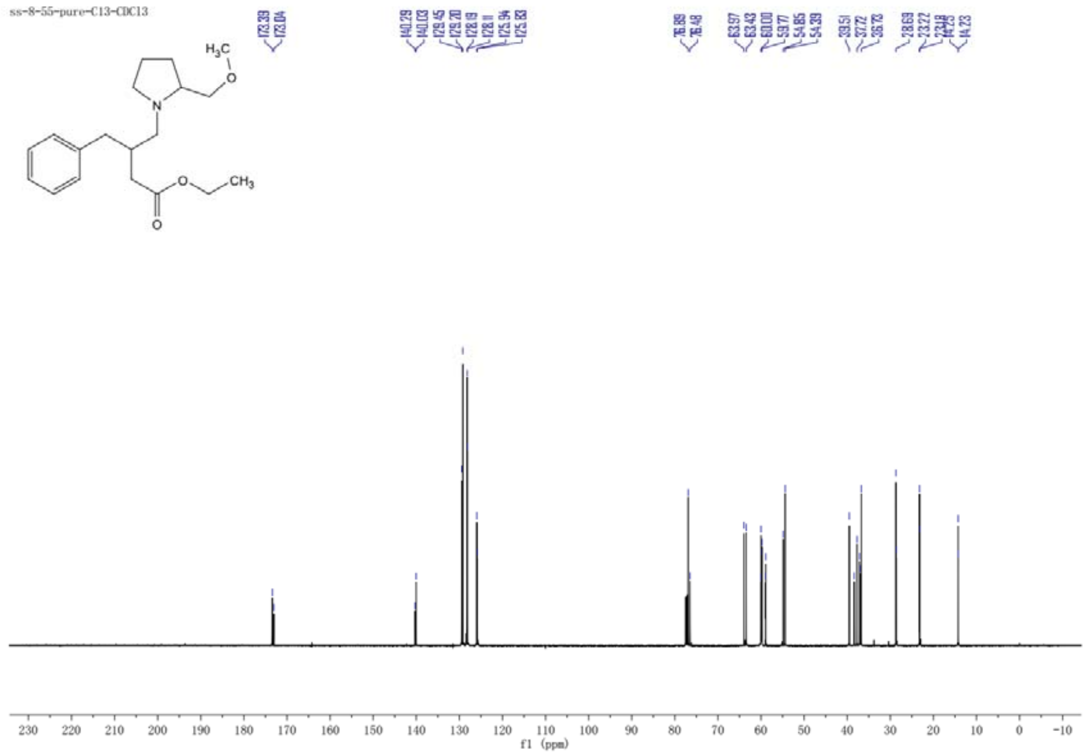

21

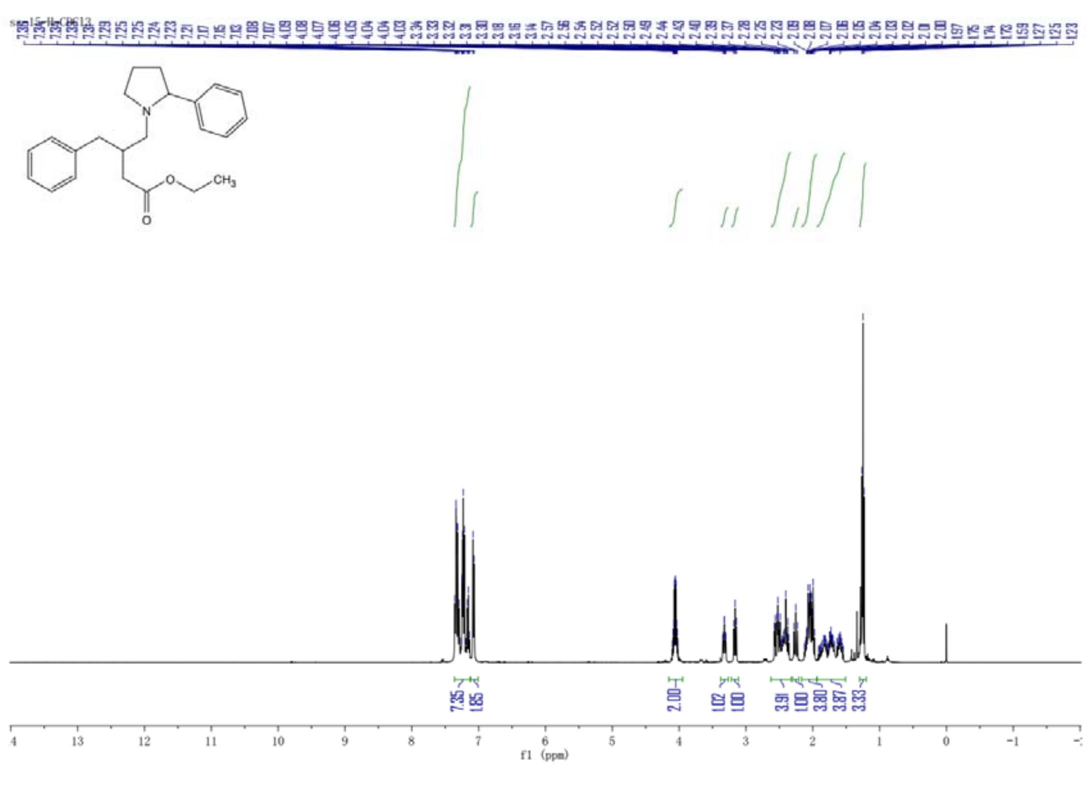

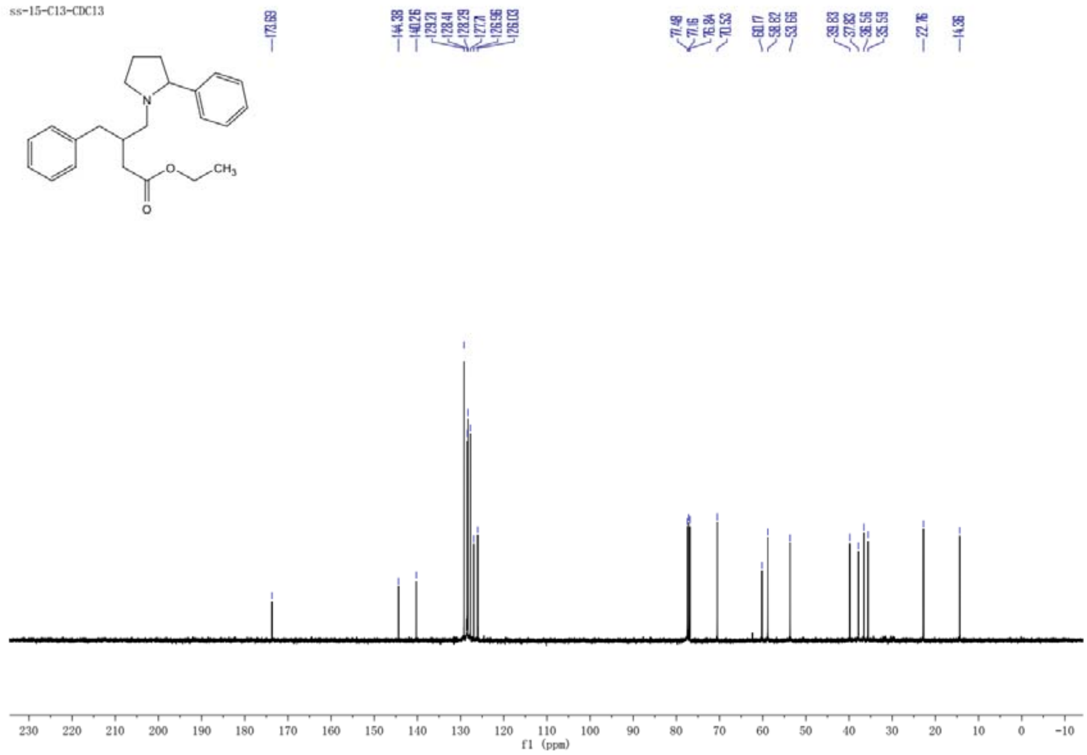

22

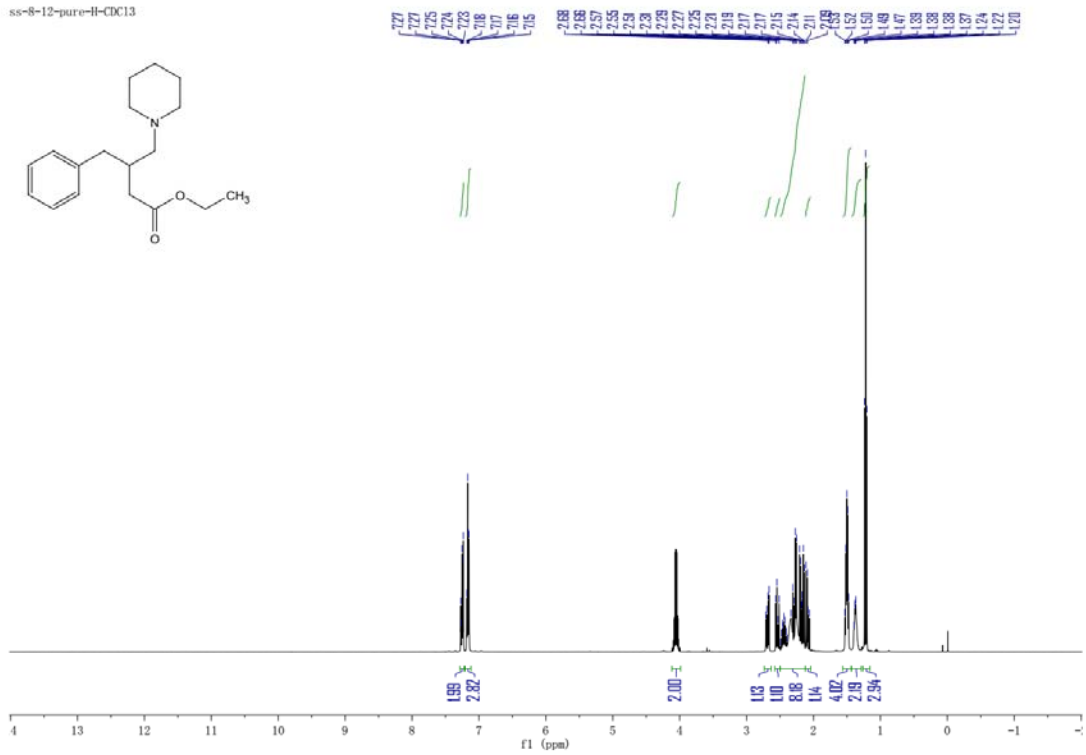

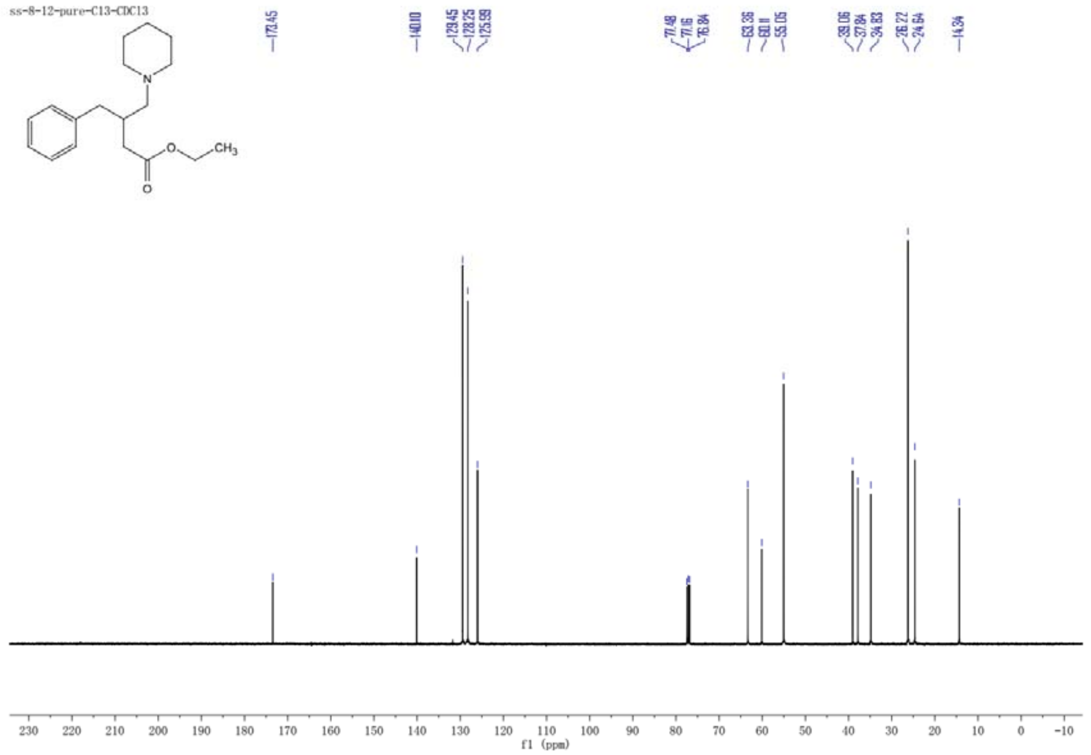

23

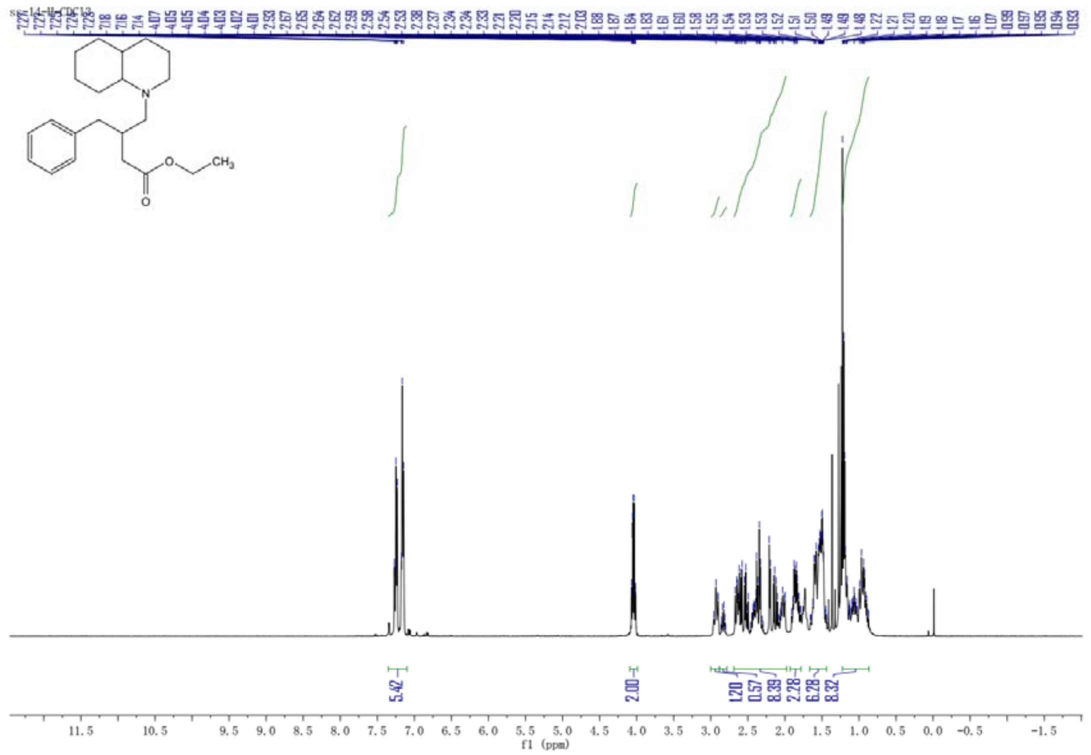

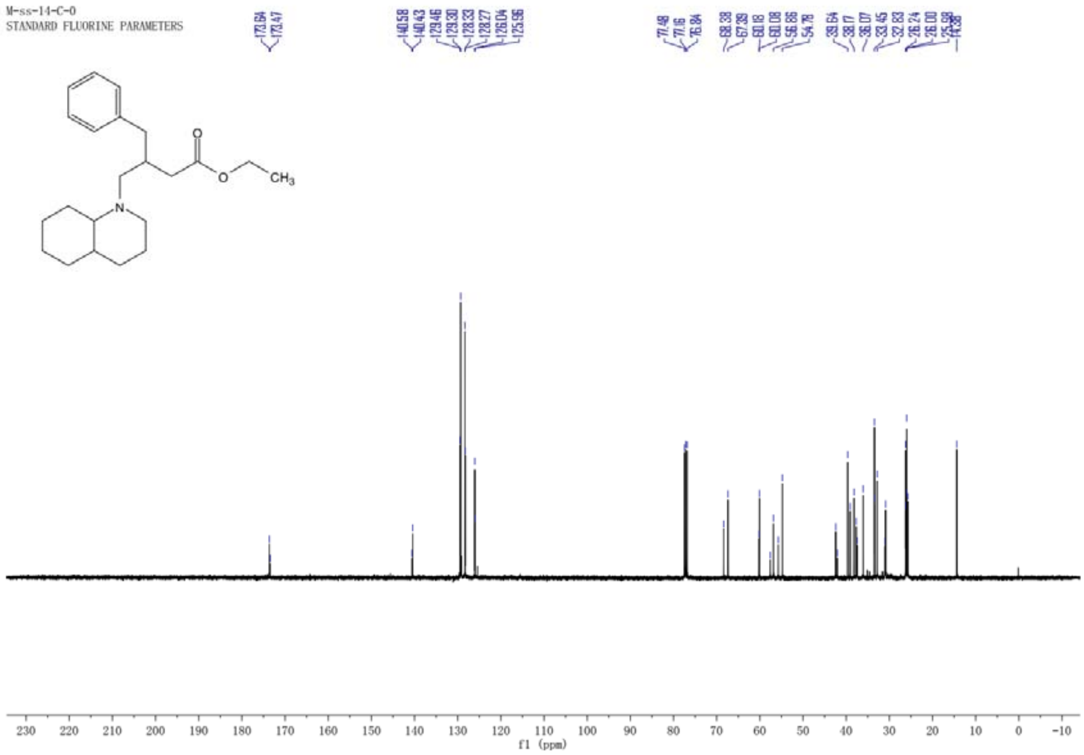
M-ss-14-C-0
STANDARD FLUORINE PARAMETERS
CH3
N
O
f1 (ppm)

24

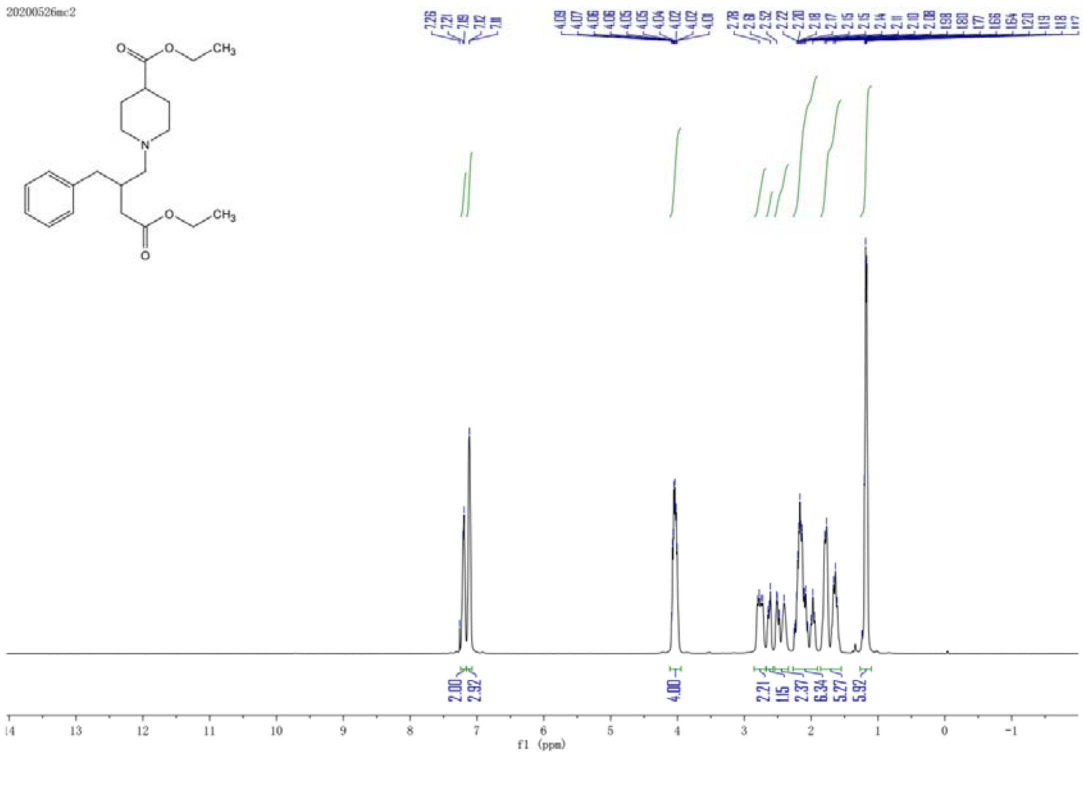
20200526mc2
CH3
N
O
f1 (ppm)

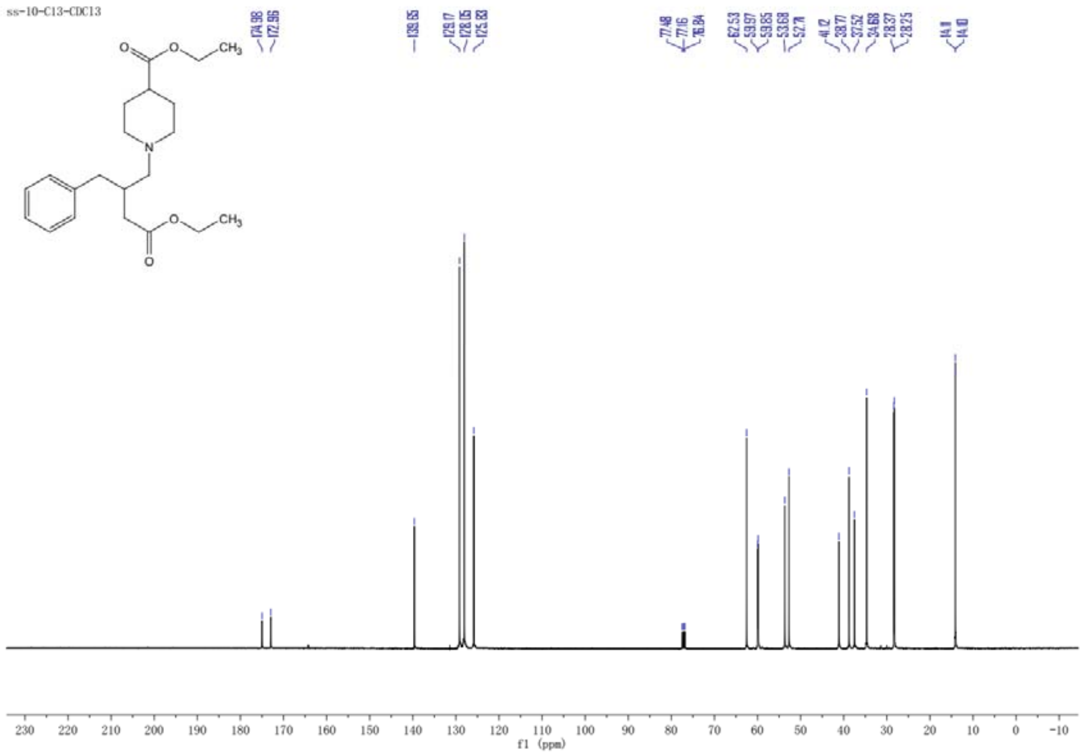
ss-10-C13-CDCl3
174.98
172.96
139.65
129.17
128.15
125.83
77.48
77.16
76.84
62.53
59.97
59.85
53.68
52.71
41.12
38.77
37.52
34.68
28.37
28.25
14.11
14.10
f1 (ppm)

25

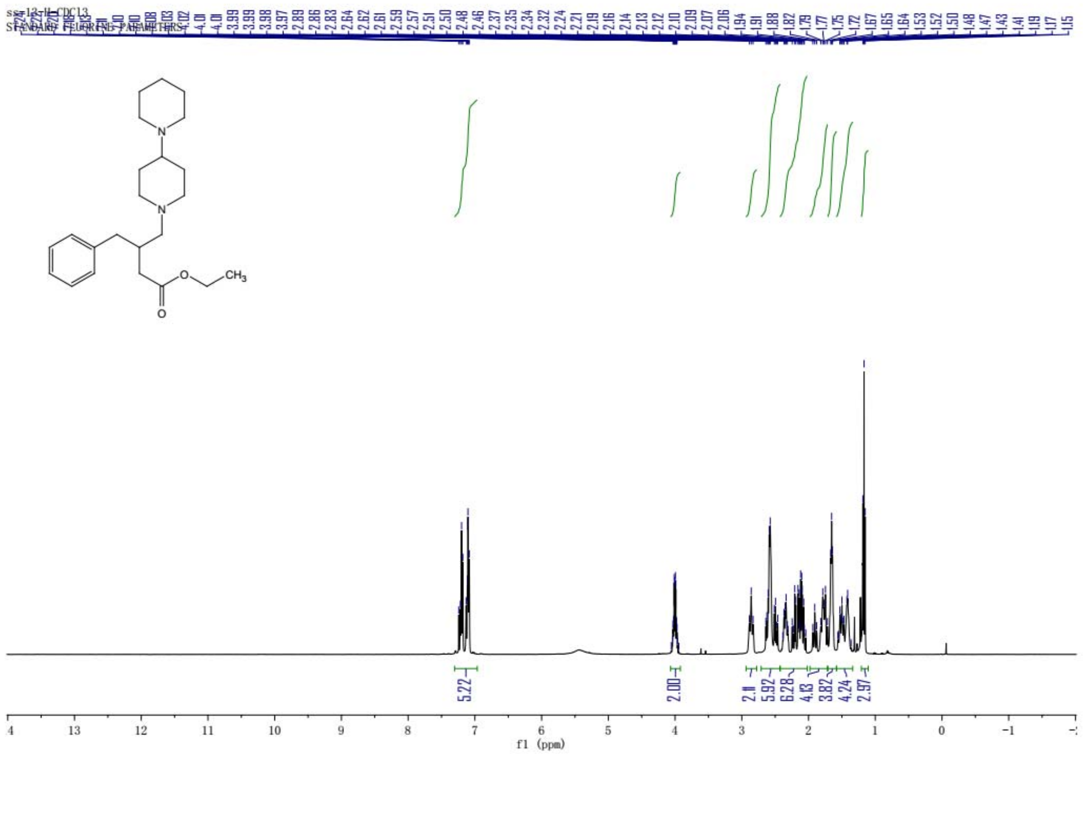
5.22
2.00
2.11
5.92
6.28
4.13
3.82
4.24
2.97
f1 (ppm)

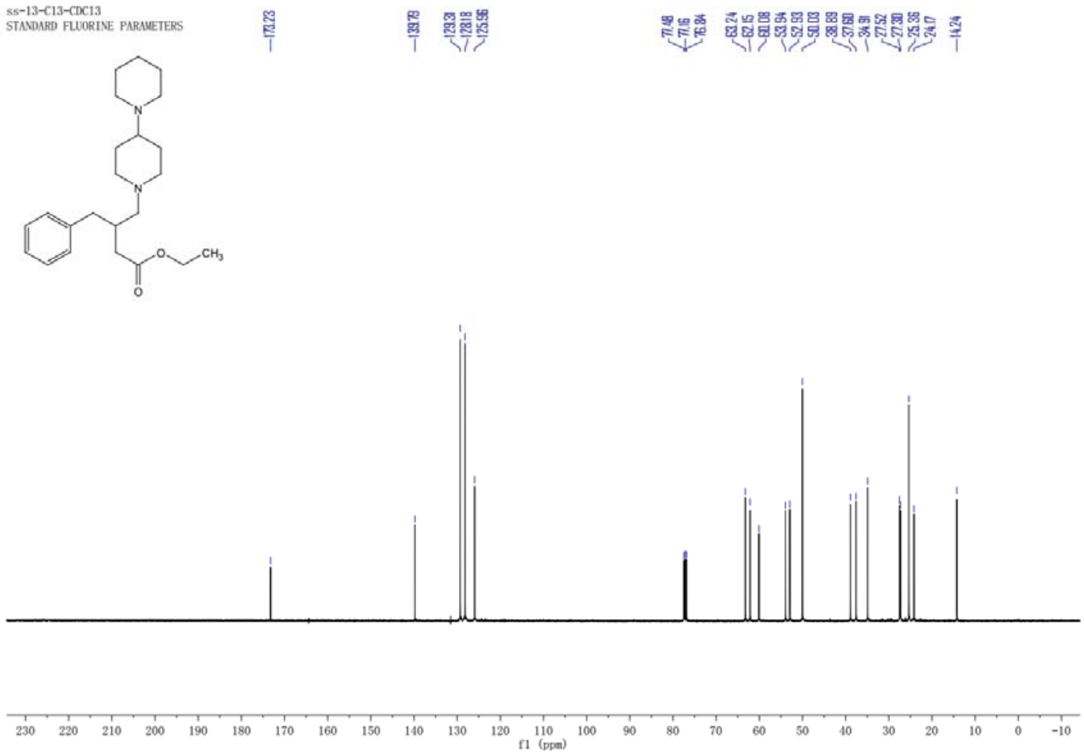
ss-13-C13-CDCl3
STANDARD FLUORINE PARAMETERS
f1 (ppm)

26

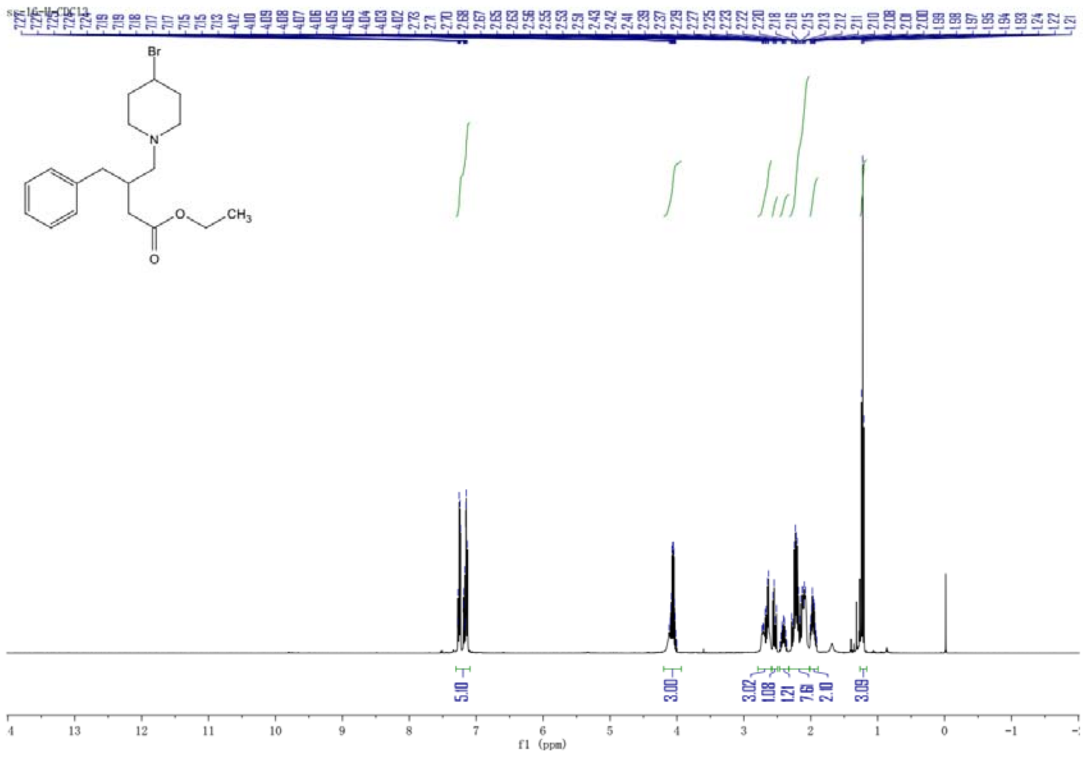
Br
f1 (ppm)

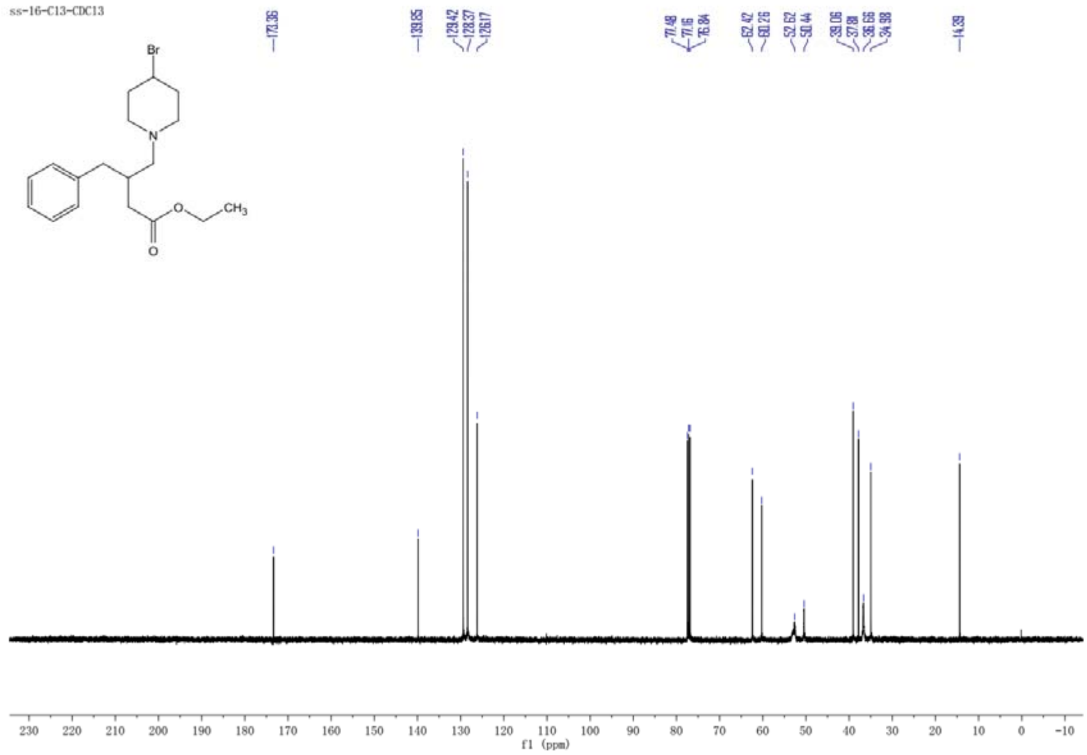
ss-16-C13-CDCl3
173.36
139.85
129.42
128.37
126.17
77.48
77.16
76.84
62.42
60.26
52.62
50.44
39.06
37.81
36.66
34.98
14.39
Br
N
O
CH3
f1 (ppm)

27

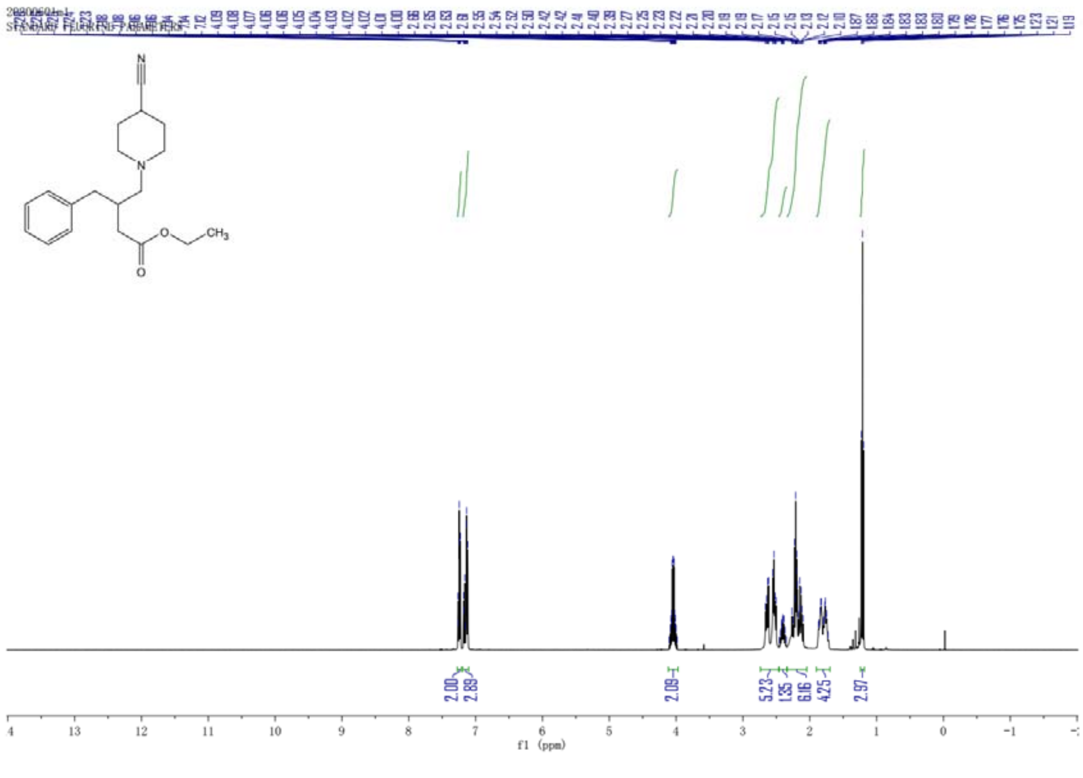
N
O
CH3
2.00
2.89
2.09
5.23
1.35
6.16
4.25
2.97
f1 (ppm)

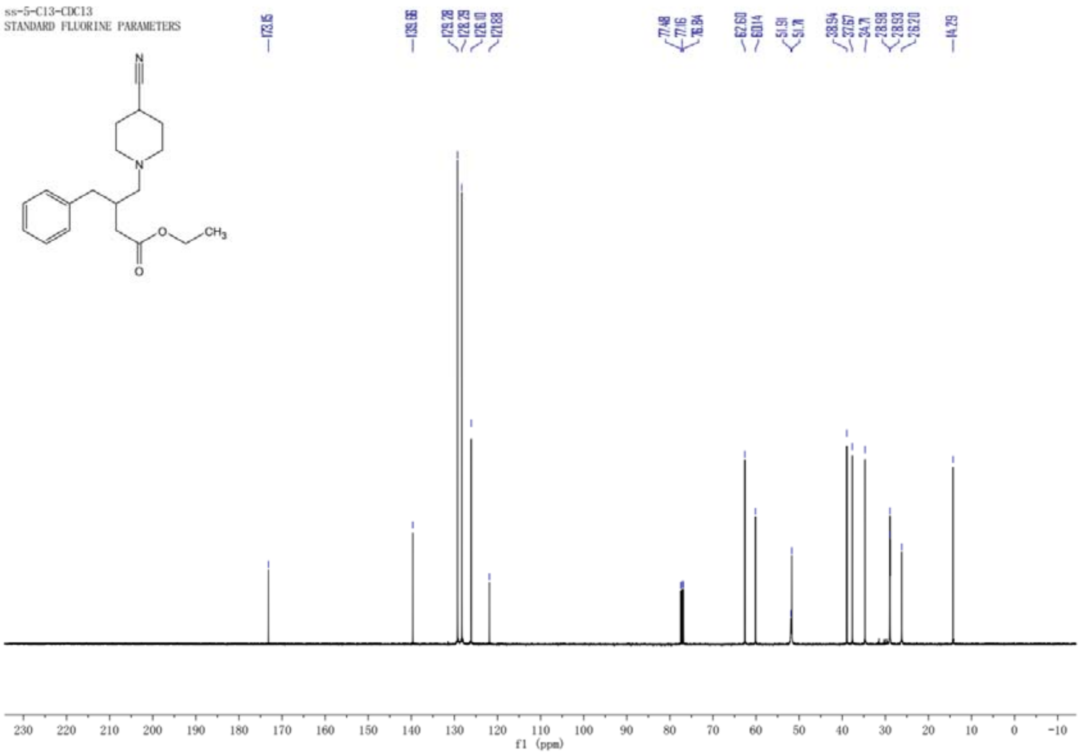
ss-5-C13-CDCl3
STANDARD FLUORINE PARAMETERS
f1 (ppm)

28

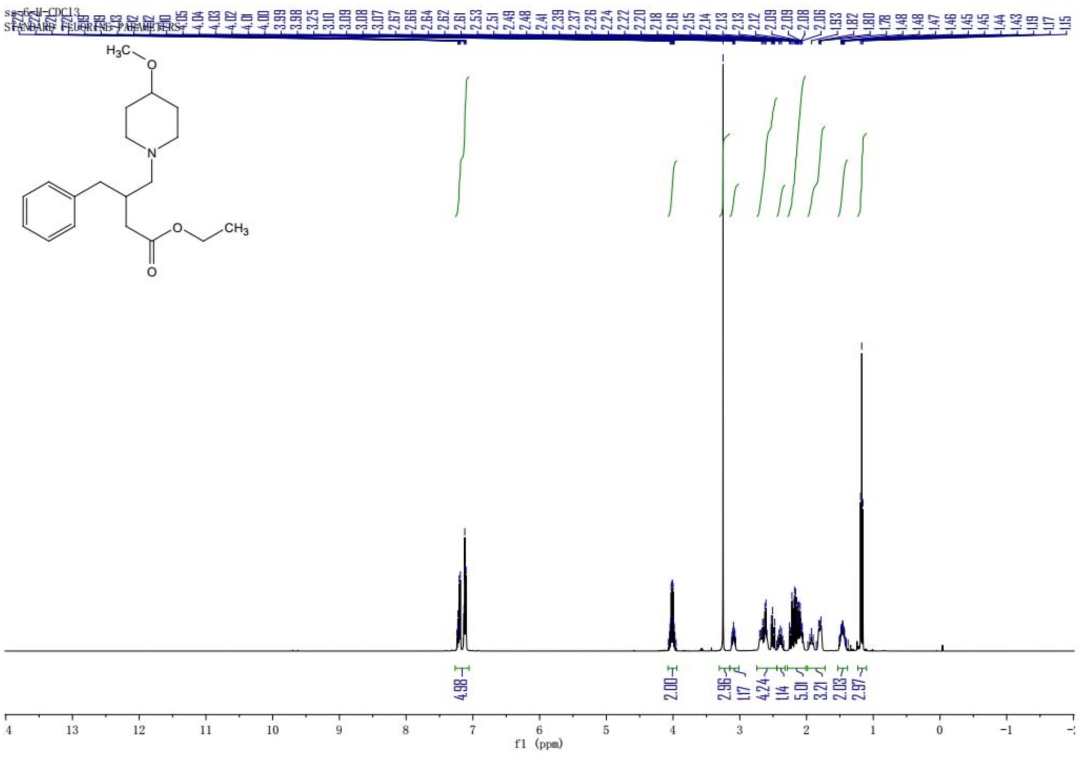
STANDARD FLUORINE PARAMETERS
f1 (ppm)

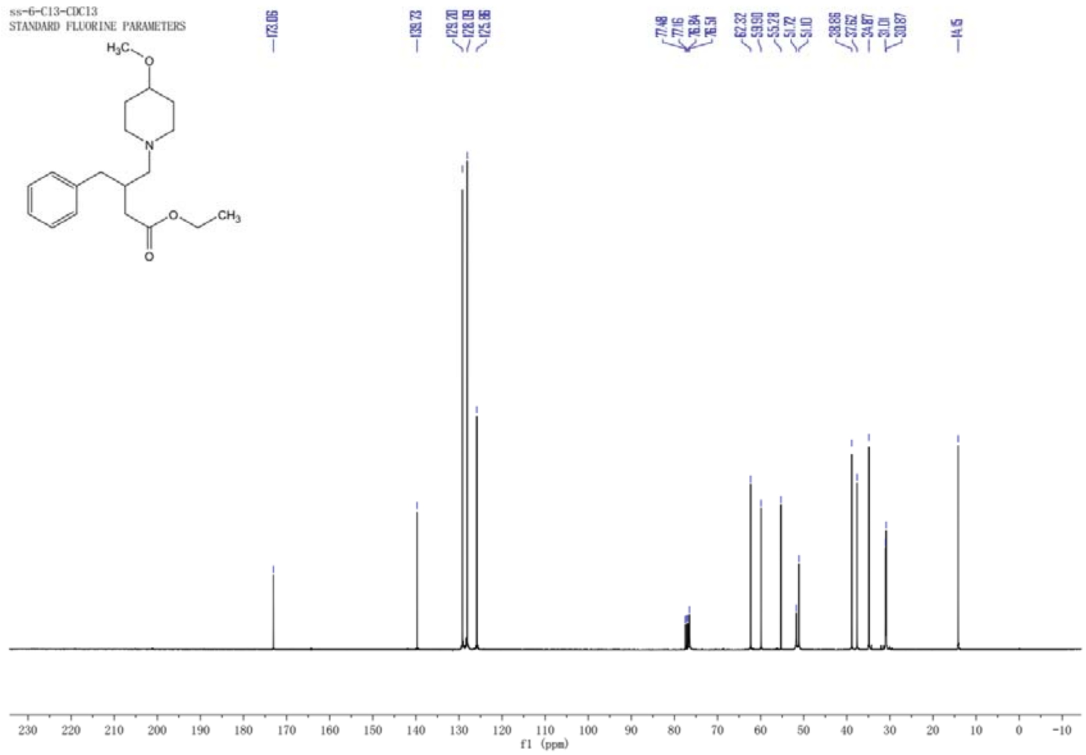
ss-6-C13-CDCl3
STANDARD FLUORINE PARAMETERS
f1 (ppm)

29

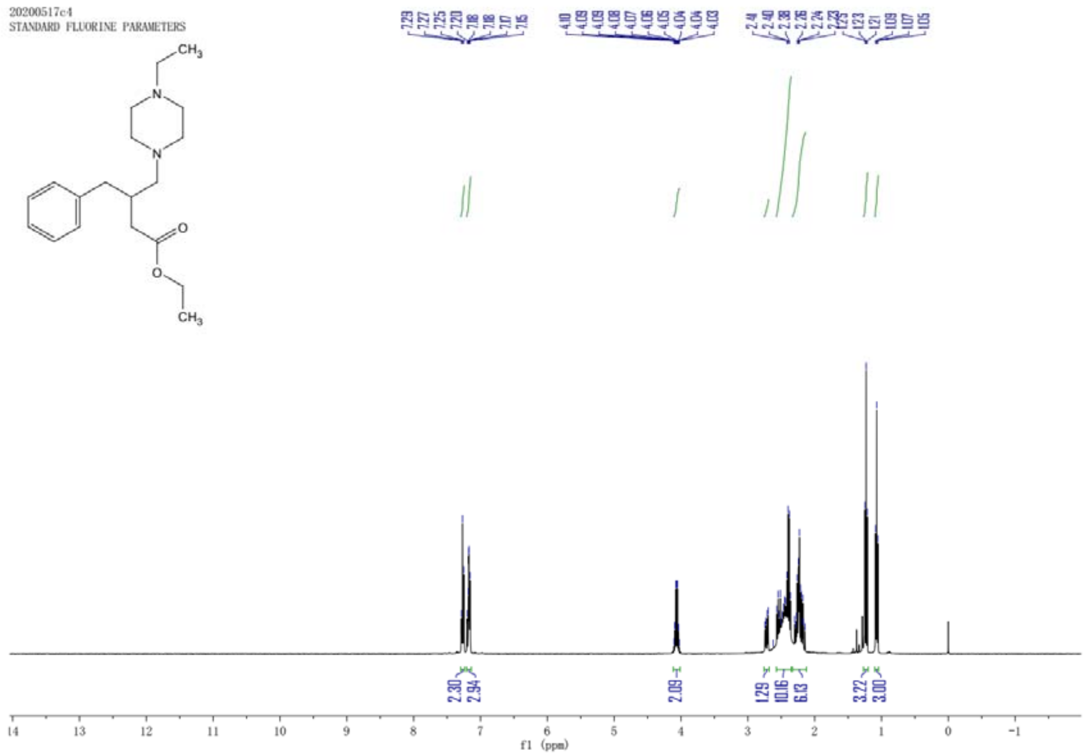
20200517c4
STANDARD FLUORINE PARAMETERS
f1 (ppm)

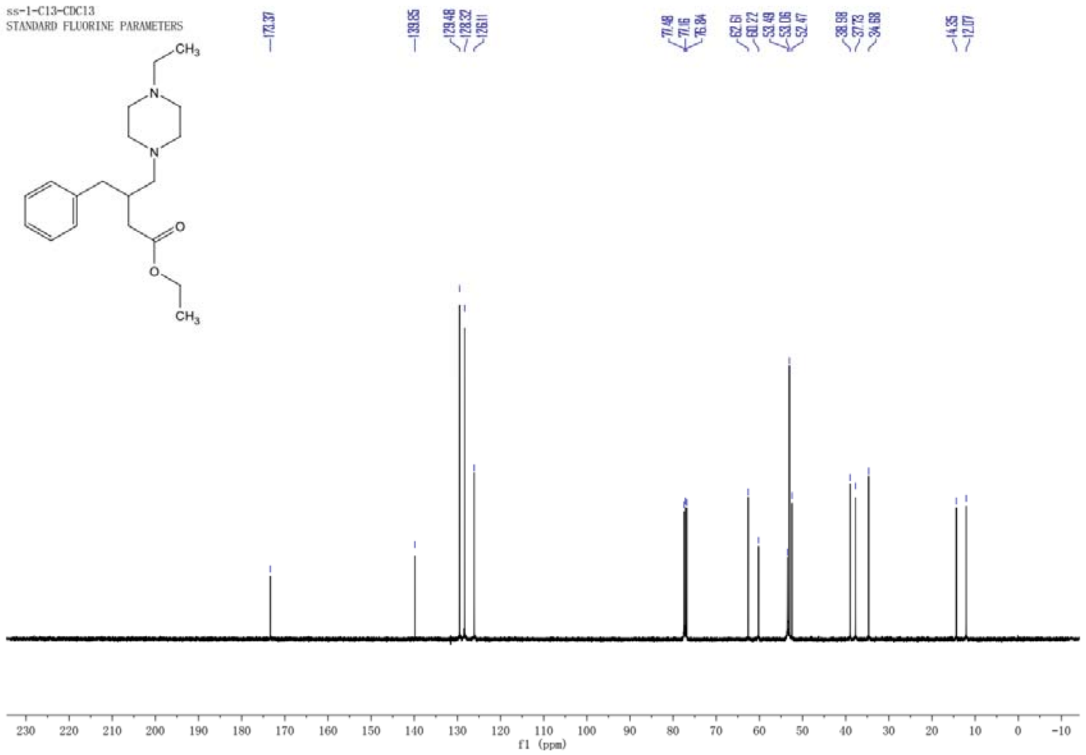

30

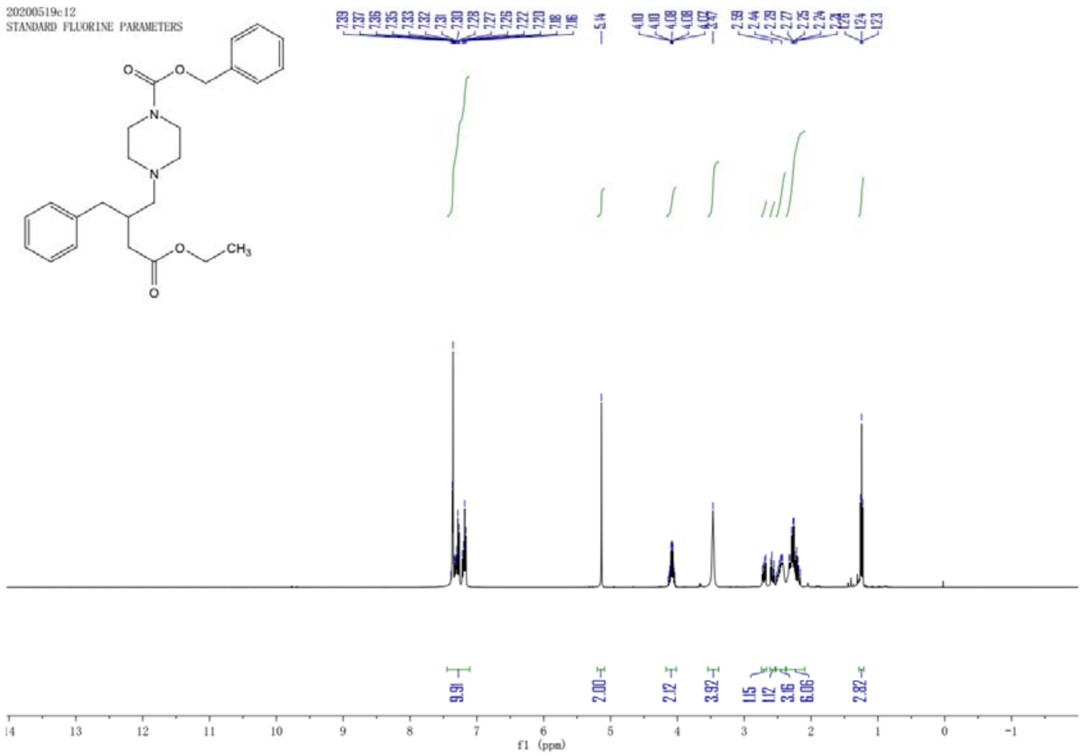

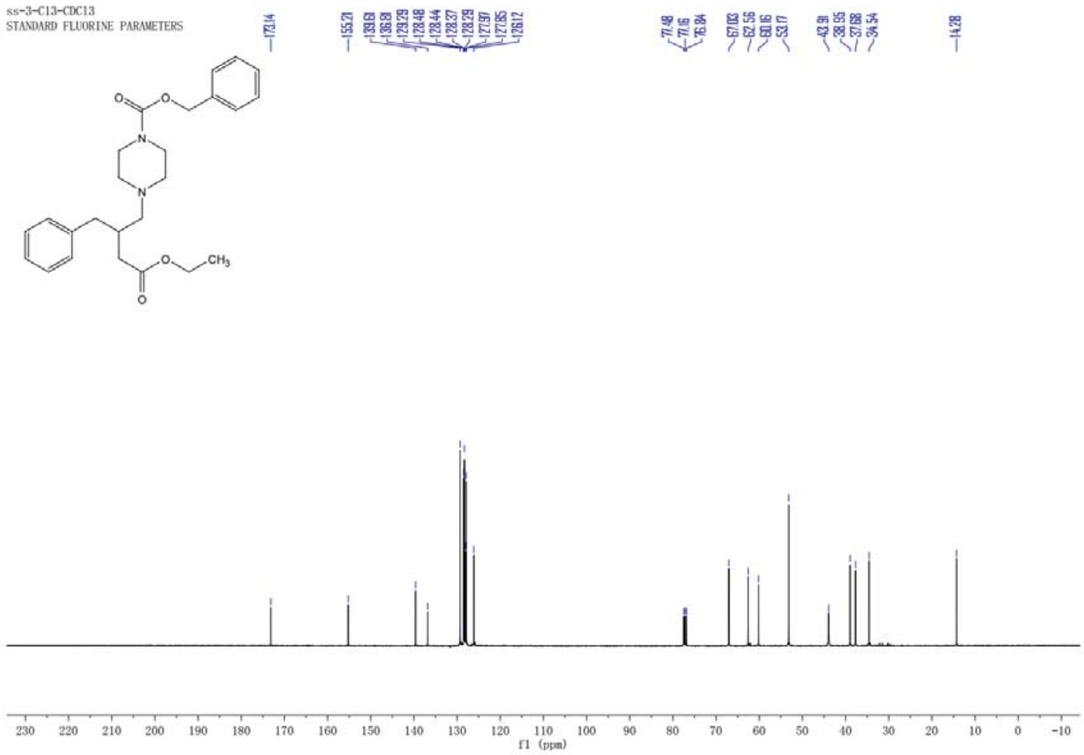
ss-3-C13-CDCl3
STANDARD FLUORINE PARAMETERS
f1 (ppm)

31

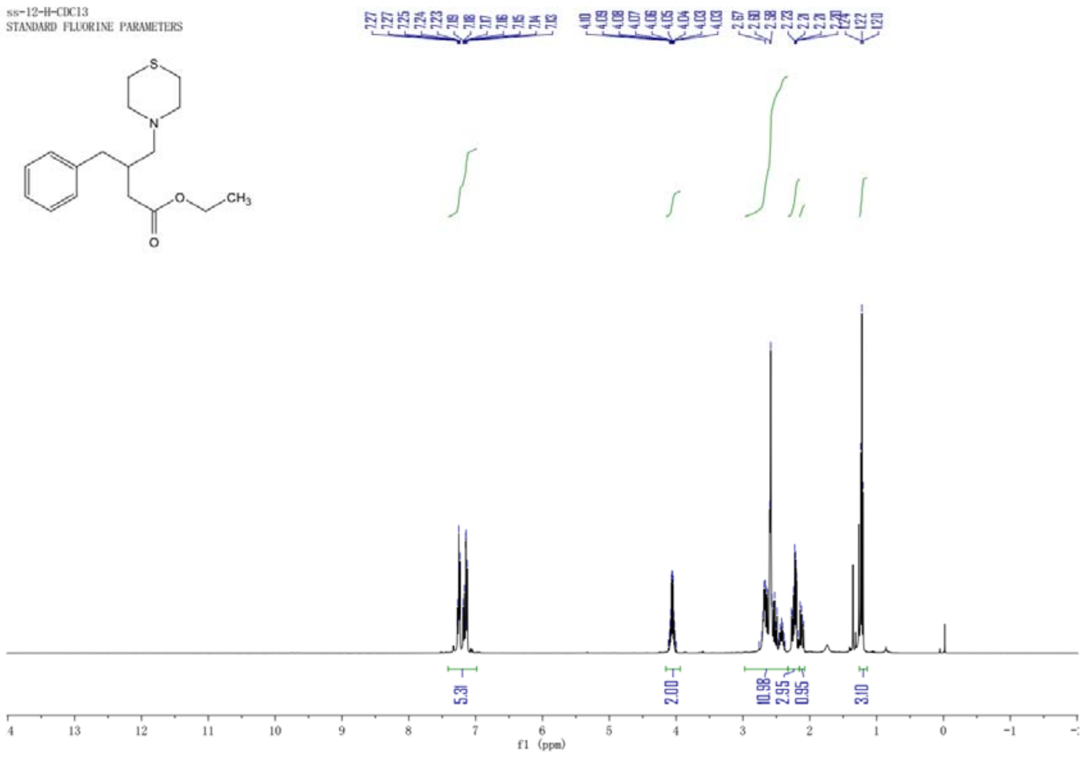
ss-12-H-CDCl3
STANDARD FLUORINE PARAMETERS
f1 (ppm)

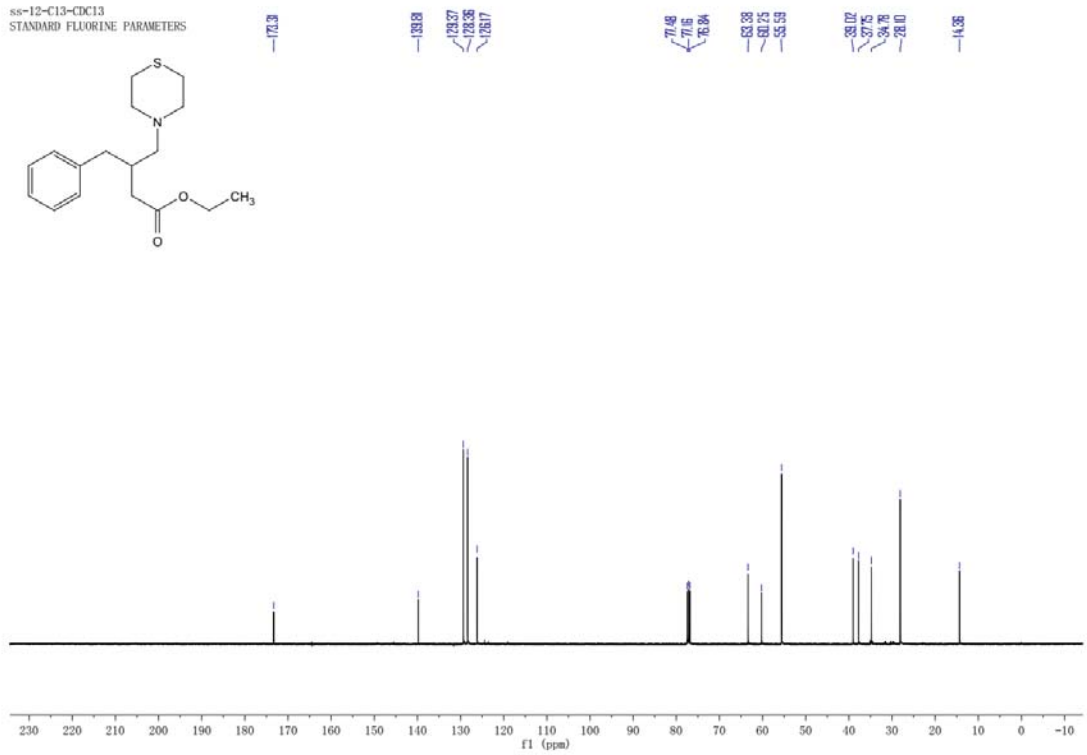
ss-12-C13-CDCl3
STANDARD FLUORINE PARAMETERS
173.31
139.81
129.37
128.36
126.17
77.48
77.16
76.84
63.38
60.25
55.59
39.02
37.75
34.78
28.10
14.36
f1 (ppm)

32

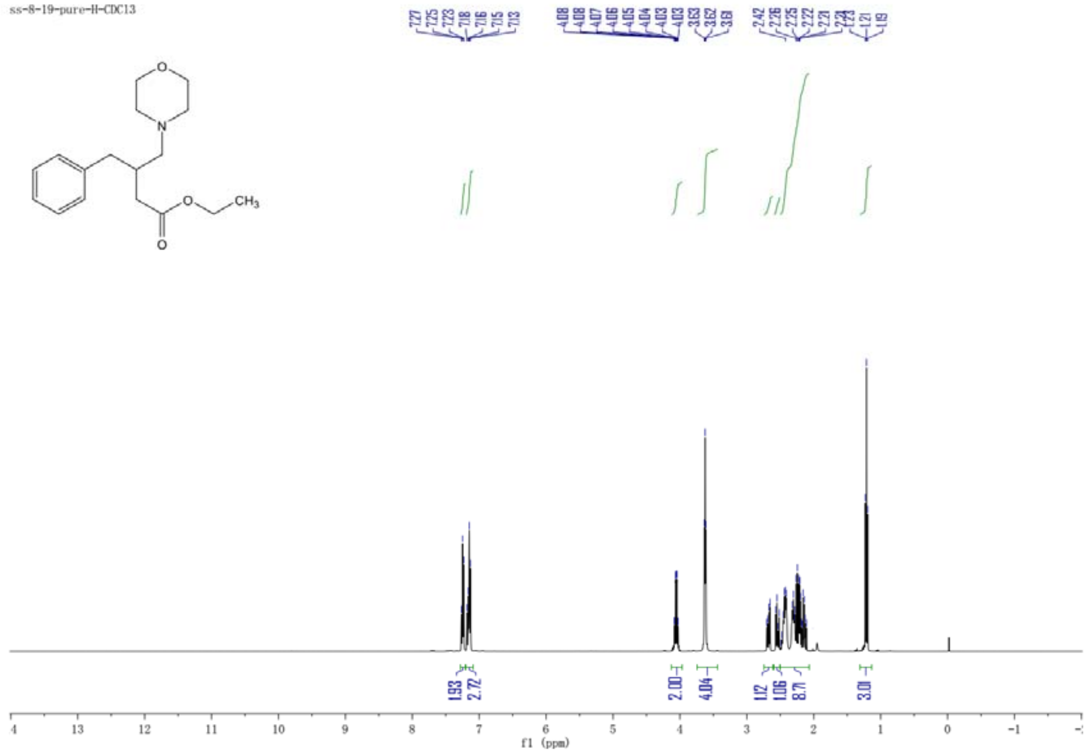
ss-8-19-pure-H-CDCl3
1.93
2.72
2.00
4.04
1.12
1.06
8.71
3.01
f1 (ppm)

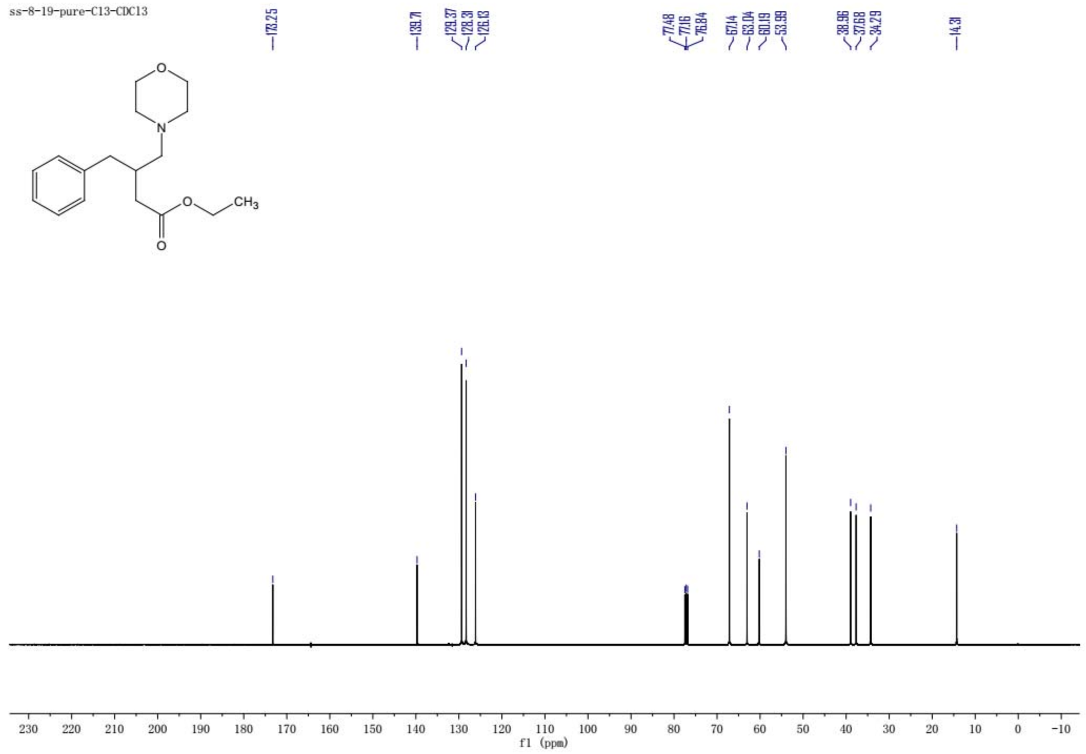
ss-8-19-pure-C13-CDCl3
173.25
139.71
129.37
128.31
126.13
77.48
77.16
76.84
67.14
63.04
60.19
53.99
38.96
37.68
34.29
14.31
f1 (ppm)

33

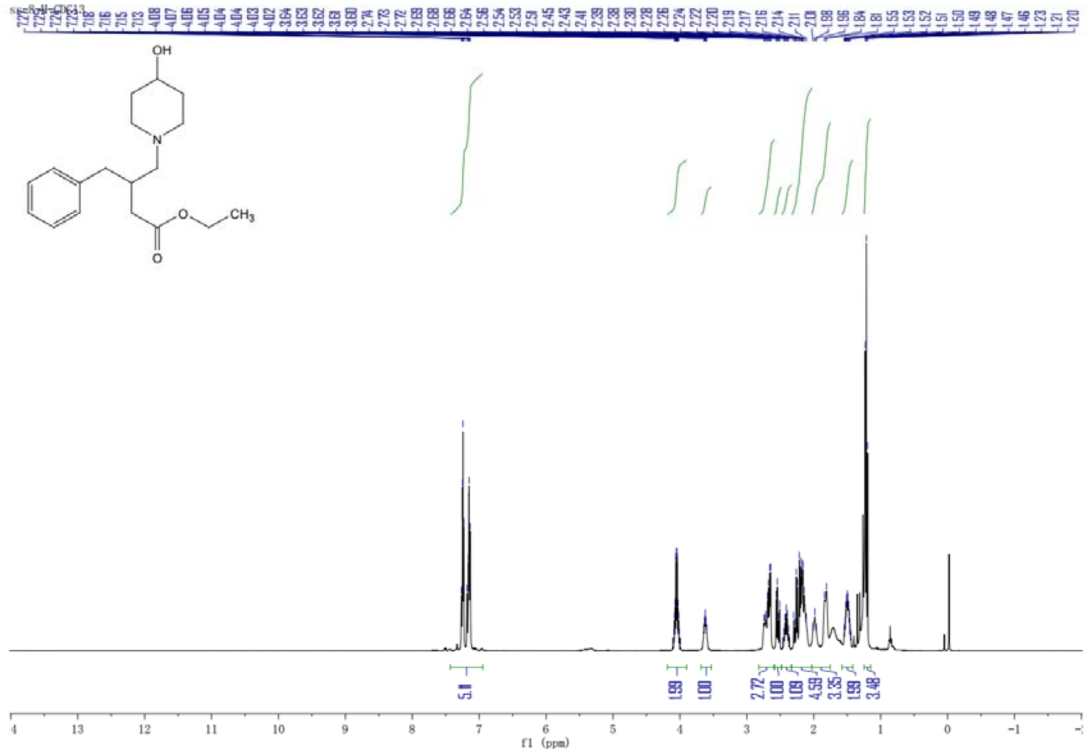
OH
N
O
CH3
5.11
1.99
1.00
2.72
1.00
1.09
4.59
3.35
1.99
3.49
f1 (ppm)

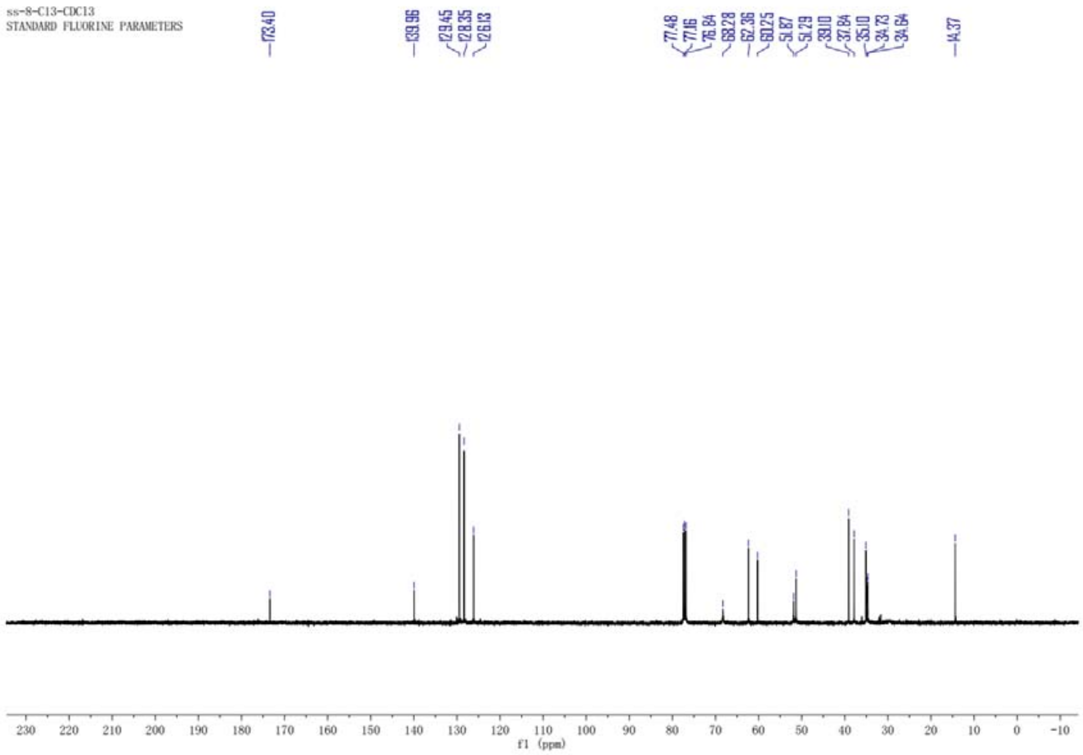
ss-8-C13-CDCl3
STANDARD FLUORINE PARAMETERS
173.40
139.96
129.45
128.35
126.13
77.48
77.16
76.84
68.28
62.36
60.25
51.87
51.29
39.10
37.84
35.10
34.73
34.64
14.37
f1 (ppm)

34

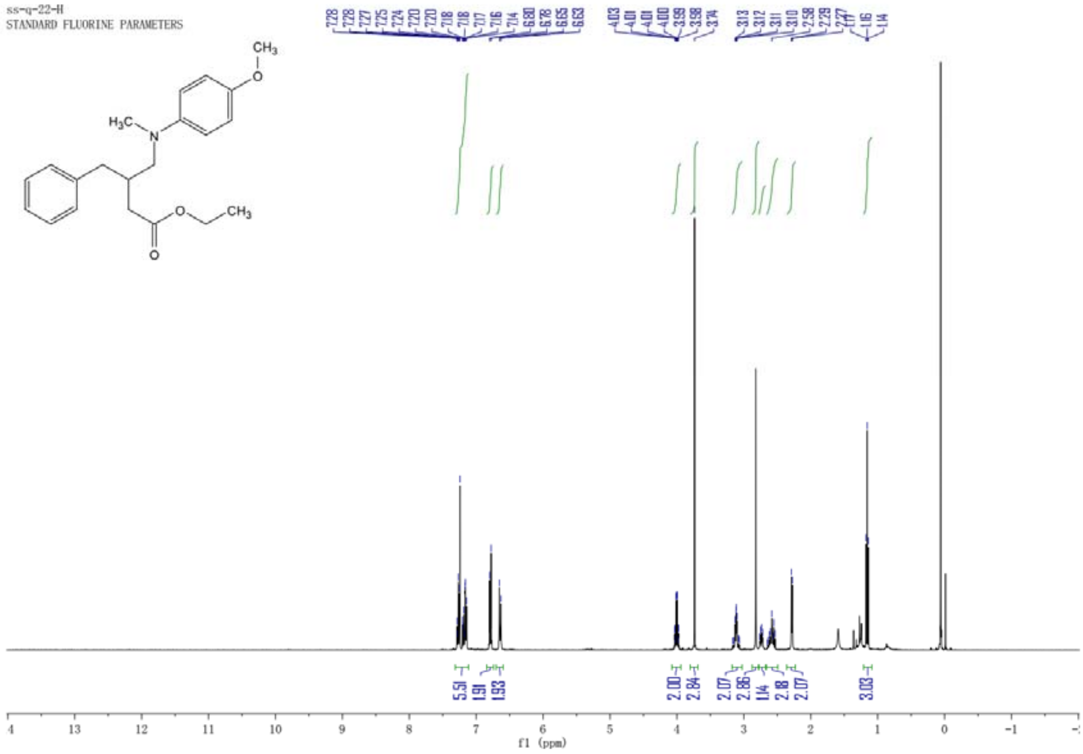
ss-q-22-H
STANDARD FLUORINE PARAMETERS
CH3
O
H3C
N
O
O
CH3
5.51
1.91
1.93
2.00
2.84
2.07
2.86
1.14
2.18
2.07
3.03
f1 (ppm)

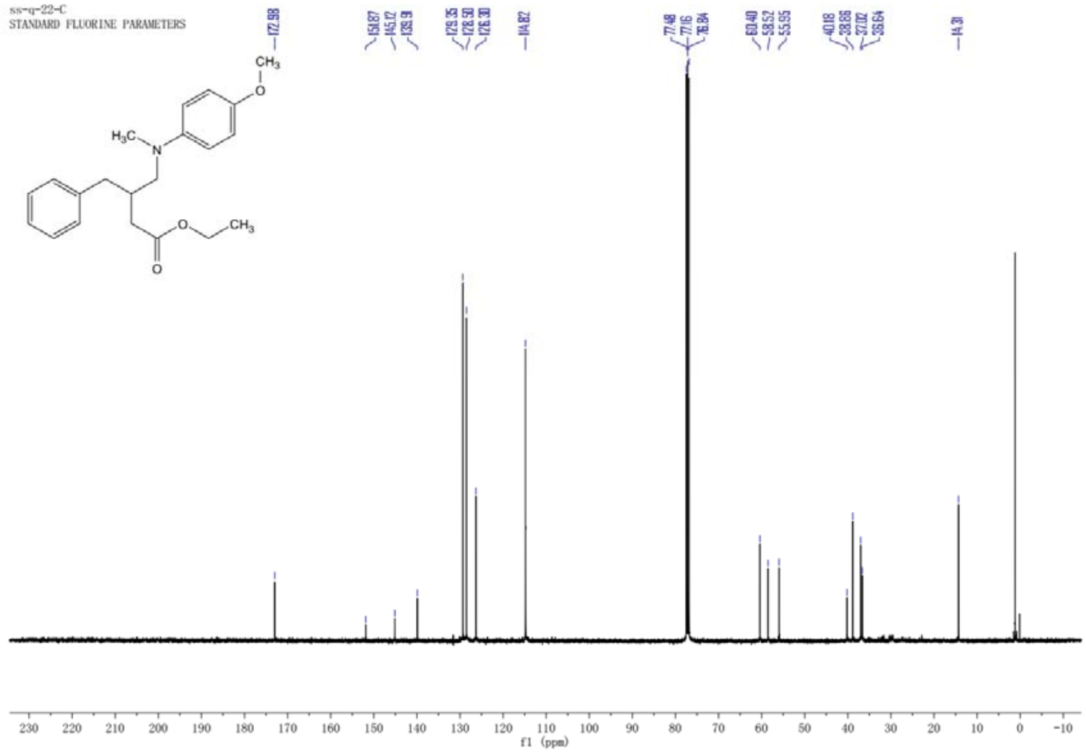
ss-q-22-C
STANDARD FLUORINE PARAMETERS
172.98
151.87
145.12
139.91
129.35
128.50
126.30
114.82
77.48
77.16
76.84
60.40
58.52
55.95
40.18
38.86
37.02
36.64
14.31
f1 (ppm)

35

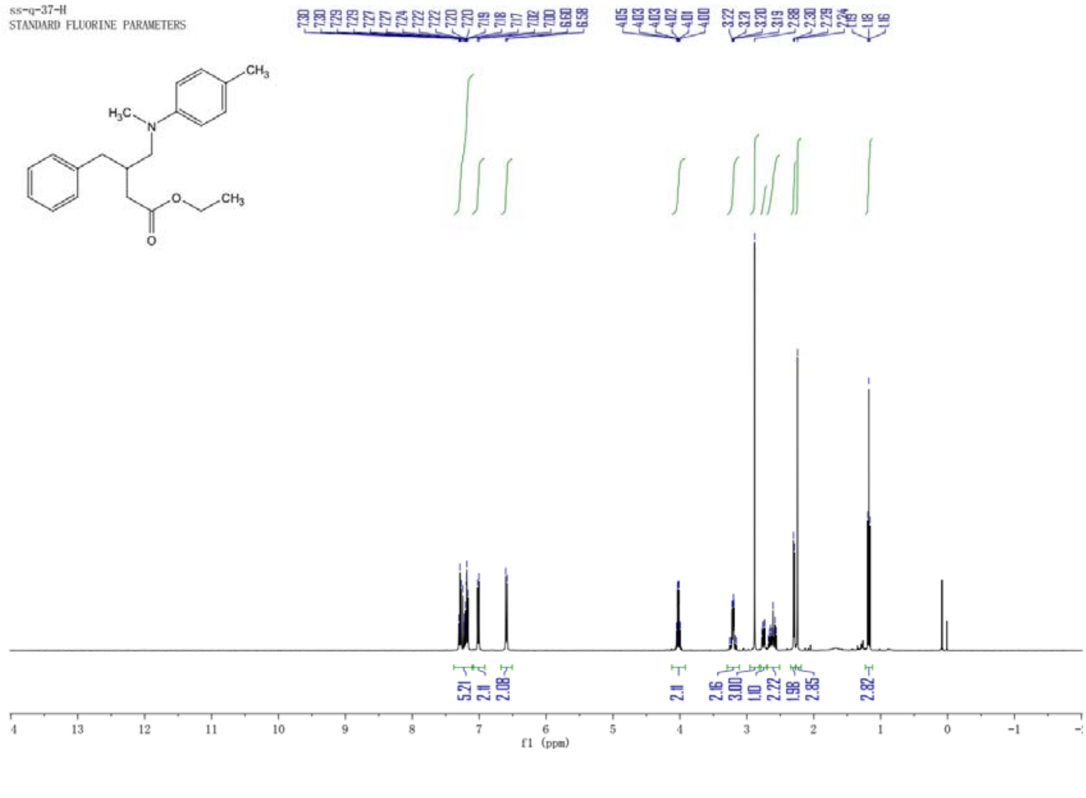
ss-q-37-H
STANDARD FLUORINE PARAMETERS
5.21
2.11
2.08
2.11
2.16
3.00
1.10
2.22
1.98
2.85
2.82
f1 (ppm)

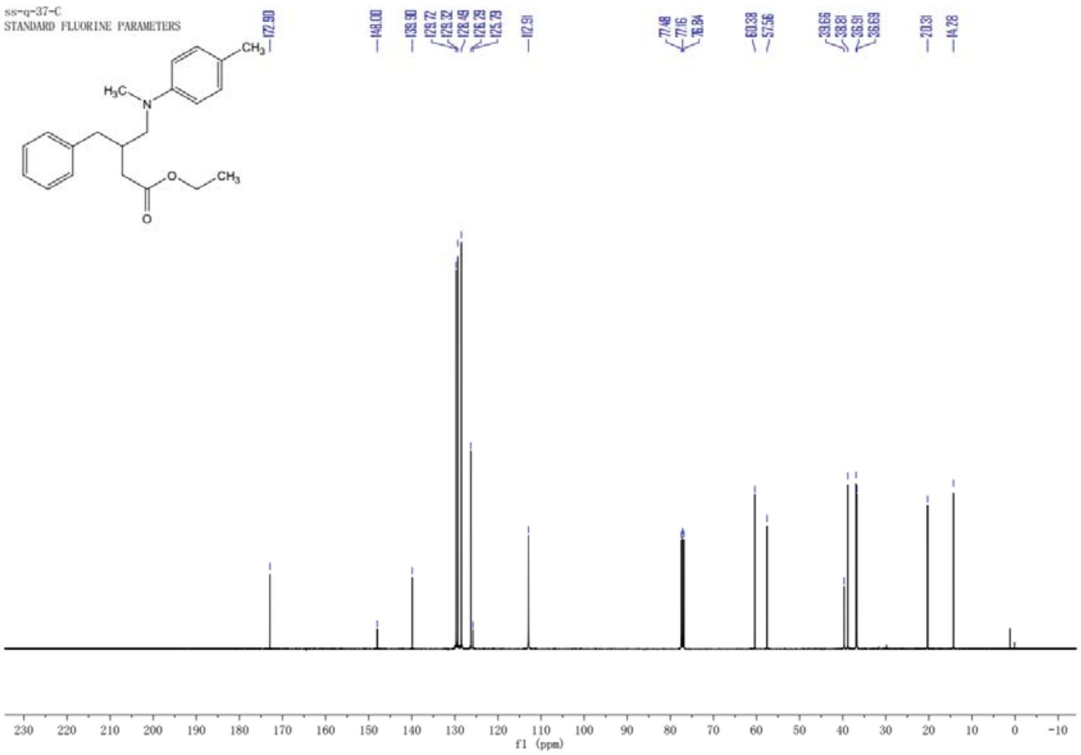

36

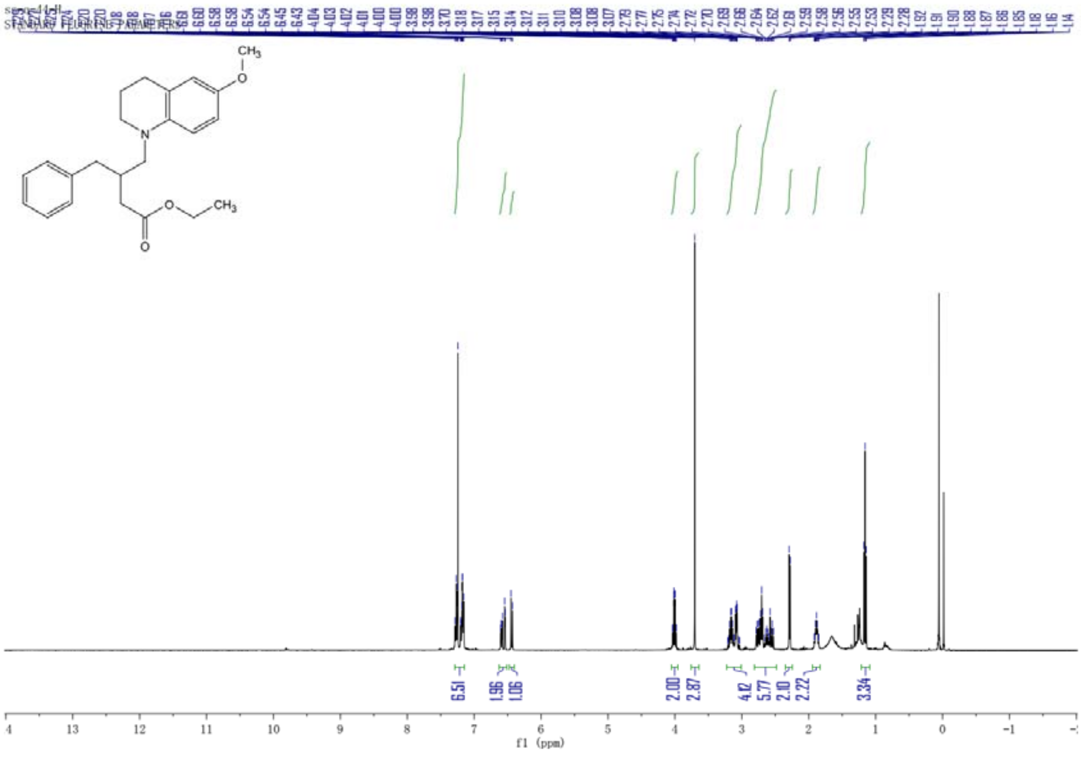

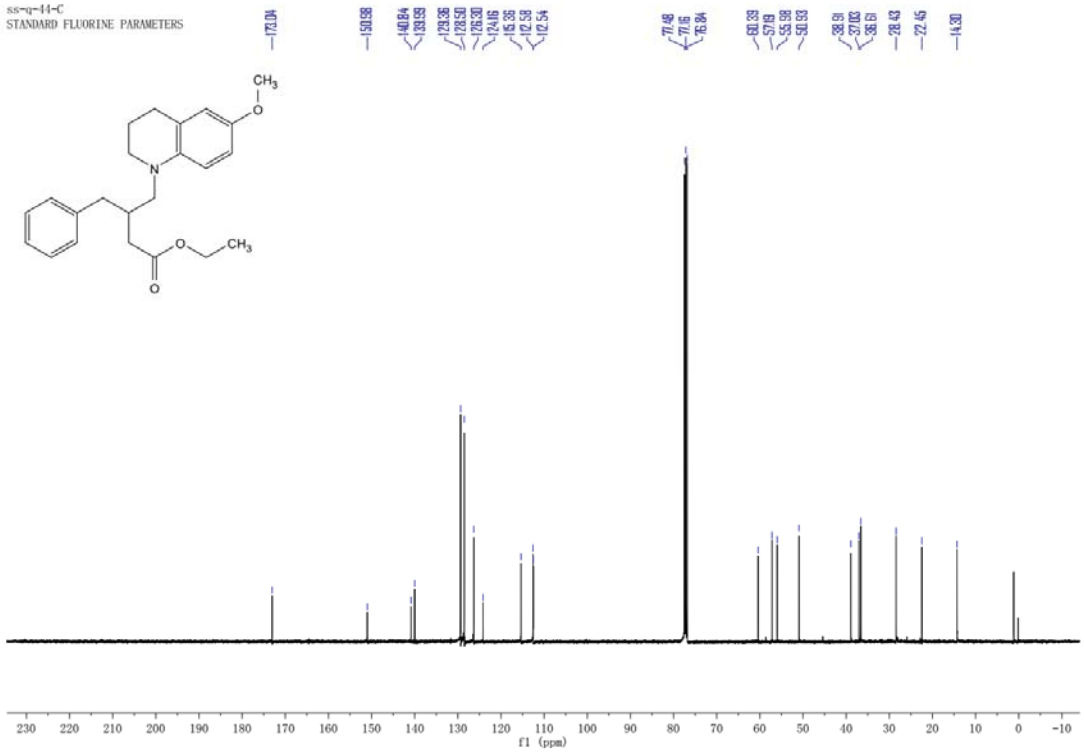
ss-q-44-C
STANDARD FLUORINE PARAMETERS
173.04
150.98
140.84
139.99
129.36
128.50
126.30
124.16
115.36
112.58
112.54
77.48
77.16
76.84
60.39
57.19
55.98
50.93
38.91
37.03
36.61
28.43
22.45
14.30
f1 (ppm)

37

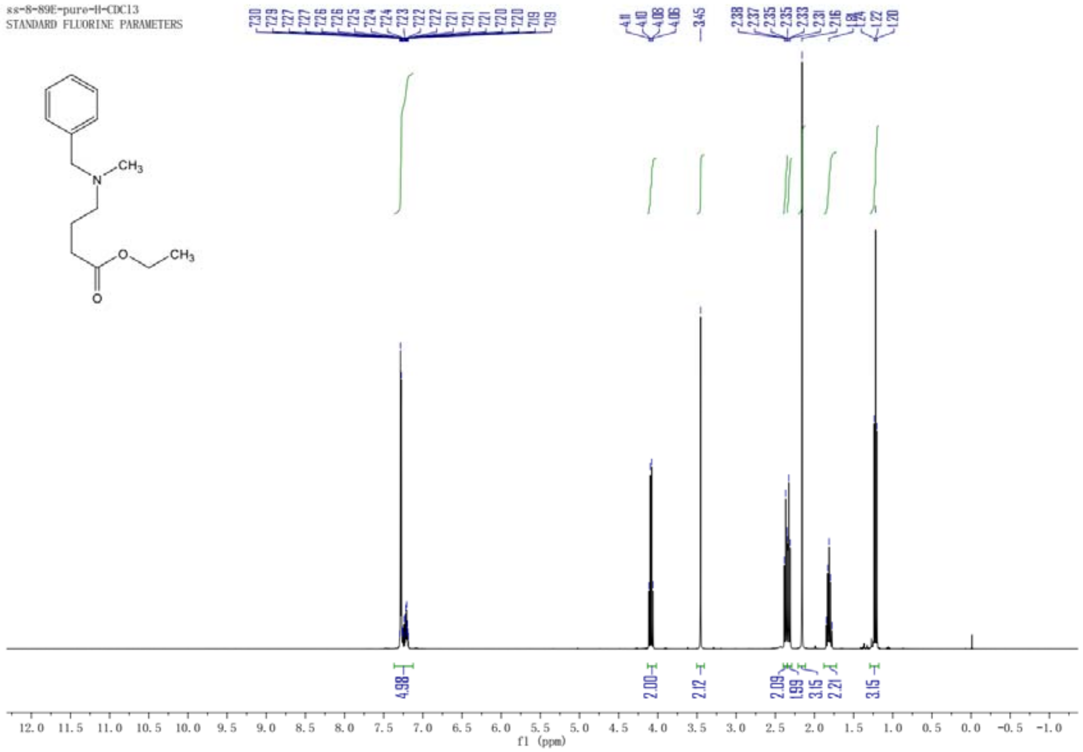
ss-8-89E-pure-H-CDCl3
STANDARD FLUORINE PARAMETERS
3.45
4.98
2.00
2.12
2.09
1.99
3.15
2.21
3.15
f1 (ppm)

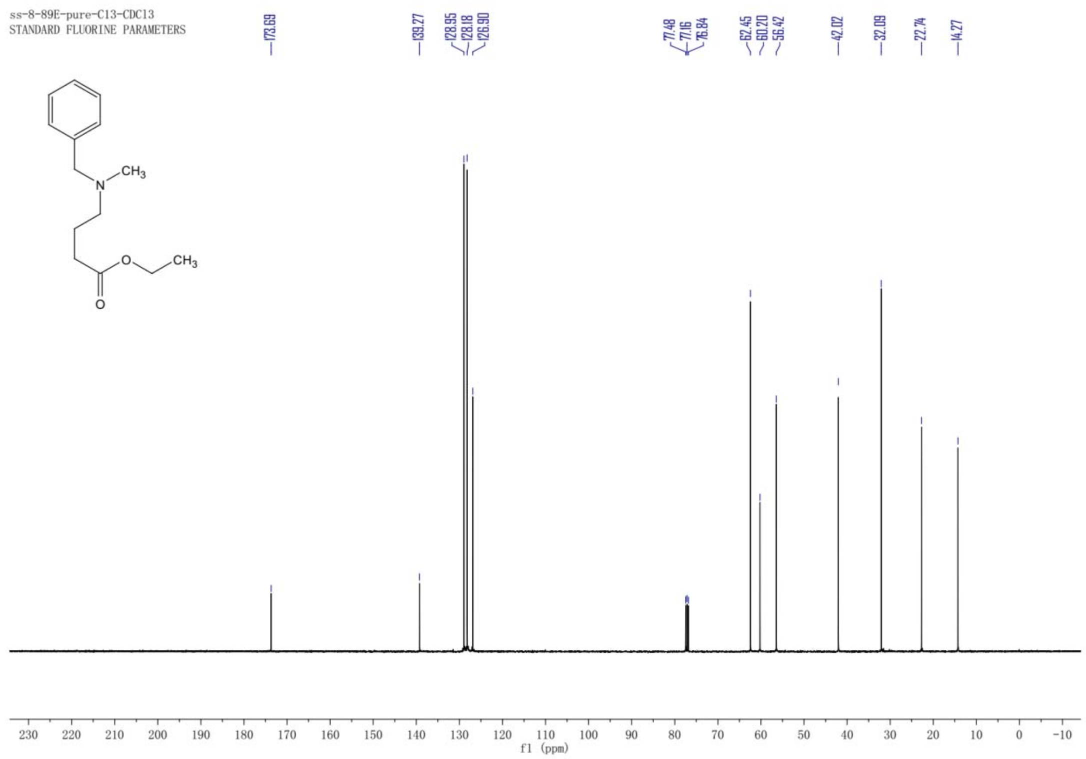
ss-8-89E-pure-C13-CDCl3
STANDARD FLUORINE PARAMETERS
173.69
139.27
128.95
128.18
126.90
77.48
77.16
76.84
62.45
60.20
56.42
42.02
32.09
22.74
14.27
CH3
N
O
CH3
O
f1 (ppm)

38

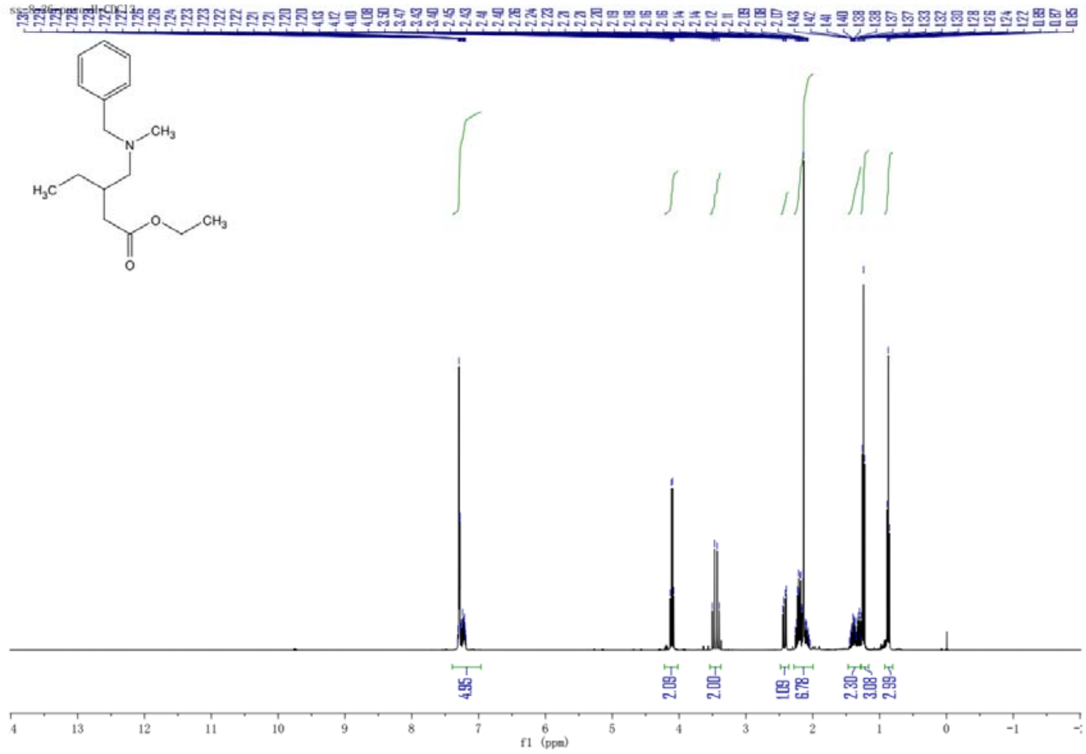
CH3
N
H3C
O
CH3
O
4.95
2.09
2.00
1.09
6.78
2.30
3.08
2.99
f1 (ppm)

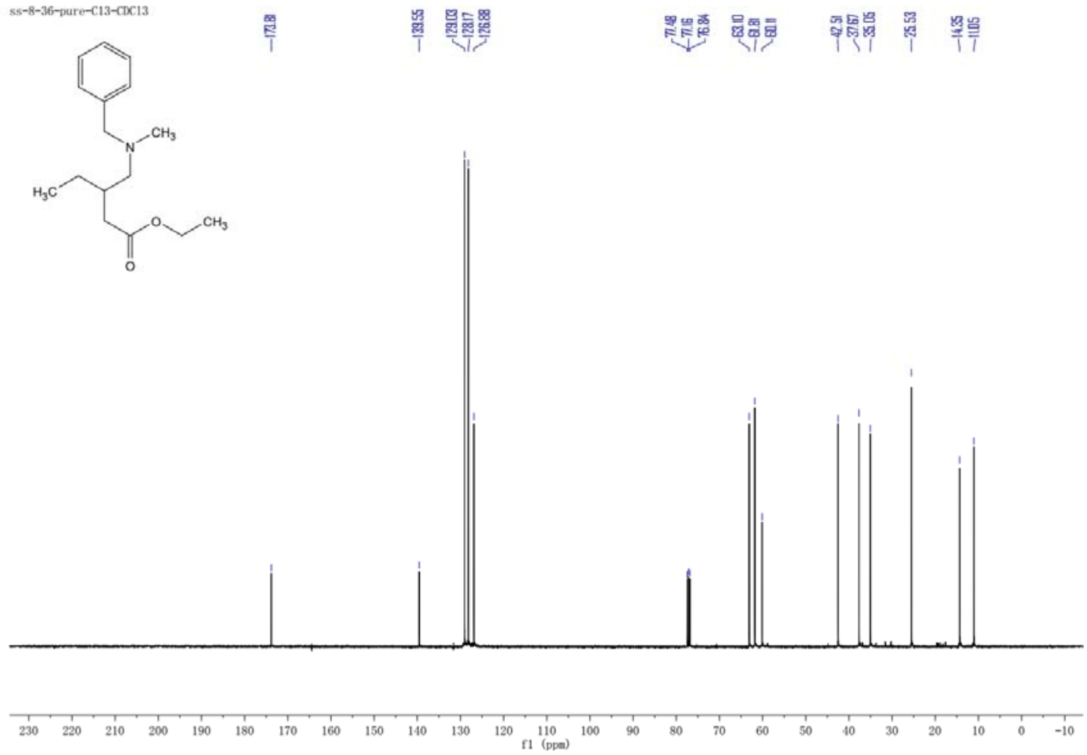

39

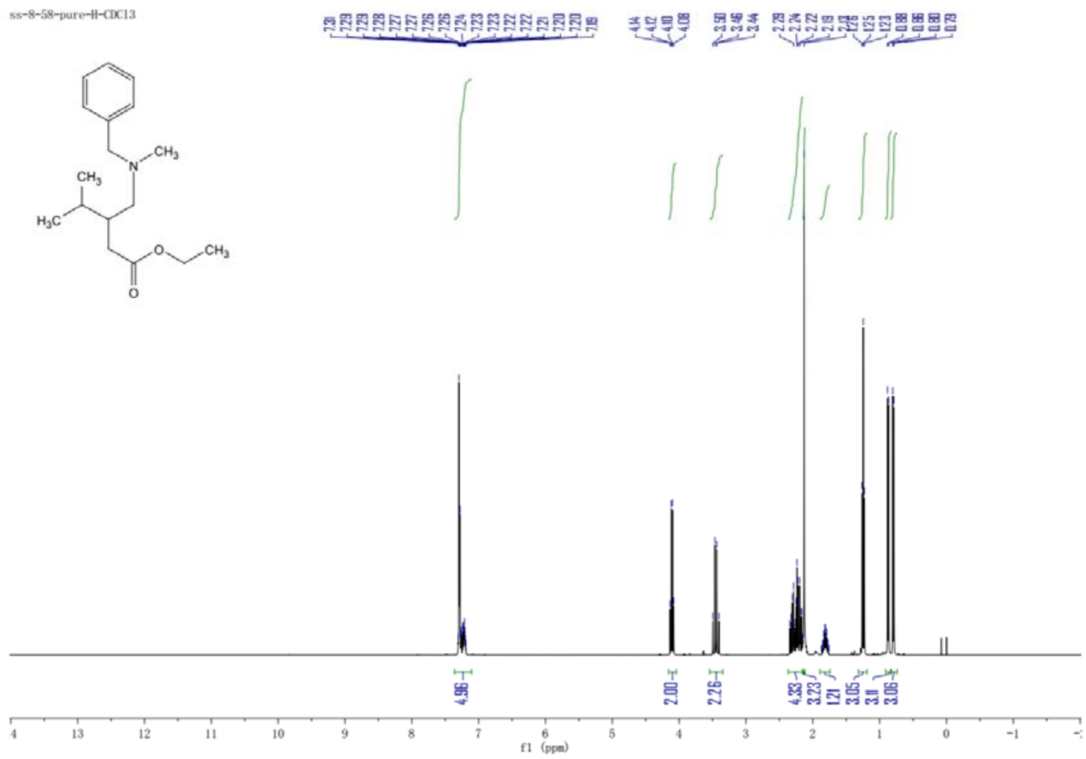

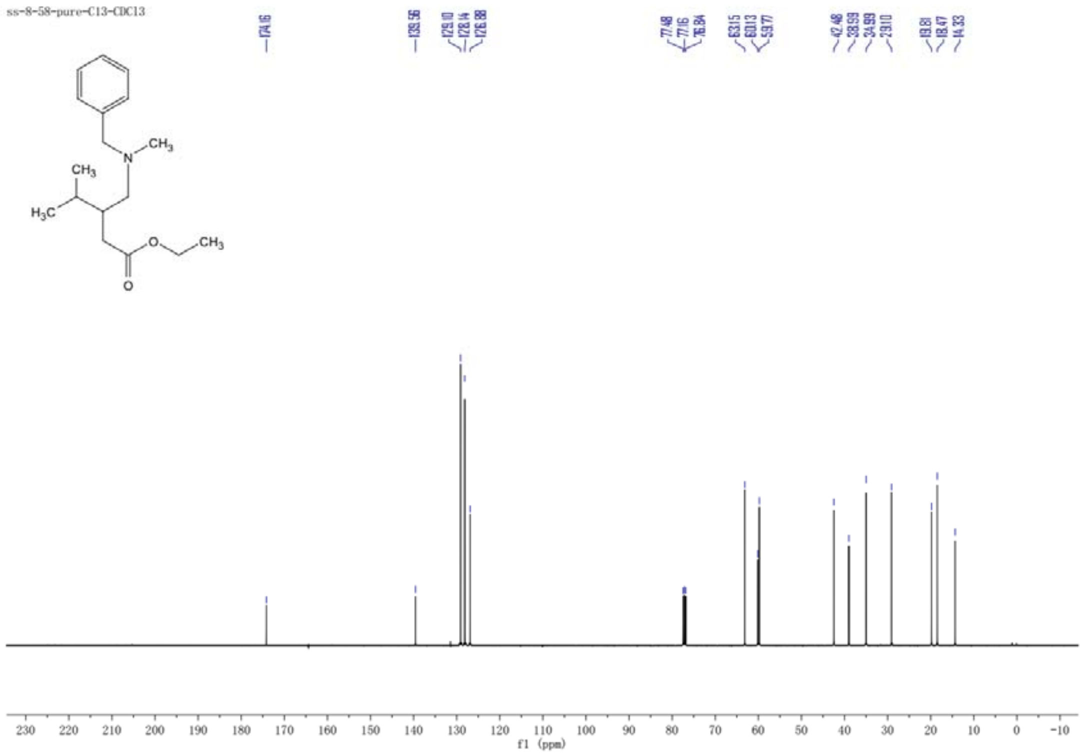
ss-8-58-pure-C13-CDCl3
174.16
139.56
129.10
128.14
126.88
77.48
77.16
76.84
63.15
60.13
59.77
42.48
38.99
34.93
29.10
19.81
18.47
14.33
f1 (ppm)

40

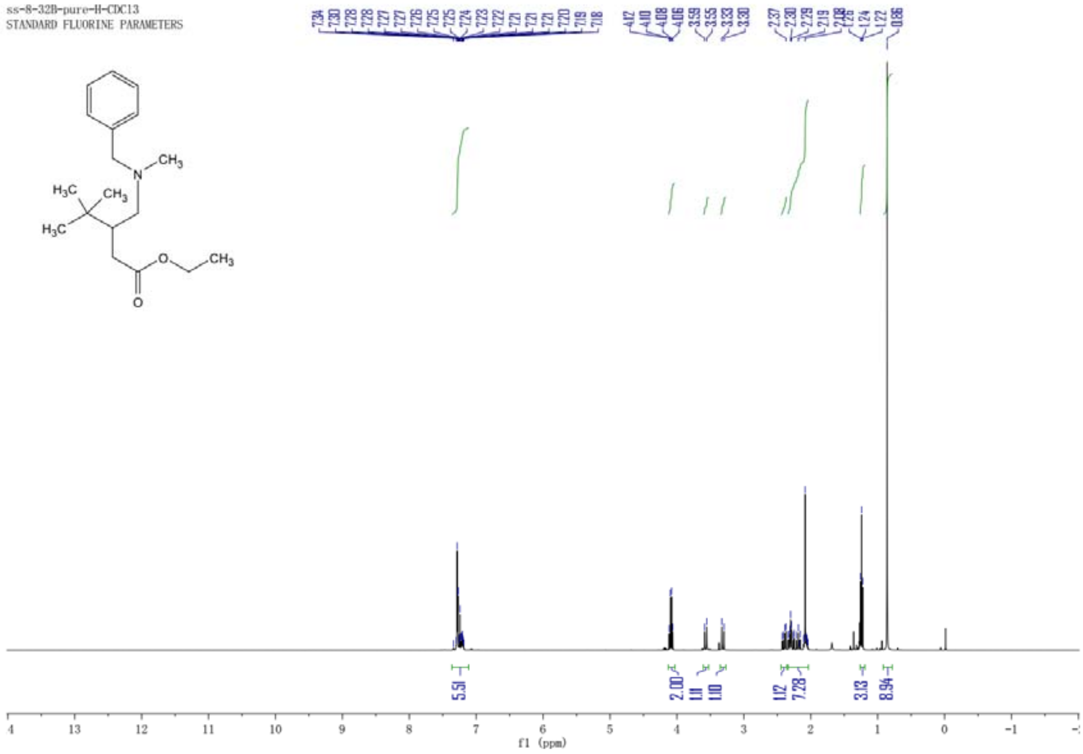
ss-8-32B-pure-H-CDCl3
STANDARD FLUORINE PARAMETERS
f1 (ppm)

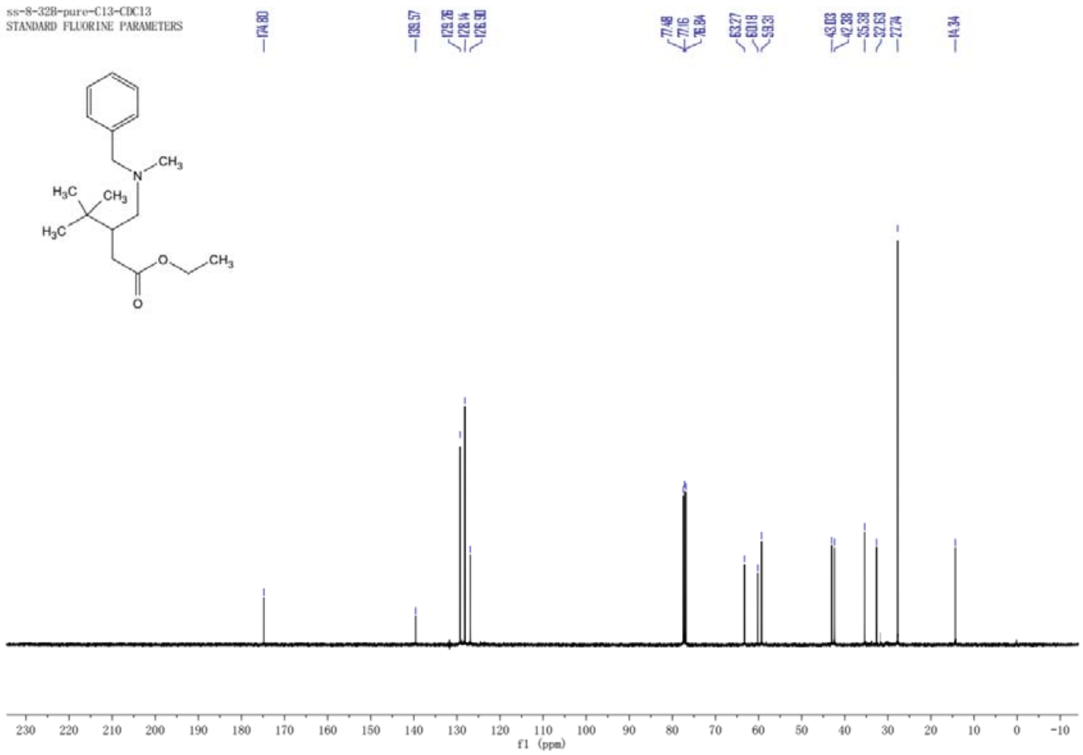

41

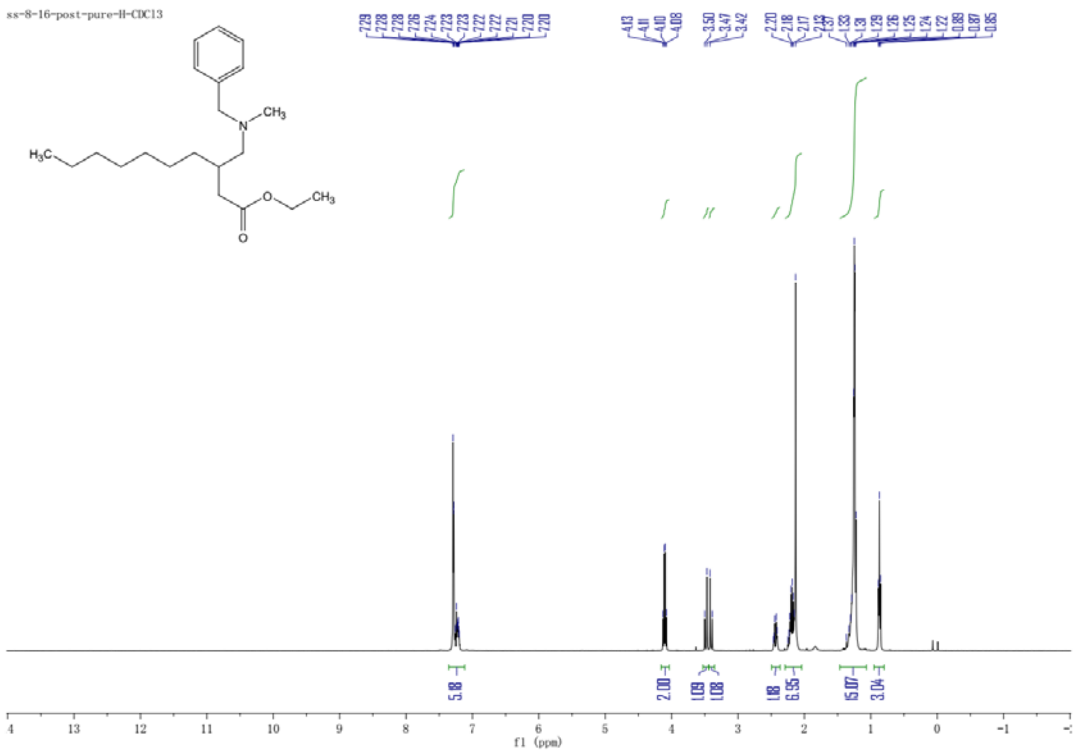

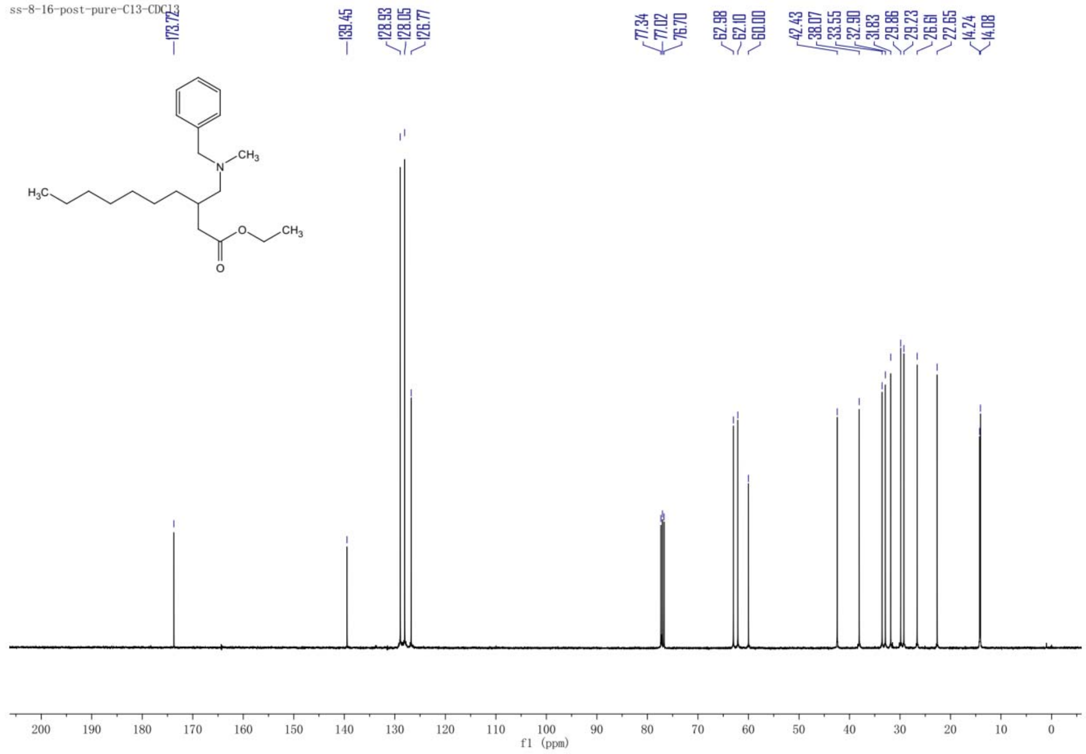
ss-8-16-post-pure-C13-CDCl3
173.72
139.45
128.93
128.05
126.77
77.34
77.02
76.70
62.98
62.10
60.00
42.43
38.07
33.55
32.90
31.83
29.86
29.23
26.61
22.65
14.24
14.08
f1 (ppm)

42

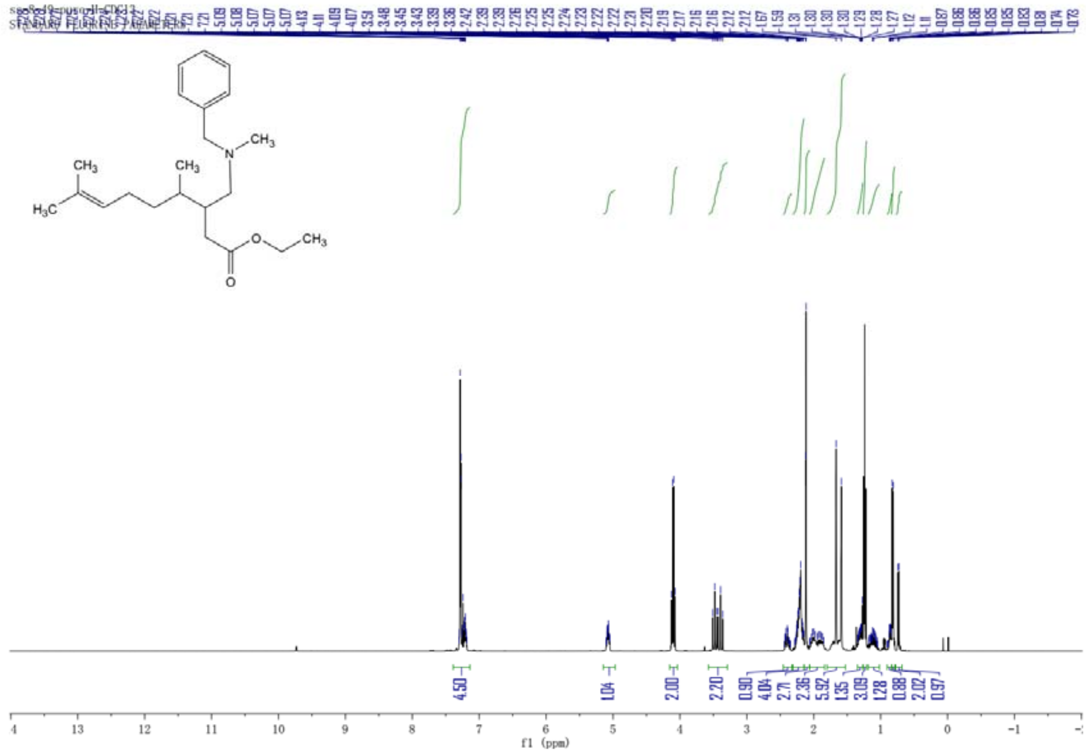
4.50
1.04
2.00
2.20
0.90
4.04
2.71
2.36
5.92
1.35
3.09
1.28
0.88
2.02
0.97
f1 (ppm)

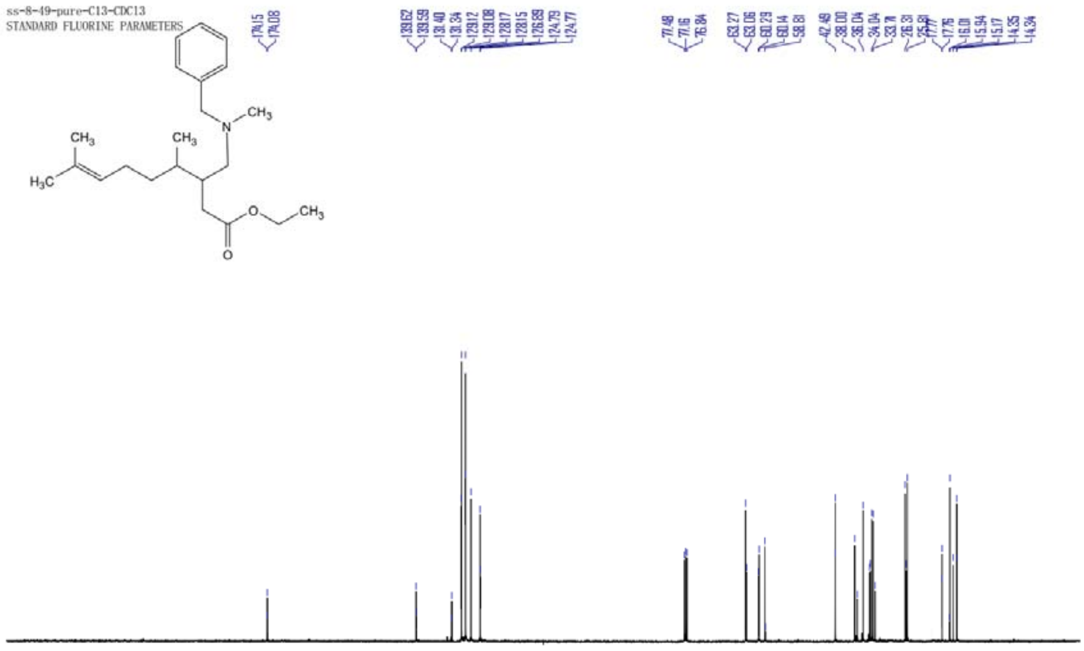
ss-8-49-pure-C13-CDCl3
STANDARD FLUORINE PARAMETERS

43

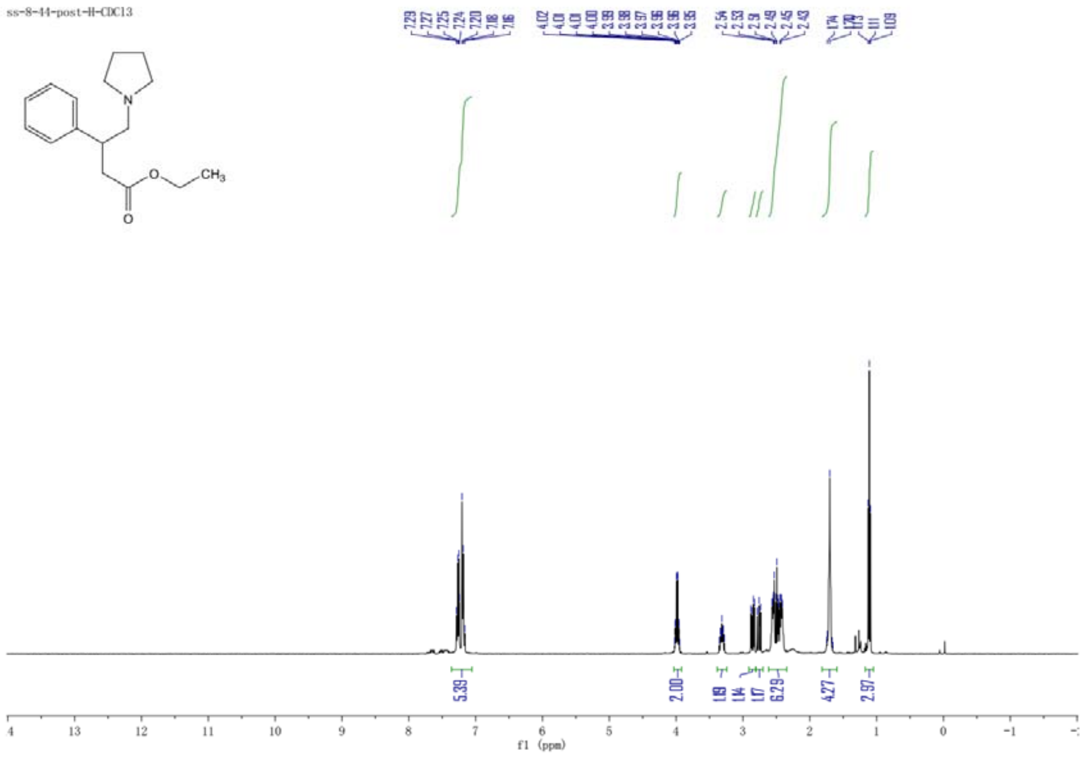
ss-8-44-post-H-CDCl3
5.39
2.00
1.19
1.14
1.17
6.29
4.27
2.97
f1 (ppm)

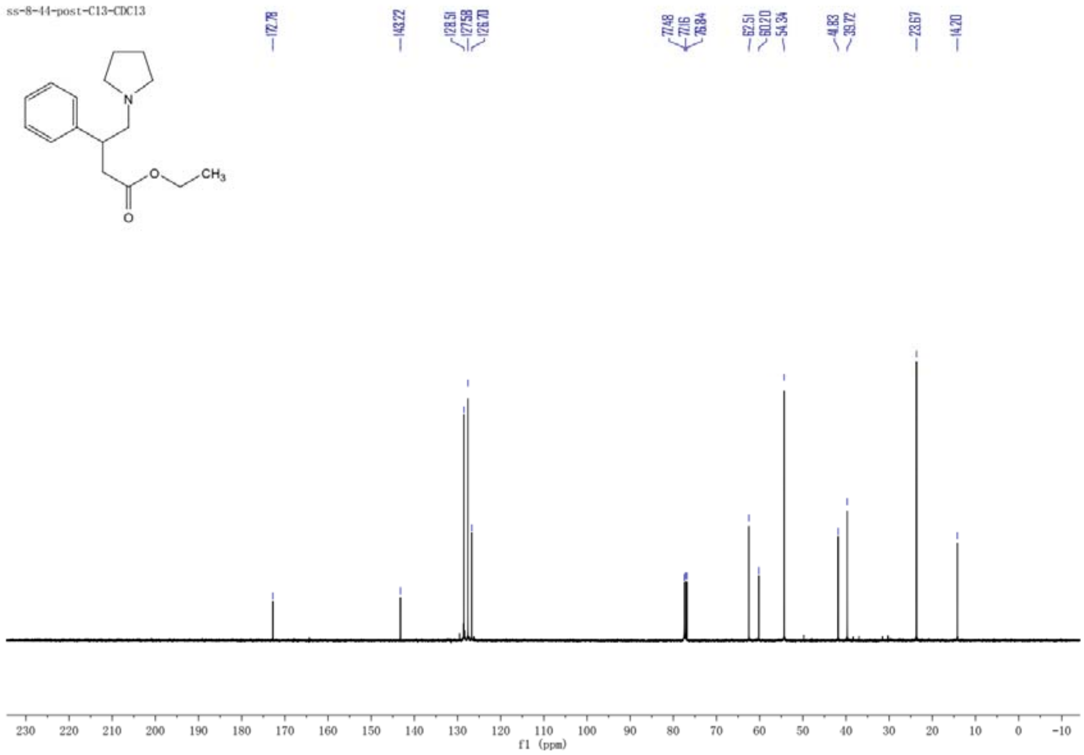
ss-8-44-post-C13-CDCl3
172.78
143.22
128.51
127.58
126.70
77.48
77.16
76.84
62.51
60.20
54.34
41.83
39.72
23.67
14.20
f1 (ppm)

44

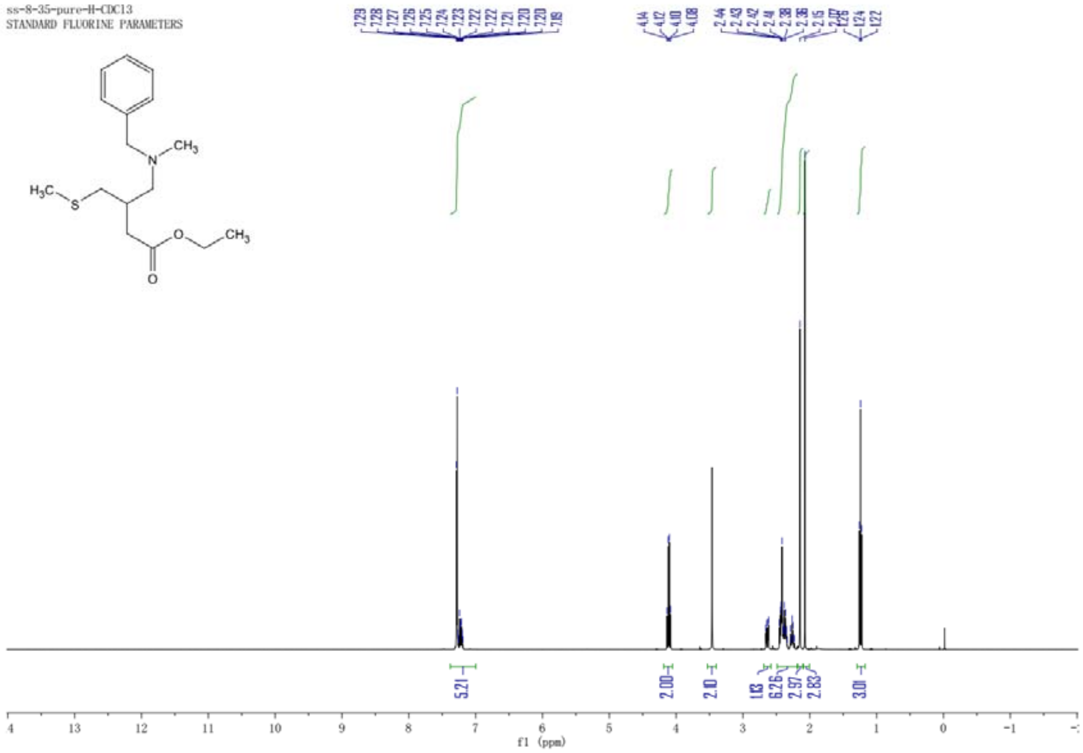
ss-8-35-pure-H-CDCl3
STANDARD FLUORINE PARAMETERS
4.14
4.12
4.10
4.08
5.21
2.00
2.10
6.26
2.97
2.83
3.01
f1 (ppm)

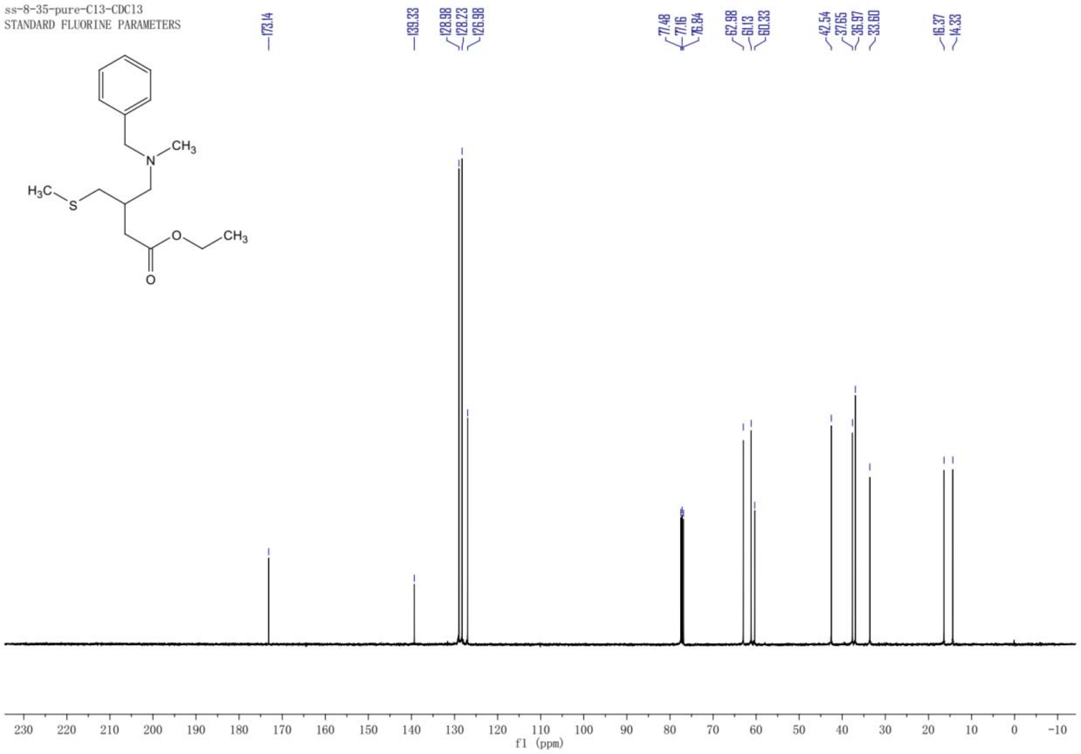
ss-8-35-pure-C13-CDCl3
STANDARD FLUORINE PARAMETERS
f1 (ppm)

45

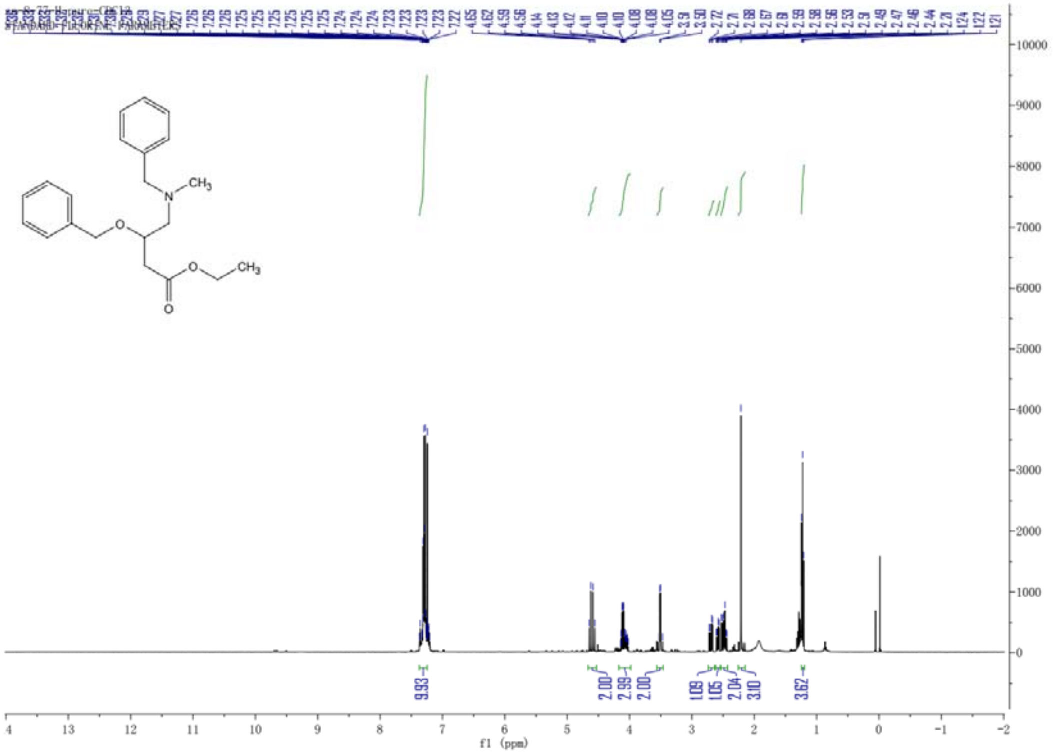
f1 (ppm)

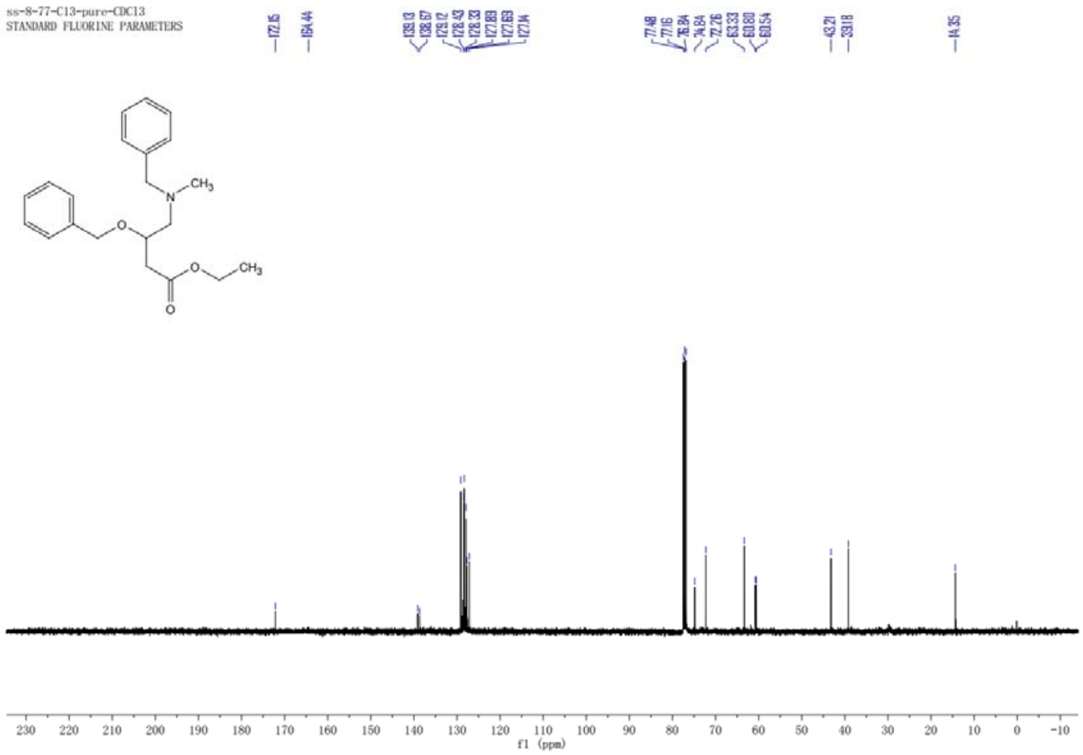
ss-8-77-C13-pure-CDCl3
STANDARD FLUORINE PARAMETERS
172.15
164.44
139.13
138.67
129.12
128.43
128.33
127.83
127.63
127.14
77.48
77.16
76.84
74.84
72.26
63.33
60.80
60.54
43.21
39.18
14.35
f1 (ppm)

46

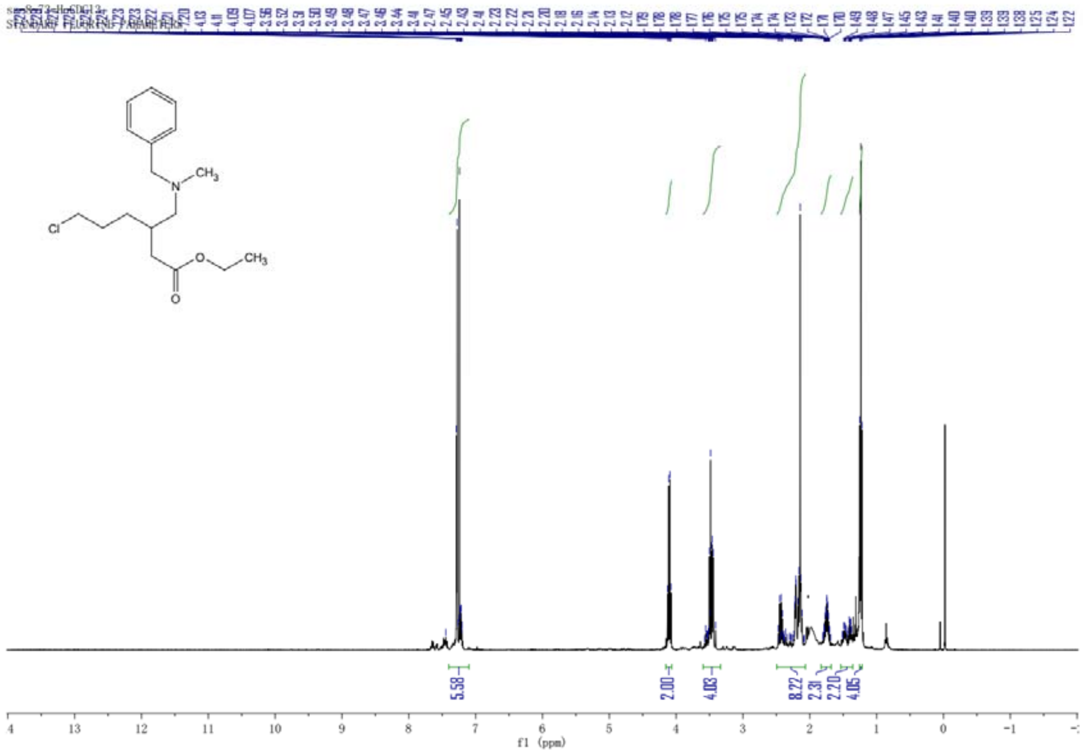
4.13
4.11
4.09
4.07
3.56
3.52
3.51
3.50
3.49
3.48
3.47
3.46
3.44
3.41
2.47
2.45
2.43
2.41
2.23
2.22
2.21
2.20
2.18
2.16
2.14
2.13
2.12
1.79
1.78
1.78
1.77
1.76
1.75
1.75
1.74
1.74
1.72
1.72
1.71
1.70
1.49
1.48
1.47
1.45
1.43
1.41
1.40
1.40
1.39
1.38
1.38
1.25
1.24
1.22
5.58
2.00
4.03
8.22
2.31
2.20
4.05
f1 (ppm)

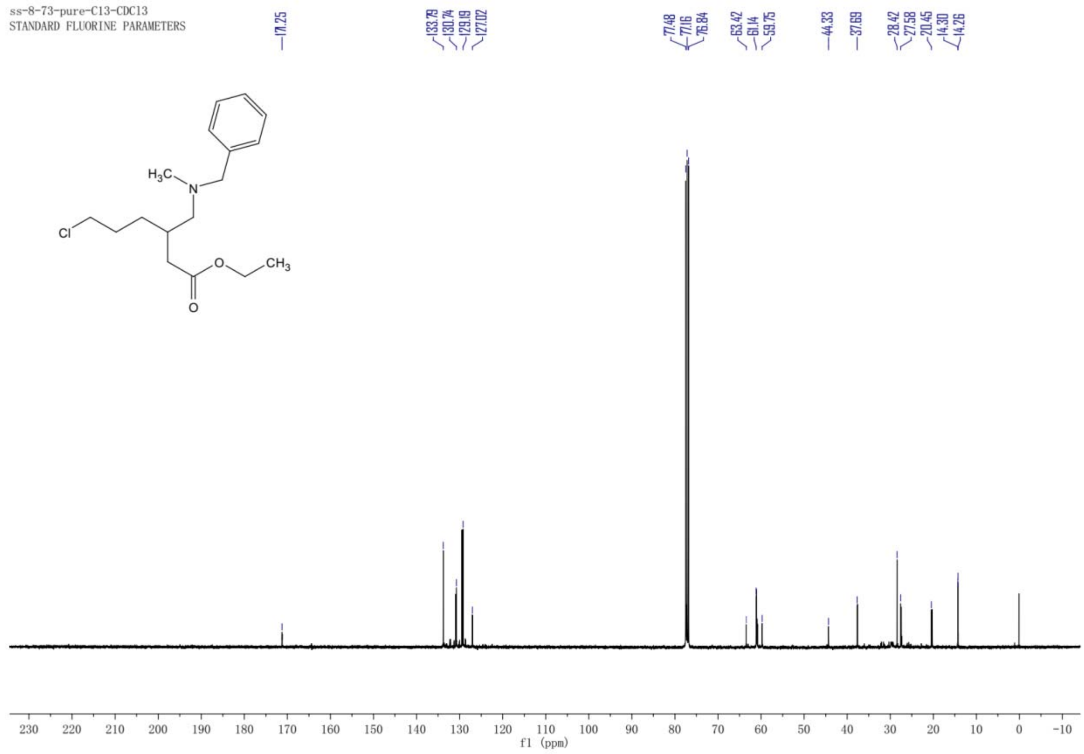
ss-8-73-pure-C13-CDCl3
STANDARD FLUORINE PARAMETERS
171.25
133.79
130.74
129.19
127.02
77.48
77.16
76.84
63.42
61.14
59.75
44.33
37.69
28.42
27.58
20.45
14.30
14.26
Cl
H_3C
N
O
CH_3
O
f1 (ppm)

47

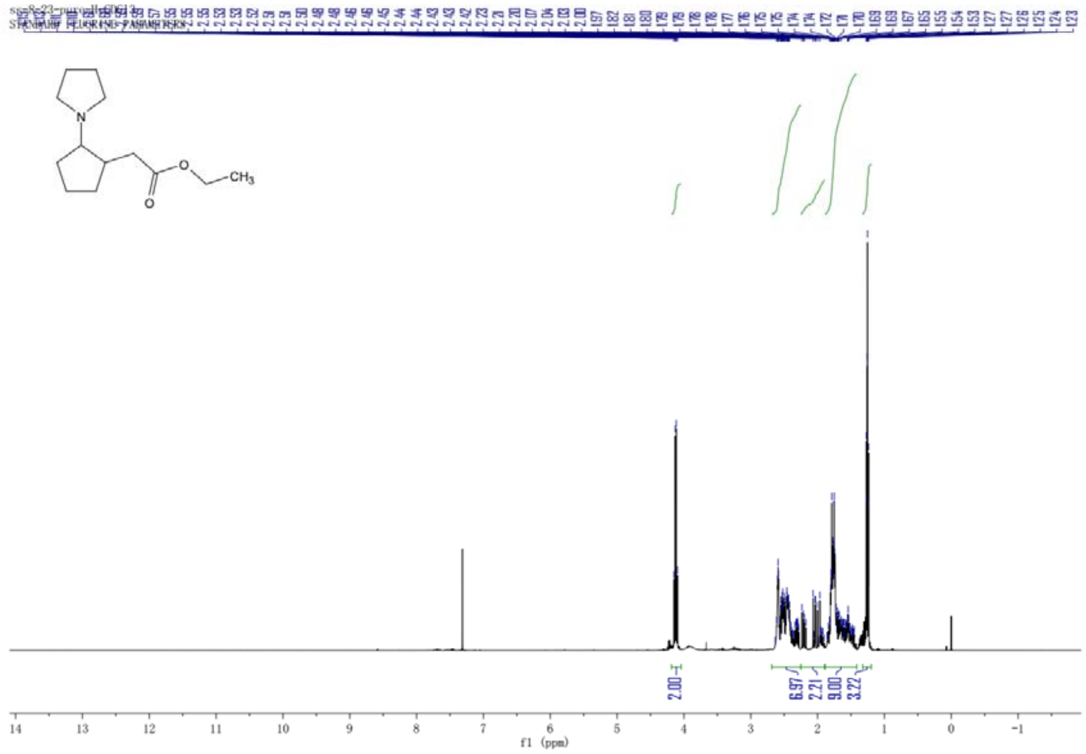
N
O
CH_3
O
2.00
6.97
2.21
9.00
3.22
f1 (ppm)

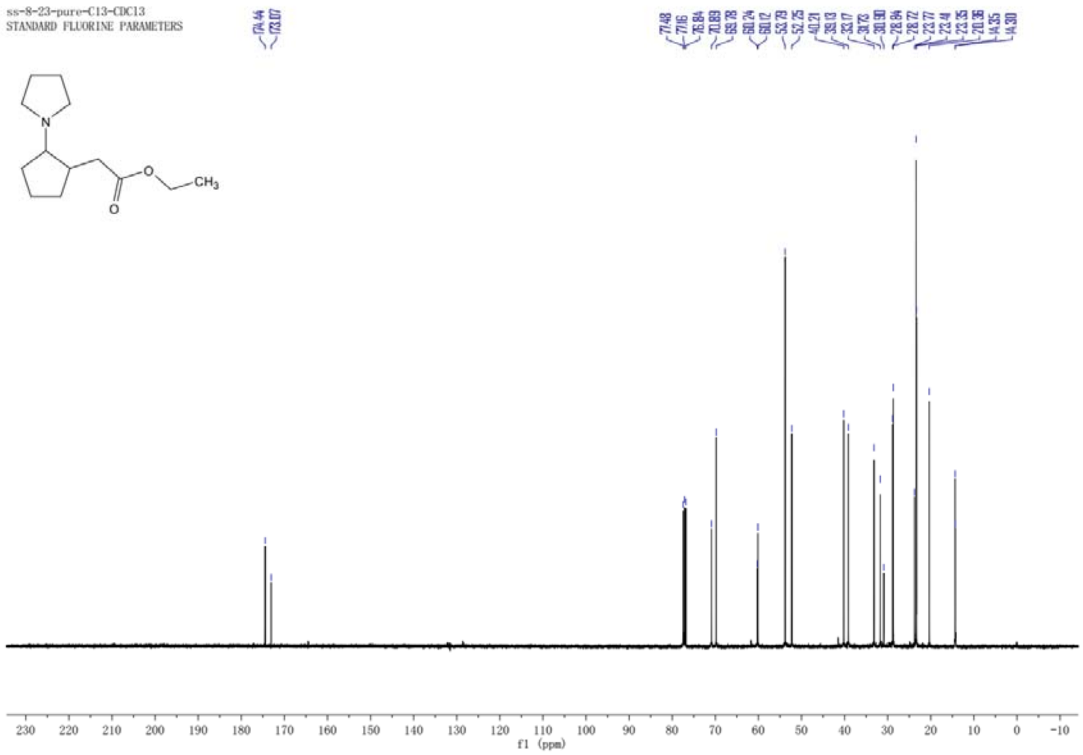
ss-8-23-pure-C13-CDCl3
STANDARD FLUORINE PARAMETERS
f1 (ppm)

48

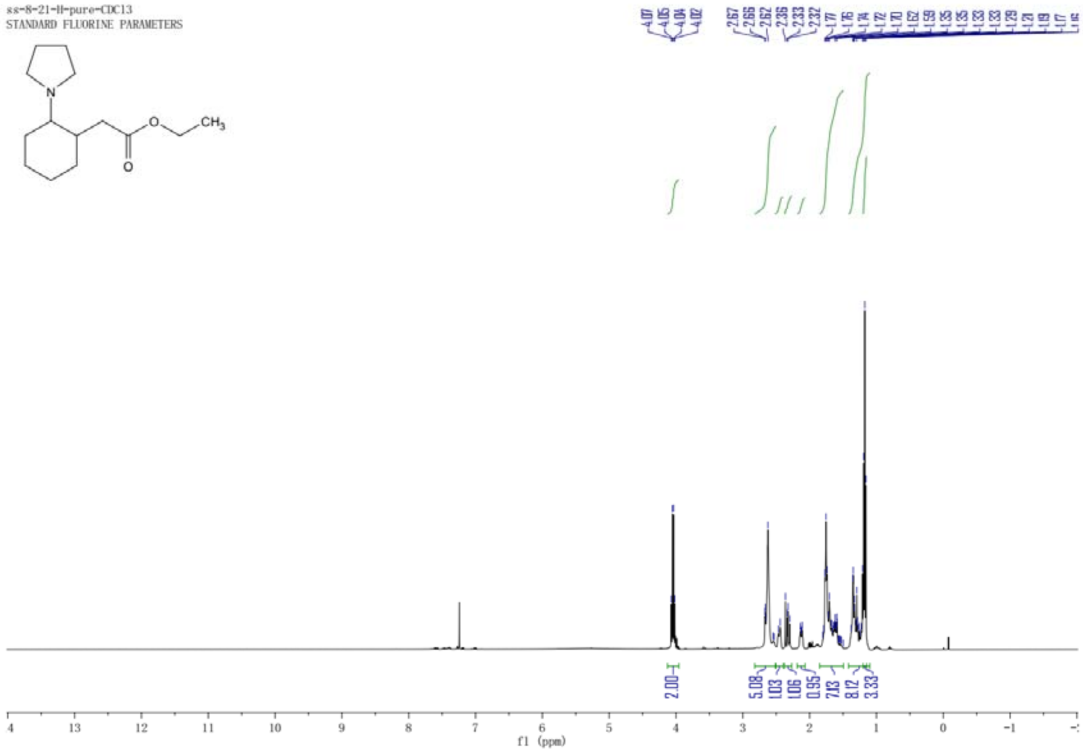
ss-8-21-H-pure-CDCl3
STANDARD FLUORINE PARAMETERS
f1 (ppm)

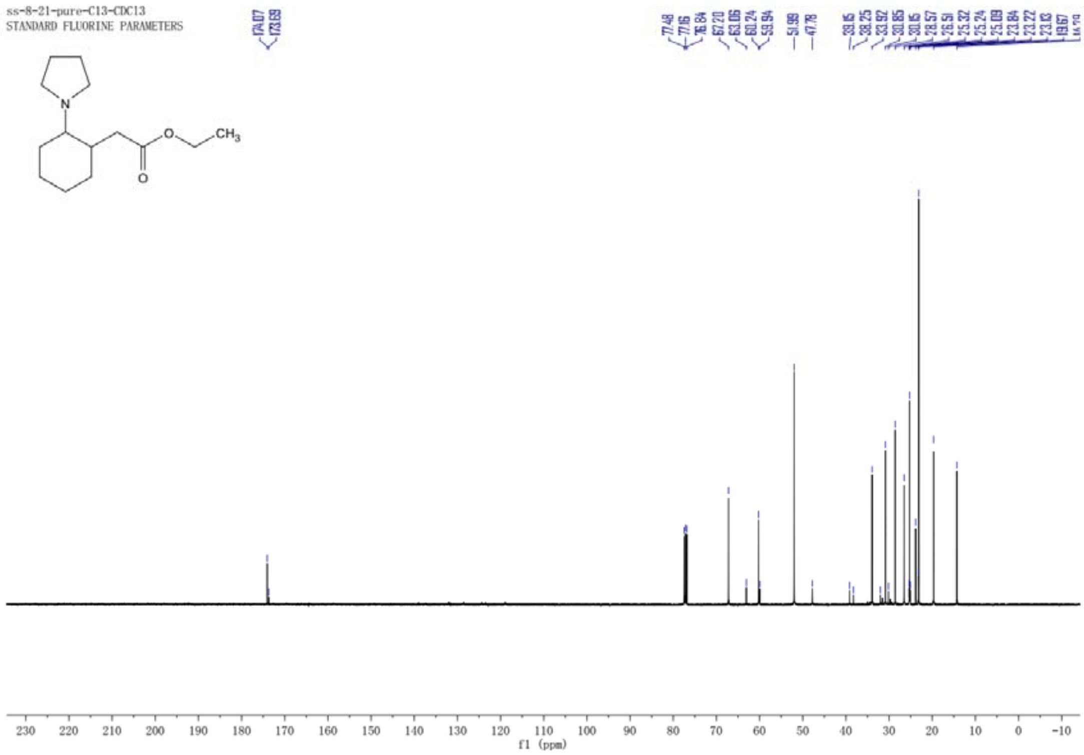
ss-8-21-pure-C13-CDCl3
STANDARD FLUORINE PARAMETERS
N
O
O
CH3
174.07
173.89
77.48
77.16
76.84
67.20
63.06
61.24
59.94
51.99
47.78
39.15
38.25
33.92
30.85
30.15
28.57
26.51
25.32
25.24
25.09
23.84
23.22
23.13
19.67
230 220 210 200 190 180 170 160 150 140 130 120 110 100 90 80 70 60 50 40 30 20 10 0 -10
f1 (ppm)

49

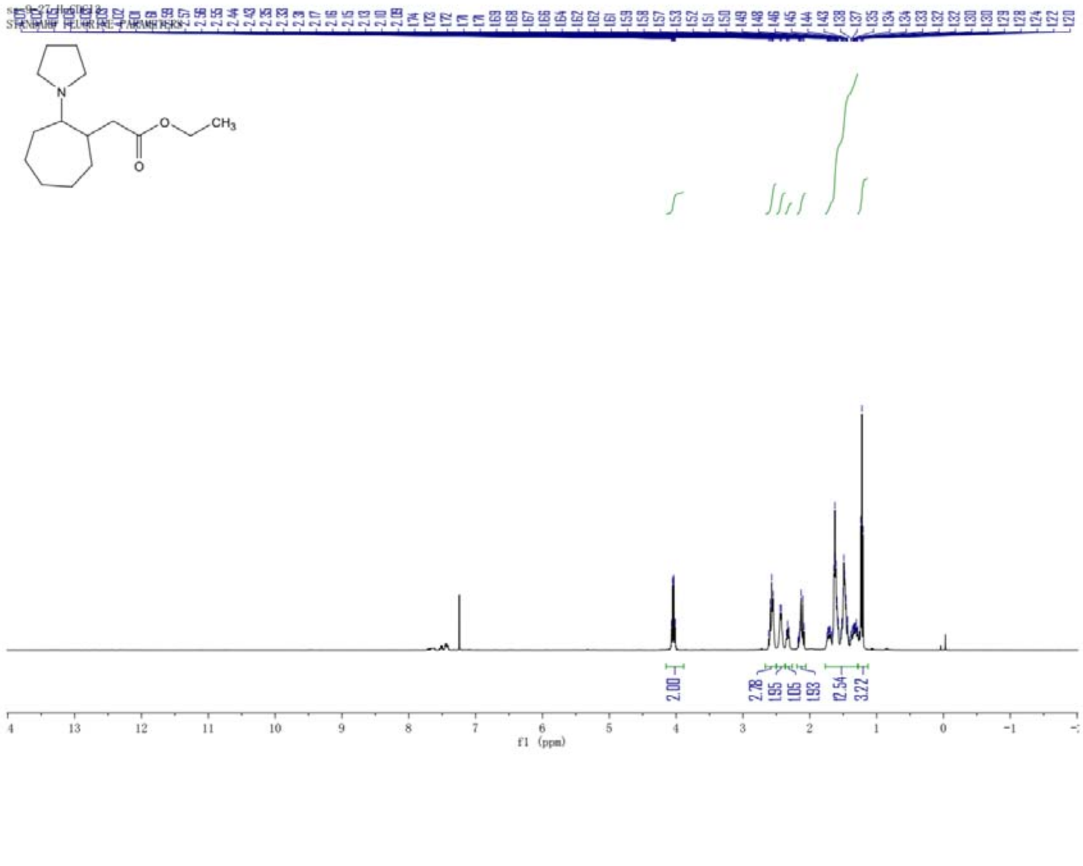
STANDARD FLUORINE PARAMETERS
N
O
O
CH3
2.00
2.78
1.95
1.05
1.93
12.54
3.22
13 12 11 10 9 8 7 6 5 4 3 2 1 0 -1
f1 (ppm)

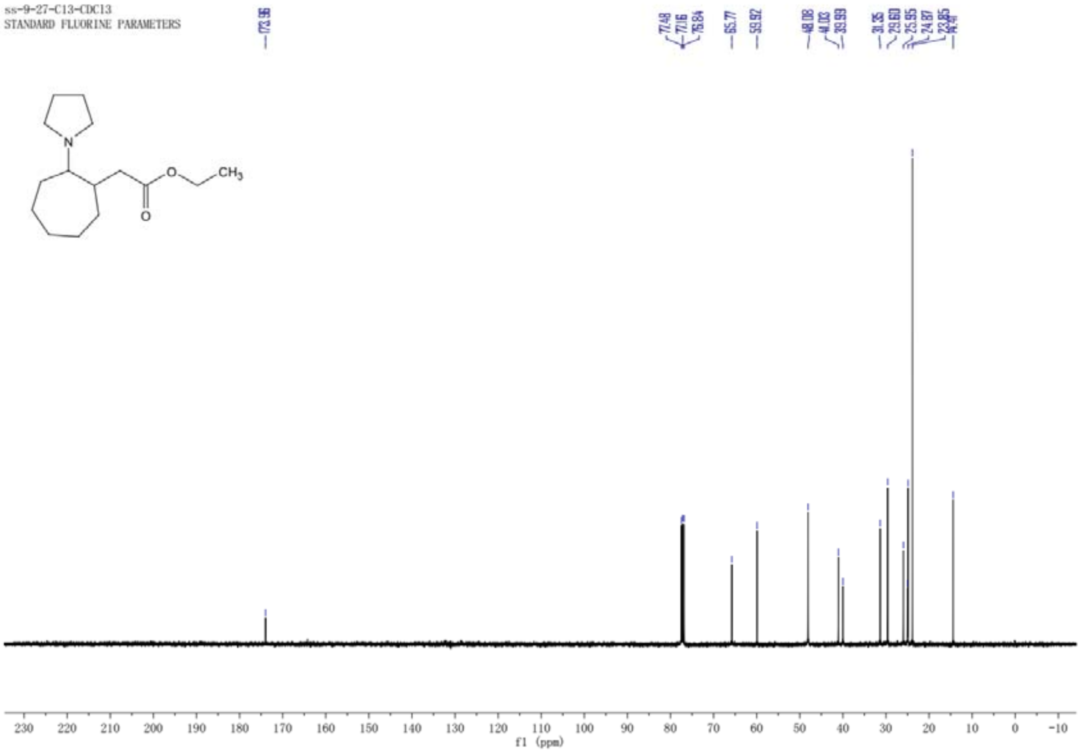
ss-9-27-C13-CDCl3
STANDARD FLUORINE PARAMETERS
173.96
77.48
77.16
76.84
65.77
59.92
48.08
41.03
39.93
31.35
29.60
25.95
24.87
23.85
f1 (ppm)

50

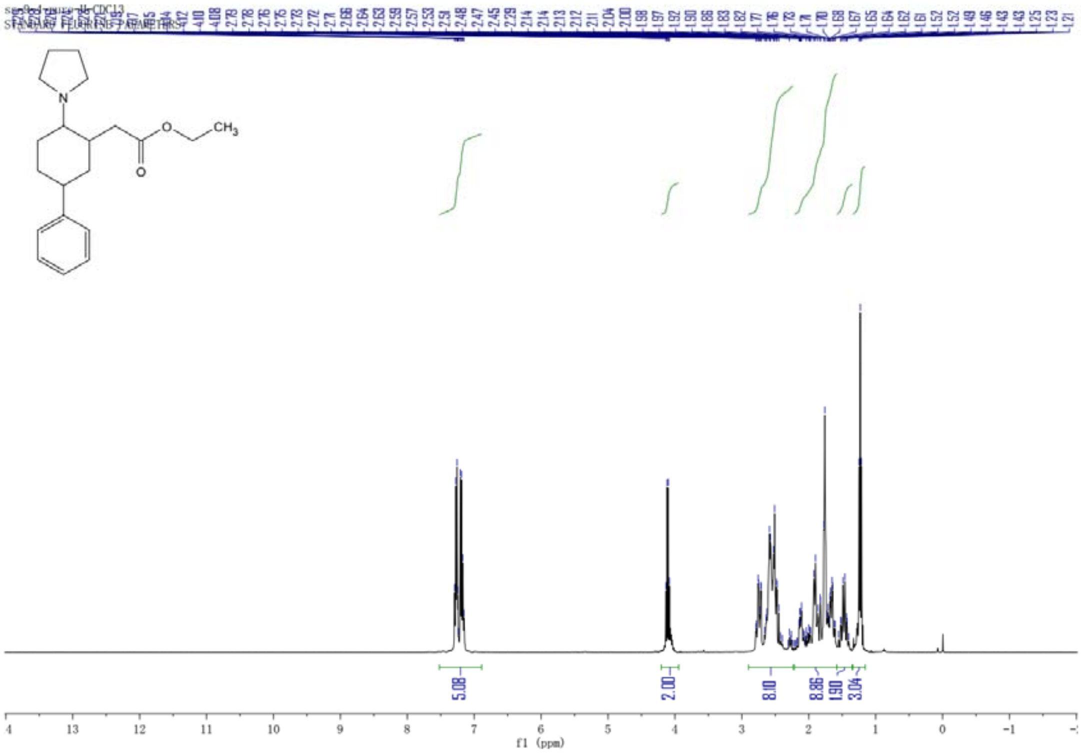
5.08
2.00
8.10
8.86
1.90
3.04
f1 (ppm)

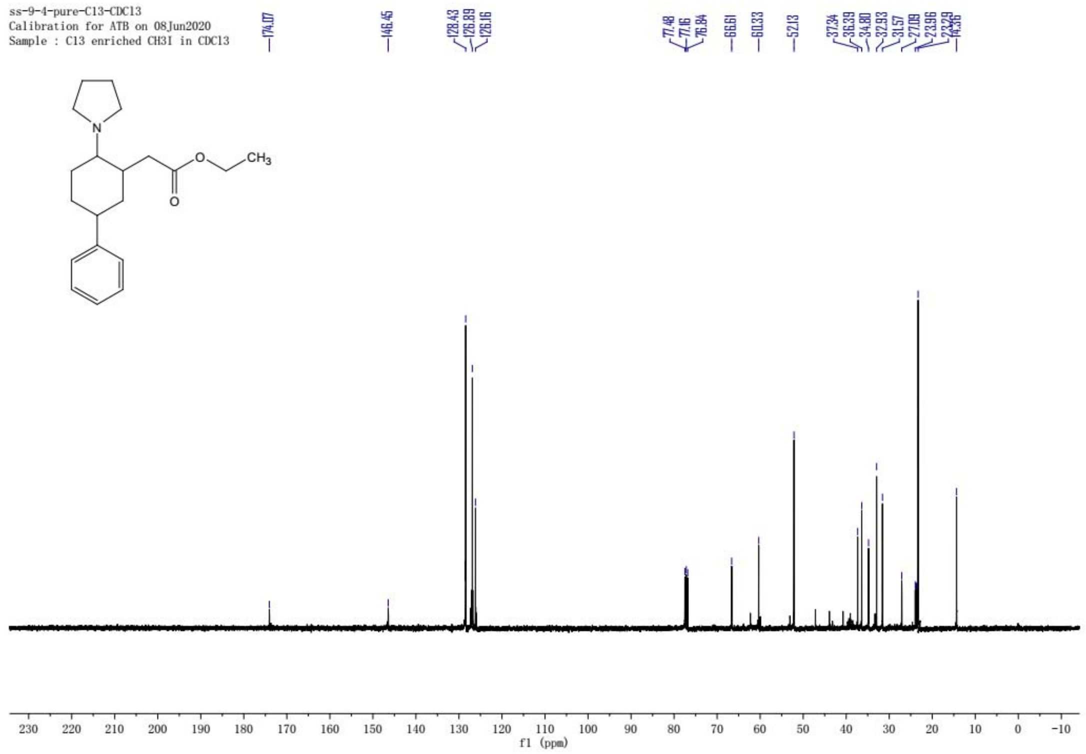
ss-9-4-pure-C13-CDCl3
Calibration for ATB on 08Jun2020
Sample : C13 enriched CH3I in CDCl3
174.07
146.45
128.43
126.89
126.16
77.48
77.16
76.84
66.61
60.33
52.13
37.34
36.39
34.80
32.93
31.57
27.09
23.96
f1 (ppm)

51

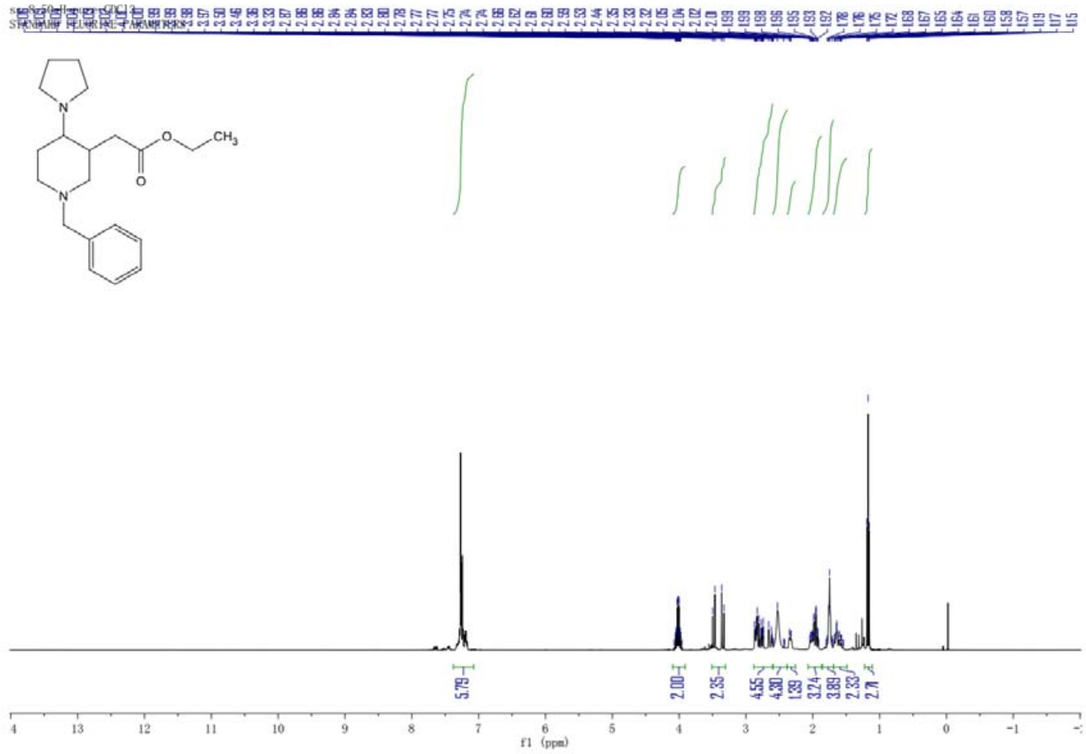
5.79
2.00
2.35
4.55
4.30
1.39
3.24
3.89
2.33
2.71
f1 (ppm)

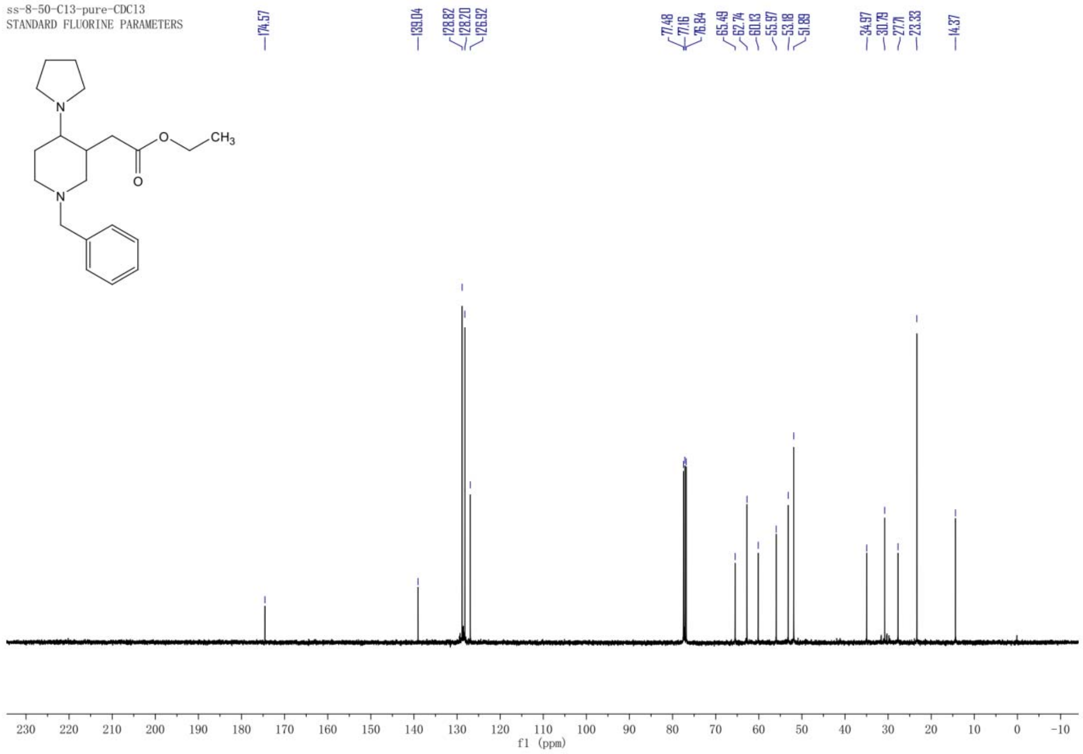
ss-8-50-C13-pure-CDCl3
STANDARD FLUORINE PARAMETERS
f1 (ppm)

52

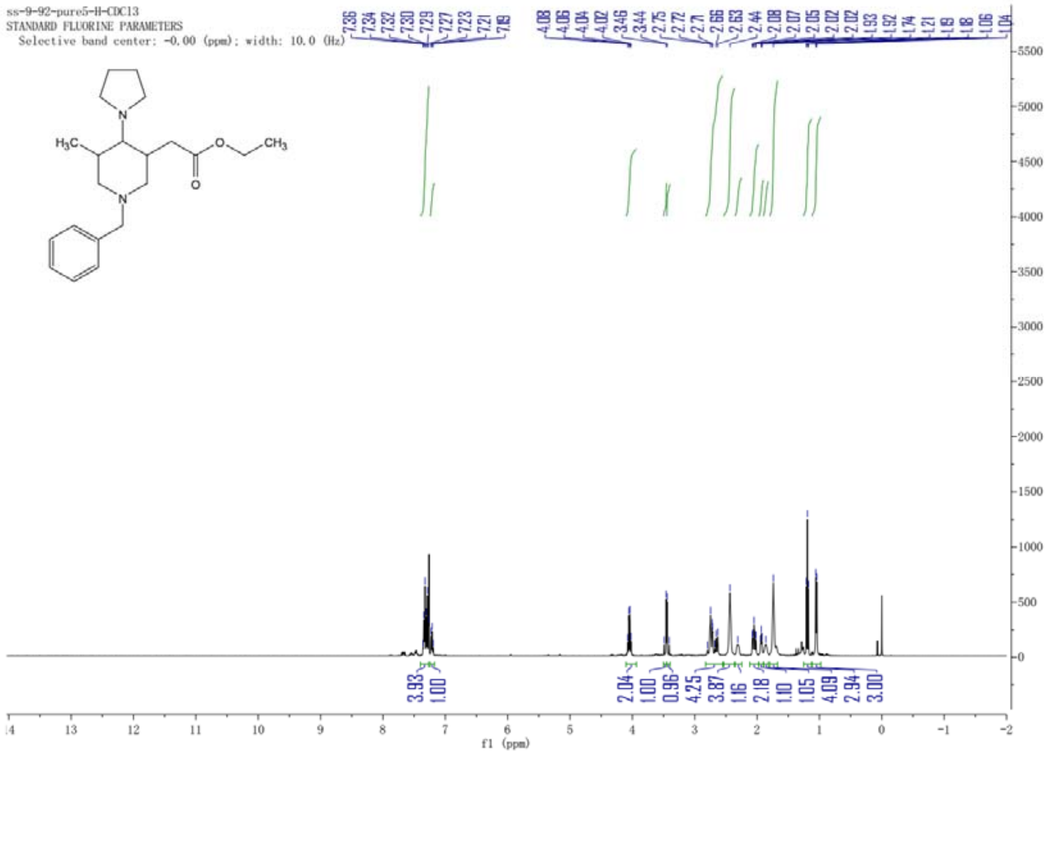
ss-9-92-pure5-H-CDCl3
STANDARD FLUORINE PARAMETERS
Selective band center: -0.00 (ppm); width: 10.0 (Hz)
f1 (ppm)

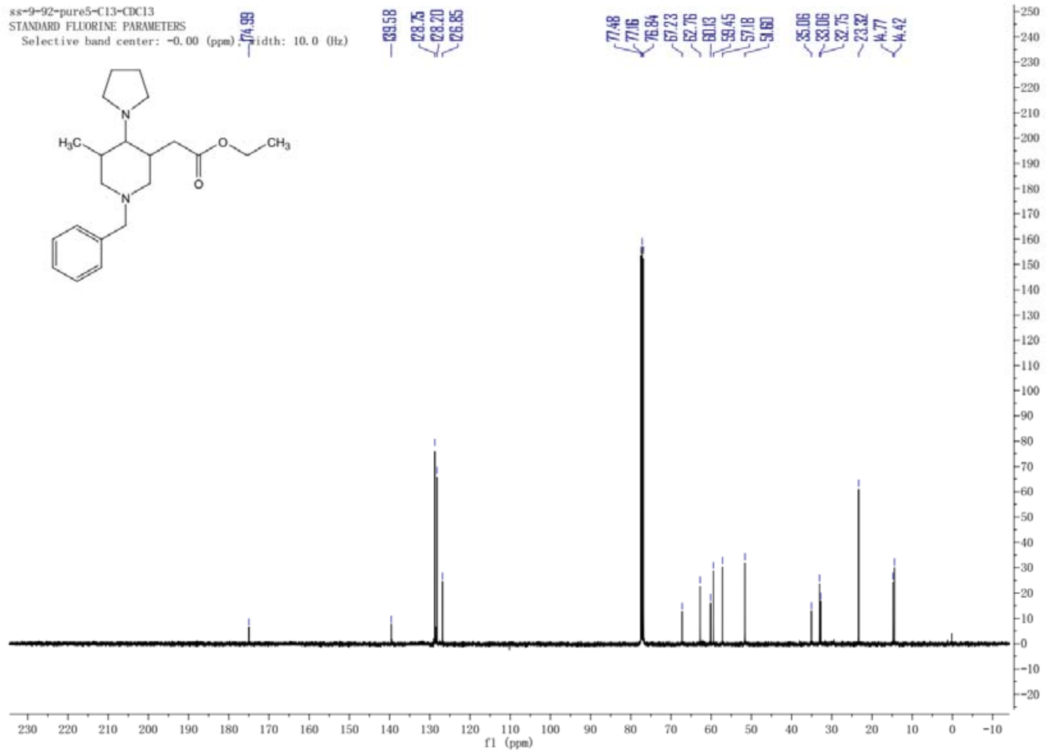

53

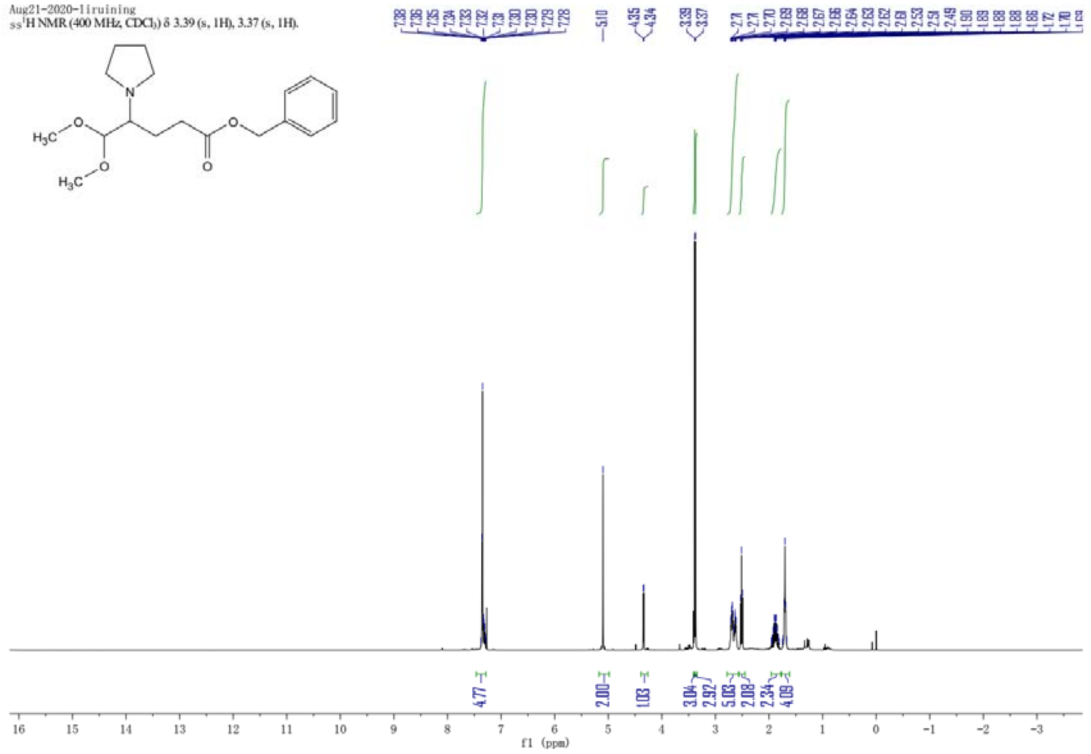

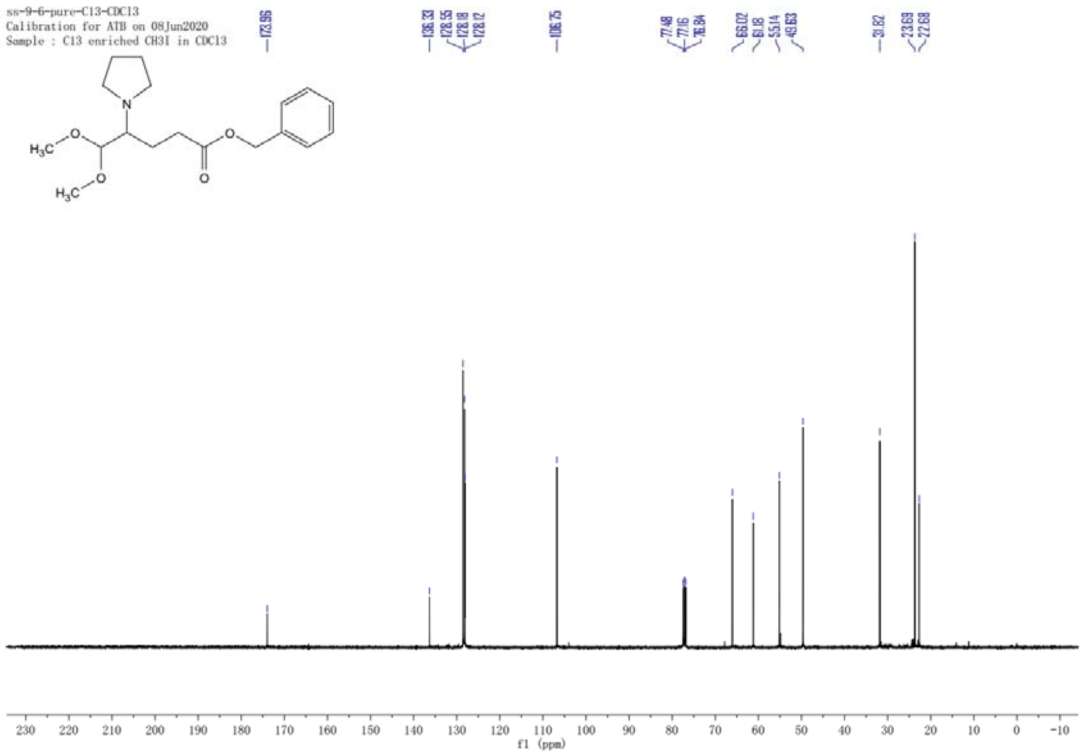
ss-9-6-pure-C13-CDCl3
Calibration for ATB on 08Jun2020
Sample : C13 enriched CH3I in CDCl3
f1 (ppm)

54

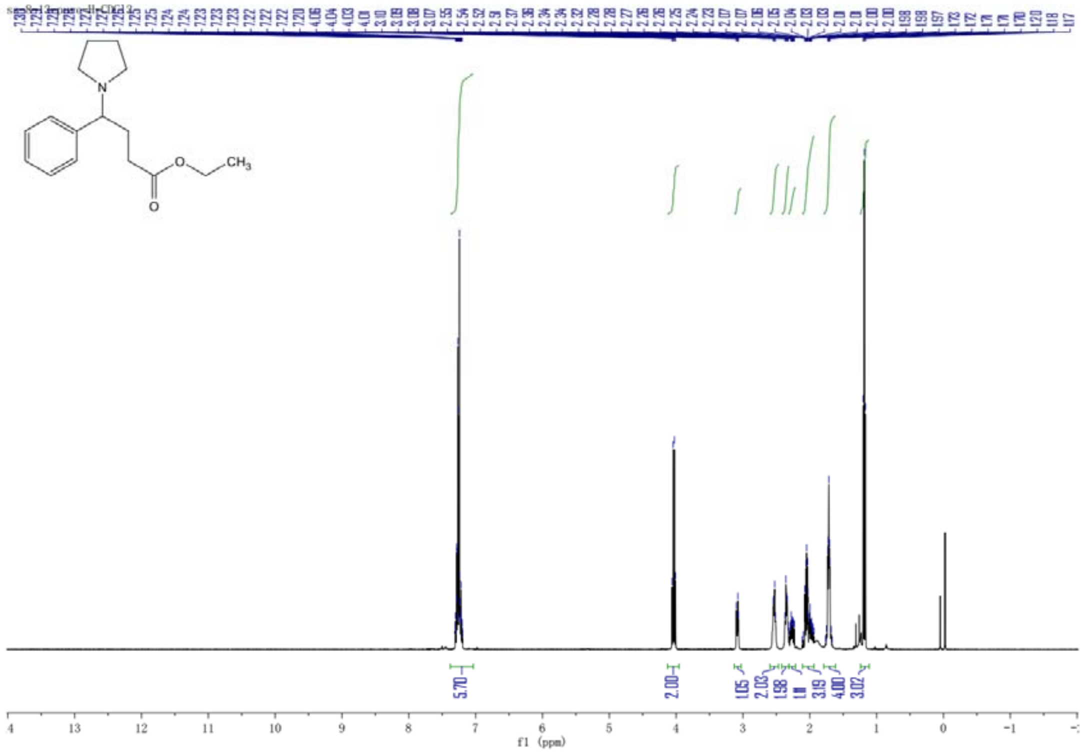
f1 (ppm)

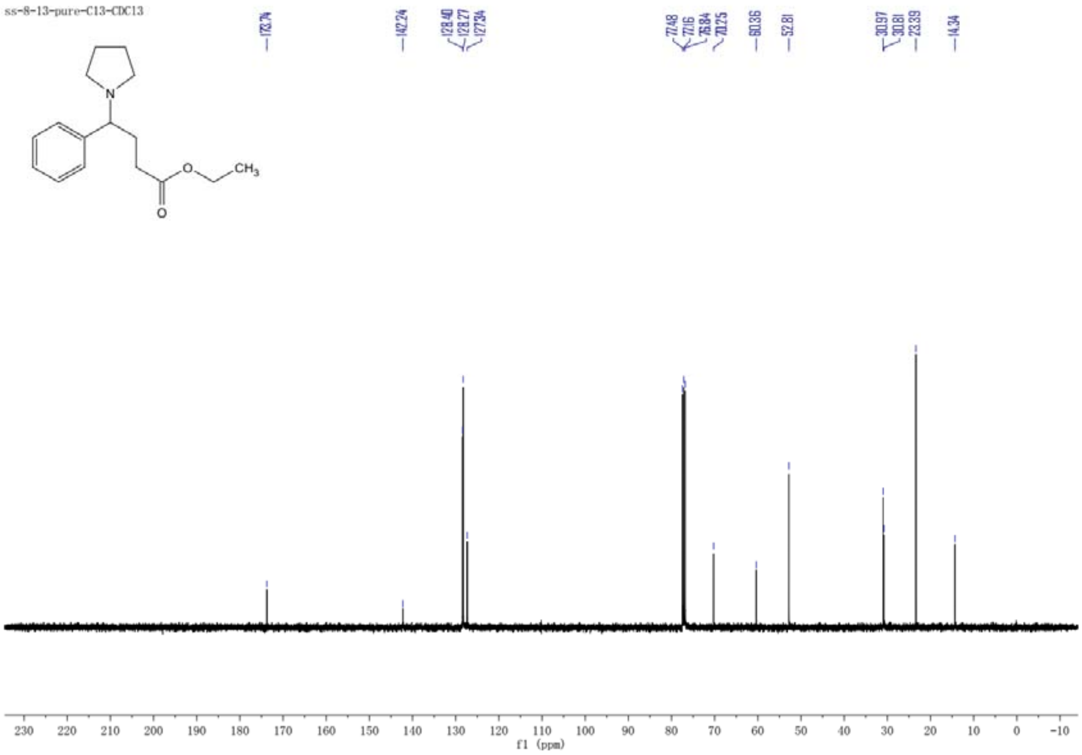
ss-8-13-pure-C13-CDCl3
173.74
142.24
128.40
128.27
127.34
77.48
77.16
76.84
70.25
60.36
52.81
30.97
30.81
23.39
14.34
f1 (ppm)

55

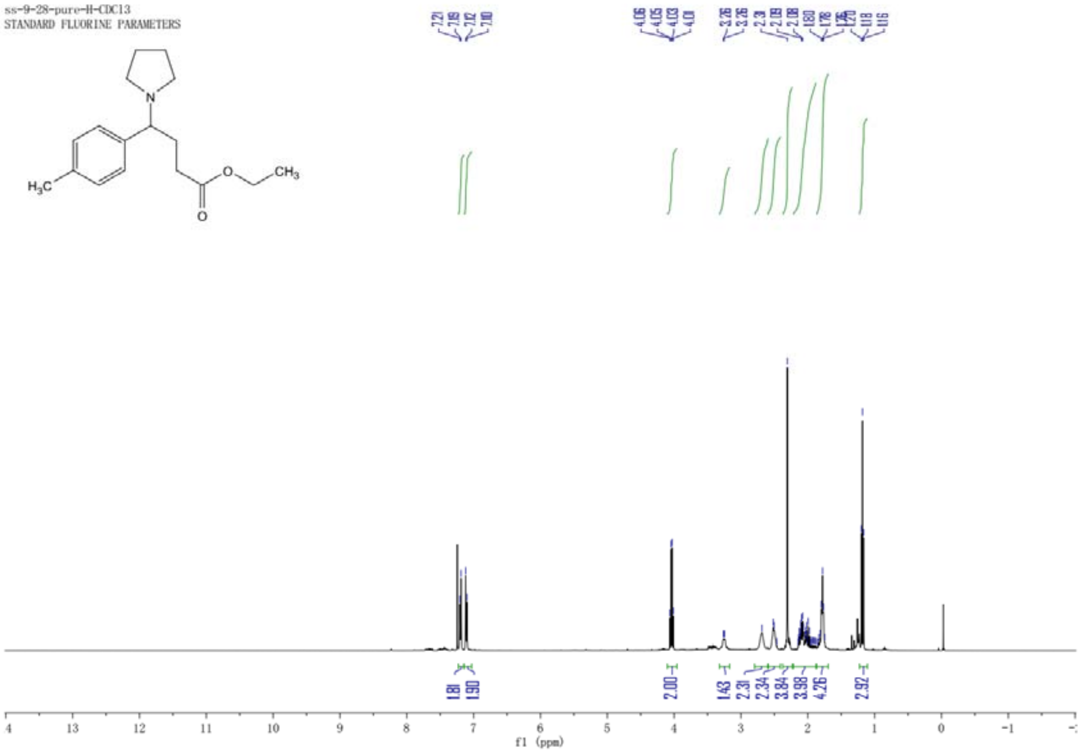
ss-9-28-pure-H-CDCl3
STANDARD FLUORINE PARAMETERS
1.81
1.90
2.00
1.43
2.31
2.34
3.84
3.98
4.26
2.92
f1 (ppm)

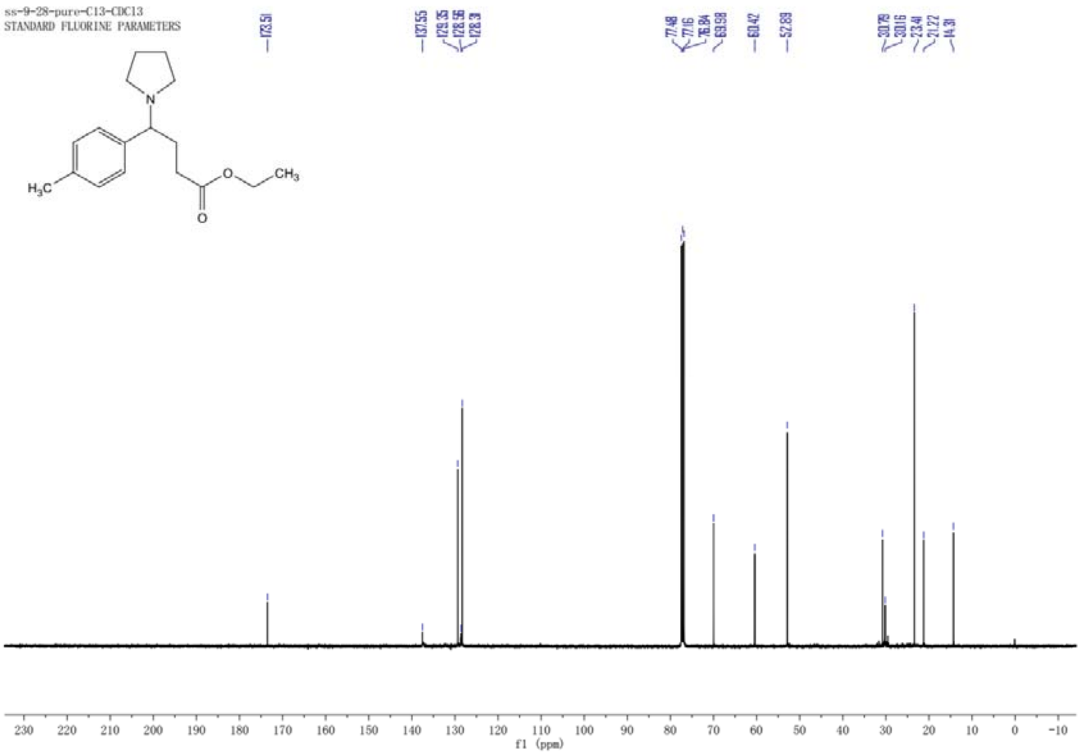
ss-9-28-pure-C13-CDCl3
STANDARD FLUORINE PARAMETERS
173.51
137.55
129.35
128.56
128.31
77.48
77.16
76.84
69.98
60.42
52.89
30.79
30.16
23.41
21.22
14.31
N
O
O
H3C
CH3
230 220 210 200 190 180 170 160 150 140 130 120 110 100 90 80 70 60 50 40 30 20 10 0 -10
f1 (ppm)

56

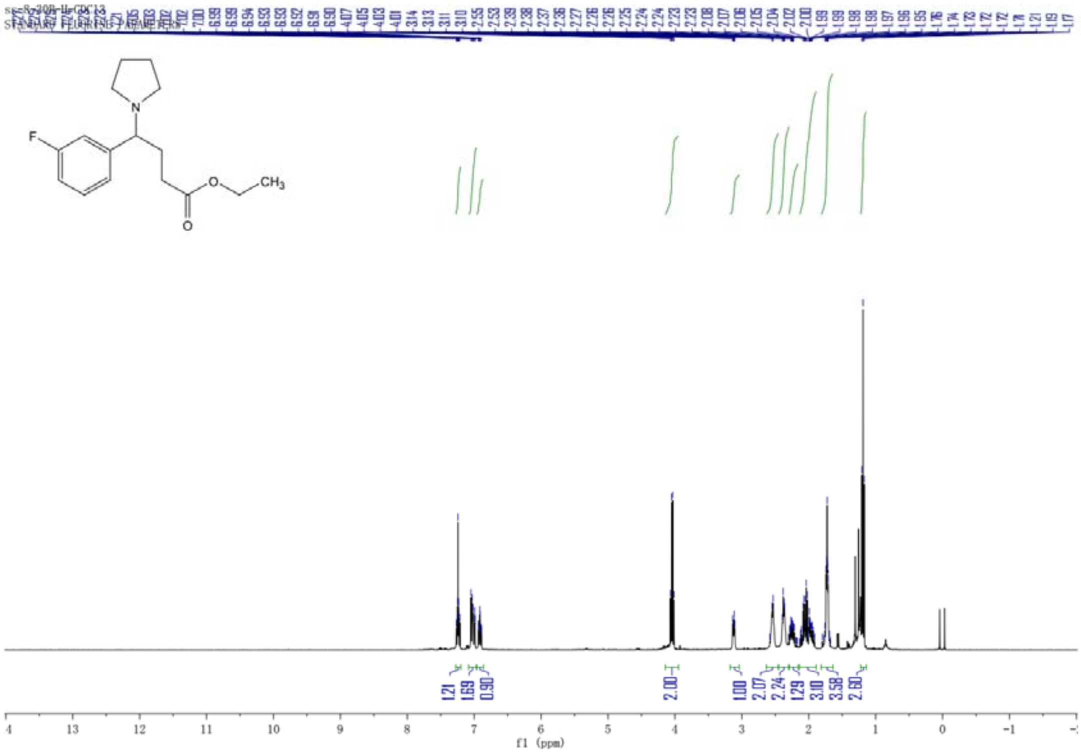
N
F
O
O
CH3
1.21
1.69
0.90
2.00
1.00
2.07
2.24
1.29
3.10
3.58
2.60
13 12 11 10 9 8 7 6 5 4 3 2 1 0 -1
f1 (ppm)

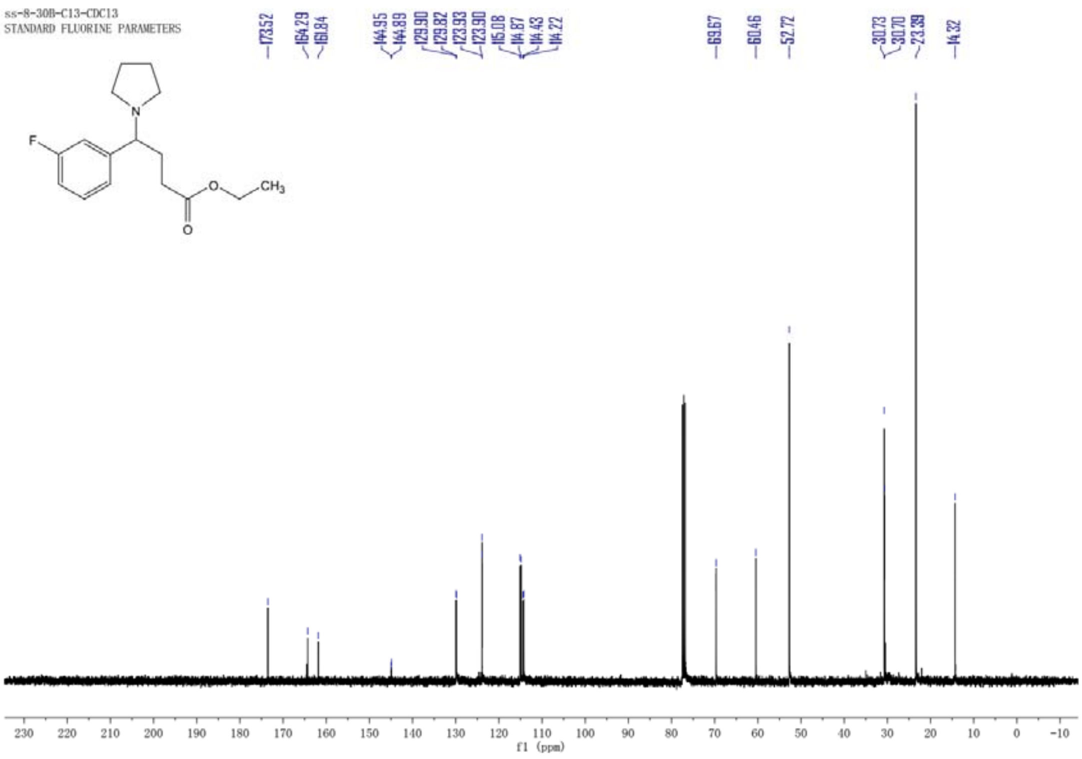
ss-8-30B-C13-CDCl3
STANDARD FLUORINE PARAMETERS
173.52
164.29
161.84
144.95
144.89
129.90
129.82
123.93
123.90
115.08
114.87
114.43
114.22
69.67
60.46
52.72
30.73
30.70
23.38
14.32
F
N
O
CH3
O
f1 (ppm)

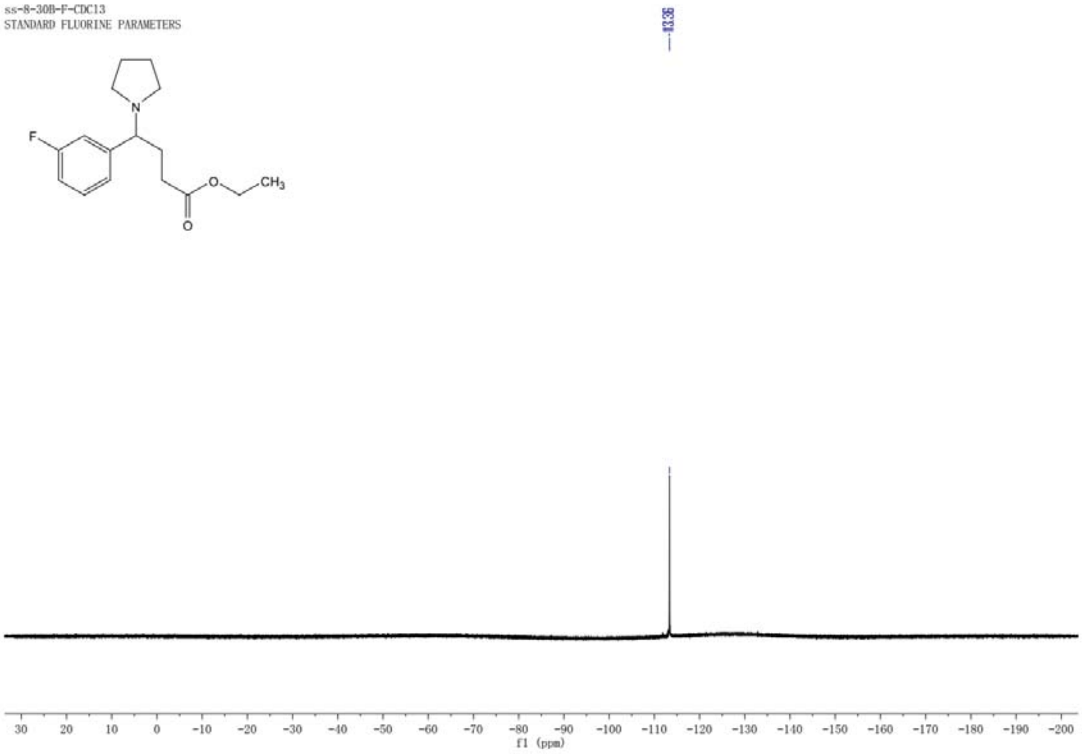
ss-8-30B-F-CDCl3
STANDARD FLUORINE PARAMETERS
-113.36
F
N
O
CH3
O
f1 (ppm)

57

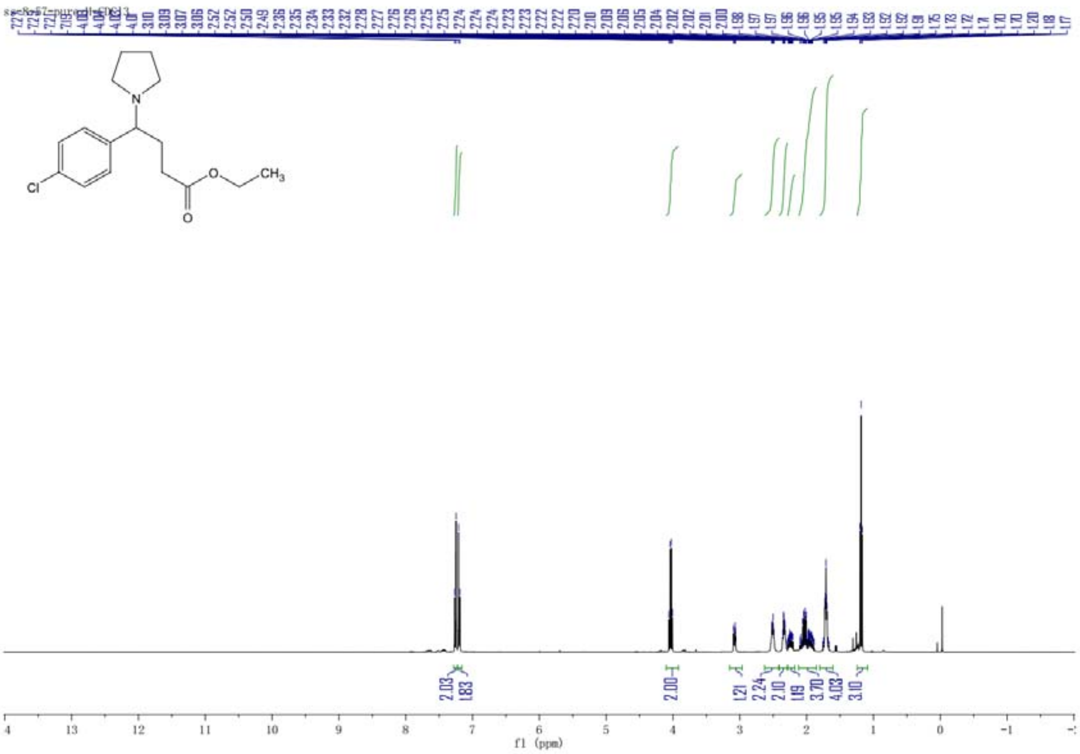
Cl
N
O
O
CH3
2.03
1.83
2.00
1.21
2.24
2.10
1.19
3.70
4.03
3.10
f1 (ppm)

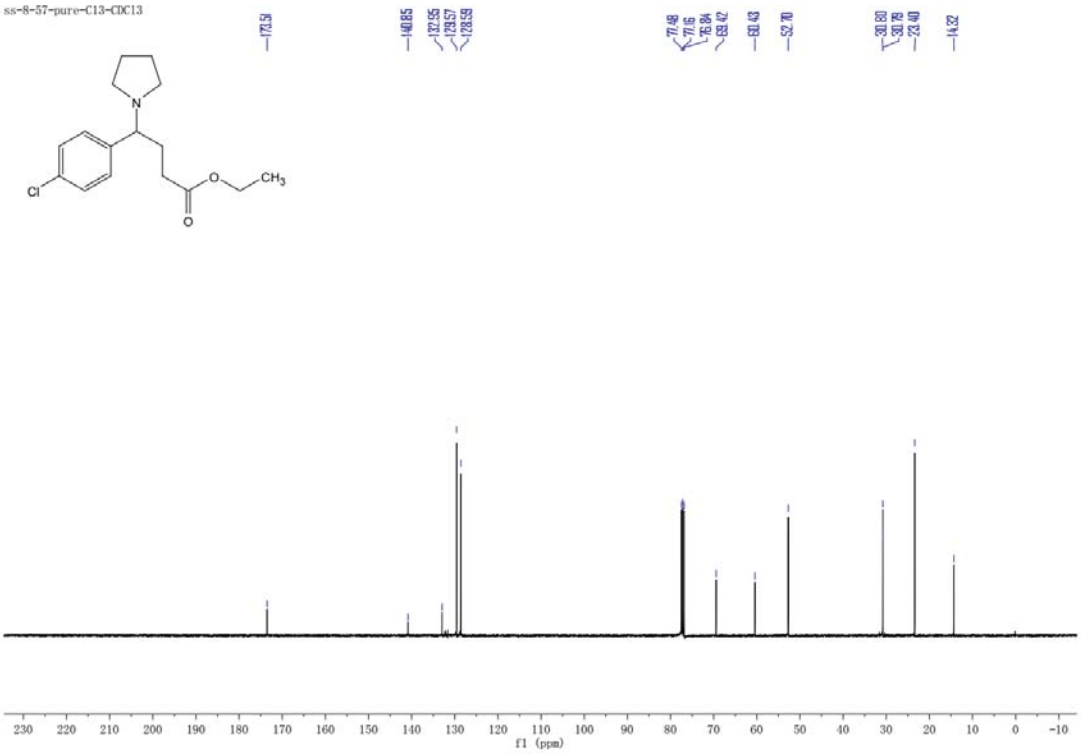
ss-8-57-pure-C13-CDCl3
173.51
140.85
132.55
129.57
128.59
77.48
77.16
76.84
69.42
60.43
52.70
30.80
30.79
23.40
14.32
Cl
N
O
O
CH3
f1 (ppm)

58

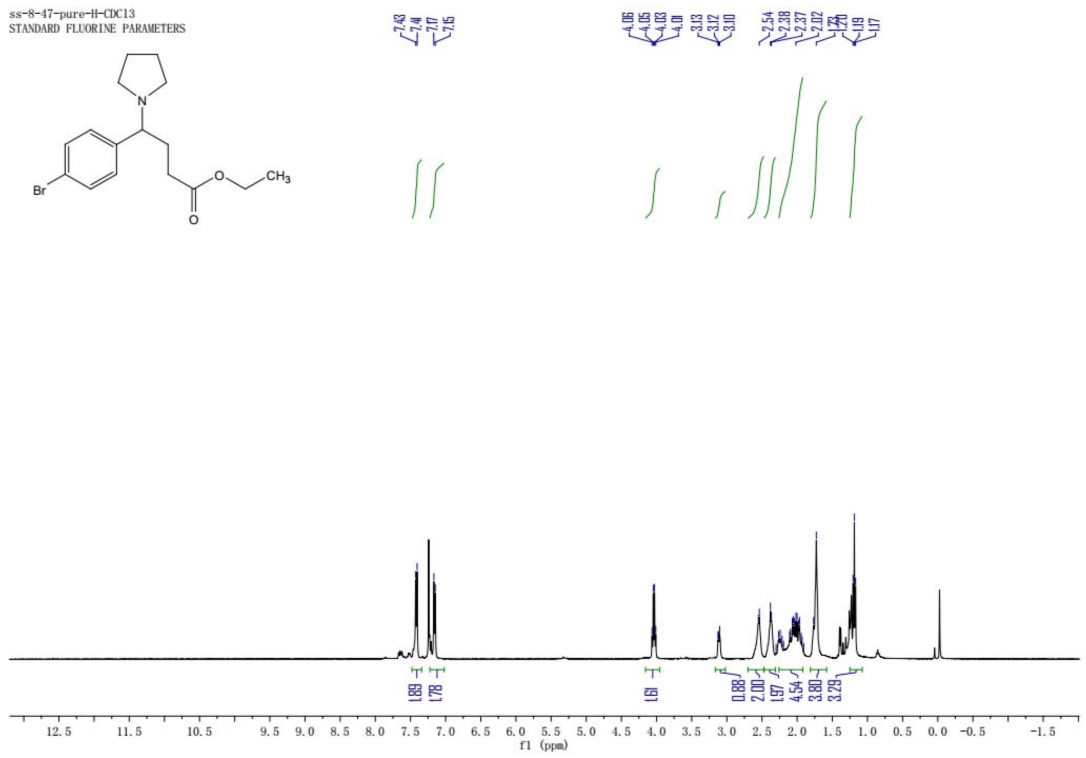
ss-8-47-pure-H-CDCl3
STANDARD FLUORINE PARAMETERS
7.43
7.41
7.17
7.15
4.06
4.05
4.03
4.01
3.13
3.12
3.10
2.54
2.38
2.37
2.02
1.19
1.17
Br
N
O
CH3
1.89
1.78
1.61
0.88
2.00
1.97
4.54
3.80
3.29
f1 (ppm)

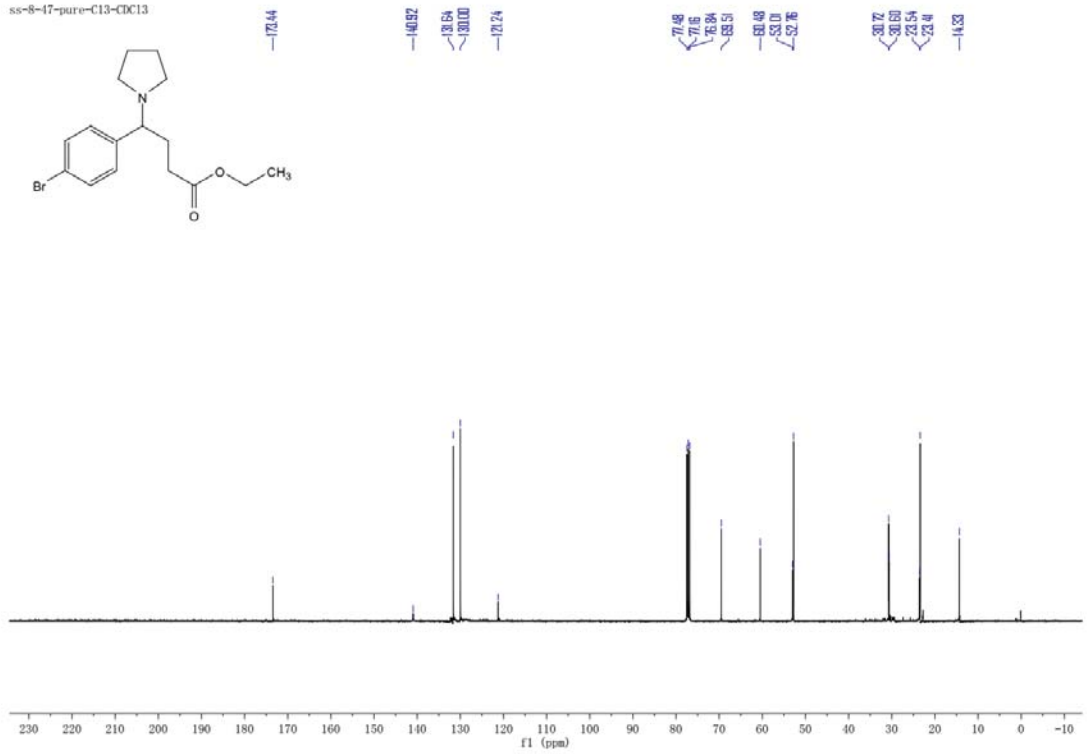
ss-8-47-pure-C13-CDCl3
173.44
140.92
131.64
130.00
121.24
77.48
77.16
76.84
69.51
60.48
53.01
52.76
30.72
30.60
23.54
23.41
14.33
Br
N
O
CH3
f1 (ppm)

59

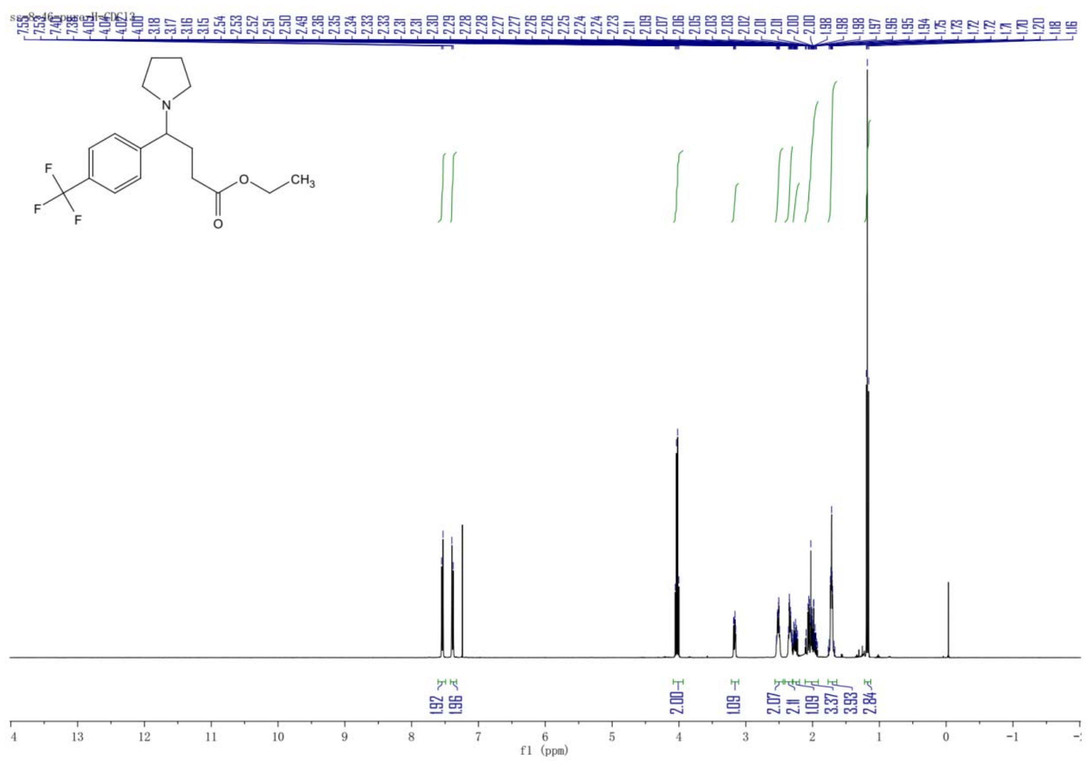
ss-8-46-pure-H-CDCl3
f1 (ppm)

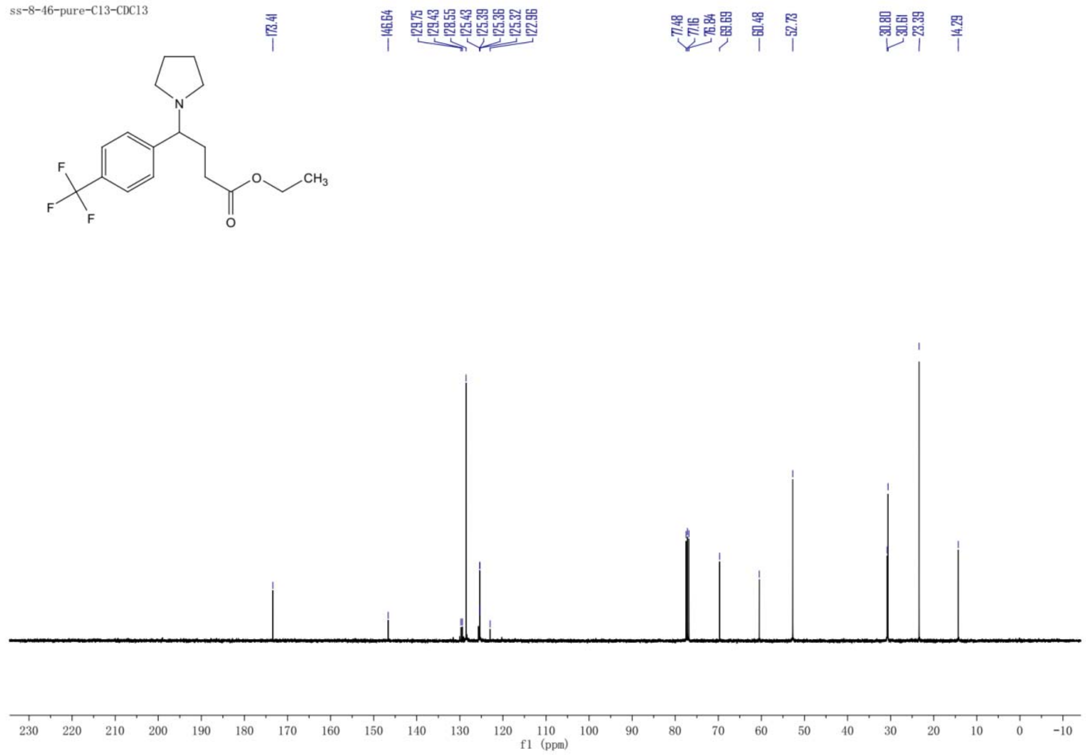
ss-8-46-pure-C13-CDCl3
173.41
146.64
129.75
129.43
128.55
125.43
125.39
125.36
125.32
122.96
77.48
77.16
76.84
69.69
60.48
52.73
30.80
30.61
23.39
14.29
f1 (ppm)

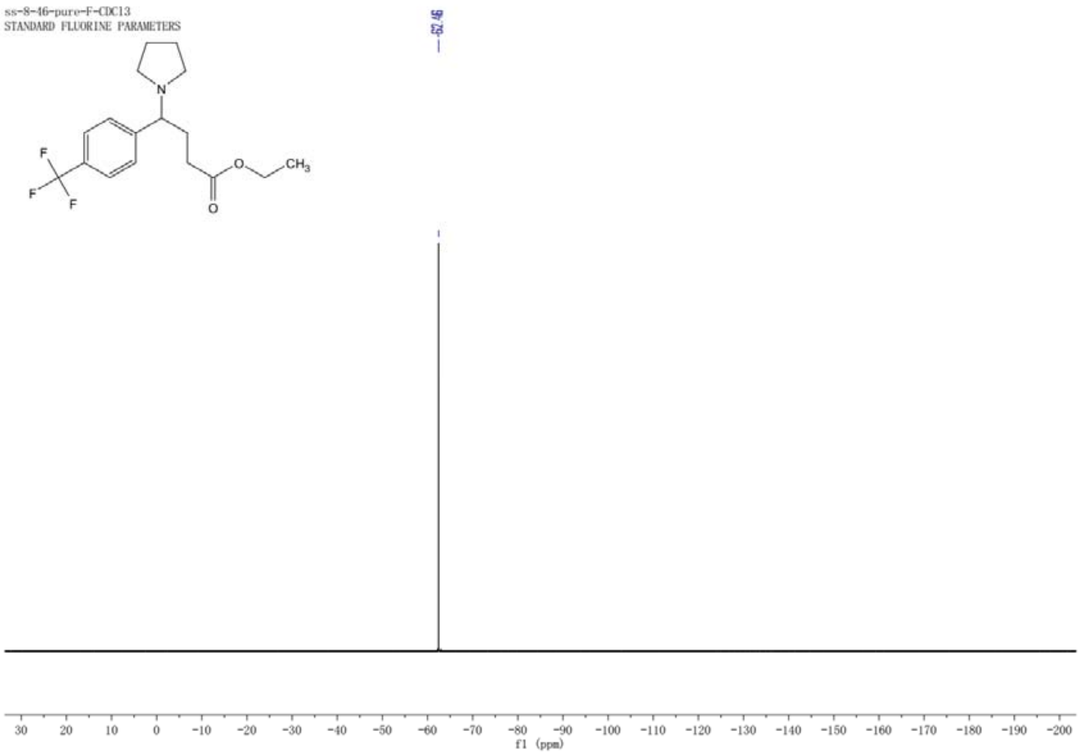
ss-8-46-pure-F-CDCl3
STANDARD FLUORINE PARAMETERS
-62.46
f1 (ppm)

60

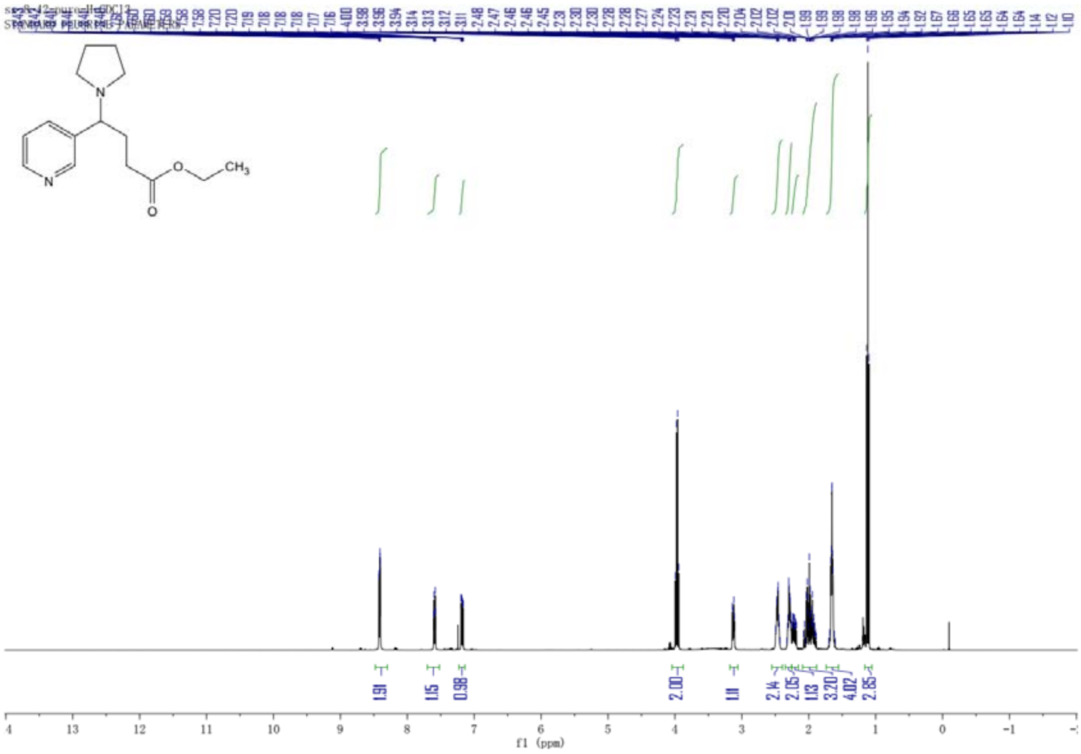
1.91
1.15
0.98
2.00
1.11
2.14
2.05
1.13
3.20
4.02
2.85
f1 (ppm)

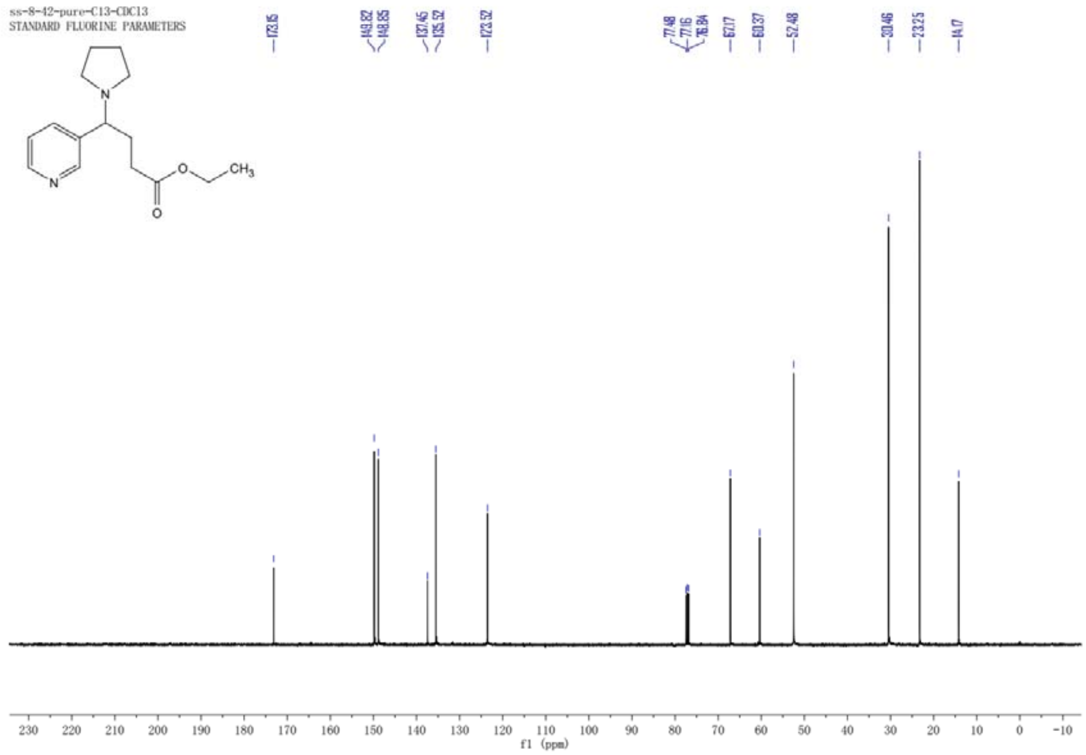
ss-8-42-pure-C13-CDC13
STANDARD FLUORINE PARAMETERS
173.15
149.82
148.85
137.45
135.52
123.52
77.48
77.16
76.84
67.17
60.37
52.48
30.46
23.25
14.17
f1 (ppm)

61

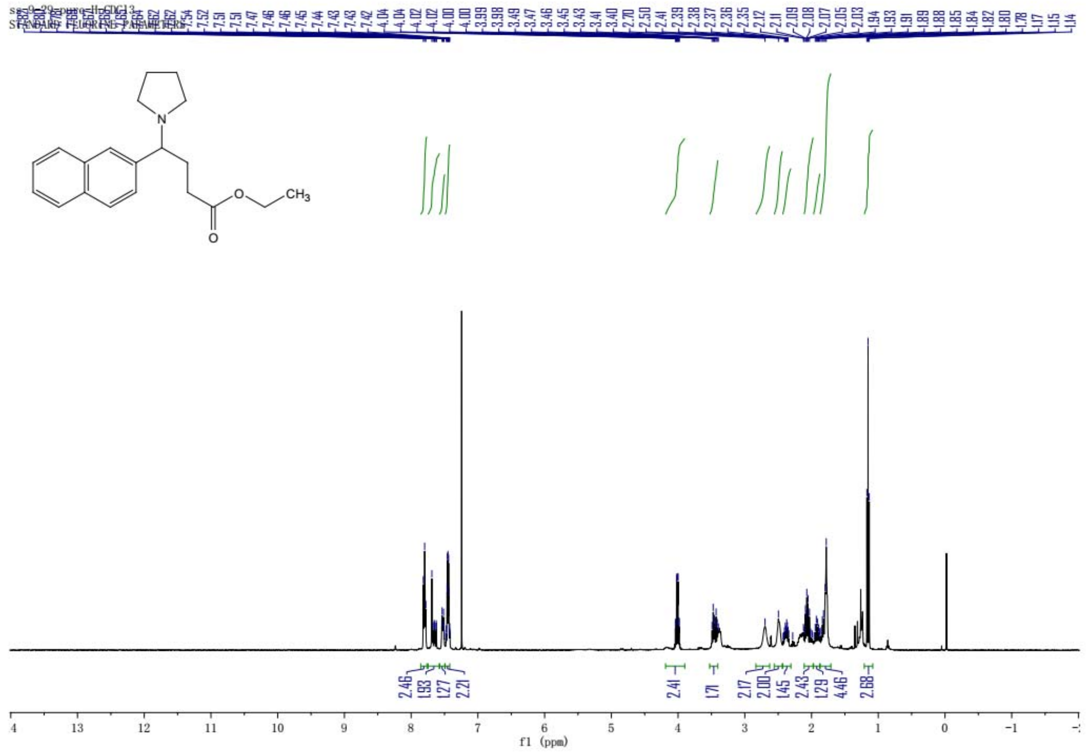
2.46
1.93
1.27
2.21
2.41
1.71
2.17
2.00
1.45
2.43
1.29
4.46
2.68
f1 (ppm)

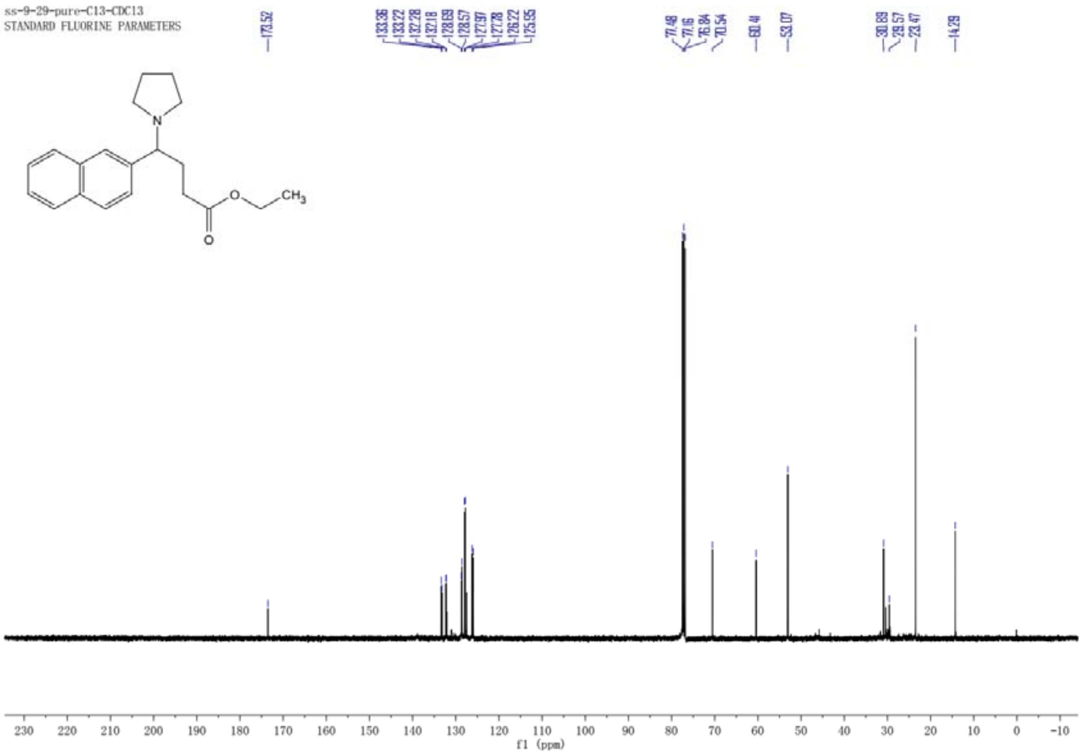
ss-9-29-pure-C13-CDC13
STANDARD FLUORINE PARAMETERS
173.52
133.36
133.22
132.28
132.18
128.69
128.57
127.97
127.78
126.22
125.95
77.48
77.16
76.84
70.54
60.41
53.07
30.89
29.57
23.47
14.29
N
O
CH3
f1 (ppm)

62

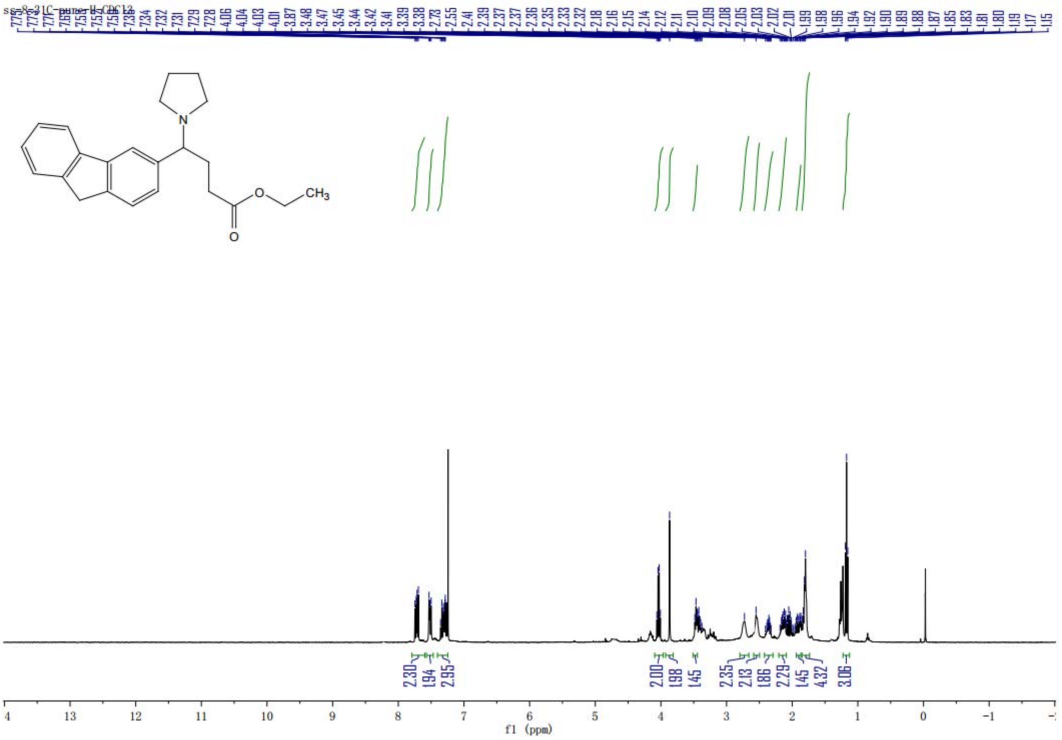
N
O
CH3
f1 (ppm)

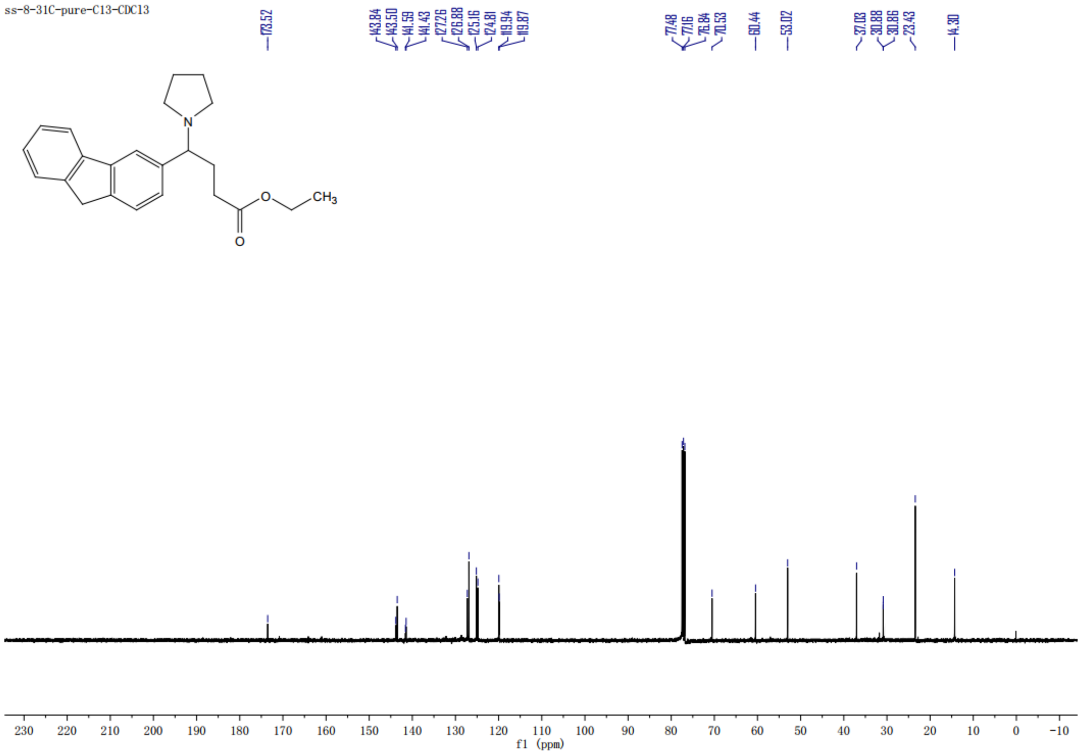
ss-8-31C-pure-C13-CDCl3
f1 (ppm)

63

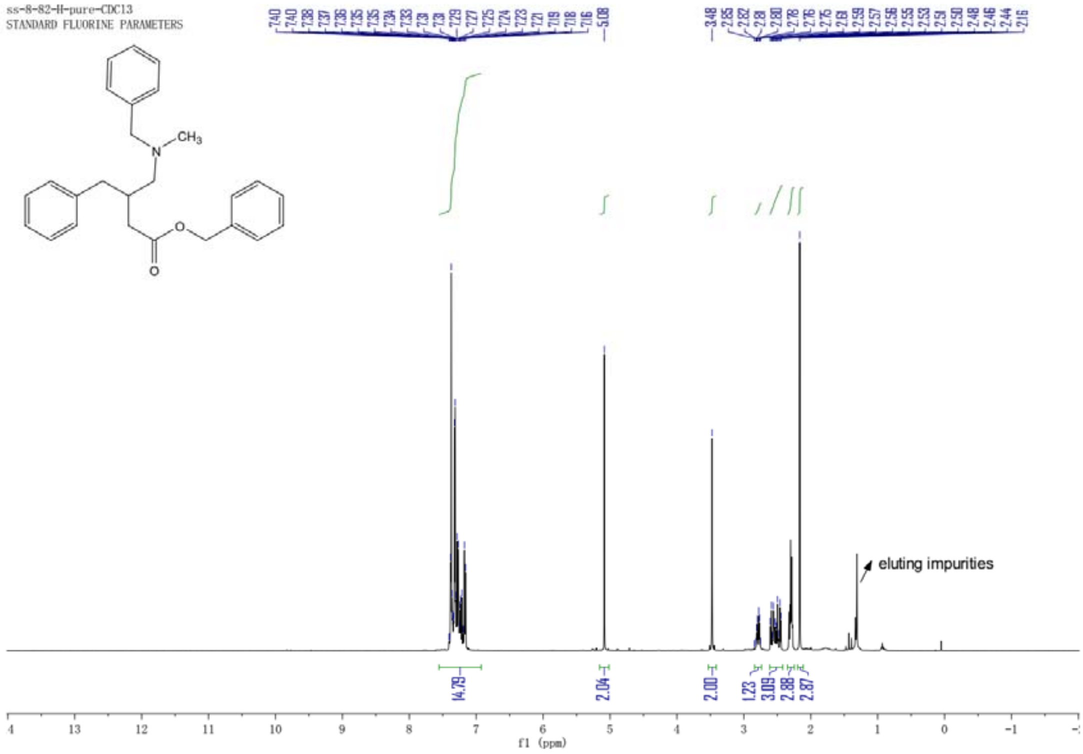
ss-8-82-H-pure-CDCl3
STANDARD FLUORINE PARAMETERS
eluting impurities
f1 (ppm)

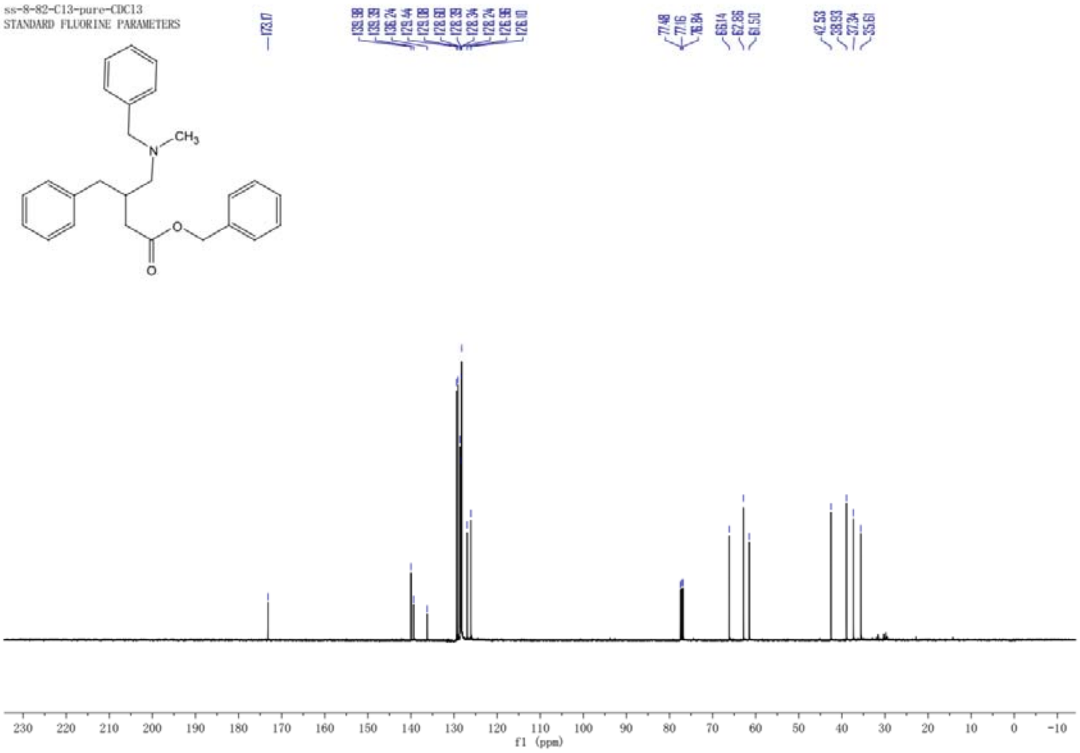
ss-8-82-C13-pure-CDCl3
STANDARD FLUORINE PARAMETERS
173.17
139.98
139.39
136.24
129.44
129.08
128.60
128.39
128.34
128.24
126.96
126.10
77.48
77.16
76.84
66.14
62.86
61.50
42.53
38.93
37.34
35.61
f1 (ppm)

63

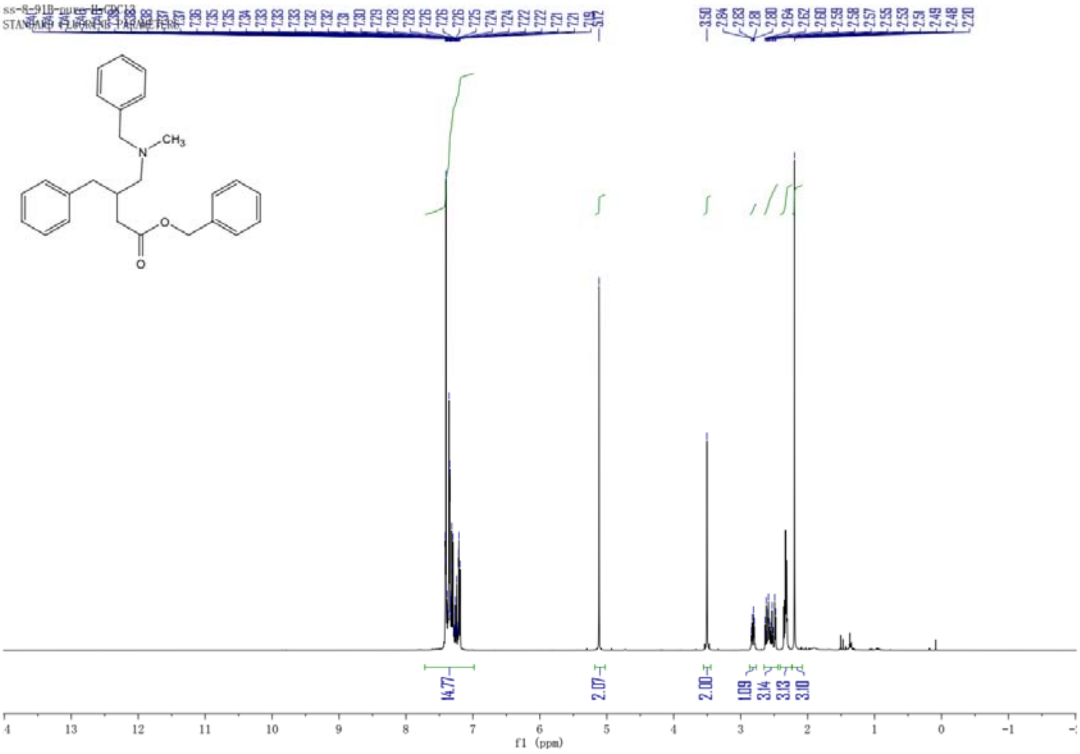
STANDARD FLUORINE PARAMETERS
14.77
2.07
2.00
1.09
3.14
3.13
3.10
f1 (ppm)

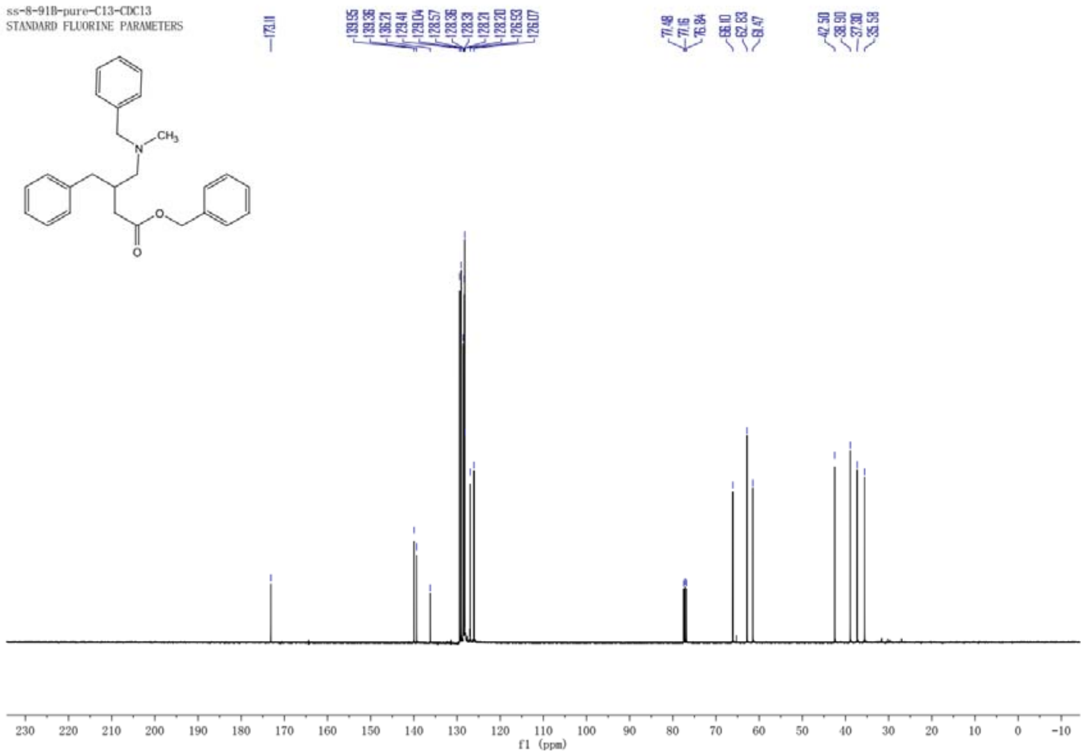

64

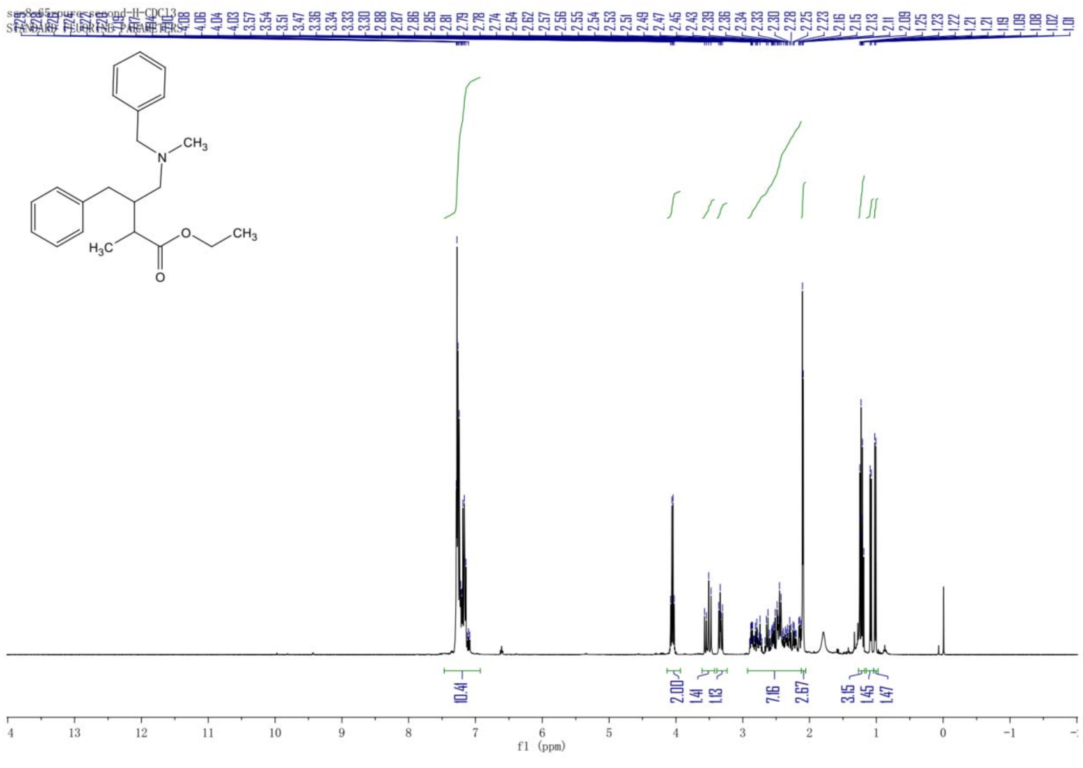

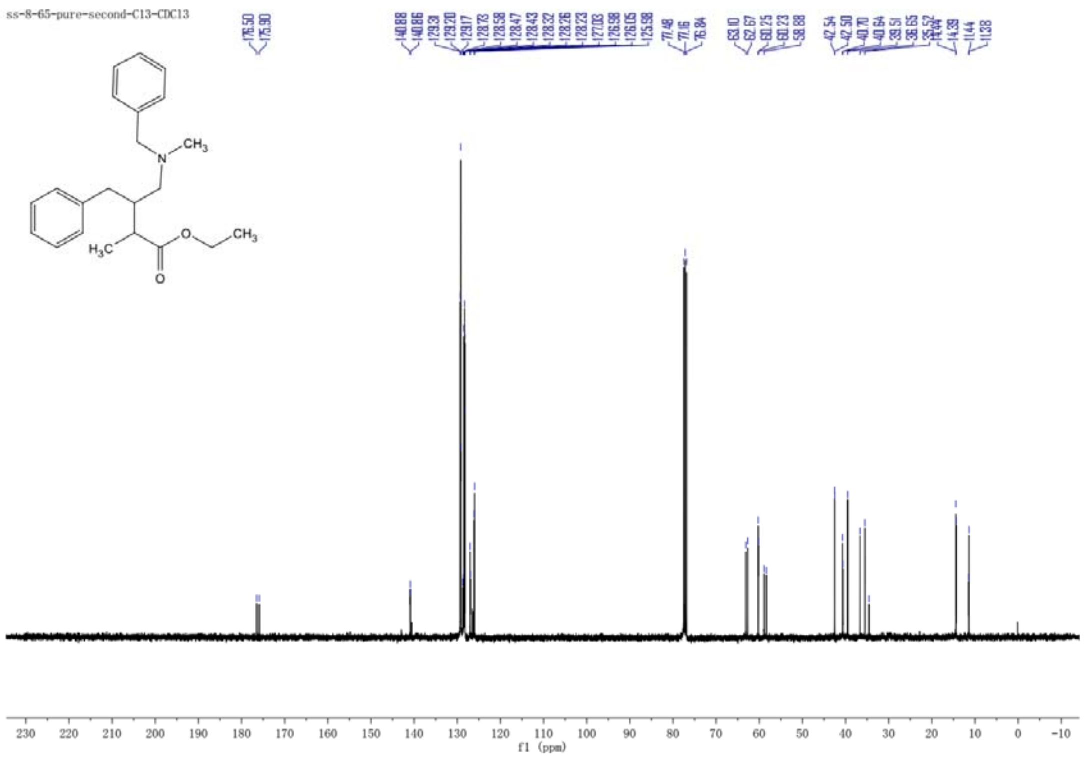

65

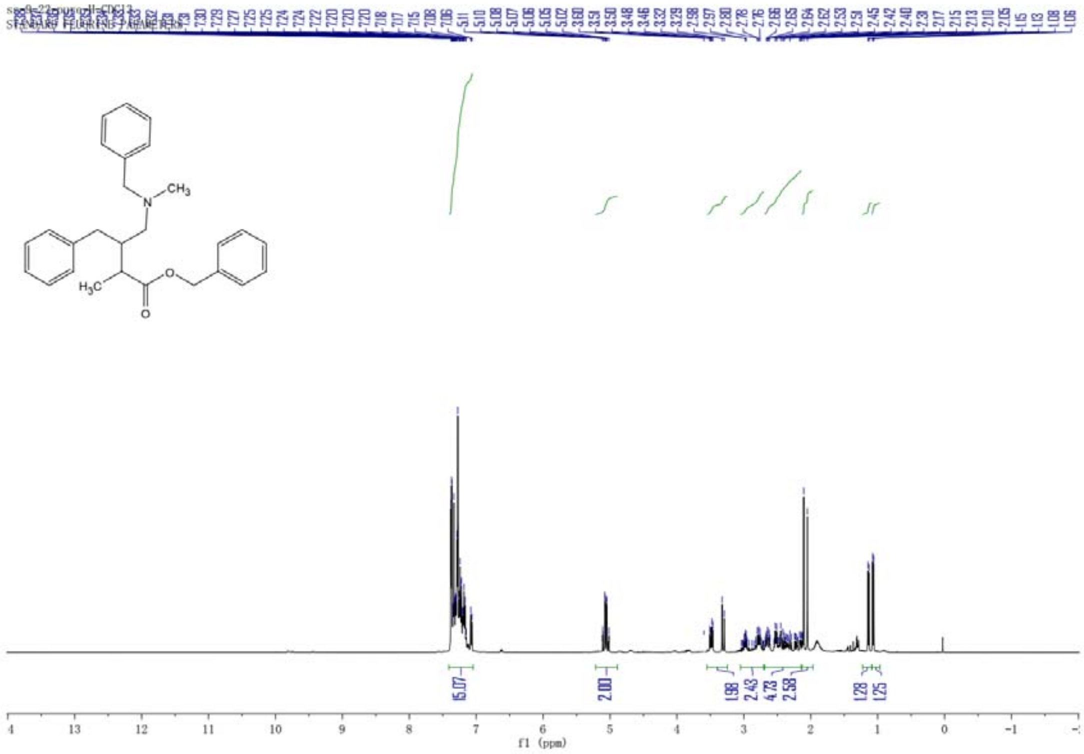

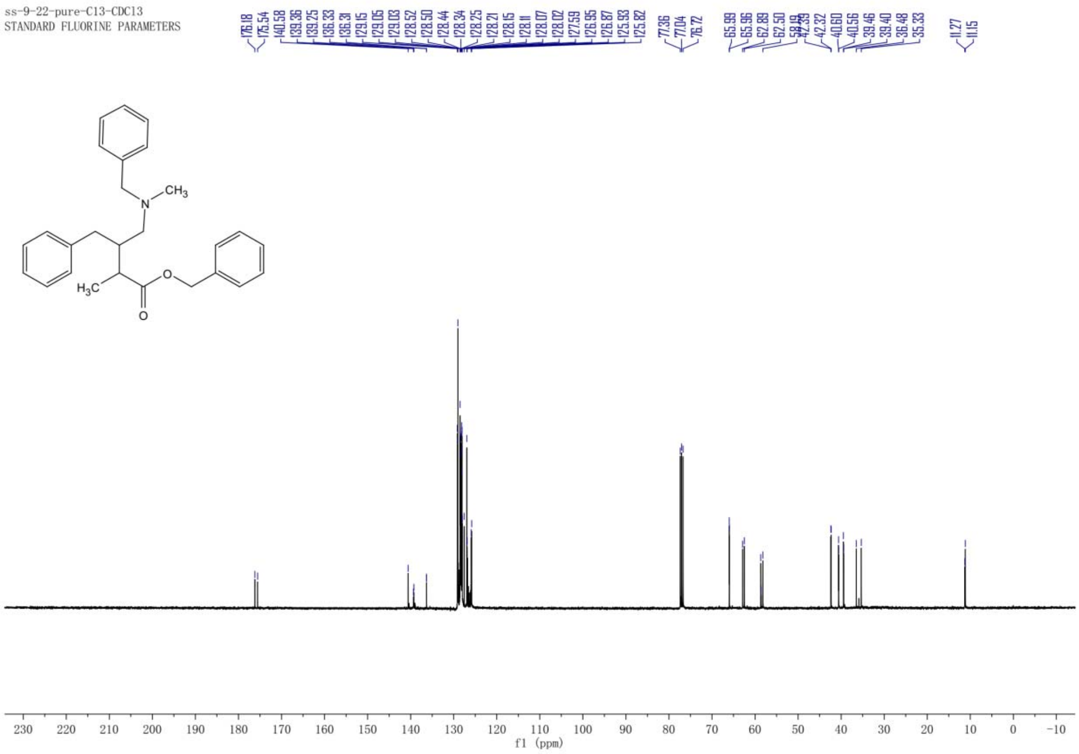
ss-9-22-pure-C13-CDCl3
STANDARD FLUORINE PARAMETERS
f1 (ppm)

66

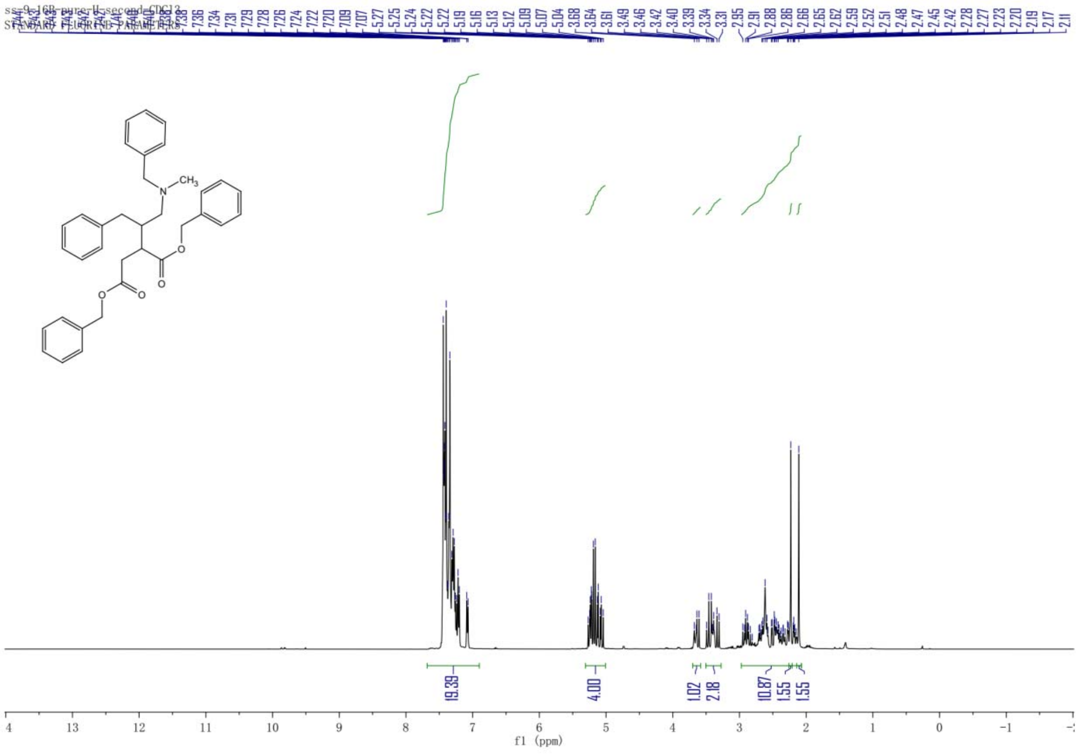
f1 (ppm)

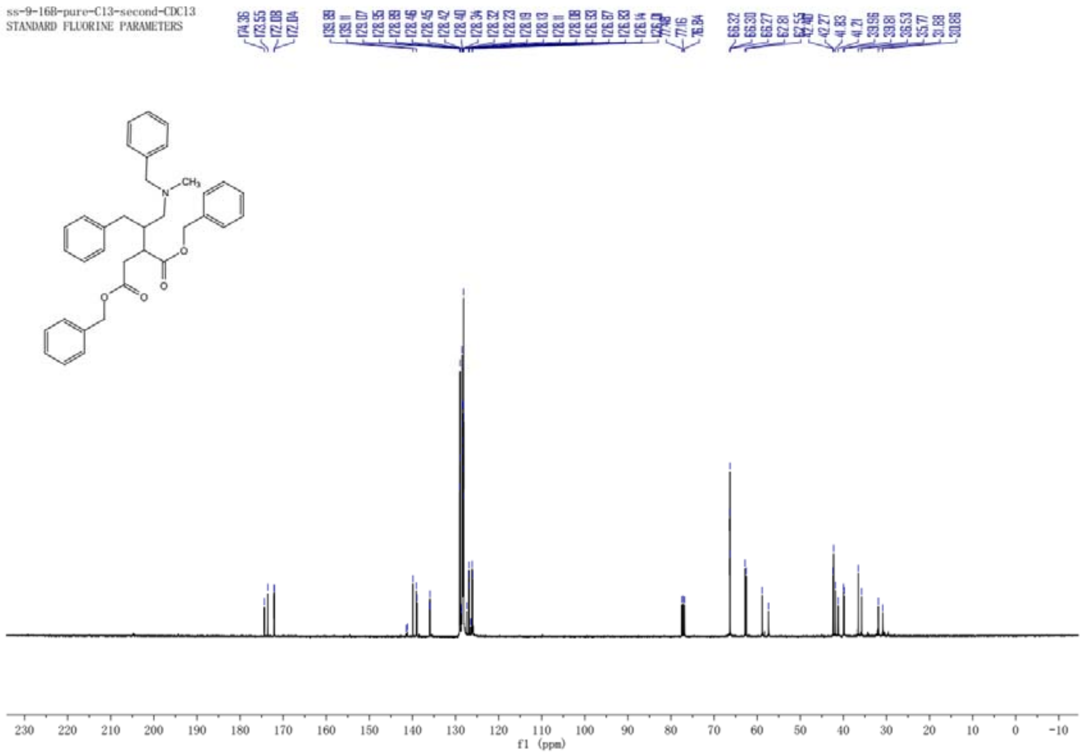

67

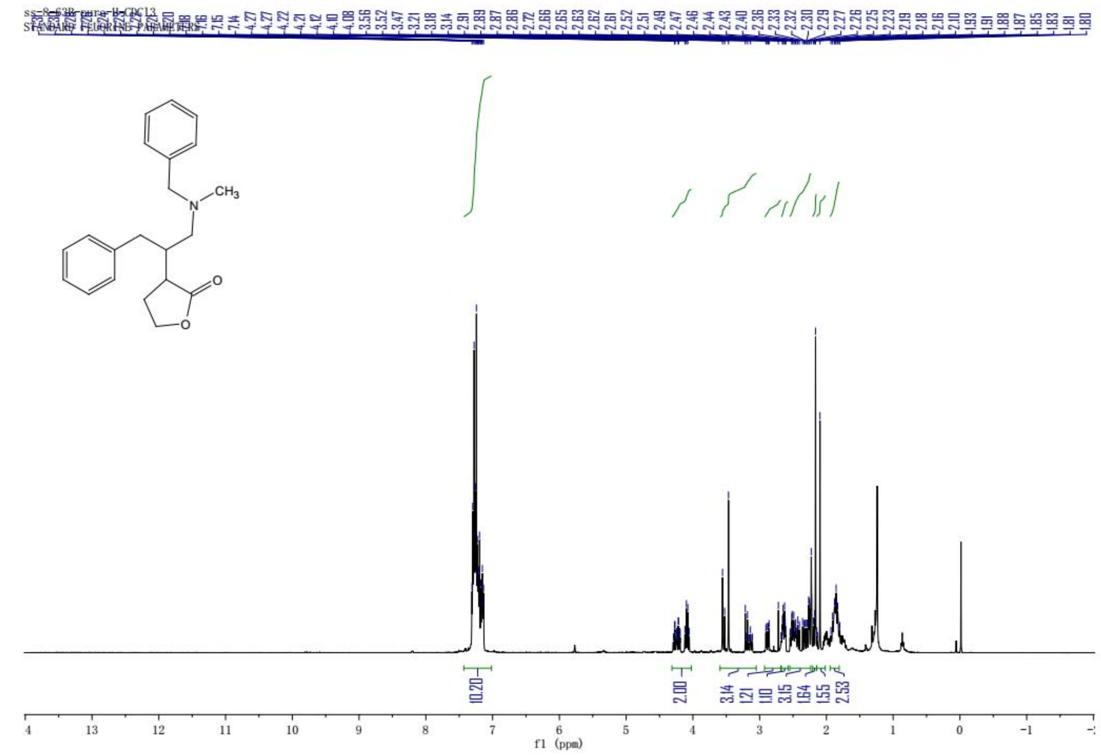

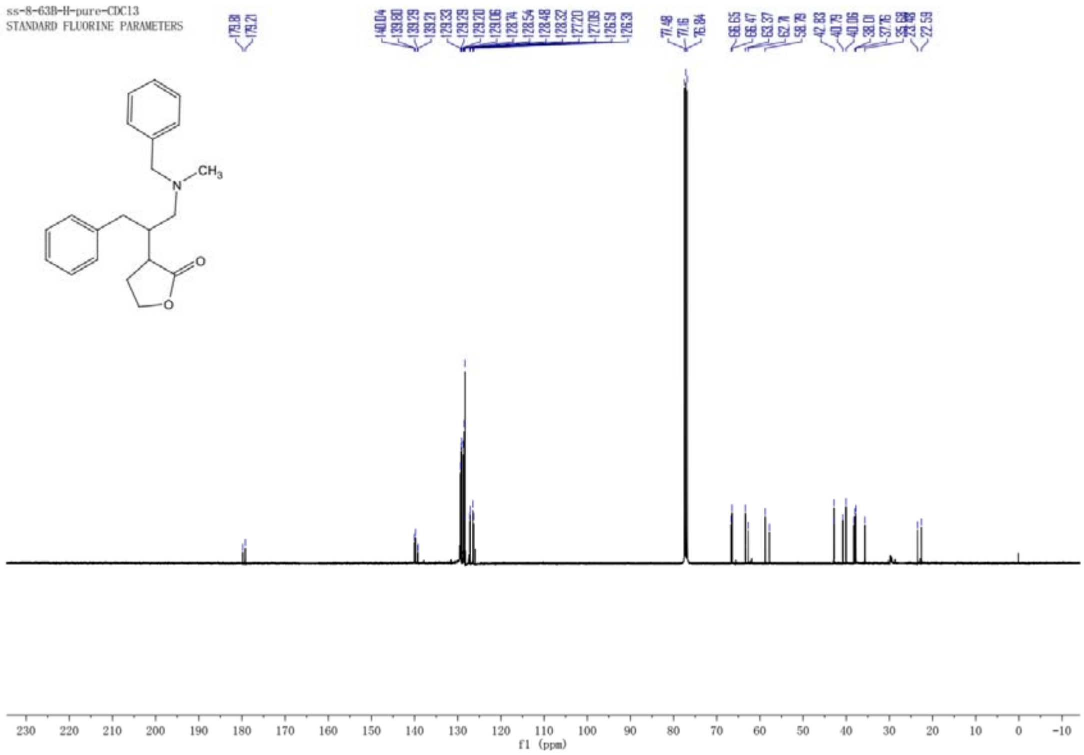
ss-8-63B-H-pure-CDCl3
STANDARD FLUORINE PARAMETERS
f1 (ppm)

68

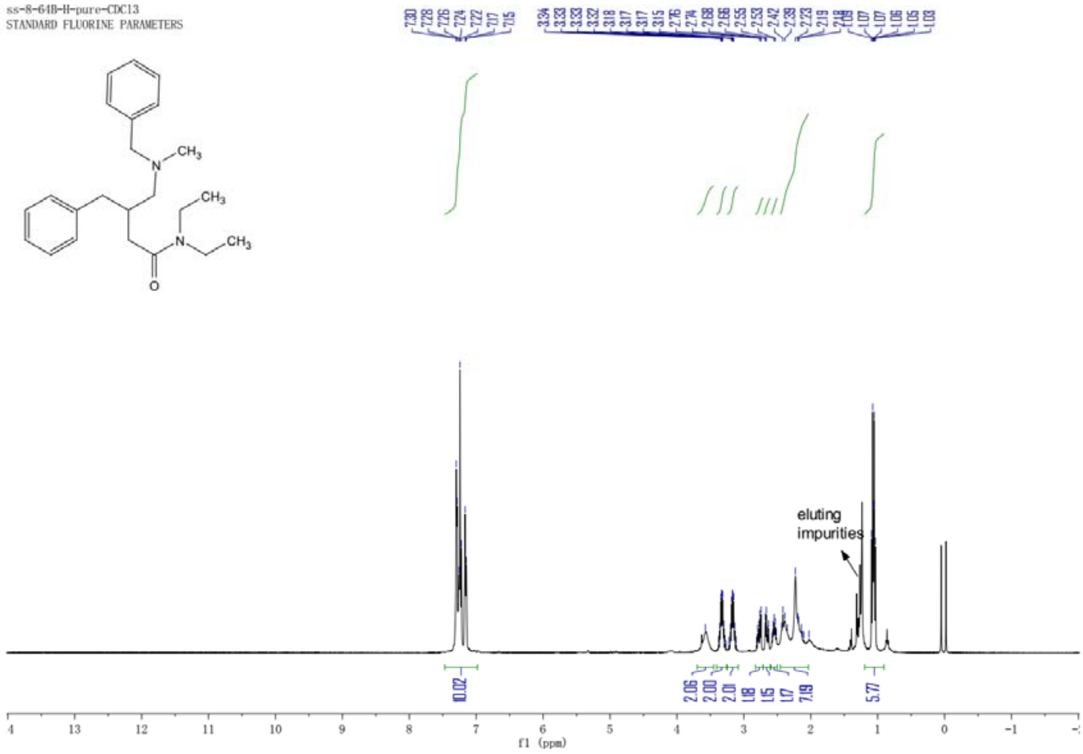
ss-8-64B-H-pure-CDCl3
STANDARD FLUORINE PARAMETERS
eluting
impurities
f1 (ppm)

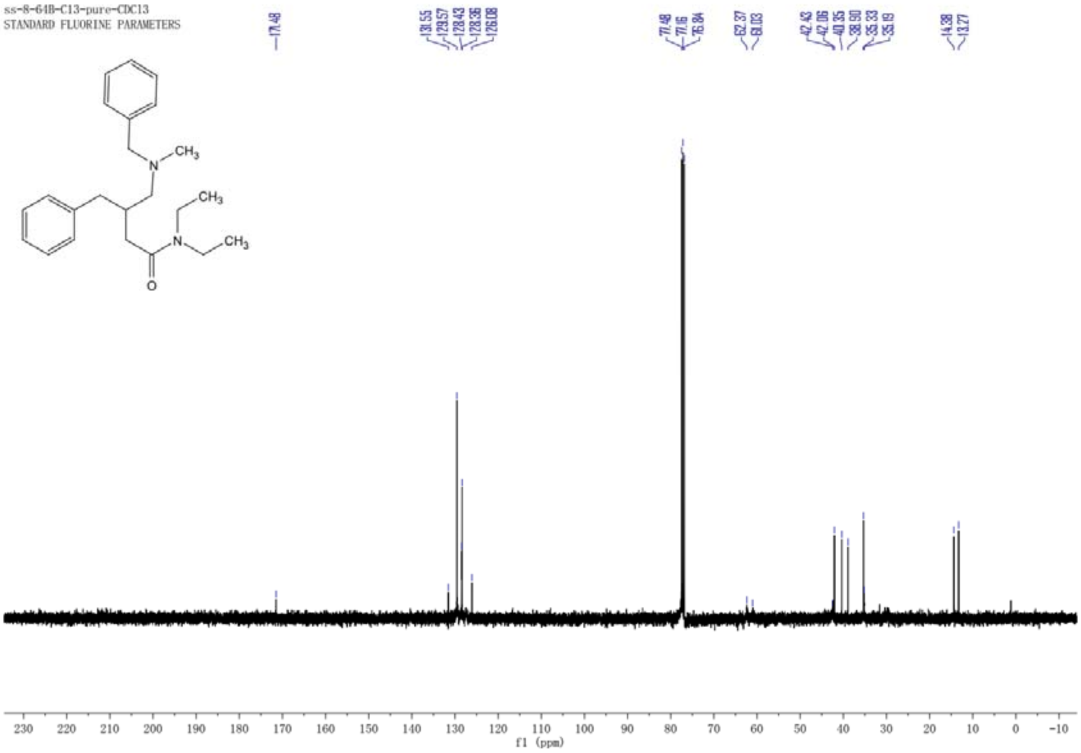
ss-8-64B-C13-pure-CDCl3
STANDARD FLUORINE PARAMETERS
CH3
N
O
f1 (ppm)

69

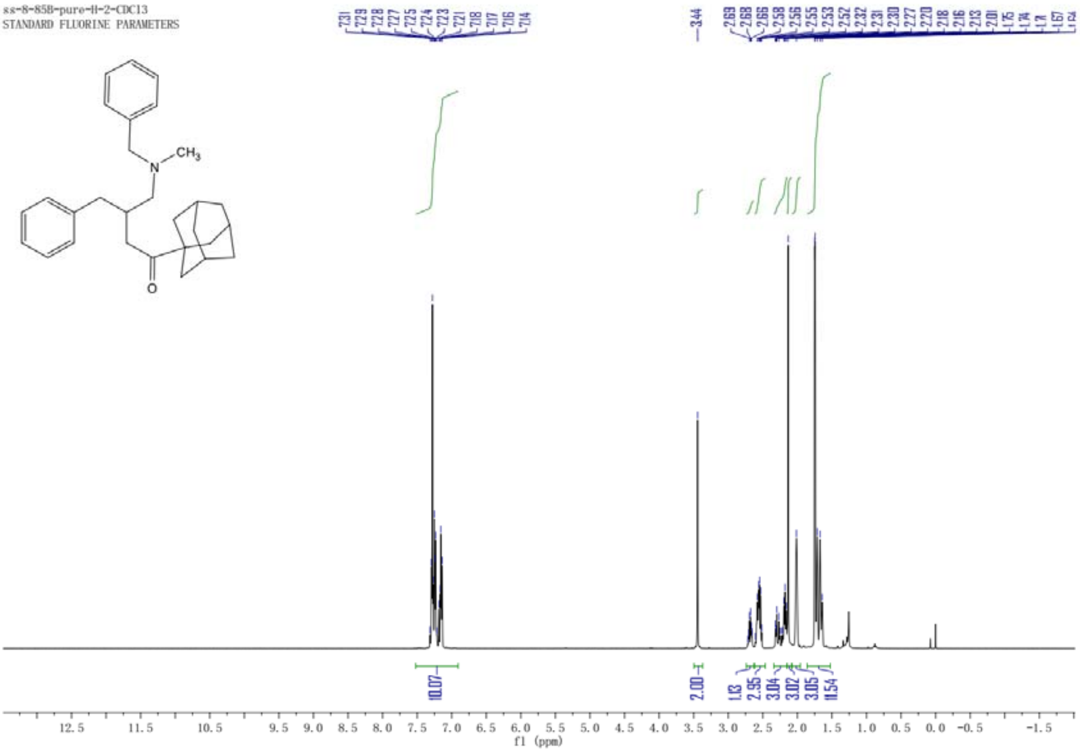
ss-8-85B-pure-H-2-CDCl3
STANDARD FLUORINE PARAMETERS
N
CH3
O
f1 (ppm)

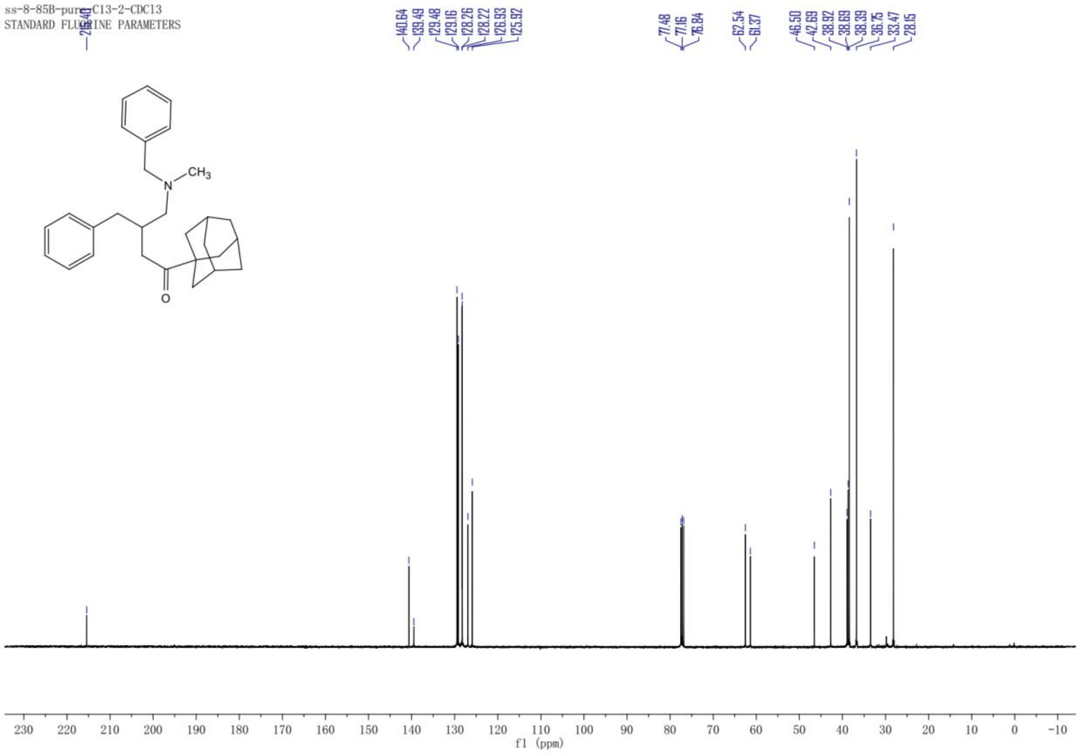

70

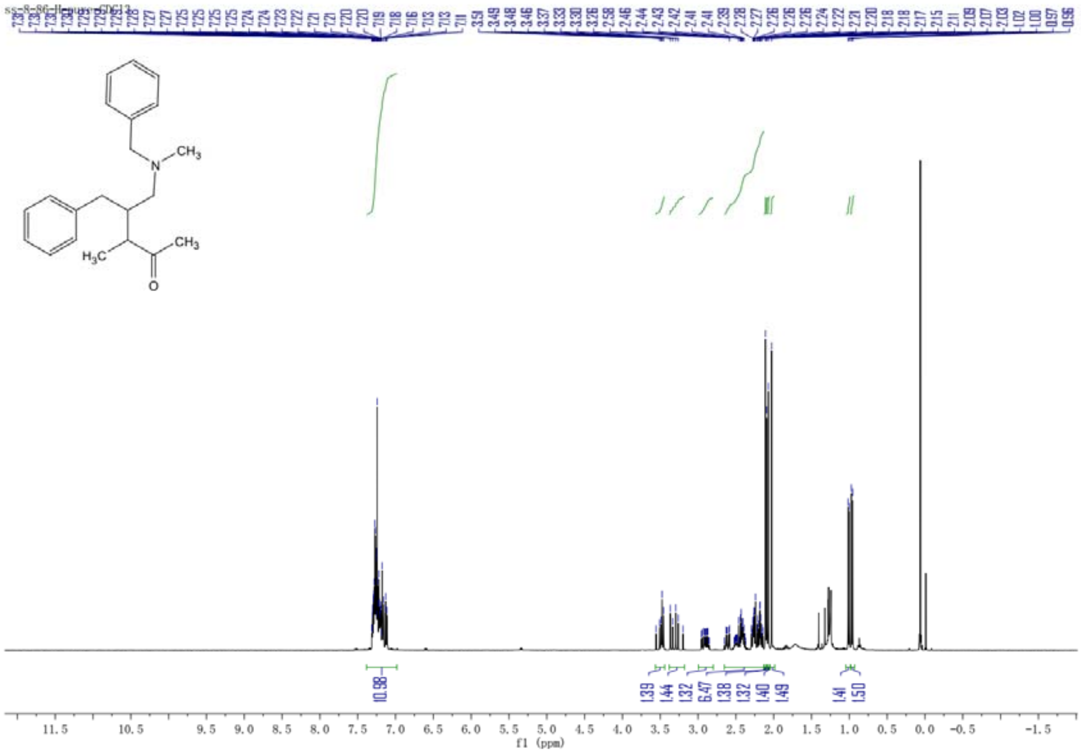

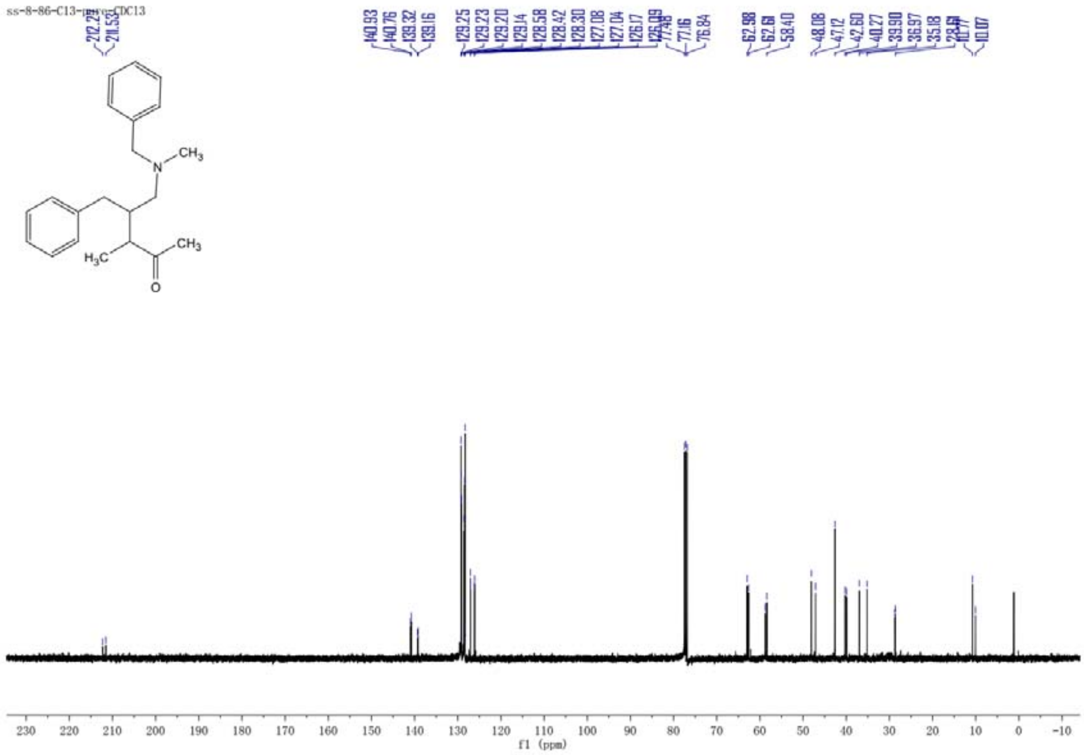

71

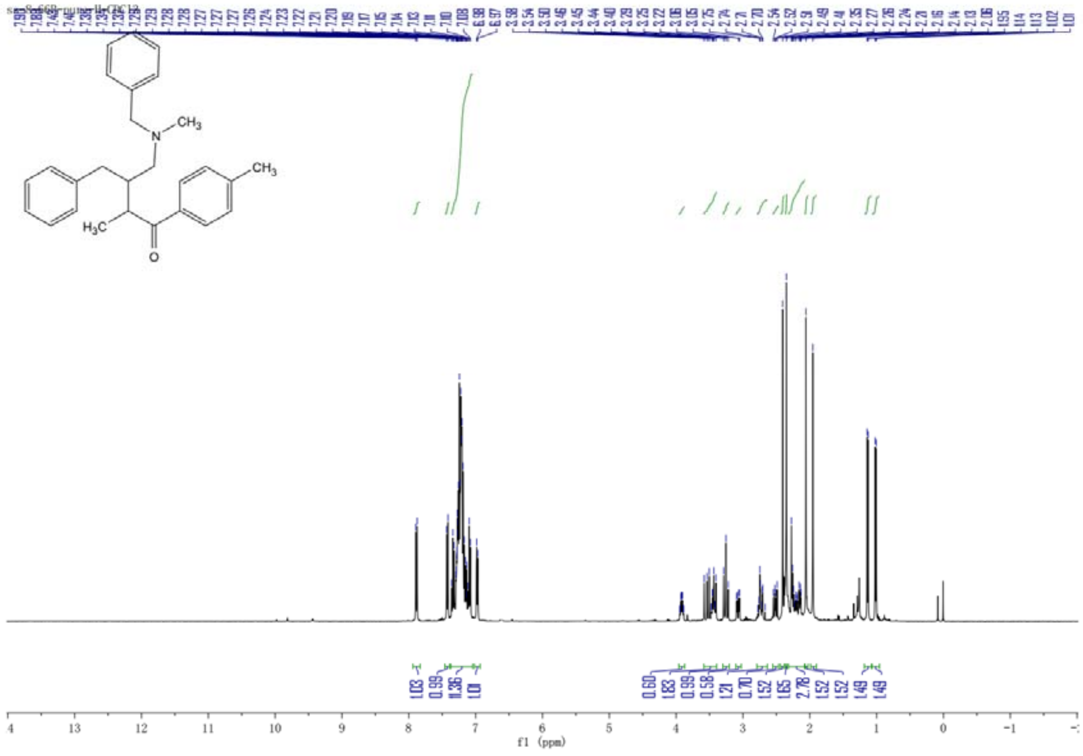

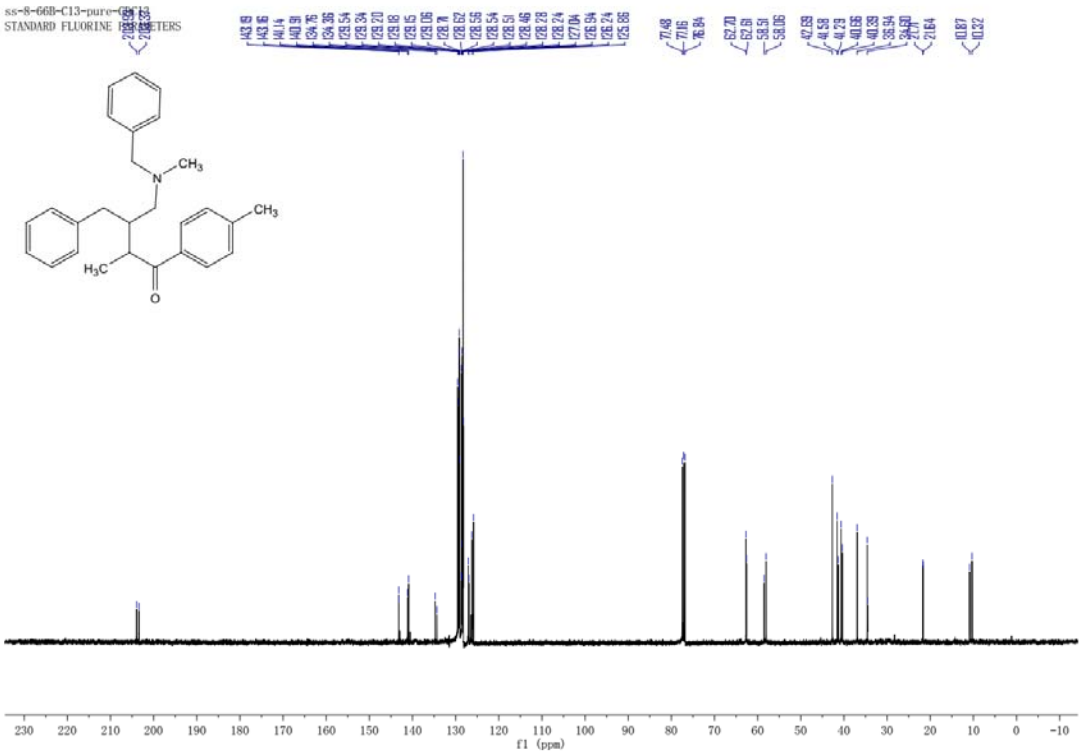

72

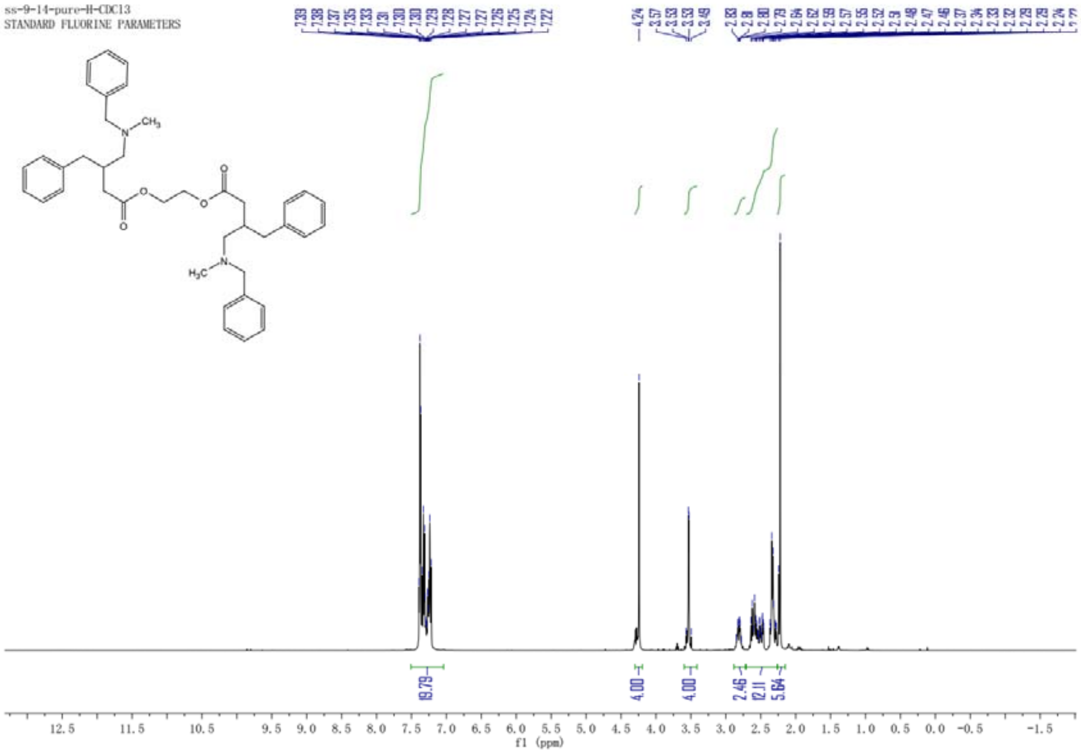

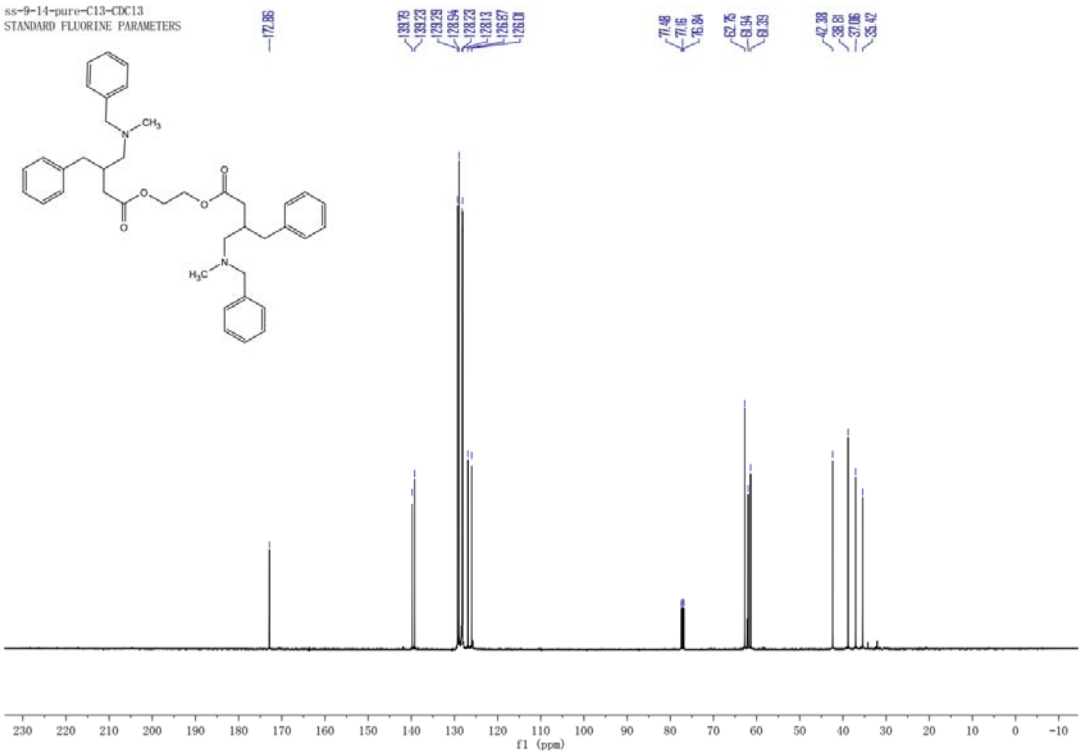

73

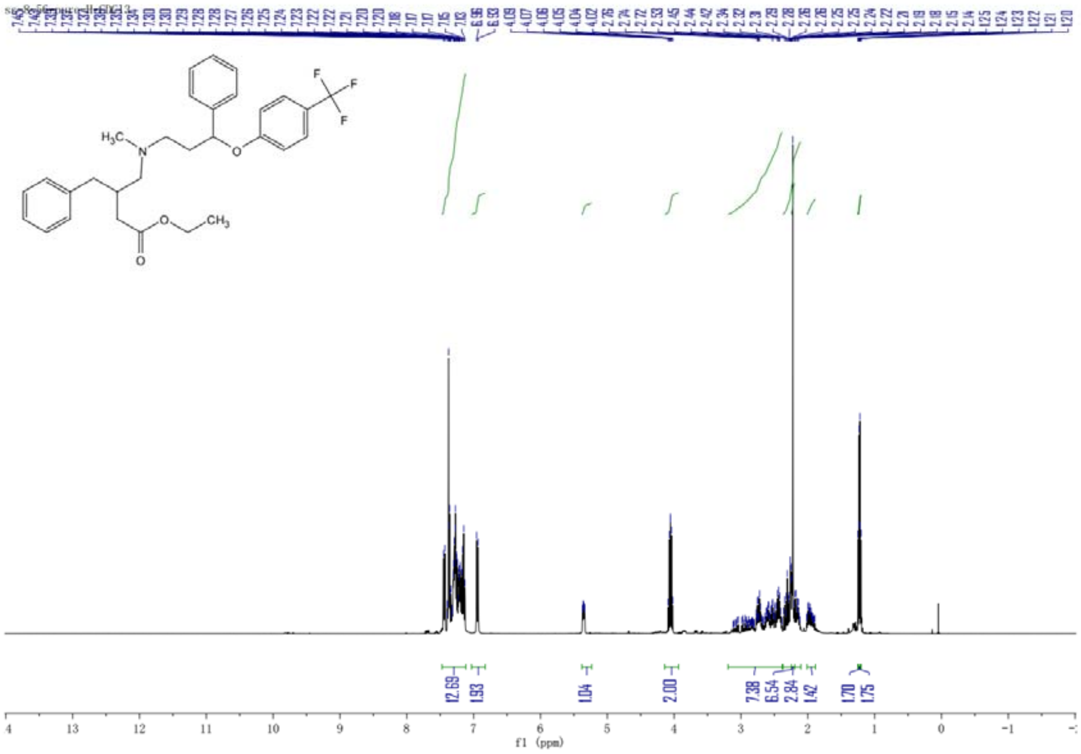

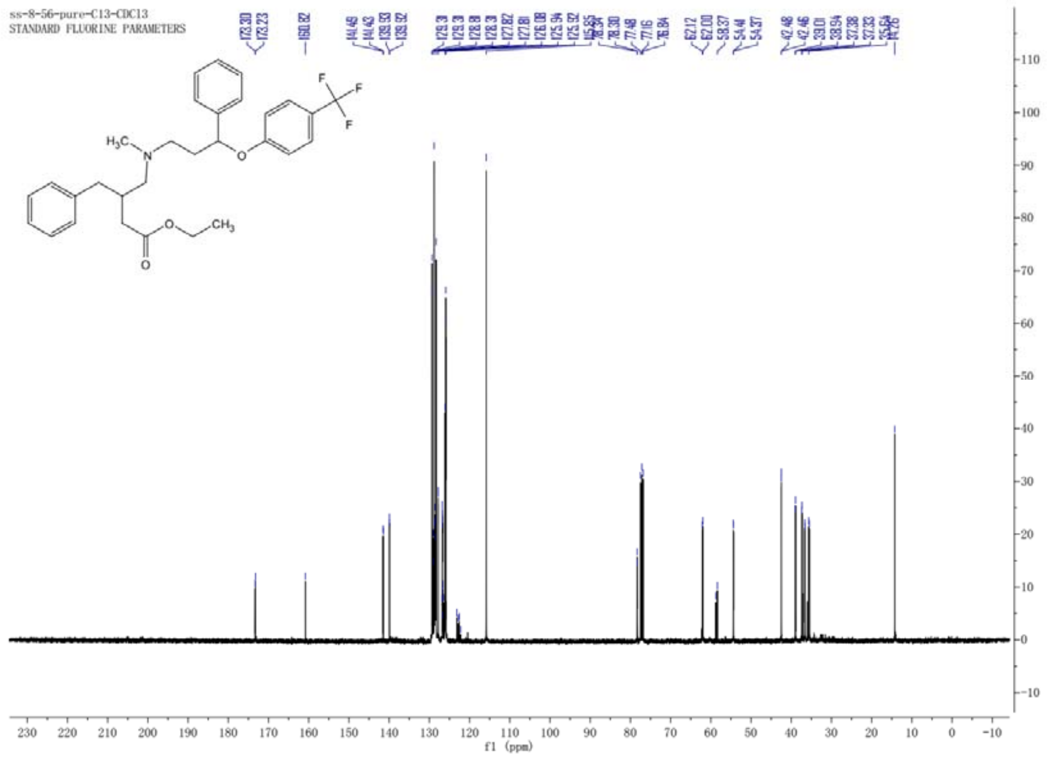
ss-8-56-pure-C13-CDCl3
STANDARD FLUORINE PARAMETERS
f1 (ppm)

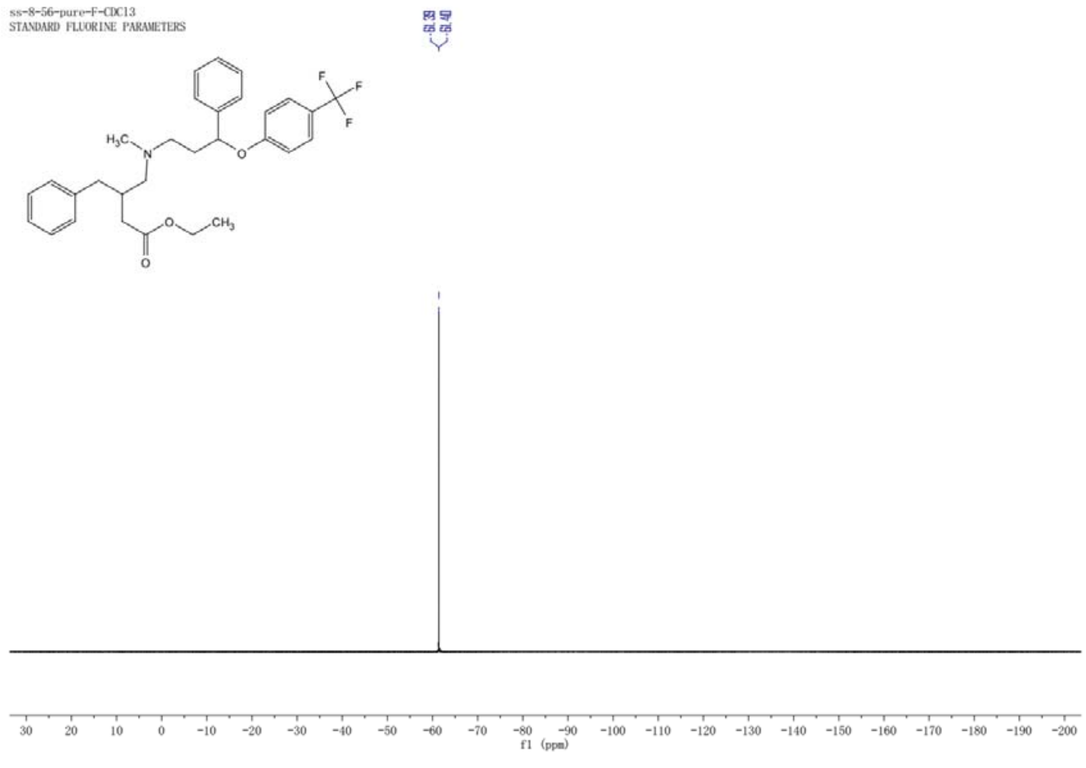
ss-8-56-pure-F-CDCl3
STANDARD FLUORINE PARAMETERS
f1 (ppm)

74

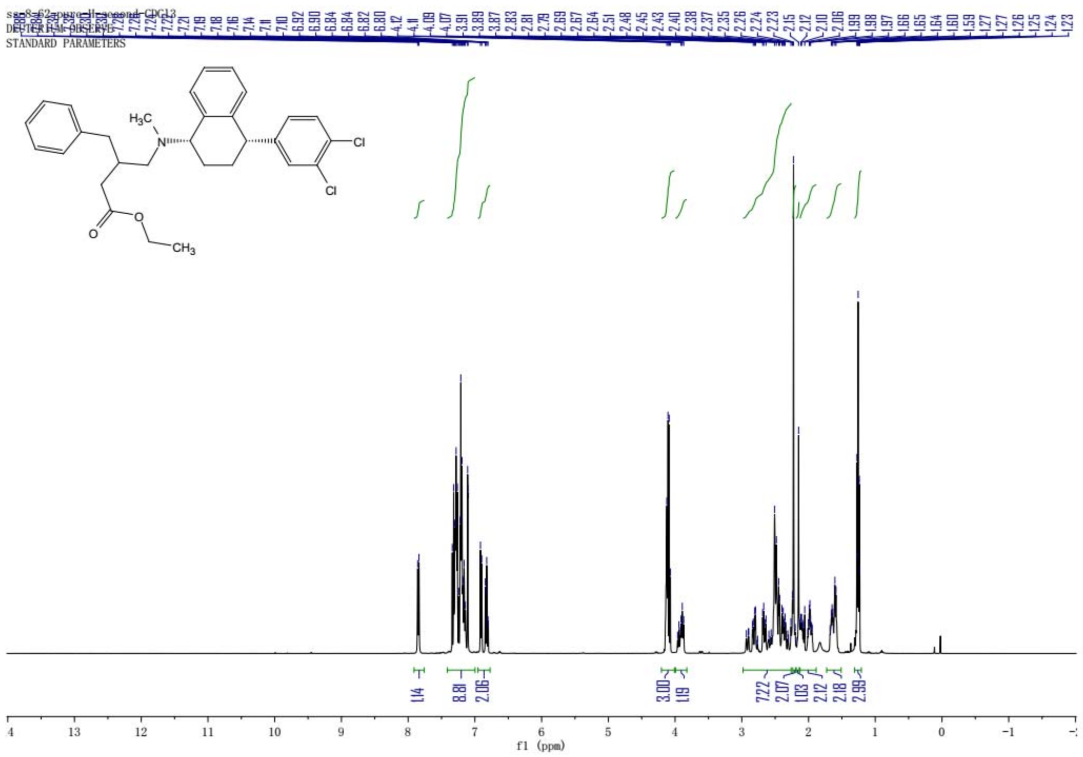
DEUTERIUM OBSERVE
STANDARD PARAMETERS
f1 (ppm)

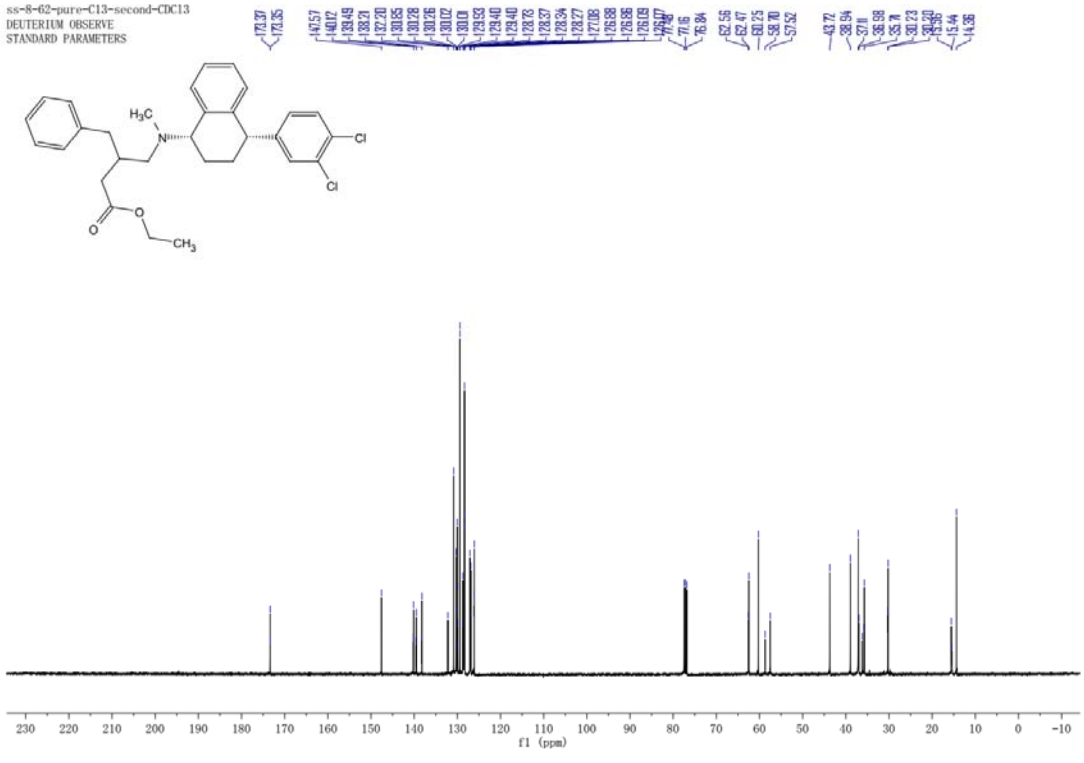
ss-8-62-pure-C13-second-CDC13
DEUTERIUM OBSERVE
STANDARD PARAMETERS
f1 (ppm)

75

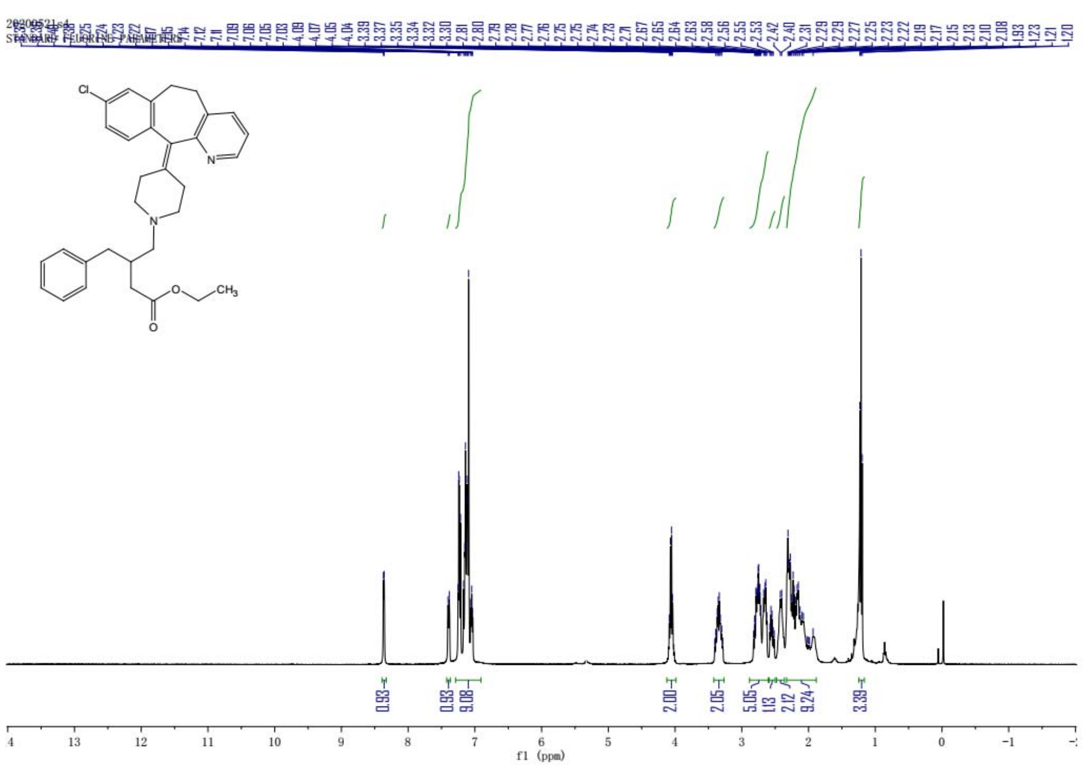

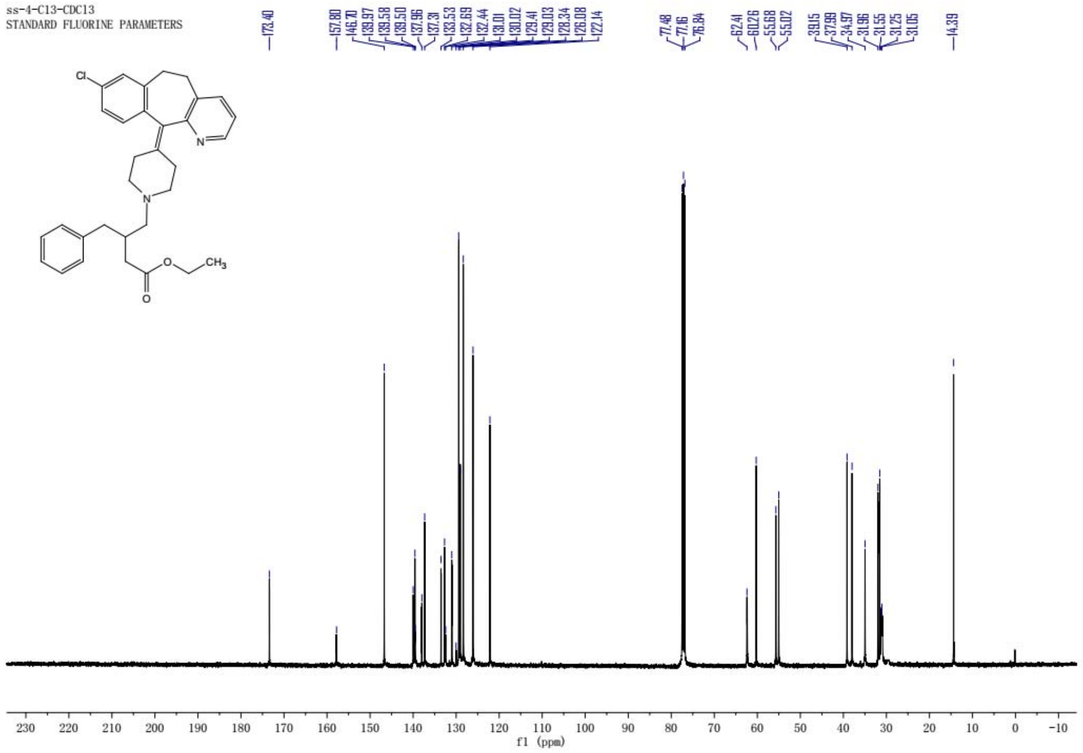

76

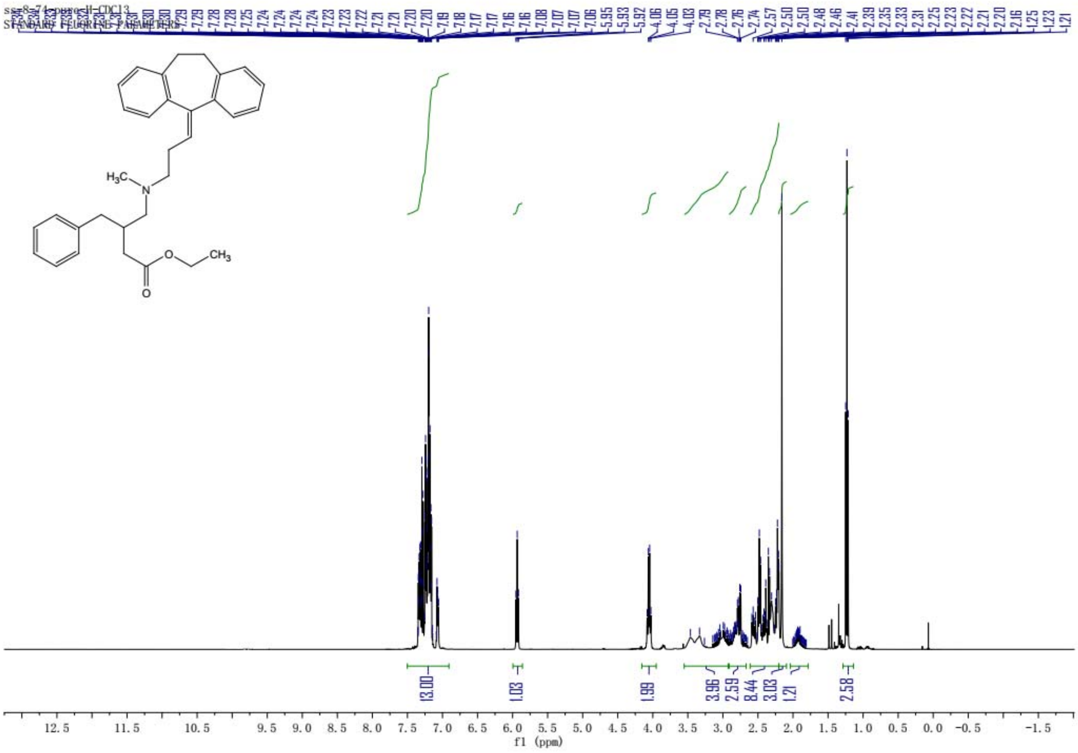
ss-8-74-pure-H-CDCl3
STANDARD FLUORINE PARAMETERS
H3C
N
O
CH3
O
13.00
1.03
1.99
3.96
2.59
8.44
3.03
1.21
2.58
f1 (ppm)

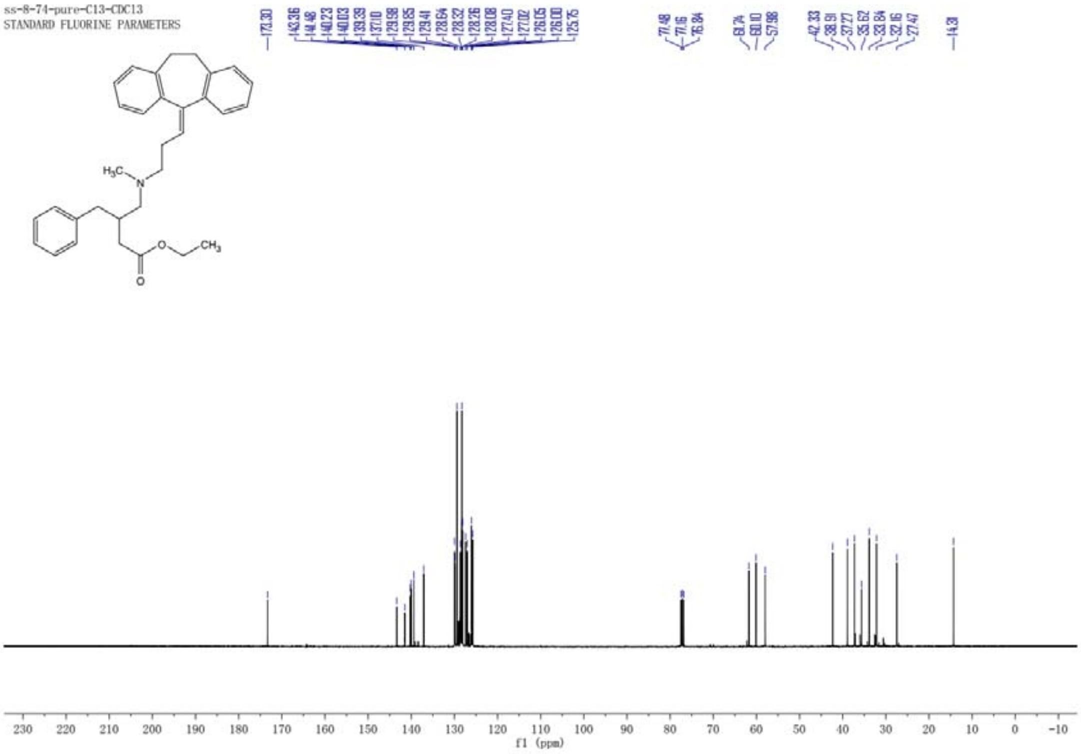
ss-8-74-pure-C13-CDCl3
STANDARD FLUORINE PARAMETERS
173.30
143.36
141.48
140.23
140.03
139.39
137.10
129.98
129.85
129.41
128.64
128.32
128.26
128.08
127.40
127.02
126.05
126.00
125.75
77.48
77.16
76.84
61.74
60.10
57.98
42.33
38.91
37.27
35.62
33.84
32.16
27.47
14.31
H3C
N
O
CH3
O
f1 (ppm)

77

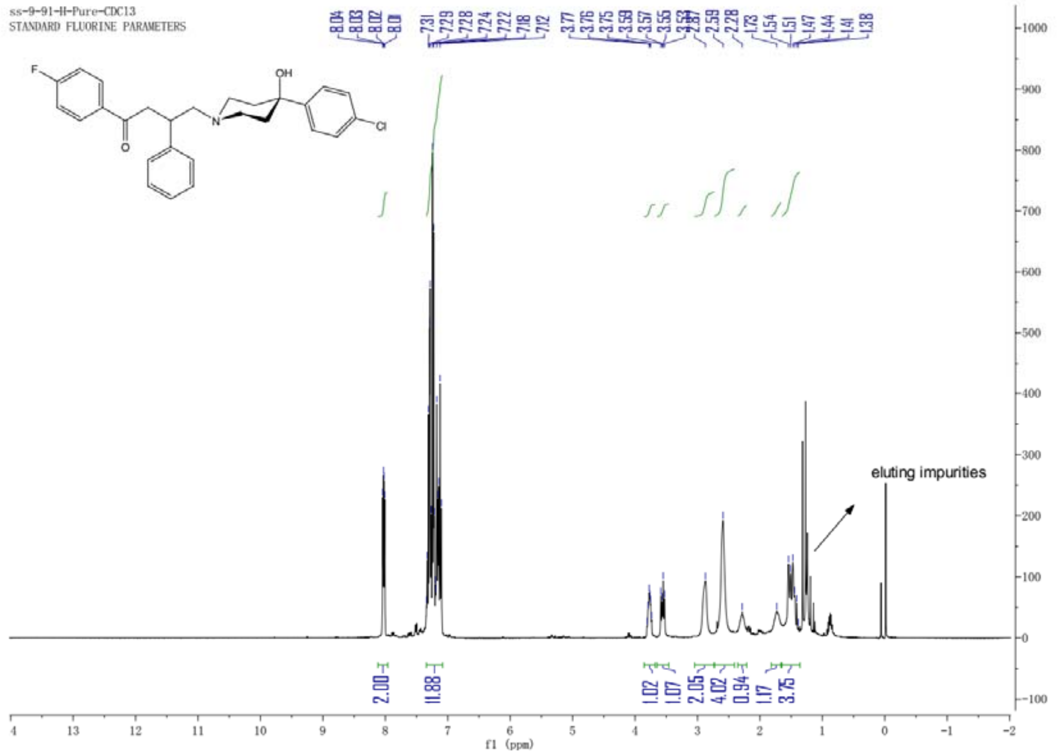

ss-9-91-H-Pure-CDCl3
STANDARD FLUORINE PARAMETERS
eluting impurities
f1 (ppm)

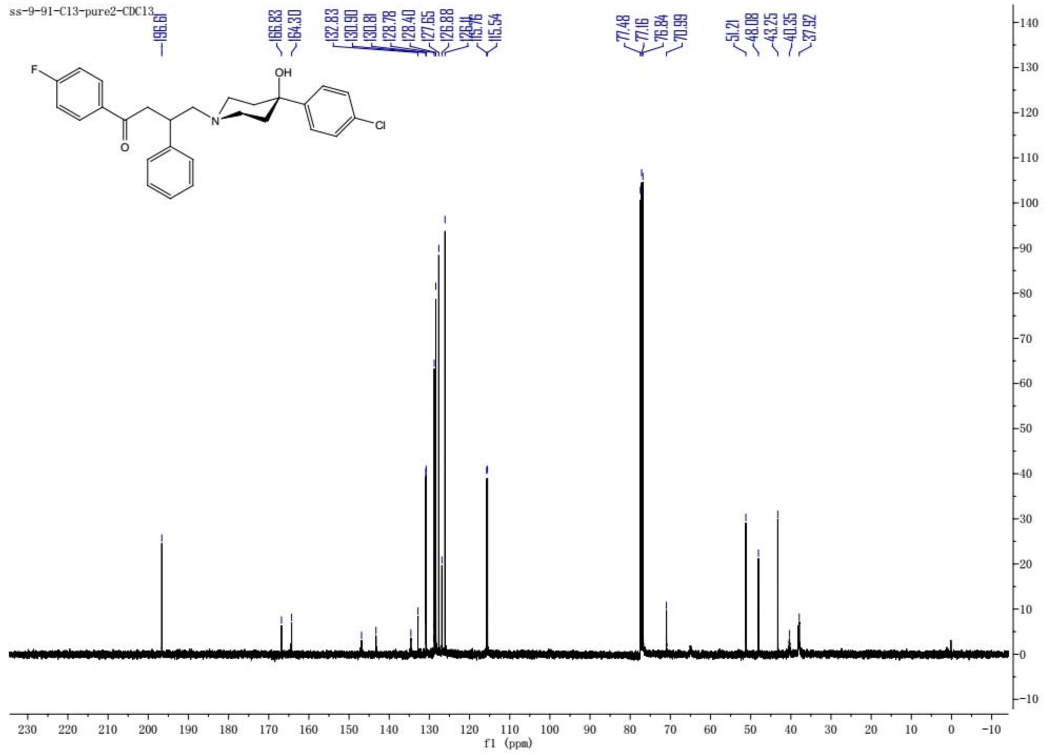

ss-9-91-C13-pure2-CDCl3
f1 (ppm)

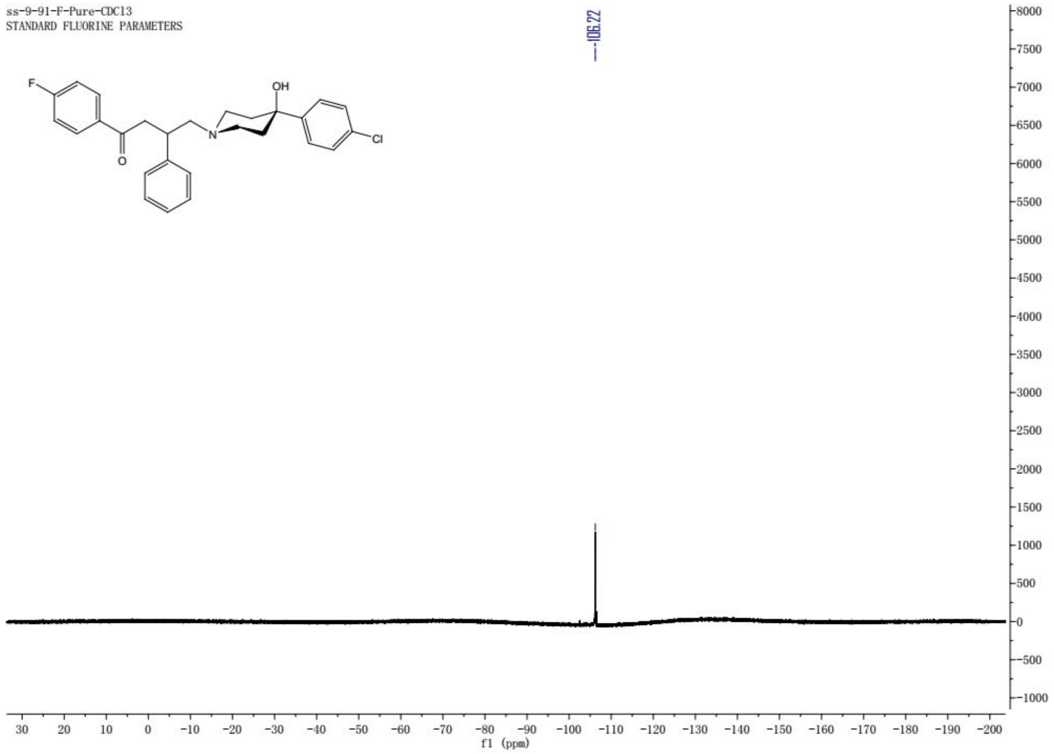
ss-9-91-F-Pure-CDCl3
STANDARD FLUORINE PARAMETERS
-106.22
f1 (ppm)

79

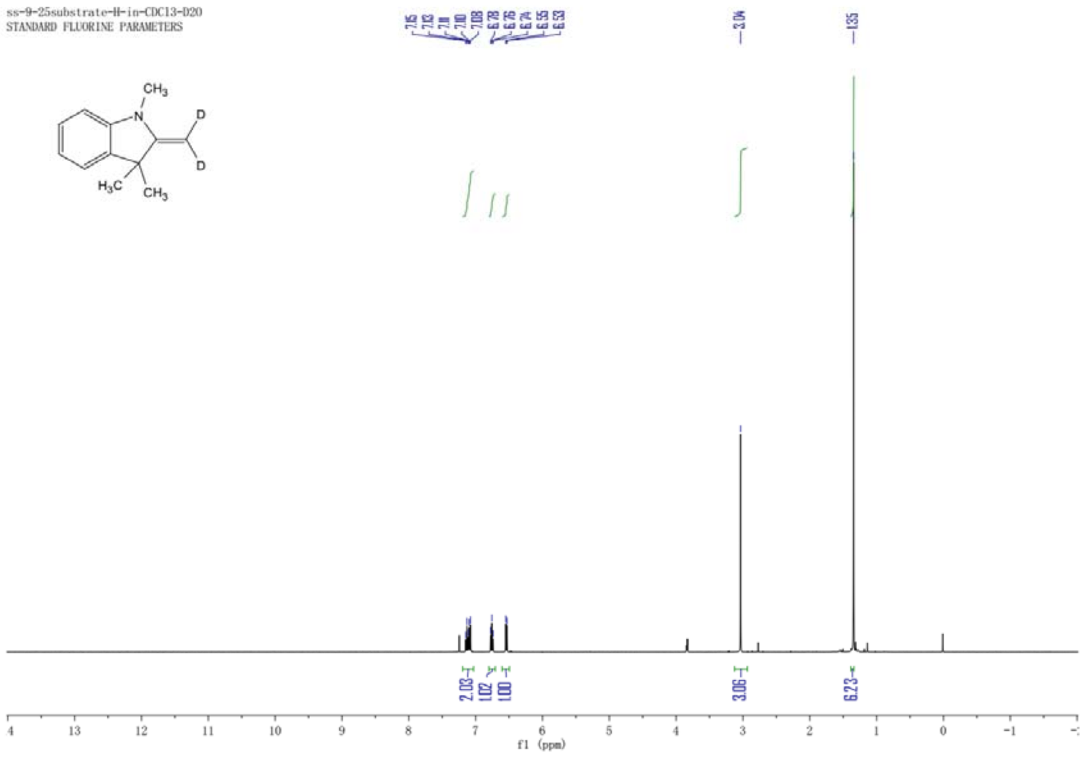
ss-9-25substrate-H-in-CDCl3-D2O
STANDARD FLUORINE PARAMETERS
3.04
1.35
2.03
1.02
1.00
3.06
6.23
f1 (ppm)

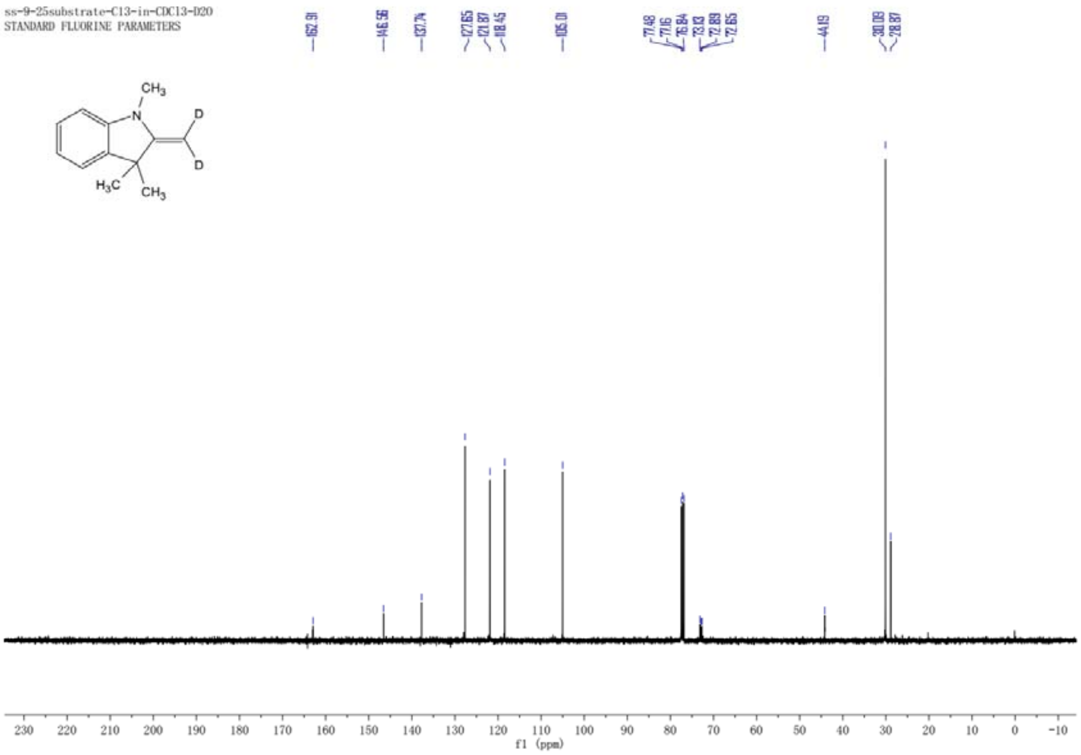

80a

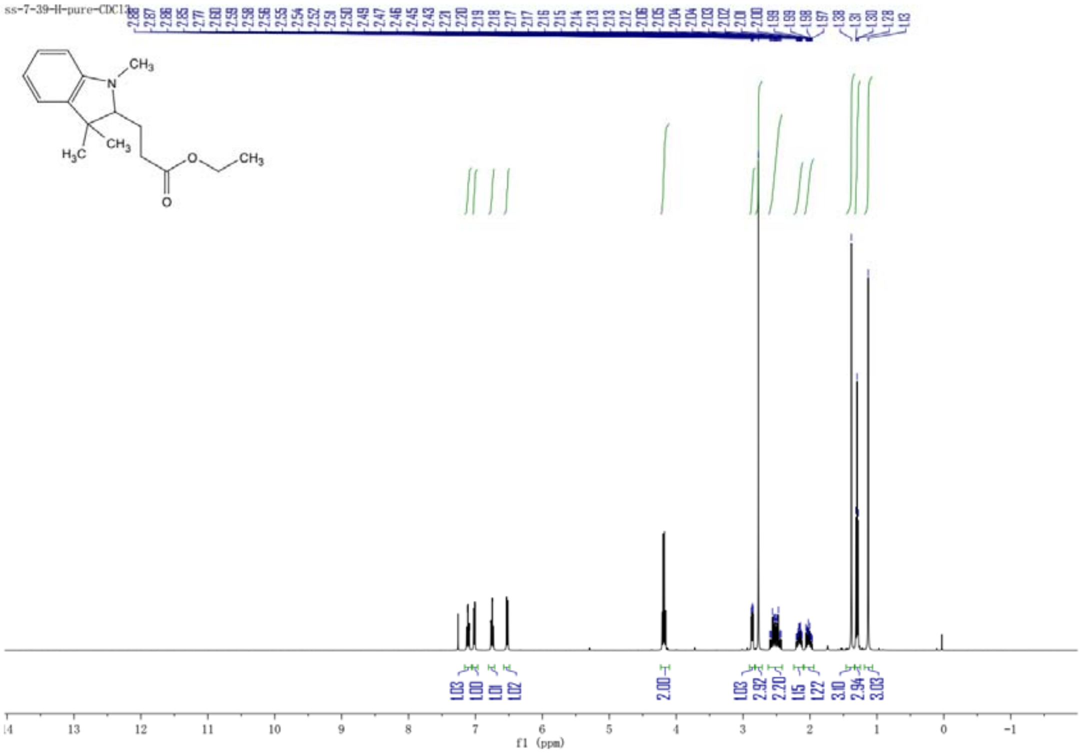

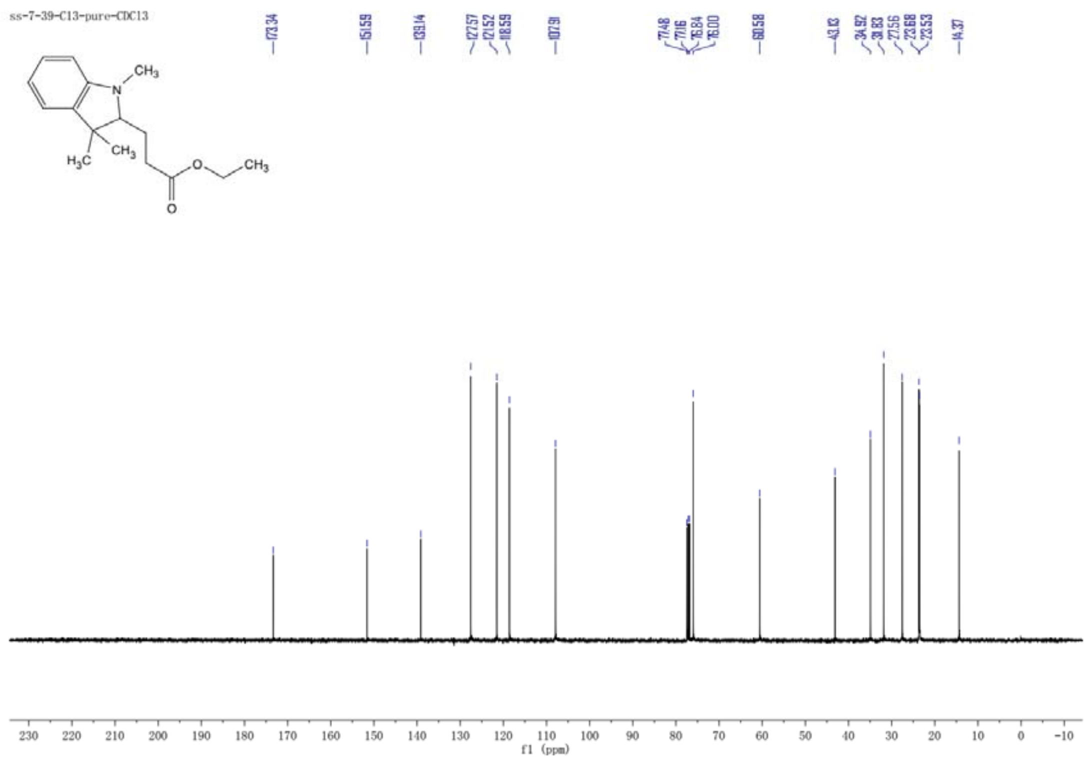

80

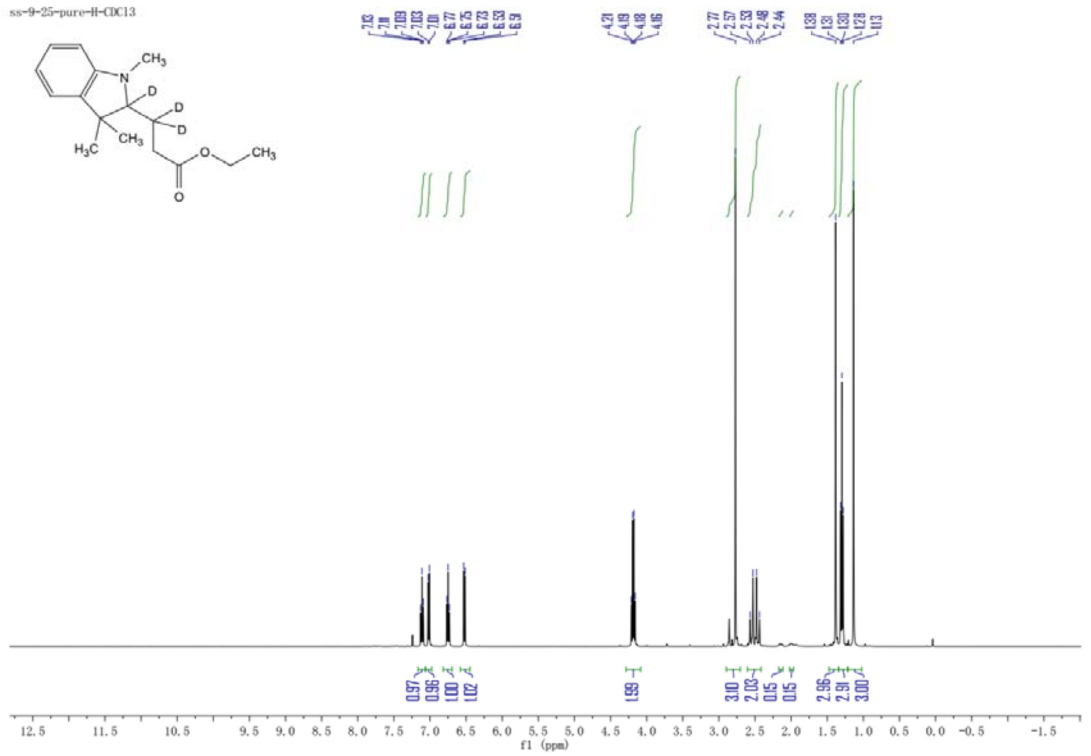

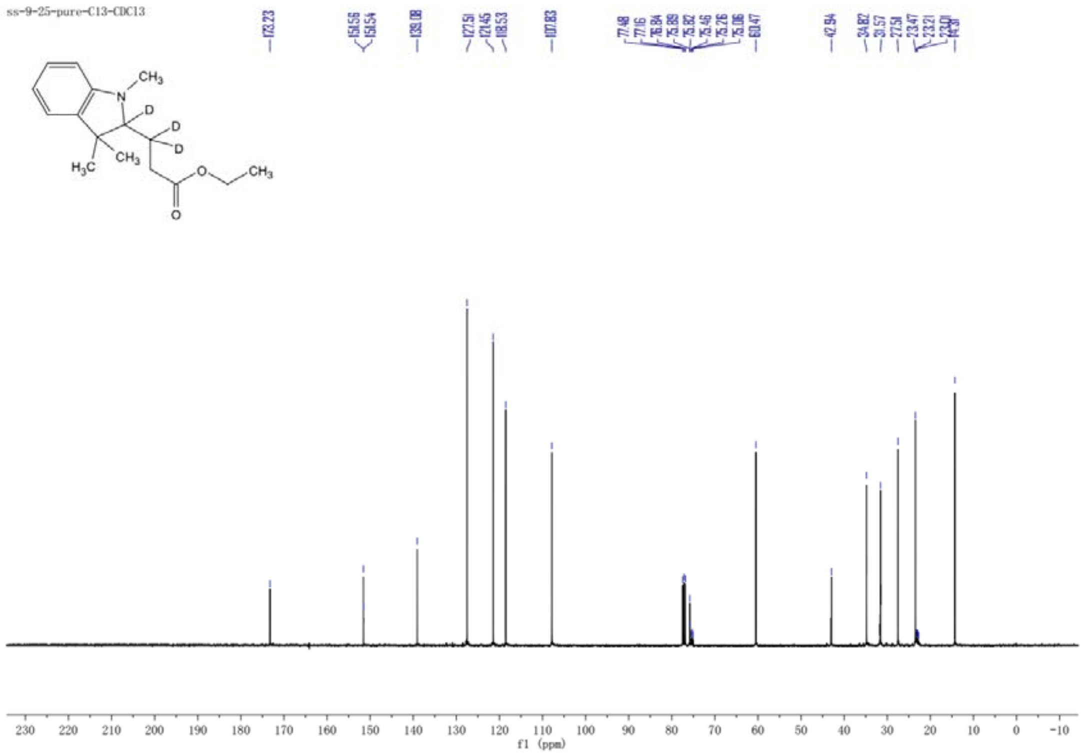

a

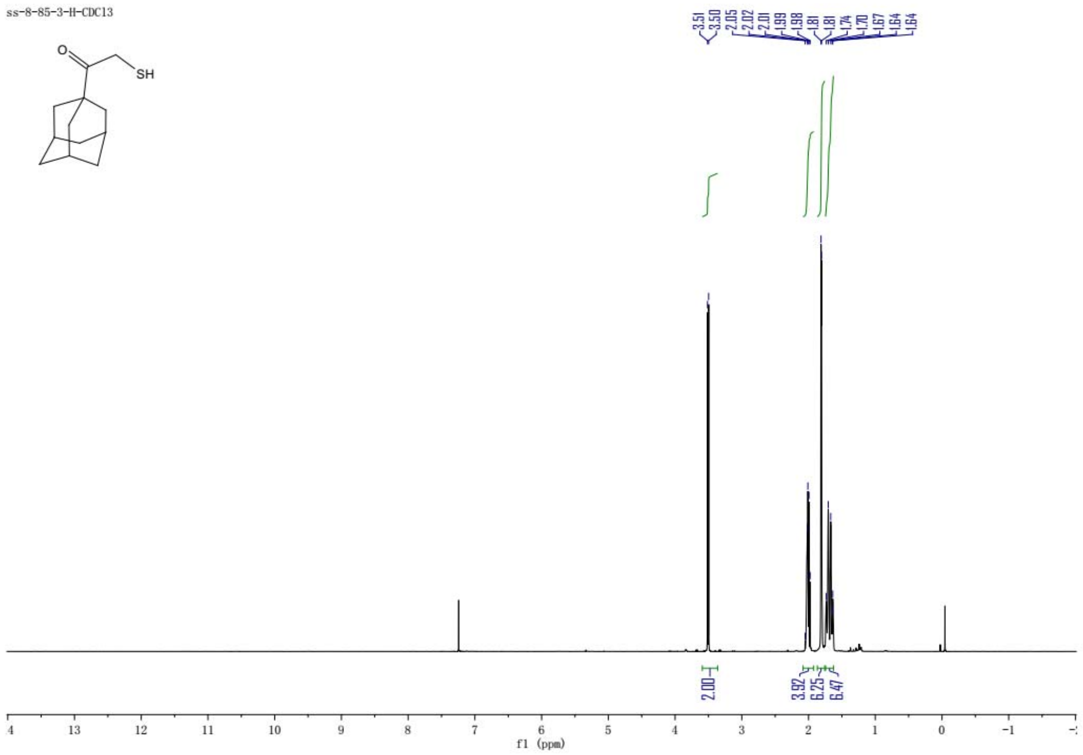

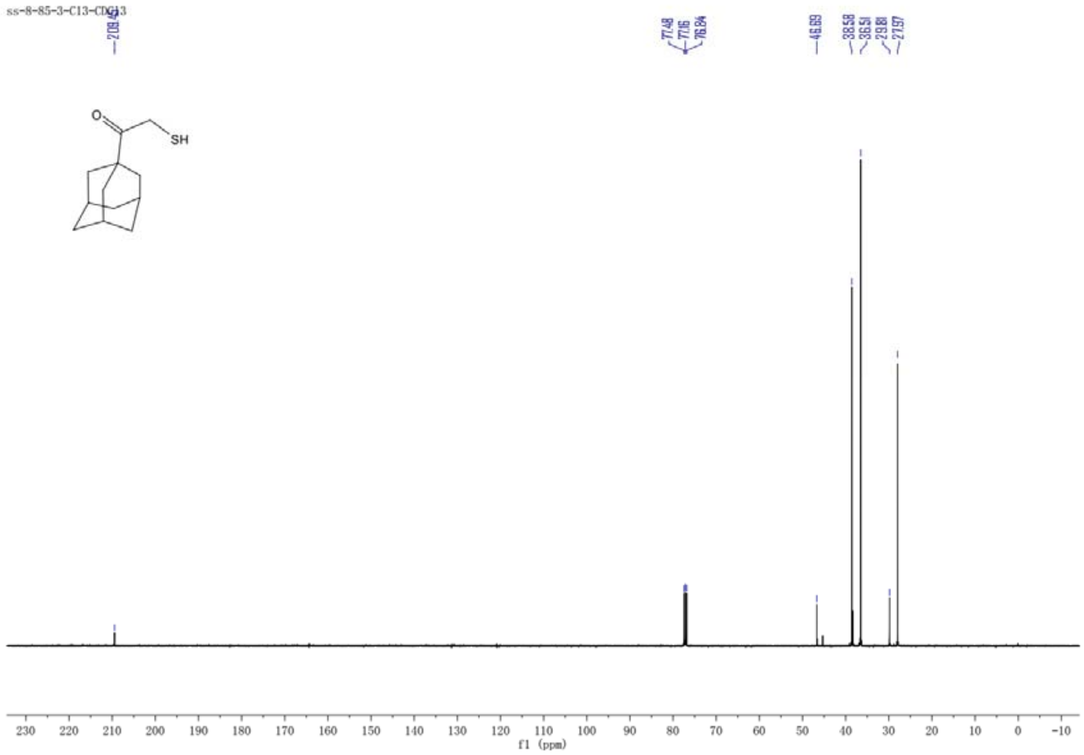
ss-8-85-3-C13-CDCl3
77.48
77.16
76.84
46.69
38.58
36.51
29.81
27.97
SH
O
f1 (ppm)

b

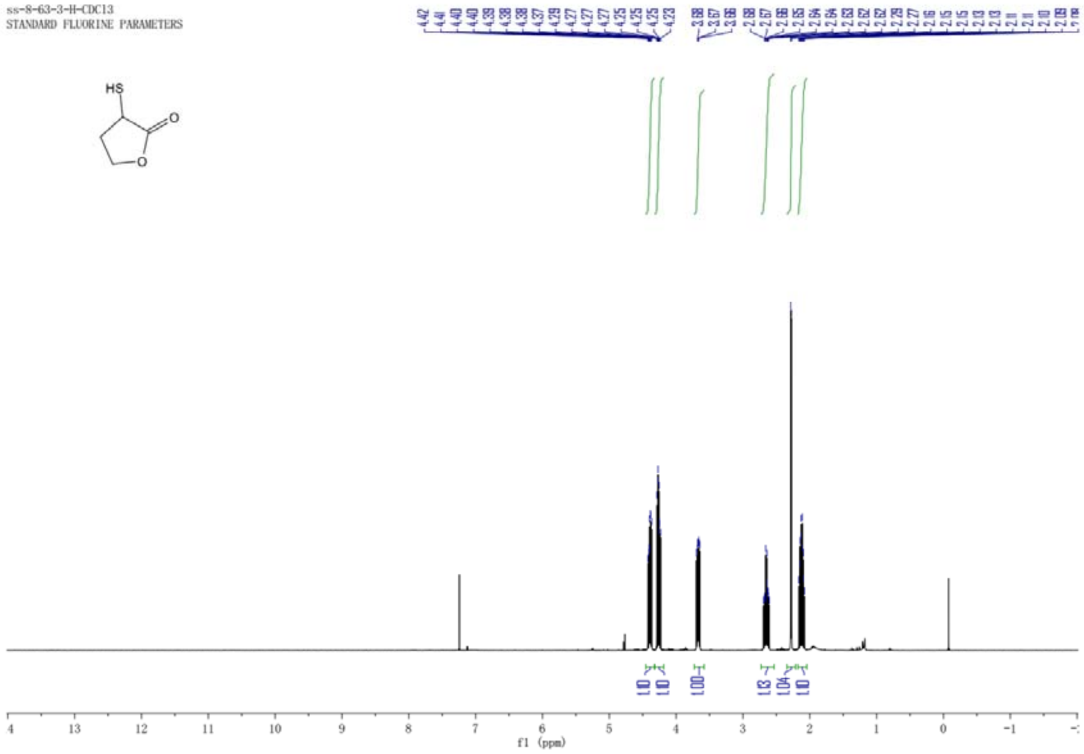
ss-8-63-3-H-CDCl3
STANDARD FLUORINE PARAMETERS
HS
O
O
f1 (ppm)

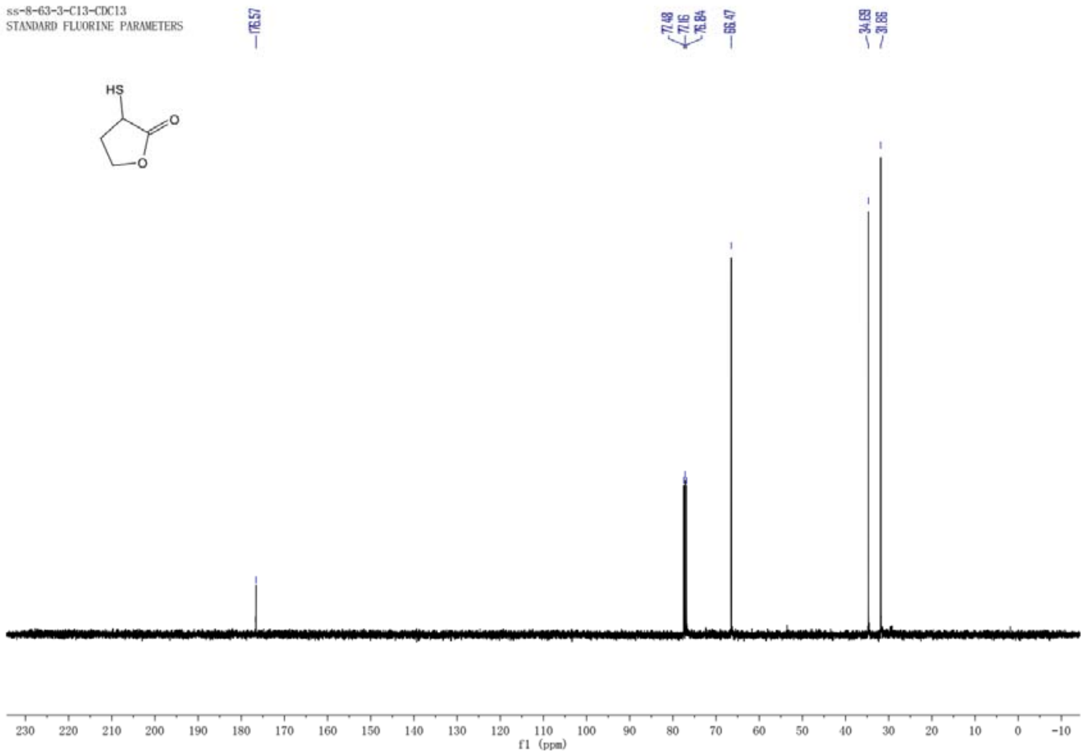
ss-8-63-3-C13-CDCl3
STANDARD FLUORINE PARAMETERS
176.57
77.48
77.16
76.84
66.47
34.69
31.66
HS
O
O
f1 (ppm)

c

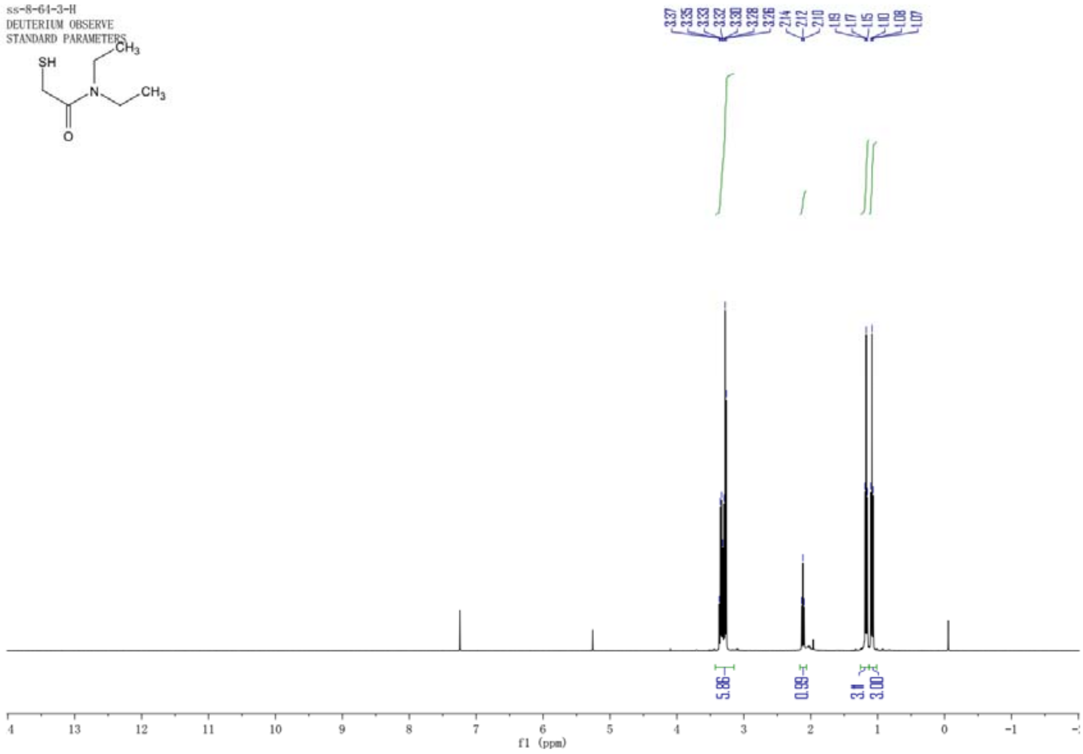
ss-8-64-3-H
DEUTERIUM OBSERVE
STANDARD PARAMETERS
SH
N
CH3
CH3
O
5.86
0.99
3.11
3.00
f1 (ppm)

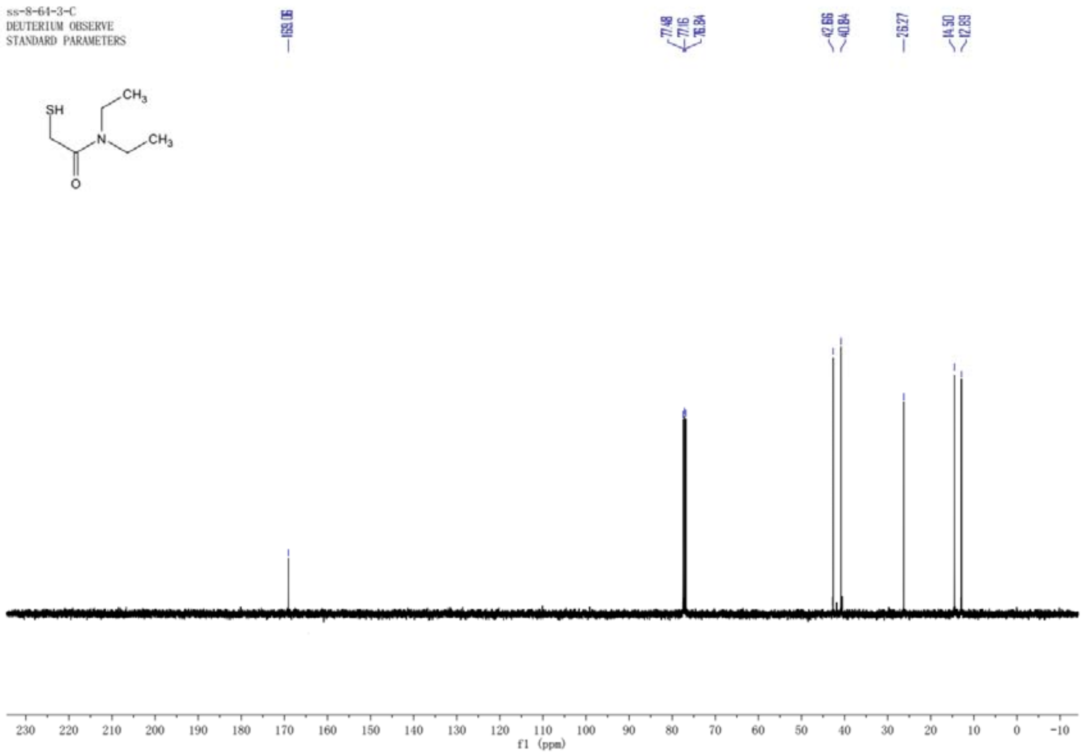
ss-8-64-3-C
DEUTERIUM OBSERVE
STANDARD PARAMETERS
169.06
77.48
77.16
76.84
42.66
40.84
26.27
14.50
12.89
SH
CH3
N
CH3
O
230
220
210
200
190
180
170
160
150
140
130
120
110
100
90
80
70
60
50
40
30
20
10
0
-10
f1 (ppm)

d

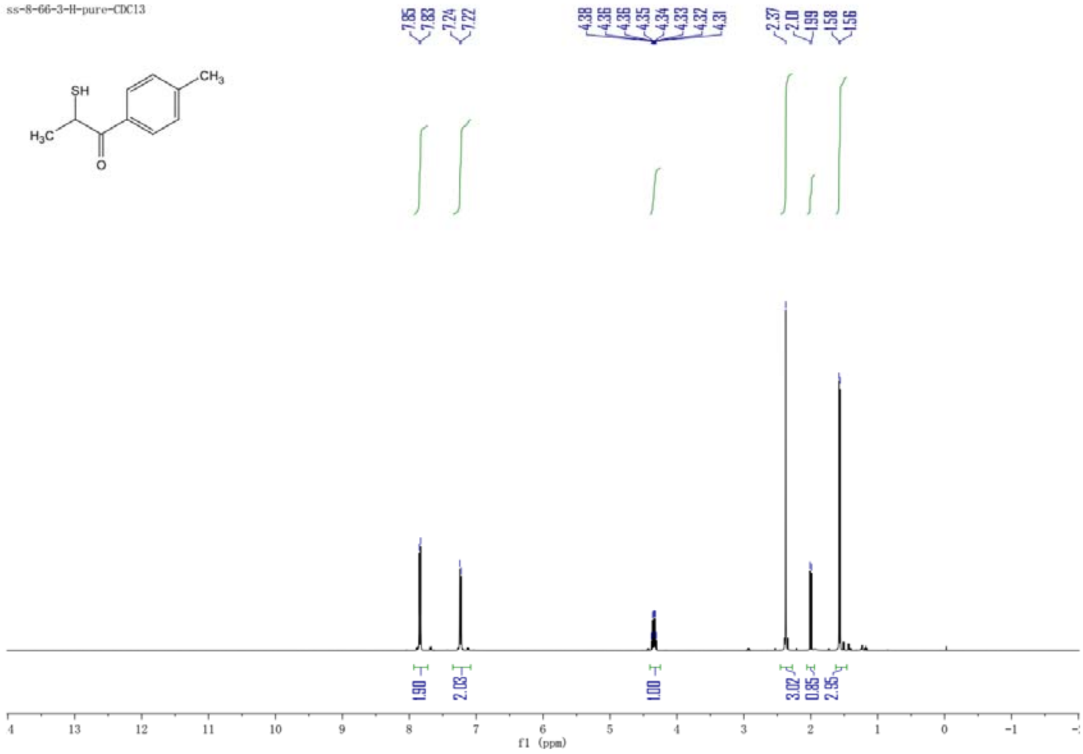
ss-8-66-3-H-pure-CDCl3
7.85
7.83
7.24
7.22
4.38
4.36
4.36
4.35
4.34
4.33
4.32
4.31
2.37
2.01
1.99
1.58
1.56
SH
CH3
H3C
O
1.90
2.03
1.00
3.02
0.85
2.95
13
12
11
10
9
8
7
6
5
4
3
2
1
0
-1
f1 (ppm)

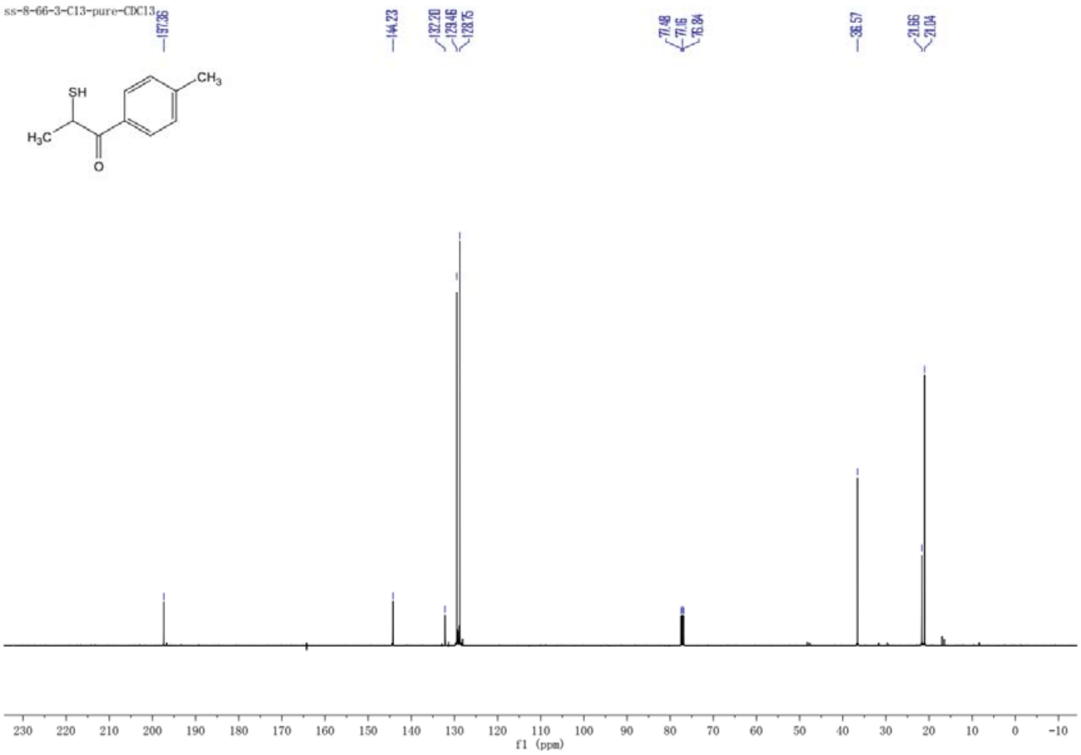
ss-8-66-3-C13-pure-CDCl3
197.36
144.23
132.20
129.46
128.75
77.48
77.16
76.84
36.57
21.66
21.04
SH
CH3
H3C
O
230 220 210 200 190 180 170 160 150 140 130 120 110 100 90 80 70 60 50 40 30 20 10 0 -10
f1 (ppm)

e

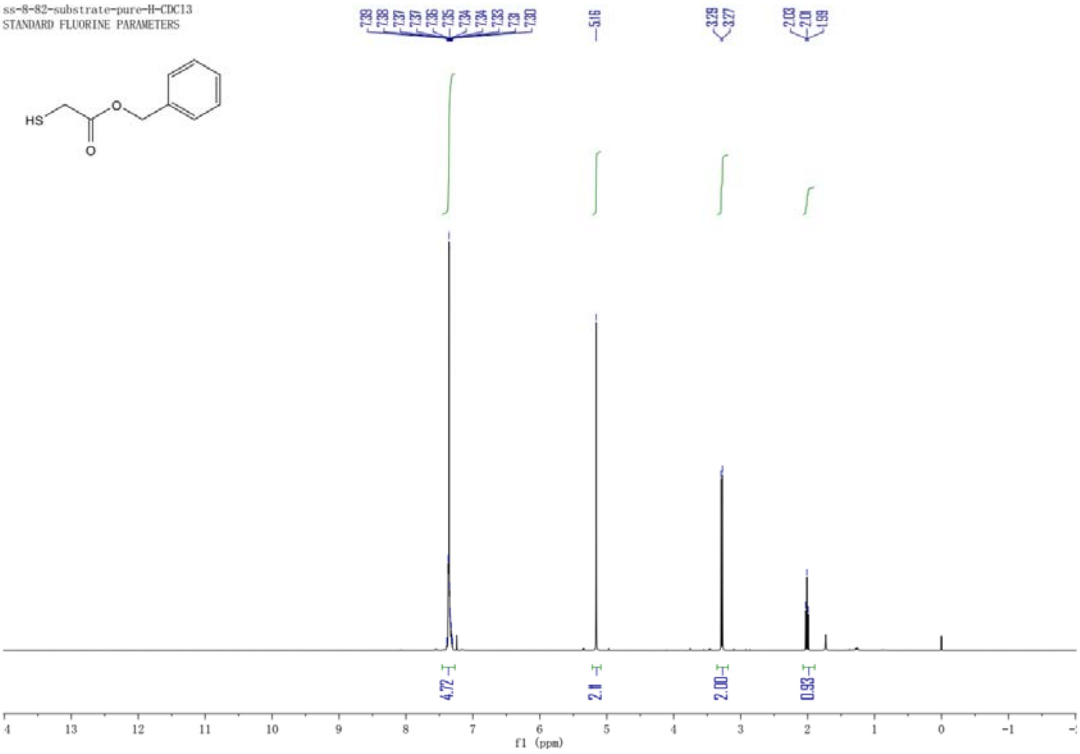
ss-8-82-substrate-pure-H-CDCl3
STANDARD FLUORINE PARAMETERS
7.39
7.38
7.37
7.37
7.36
7.35
7.34
7.34
7.33
7.31
7.30
5.16
3.29
3.27
2.03
2.01
1.99
HS
O
O
4.72
2.11
2.00
0.93
13 12 11 10 9 8 7 6 5 4 3 2 1 0 -1
f1 (ppm)

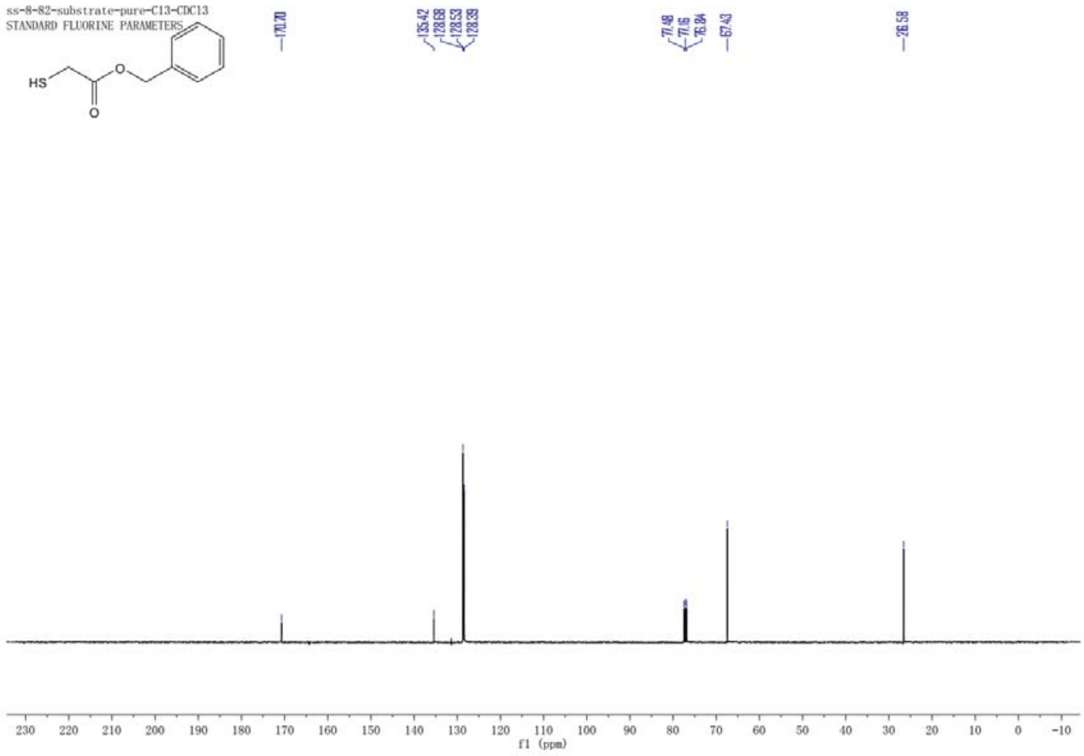
ss-8-82-substrate-pure-C13-CDC13
STANDARD FLUORINE PARAMETERS
HS
O
O
170.70
135.42
128.68
128.53
128.39
77.48
77.16
76.84
67.43
26.58
230 220 210 200 190 180 170 160 150 140 130 120 110 100 90 80 70 60 50 40 30 20 10 0 -10
f1 (ppm)

f

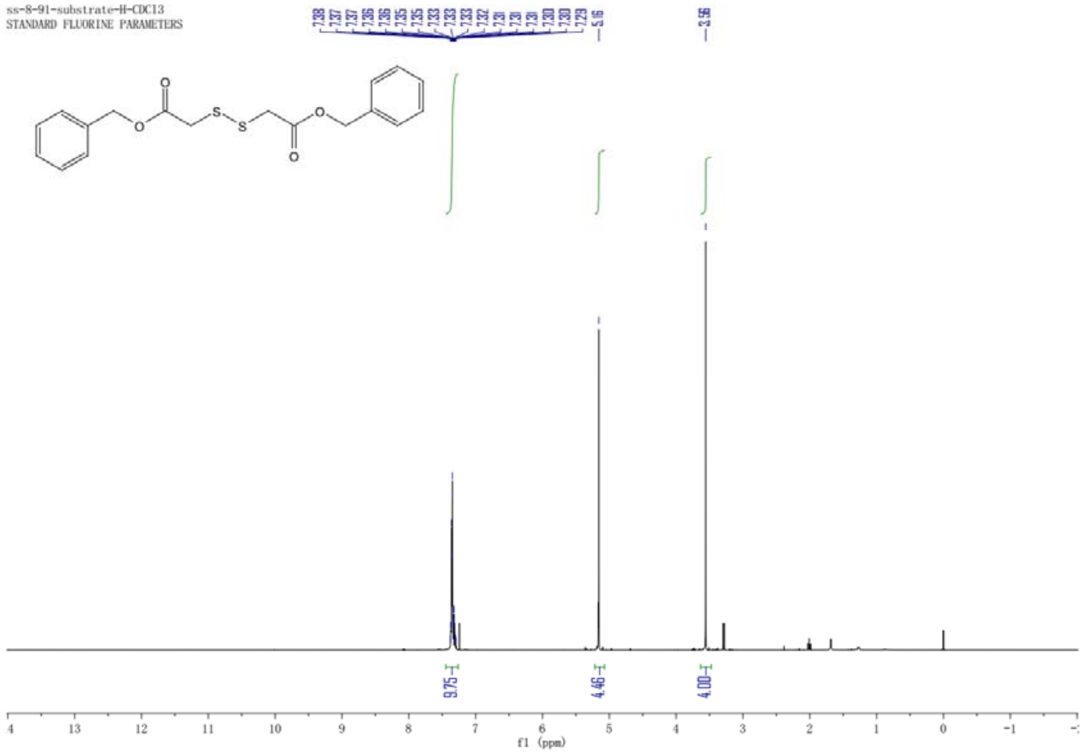
ss-8-91-substrate-H-CDC13
STANDARD FLUORINE PARAMETERS
O
O
S
S
O
O
7.38
7.37
7.37
7.36
7.36
7.35
7.35
7.33
7.33
7.33
7.32
7.31
7.31
7.31
7.30
7.30
7.29
5.16
3.56
9.75
4.46
4.00
13 12 11 10 9 8 7 6 5 4 3 2 1 0 -1
f1 (ppm)

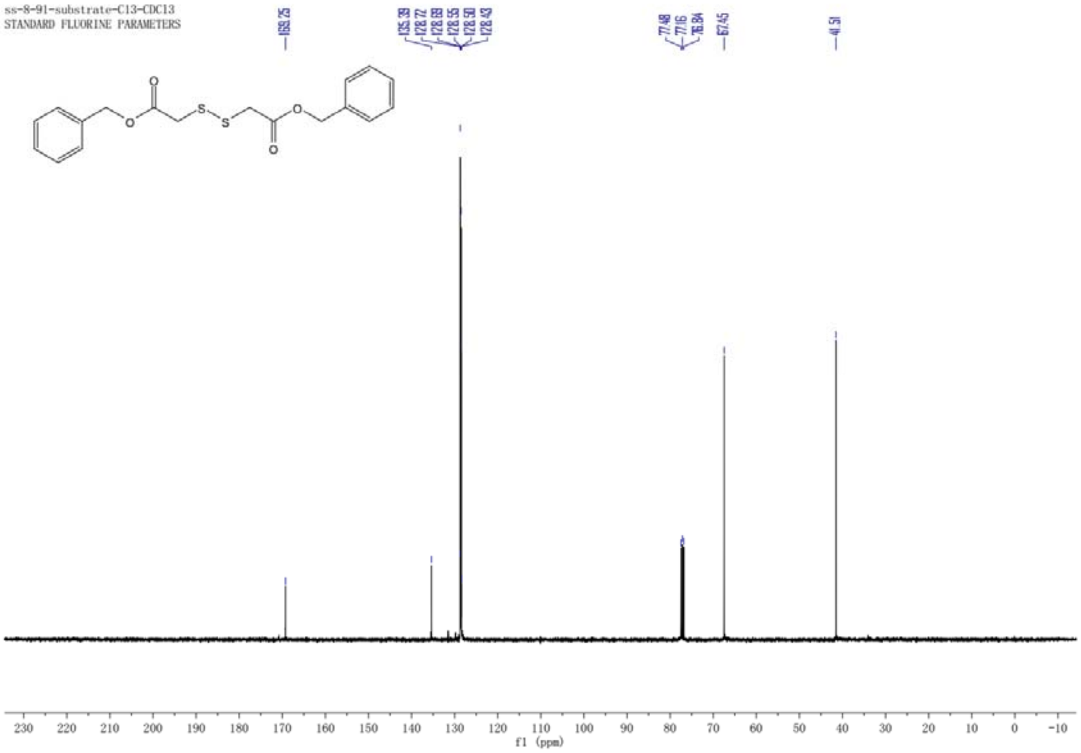
ss-8-91-substrate-C13-CDCl3
STANDARD FLUORINE PARAMETERS
f1 (ppm)

h

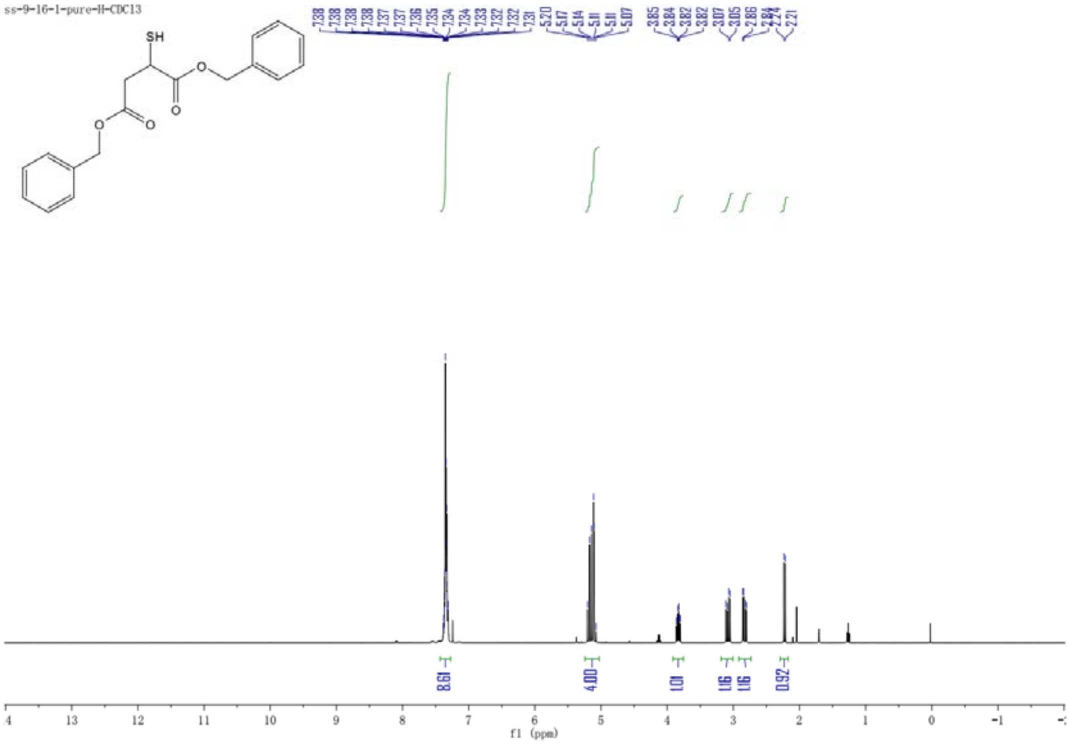
ss-9-16-1-pure-H-CDCl3
SH
f1 (ppm)

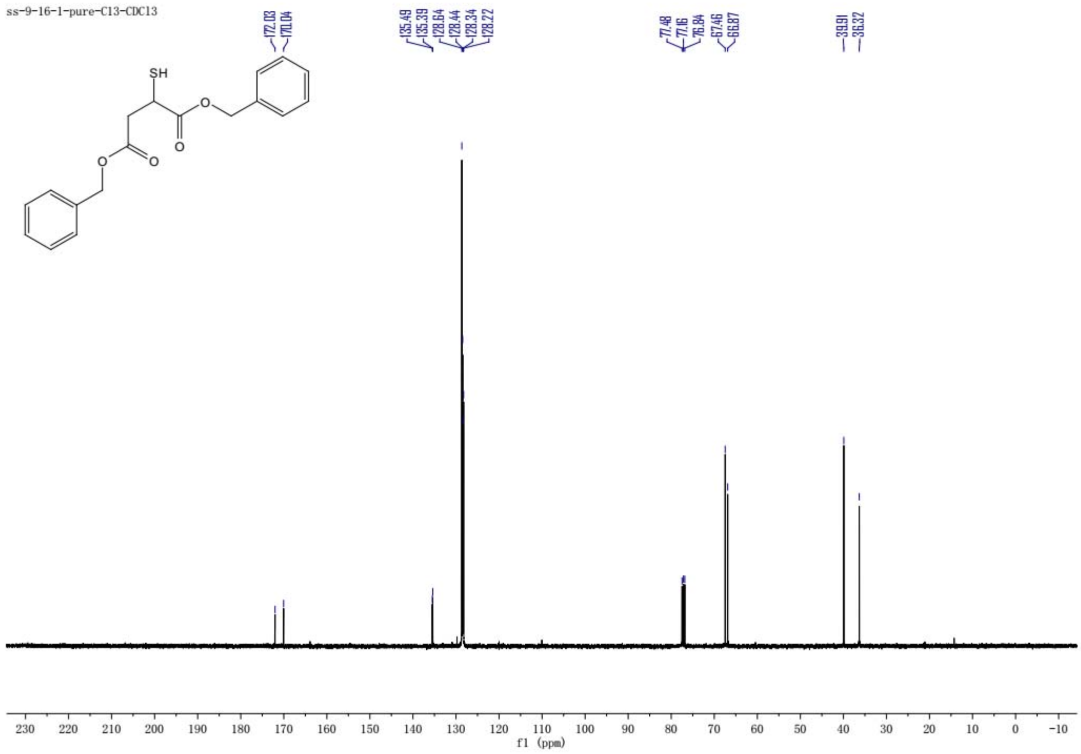
ss-9-16-1-pure-C13-CDCl3
172.03
171.04
135.49
135.39
128.64
128.44
128.34
128.22
77.48
77.16
76.84
67.46
66.87
39.91
36.32
SH
O
230 220 210 200 190 180 170 160 150 140 130 120 110 100 90 80 70 60 50 40 30 20 10 0 -10
f1 (ppm)

9. Supplementary References

1. Still, W. C., Kahn, M. & Mitra, A. Rapid chromatographic technique for preparative separations with moderate resolution. *J. Org. Chem.* **43**, 2923-2925 (1978).
2. Lopp, J. M. & Schmidt, V. A. Intermolecular Phosphite-Mediated Radical Desulfurative Alkene Alkylation Using Thiols. *Org. Lett.* **21**, 8031-8036 (2019).
3. Benati, L., Leardini, R., Minozzi, M., Nanni, D., Scialpi, R., Spagnolo, P., Strazzari, S. & Zanardi, G. A Novel Tin‐Free Procedure for Alkyl Radical Reactions. *Angew. Chem. Int. Ed.* **43**, 3598-3601 (2004).
4. Dardonville, C., Rinaldi, E., Barrett, M. P., Brun, R., Gilbert, I. H. & Hanau, S. Selective inhibition of Trypanosoma brucei 6-phosphogluconate dehydrogenase by high-energy intermediate and transition-state analogues. *J. Med. Chem.* **47**, 3427-3437 (2004).
5. Zhang, Y.-Z., Zhu, S.-F., Cai, Y., Mao, H.-X. & Zhou, Q.-L. Copper-catalyzed enantioselective carbenoid insertion into S–H bonds. *Chem. Commun.* **36**, 5362-5364 (2009).
